# In Silico Discovery of Small Molecule Modulators Targeting the Achilles’ Heel of SARS-CoV-2 Spike Protein

**DOI:** 10.1021/acscentsci.2c01190

**Published:** 2023-02-08

**Authors:** Qing Wang, Fanhao Meng, Yuting Xie, Wei Wang, Yumin Meng, Linjie Li, Tao Liu, Jianxun Qi, Xiaodan Ni, Sanduo Zheng, Jianhui Huang, Niu Huang

**Affiliations:** † School of Pharmaceutical Science and Technology, Tianjin University, Tianjin 300072, China; ‡ National Institute of Biological Sciences, Beijing, Zhongguancun Life Science Park, No. 7 Science Park Road, Beijing 102206, China; § Shuimu Biosciences, Zhongguancun Life Science Park, No. 7 Science Park Road, Beijing 102206, China; ∥ CAS Key Laboratory of Pathogen Microbiology and Immunology, Institute of Microbiology, Chinese Academy of Sciences, Beijing 100101, China; ⊥ Tsinghua Institute of Multidisciplinary Biomedical Research, Tsinghua University, Beijing 102206, China

## Abstract

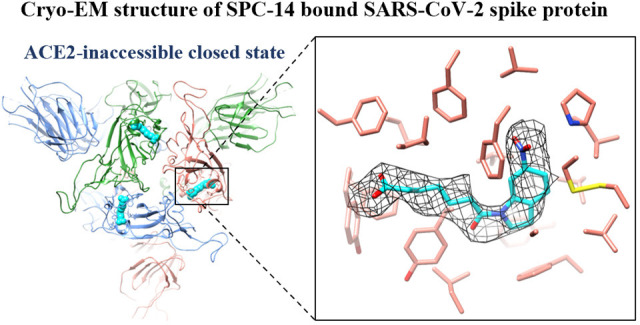

The spike protein of SARS-CoV-2 has been a promising target for developing vaccines and therapeutics due to its crucial role in the viral entry process. Previously reported cryogenic electron microscopy (cryo-EM) structures have revealed that free fatty acids (FFA) bind with SARS-CoV-2 spike protein, stabilizing its closed conformation and reducing its interaction with the host cell target in vitro. Inspired by these, we utilized a structure-based virtual screening approach against the conserved FFA-binding pocket to identify small molecule modulators of SARS-CoV-2 spike protein, which helped us identify six hits with micromolar binding affinities. Further evaluation of their commercially available and synthesized analogs enabled us to discover a series of compounds with better binding affinities and solubilities. Notably, our identified compounds exhibited similar binding affinities against the spike proteins of the prototypic SARS-CoV-2 and a currently circulating Omicron BA.4 variant. Furthermore, the cryo-EM structure of the compound SPC-14 bound spike revealed that SPC-14 could shift the conformational equilibrium of the spike protein toward the closed conformation, which is human ACE2 (hACE2) inaccessible. Our identified small molecule modulators targeting the conserved FFA-binding pocket could serve as the starting point for the future development of broad-spectrum COVID-19 intervention treatments.

## Abstract 2

Computationally identified compound SPC-14 stabilizes the ACE2-inaccessible closed conformation of the SARS-CoV-2 spike protein by targeting a less mutation-prone free fatty acids-binding pocket.

## Introduction

**Figure 1:**
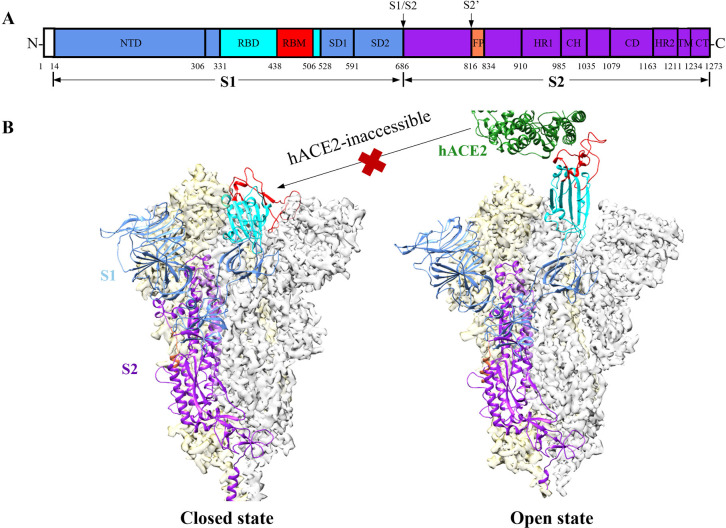
Overall structures of the prefusion SARS-CoV-2 spike protein in different conformational states. (A) Schematic diagram of SARS-CoV-2 spike protein monomer representing domain organization. NTD: N-terminal domain; RBD: receptor-binding domain; RBM: receptor-binding motif; SD1: subdomain 1; SD2: subdomain 2; FP: fusion peptide; HR1: heptad repeat 1; CH: central helix; CD: connector domain; HR2: heptad repeat 2; TM: transmembrane domain; CT: cytoplasmic tail. (B) Cryo-EM structures of spike protein with the closed state (left, PDB ID: 6XR8) and the open state (right, PDB ID: 7A94). One protomer of spike protein is represented as ribbons, and the other two protomers are displayed as density maps. All the molecular images are rendered in UCSF Chimera.^7^

Like other β-coronaviruses, SARS-CoV-2 utilizes the trimeric spike glycoprotein on its envelope to mediate viral entry into host cells. Each protomer of spike protein in the extracellular domain is composed of S1 and S2 subunits (Figure 1A), which are responsible for binding to host cell targets and the membrane fusion process, respectively.^1,2^ It has been revealed that the prefusion spike protein mainly includes two conformational states: the closed state with all three “down” receptor-binding domains (RBDs) in the S1 subunit, and the open state with one or more “up” RBDs (Figure 1B).^2^ During viral entry, only the “up” RBD could bind to the host cell target angiotensin-converting enzyme 2 (ACE2).^2−4^ Cryo-EM studies have suggested that ACE2 binding destabilizes the trimeric spike protein and promotes cleavage at the S2′ site (perhaps also the S1/S2 site), resulting in S1 dissociation and S2 refolding, as well as the exposure of the fusion peptide and further facilitates the membrane fusion process.^4−6^

Since its significant role in infection, spike protein has been the primary target for developing vaccines and therapeutics.^8−16^ A large majority of neutralizing antibodies (NAbs) isolated from COVID-19 patients were found to target the RBD domain of spike protein.^9,17^ Most of these NAbs targeting the epitope of RBD intersect with the RBD-hACE2 interface, which directly obstructs the hACE2 binding.^9,18^ Additionally, some other NAbs binding at other different RBD epitopes could prevent viral infection by trapping the RBD in up conformation and destabilizing the spike protein or stabilizing the RBD in the closed state, thus avoiding hACE2 binding.^9,18^ However, spike protein is a mutational hotspot in emerging SARS-CoV-2 variants; especially multiple high-frequency mutations in the RBD domain have been discovered to increase its binding affinity with hACE2.^19,20^ The currently circulating omicron variants, whose spike proteins predominantly occupy open conformation with enhanced ACE2 attachment,^21−23^ escape the majority of existing SARS-CoV-2 neutralizing antibodies, raising concerns about the potential efficiency decrease of vaccines and antibody therapeutics.^24−34^ A noteworthy example is the withdrawal of the Emergency Use Authorization (EUA) of three NAbs-based therapeutics (REGEN-COV, Sotrovimab, and Bamlanivimab/Etesevimab) by the U.S. Food and Drug Administration (FDA) because they are ineffective against the currently prevalent omicron variant.^35^ From a different perspective, small molecule inhibitors might provide COVID-19 therapy alternatives that are less sensitive to mutations.^14^ Although there are no small molecule drugs targeting spike protein in clinical use, developing small molecule modulators of spike protein is a worthwhile attempt.

In one cryo-EM study, Toelzer et al. involuntarily found that essential FFA linoleic acid (LA) bound in three composite pockets of the RBD domains of SARS-CoV-2 spike protein.^36^ LA binding led to a more compact trimer architecture in the region composed of the three RBDs compared to the apo spike protein, resulting in the receptor-binding motifs (RBMs) inaccessible for binding to hACE2. Besides, in vitro functional assays revealed that LA reduces the virus infectivity by suppressing the virion attachment and entry into cells,^37^ and LA treatment of SARS-CoV-2 infected cells inhibits viral replication and causes deformed virions.^38^ Notably, a recent study suggested that the FFA-binding pocket tends to be conserved in most pathogenic β-coronavirus spike proteins, evidenced by binding affinities of LA against spike proteins tested by SPR and cryo-EM structure of LA bound spike protein of SARS-CoV.^38^ Additionally, other groups reported cryo-EM structures of SARS-CoV-2 spike protein bound with small molecules in the FFA-binding pocket, including oleic acid (OA) and all-trans retinoic acid (ATRA).^12,13^ Taken together, the conserved FFA-binding pocket is promising for developing broad-spectrum small molecule modulators for COVID-19 therapy.

In this work, to identify small molecule modulators of SARS-CoV-2 spike protein, we applied a structure-based virtual screening (SBVS) approach against the FFA-binding pocket, followed by binding affinity evaluation and structure determination. Encouragingly, our identified compounds stabilize the closed hACE2-inaccessible conformation of the spike by being deeply buried into the hydrophobic FFA-binding pocket. Furthermore, those compounds did not prefer binding the prototypic spike and an Omicron BA.4 variant, highlighting the potential of the FFA-binding pocket for developing broad-spectrum small molecule modulators to interfere with virus entry.

## Results

### Analysis of the FFA-Binding Pocket

**Figure 2:**
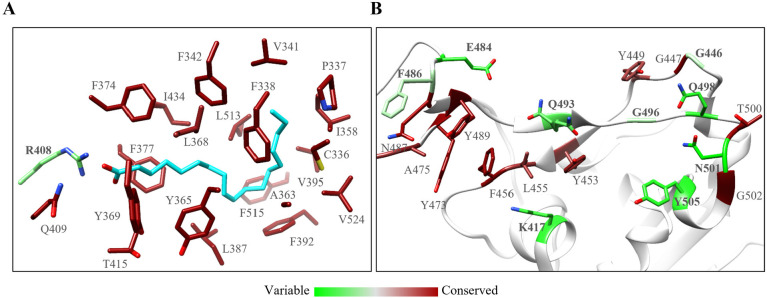
Comparison of the conservation of (A) residues in the FFA-binding pocket (PDB ID: 7E7B) and (B) residues in the RBD-hACE2 interface (PDB ID: 6M0J). Mutation frequencies of residues, derived from the GISAID database (https://www.gisaid.org/) on November 16, 2022, were mapped on the 3D structure of SARS-CoV-2 RBD. Variable residues (i.e., with high mutation frequencies) are displayed as green sticks, while conserved residues (i.e., with low mutation frequencies) are depicted as maroon sticks. Labels of residues associated with variants of concern (VOCs) are bolded.

It is essential to consider the SARS-CoV-2 mutations for developing COVID-19 therapeutics, and we analyzed the mutation frequencies of residues in the RBD of SARS-CoV-2 spike protein obtained from the GISAID database (https://www.gisaid.org/). Consistent with a previous report,^38^ we found that residues in the FFA-binding pocket are evolutionarily conserved in contrast to the residues in the RBD-hACE2 interface (Figure 2). Therefore, the FFA-binding pocket is attractive for developing small molecule modulators that are not susceptible to spike mutations.

**Figure 3:**
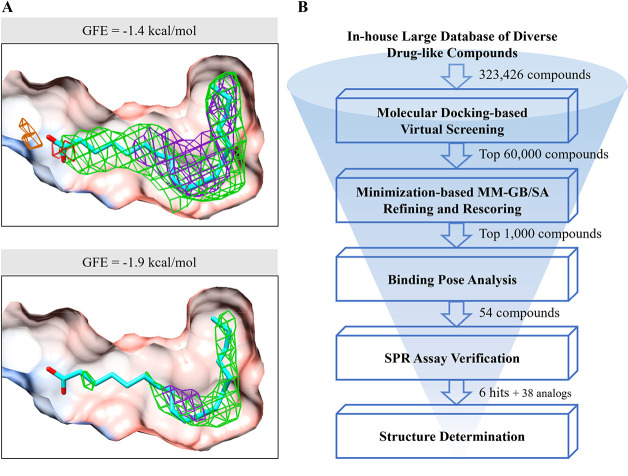
Structure-based discovery of small molecule modulators against the FFA-binding pocket. (A) SILCS FragMaps overlaid on the surface of spike protein with the OA, with a GFE cutoff of −1.4 kcal/mol and −1.9 kcal/mol at the top and bottom, respectively. FragMaps for the aliphatic carbon, aromatic carbon, hydrogen bond acceptor, and acetate oxygen are displayed as green, purple, red, and orange mesh, respectively. The OA is shown in a cyan stick to indicate the position of FraMaps in the FFA-binding pocket. The surface of the two protomers is displayed as transparent salmon and cornflower blue, respectively. Note that unrelated maps outside the pocket were removed for clear visualization. (B) Flowchart of structure-based hierarchical virtual screening.

In addition, we utilized Site Identification by Ligand Competitive Saturation (SILCS) to evaluate the druggability of the FFA-binding pocket, an approach incorporating both the receptor flexibility and solvation effects.^41,42^ FragMaps generated by SILCS simulation reflected the properties of the binding site well (Figure 3A). For example, high-probability regions (grid free energy (GFE) cutoff: −1.4 kcal/mol) in the aliphatic carbon FragMap overlapped with the aliphatic chain of OA, and regions in the hydrogen bond acceptor and acetate oxygen FragMaps almost overlapped with the carboxyl group of OA (Figure 3A). Besides, aromatic carbon FragMap (GFE = −1.4 kcal/mol) appeared in the relatively wide part of the L-shaped pocket. Notably, the aromatic carbon FragMap with higher probability (GFE = −1.9 kcal/mol) overlapped with the position of the double bond of OA, suggesting that ligands with an aromatic ring in the corresponding region may facilitate its binding with spike protein. Taken together, these FragMaps reflected the potential druggability of the FFA-binding pocket and provided a basis for prioritizing small molecule binders to complement this pocket.

### Structure-Based Hierarchical Virtual Screening

**Figure 4:**
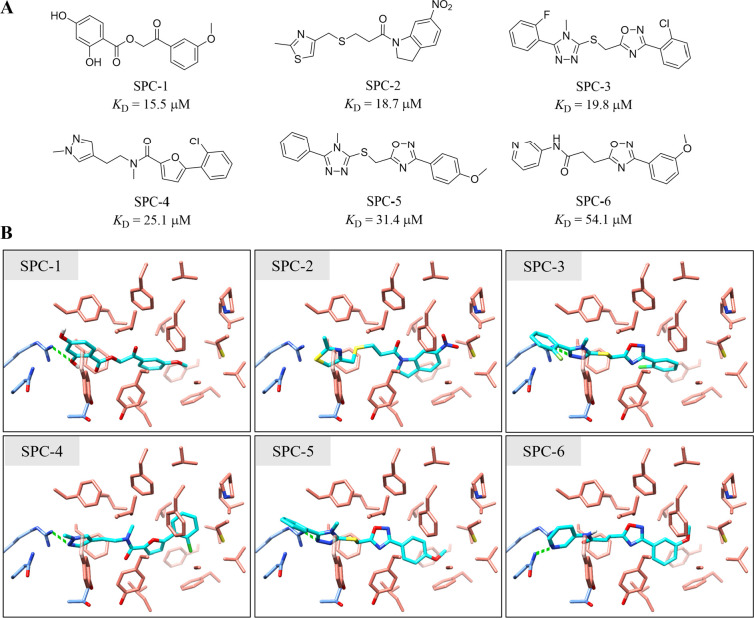
Hits identified by the hierarchical virtual screening approach, with dozens of micromolar *K*_D_ values. (A) 2D structure and corresponding binding affinity of each hit. (B) Docking poses of hits in the FFA-binding pocket in the cryo-EM structure of OA bound SARS-CoV-2 spike protein (PDB ID: 7E7B). Small molecules are depicted as cyan sticks, and residues in the binding pocket are represented as sticks colored in salmon for one RBD and cornflower blue for the adjacent RBD. Backbone atoms of spike protein are hidden for clear visualization. Hydrogen bonding interactions are displayed as green dashed lines.

We adopted a structure-based hierarchical virtual screening strategy (Figure 3B) against the FFA-binding pocket to discover small molecule modulators of SARS-CoV-2 spike protein.^43−45^ We initially performed molecular docking of an in-house compound library containing 323,426 diverse drug-like compounds. Then, 60,000 top-ranked compounds scored by molecular docking were subjected to minimization-based molecular mechanics Generalized-Born surface area (MM-GB/SA) refinement and rescoring. Afterward, 1,000 top-ranked binding poses were analyzed by visual inspection and filtered based on the criterion inspired by SILCS FragMaps: binding pose should contain an aromatic moiety at the position of aromatic carbon FragMap. After the structural analysis, 54 compounds were selected for experimental verification by surface plasmon resonance (SPR) assays (Table S1). Finally, six hits (success rate of 11%) were validated with dozens of micromolar binding affinities (Figure 4). Interestingly, these hits have common structural features, with a single linker connecting the aromatic moieties on both sides. In addition, predicted binding poses indicate that the deeply buried aromatic moiety could form favorable hydrophobic interactions and π–π stacking interactions with nearby residues. And the aromatic moiety located at the interface of two RBDs could form hydrogen bonding or van der Waals interactions with residues in the neighboring RBD.

### Binding Affinity Measurement of Analogs of Hits

After experimental feedback, analogs for the six hits in our in-house compound library and the ZINC15 database were further evaluated by molecular docking and MM-GB/SA rescoring.^46^ Subsequently, 30 compounds were chosen for SPR verification (Table 1 and Tables S2–S3). As a result, all tested compounds exhibited micromolar binding affinities, and the best compound with a *K*_D_ value of 1.7 μM (SPC-21 in Table S2). Taking analogs of SPC-2 as an example, this series of compounds are structurally varied in three sites (Table 1). The R^1^ group in the predicted binding pose (Figure S1) locates close to the protomer interface. Molecules with bulkier groups in this site exhibited better binding affinities, such as SPC-2 (*K*_D_ = 18.7 μM) compared with SPC-7 (*K*_D_ = 60.0 μM), as well as SPC-12 (*K*_D_ = 9.1 μM) and SPC-13 (*K*_D_ = 1.9 μM) compared with SPC-9 (*K*_D_ = 70.9 μM), SPC-10 (*K*_D_ = 36.9 μM), and SPC-11 (*K*_D_ = 52.7 μM). SPC-8, whose X atom is an oxygen atom, also exhibited micromolar binding affinity (*K*_D_ = 8.2 μM).

**Table 1:**
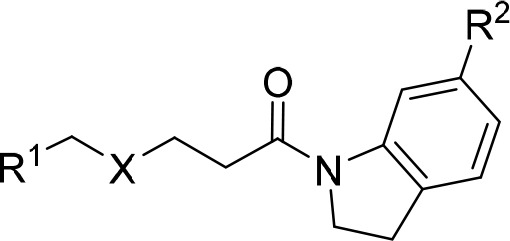

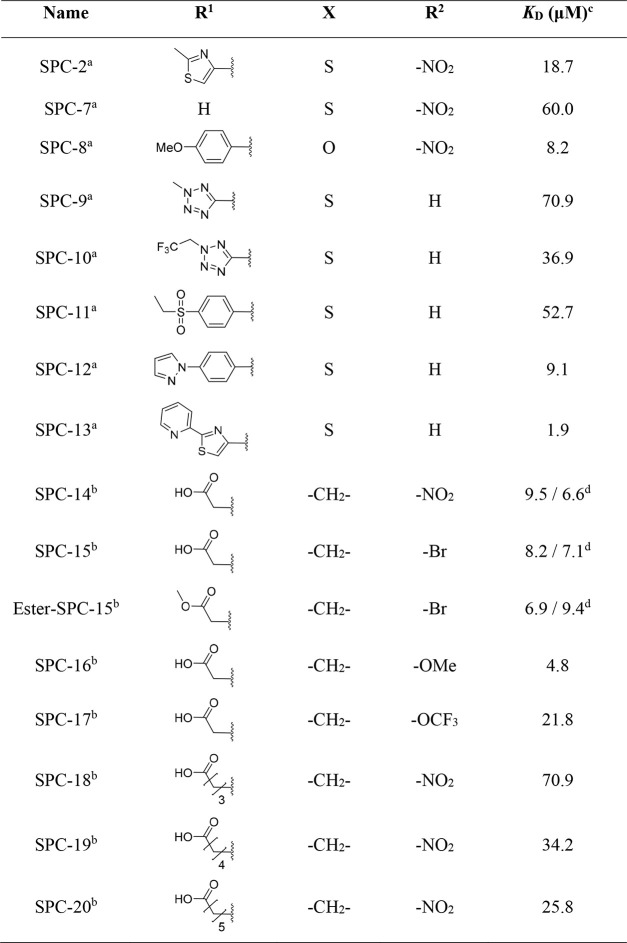
Binding Affinities of Analogs of Compound SPC-2

a Commercially available compounds.

b Synthesized compounds.

c Unless otherwise noted, *K*_D_ values represent binding affinities against the prototypic SARS-CoV-2 spike protein.

d *K*_D_ values were tested against the SARS-CoV-2 Omicron BA.4 spike protein.

**Figure 5:**
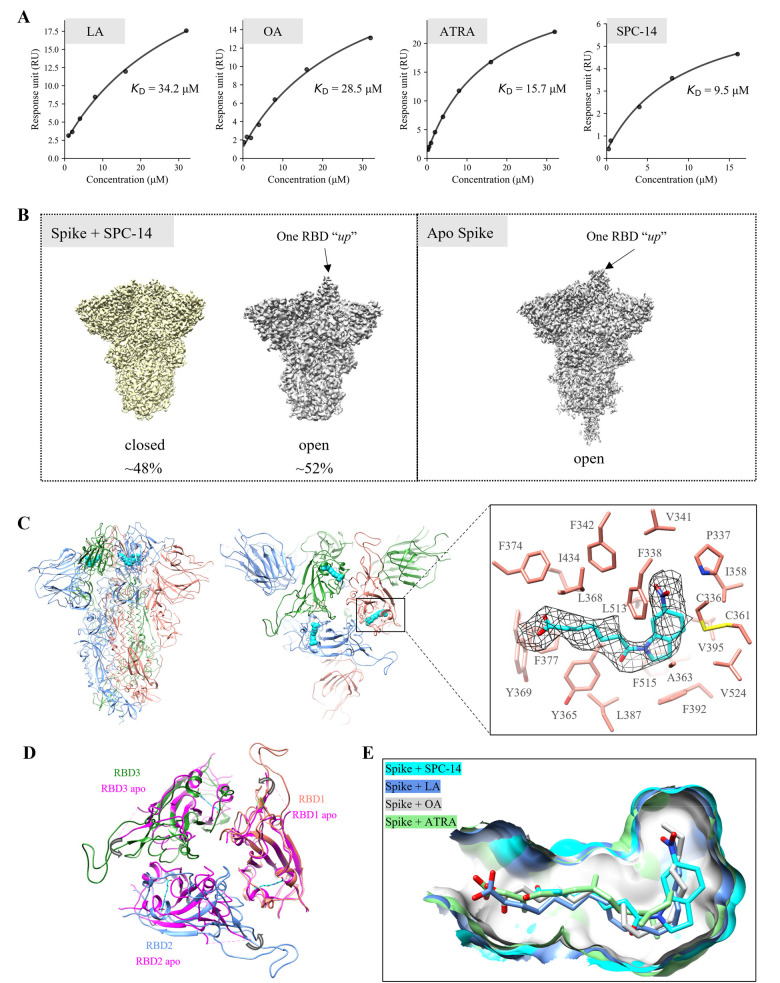
Cryo-EM structure determination. (A) The dose–response curves of LA, OA, ATRA, and SPC-14 binding to trimeric spike protein in the SPR assay. (B) Cryo-EM density maps of spike+SPC-14 (left panel) and apo spike system (right panel). (C) The complex structure of the SPC-14 bound spike is shown in a front view (left panel) and a top view (middle panel). The spike trimer is illustrated as ribbons colored in salmon, cornflower blue, and forest green for three monomers, respectively. Bound SPC-14 is displayed as cyan spheres. The right panel represents the binding mode of SPC-14 (cyan sticks). Additional density in the binding pocket is shown as mesh. Residues are in the stick representation, and their backbone atoms are hidden for clear visualization. (D) Comparison of the RBD trimer of the closed apo spike (magenta, PDB ID: 6VXX) and SPC-14 (cyan sticks) bound spike (cornflower blue, and forest green for three RBDs, respectively). (E) Comparison of the binding pockets of the SPC-14 bound spike (cyan), LA bound spike (cornflower blue, PDB ID: 6ZB5), OA bound spike (light gray, PDB ID: 7E7B), and ATRA bound spike (light green, PDB ID: 7Y42).

Furthermore, we designed and tested seven analogs of SPC-2 after the synthesizability assessment and property evaluation, corresponding to compounds SPC-14 to SPC-20 (Table 1). SPC-14 exhibited a *K*_D_ value of 9.5 μM (Figure 5A). Similar binding affinity was observed when the nitro group was substituted with a bromo group (SPC-15, *K*_D_ = 8.2 μM) or a methoxy group (SPC-16, *K*_D_ = 4.8 μM). In contrast, the trifluoromethoxy substitution exhibited a decreased binding affinity (SPC-17, *K*_D_ = 21.8 μM). Moreover, increasing the fatty chain length in SPC-14 resulted in noticeably decreased binding affinities (SPC-18, *K*_D_ = 70.9 μM; SPC-19, *K*_D_ = 34.2 μM; SPC-20, *K*_D_ = 25.8 μM). The increased flexibility of the aliphatic chain is likely associated with the increased entropy cost and reduces the binding affinity. Notably, several compounds (SPC-14, SPC-15, and SPC-16) obtained better binding affinity in the SPR assay than the reference compounds reported in previous studies (LA, 34.2 μM; OA, 28.5 μM; ATRA, 15.7 μM).^38−40^ To investigate the contribution of the carboxylic acid group for binding, we also tested the binding affinity of the ester analog of SPC-15 (Ester-SPC-15, *K*_D_ = 6.9 μM), which showed comparable binding affinity to SPC-15 (*K*_D_ = 8.2 μM).

Given that the Omicron lineages BA.2, BA.4, and BA.5 have high mutation frequencies at residue R408 in the binding pocket (Figure 2),^47^ we measured the binding affinity of several representative compounds against the spike protein of Omicron BA.4, one of the VOCs that are currently in circulation. SPC-14, SPC-15, and Ester-SPC-15 exhibited similar binding affinities against the prototypic and Omicron BA.4 spike proteins (Table 1). Since only the R408S mutation exists in the FFA-binding pocket for Omicron BA.4 compared with the prototypic spike, it suggested that the R408S mutation has no impact on the binding affinities of our identified compounds. Notably, our identified compounds are hydrophobic due to the hydrophobic nature of the FFA-binding pocket, and thus their solubilities in SPR running buffer are poor. It is evident that many representative ligands formed aggregation at 25 μM (Table S4), and the binding affinities measured for these compounds may have a caveat that saturation was not observed (see Supporting Information).^48−50^ Therefore, improving the target molecules’ solubilities in addition to the binding affinities in the lead optimization stage is desirable.

### Structure Determination

To explore the binding mode of SPC-14 against spike protein, we parallelly prepared cryo-EM grids by incubating the same batch of purified SARS-CoV-2 spike protein with SPC-14 (at a 1:10 molar ratio) or without SPC-14 as a vehicle control on ice for 40 min. For the spike+SPC-14 system, about 48% of the particles were in the closed state (three RBD “down”), and the other 52% of the particles were in the open state with one RBD “up” (Figure 5B). On the contrary, for the apo spike system, almost all the particles were in the open state with one RBD “up”, which is consistent with several previously described cryo-EM structures.^1,51^ This observation indicates that the presence of SPC-14 influences the conformation of RBDs. The cryo-EM structures of the closed state spike+SPC-14 and the apo spike were solved to resolutions of 3.06 and 3.27 Å, respectively. Intriguingly, additional densities located on the three FFA-binding pockets are evident in the closed state spike+SPC-14 map, but not the open state spike+SPC-14 map and the apo spike map. SPC-14 could be well fitted to the densities, with the 6-nitroindoline moiety positioned deeply in the highly hydrophobic environment and the carboxylic group located close to the interface of RBDs (Figure 5C). Hydrophobic interactions dominate the binding interactions, and π–π stacking interaction could be observed between the nitro group of SPC-14 and the aromatic ring of residue F338. Upon SPC-14 binding, each RBD in the trimer moves toward its neighboring RBD and results in a more compact RBD trimer architecture compared to the closed state apo spike (Figure 5D), which is consistent with the previously published locked structure of the LA bound spike.^36^ The overall binding site residues of the SPC-14 bound spike (within 5 Å of SPC-14) adopt similar conformations to those of the spike bound with LA, OA, and ATRA (Figure 5E), with root-mean-square deviations (RMSDs) of 0.47 Å, 0.79 Å, and 1.17 Å, respectively. Due to the bulkier 6-nitroindoline moiety of SPC-14, residues move toward to accommodate it, resulting in a relatively larger volume of the binding pocket nearby the 6-nitroindoline moiety (176 Å^3^, calculated by POVME 3.0^52,53^) compared to the binding pockets of LA bound spike (152 Å^3^), OA bound spike (120 Å^3^), and ATRA bound spike (165 Å^3^). Since the FFA-binding pocket bound with SPC-14 is slightly enlarged than the other compound bound structures, this pocket may be more flexible for binding diverse chemical scaffolds.

Interestingly, the carboxyl group of SPC-14 does not form direct salt-bridge interactions with the R408 residue at the adjacent RBD due to the length restriction of its aliphatic chain, which is different from the binding mode of LA, OA, and ATRA. This binding mode indicates that the deeply buried 6-nitroindoline moiety dominates the ligand binding, and the carboxylic acid group is not essential for binding. This observation is consistent with the results that the ester substitute of the carboxylic acid group (ester-SPC-15) still maintains the binding and that the R408S mutation in Omicron BA.4 showed no effect on the binding of our compounds.

## Discussion and Conclusion

To the best of our knowledge, this is the first study to identify small molecule modulators using a structure-based virtual screening approach against the FFA-binding pocket of SARS-CoV-2 spike protein. The SILCS simulations reflected the druggability of the binding pocket and provided directions for the selection of molecules for experimental verification. Notably, 74% of 1000 top-ranked binding poses enriched by hierarchical virtual screening possessed aromatic rings overlapped with the aromatic carbon FragMap. Furthermore, the successfully identified hits with single-digit micromolar binding affinities suggested the feasibility of the hierarchical virtual screening approach in drug discovery. And our cryo-EM structure of spike bound with SPC-14 serves as a good starting point for structure-based lead optimization.

The conserved FFA-binding pocket was hypothesized to be the Achilles’ Heel of SARS-COV-2.^36,38^ Small molecules targeting this pocket may have the advantage of being broad-spectrum and reducing or even diminishing the viral infectivity and transmission. Unfortunately, to date, only small molecules LA, OA, and ATRA were discovered serendipitously to bind the FFA-binding pocket. And those compounds suffer the low binding affinity, target promiscuity, and metabolic instability, which greatly limit their usage as high-quality chemical probes and in clinical applications. We are aware that our identified compounds require further structural optimization to improve the binding potency for stabilizing the closed spike trimer, and further studies will be needed to establish relevant antiviral efficacies. We also expect that the large-scale virtual screenings of billions of compounds against this pocket may provide more novel and diverse chemical structures to facilitate the development of high-affinity and drug-like binders.

Omicron variant spike proteins predominantly occupied open conformation with enhanced ACE2 attachment and antibody evasion.^21,23,27−32,34^ Therefore, although our identified compounds bind prototypic spike and an Omicron BA.4 variant with similar binding affinities, it is desirable to obtain the cryo-EM structures of Omicron variant spikes bound with SPC-14. Furthermore, the cryo-EM study may provide further evidence of shifting the dynamic equilibrium toward the closed hACE2-inaccessible conformational state by the FFA-binding pocket targeting agents.

## Experimental Methods

### SILCS Simulation

Considering both the accuracy and efficiency, the structure for SILCS simulation contains two neighboring RBD monomers (residues from 333 to 528) of the cryo-EM structure.^39^ The *N*-acetyl-β-glucosaminide (NAG) glycan linked to N343 was not considered here. The structure was solvated with the TIP3P water model,^54^ along with eight solutes (benzene, propane, methanol, formamide, imidazole, acetaldehyde, acetate, and methylammonium) at about 0.25 M concentration. Ten protein-solute-water systems were generated, with each system differing in the orientations of the protein side chains. SILCS simulation was performed with the SILCS 2020.1 program according to the default SILCS simulation protocol.^42^ Generally, the SILCS protocol used the oscillating μex Grand Canonical Monte Carlo (GCMC) program and GROMACS 2019.4 program for energy minimization and molecular dynamics (MD).^55,56^ Briefly, each of the prepared ten systems was initially minimized with the steepest descent (SD) algorithm and then equilibrated for 100 ps using the velocity rescaling thermostat and Berendsen barostat.^57,58^ Each system was subjected to 25 × 200000 step GCMC cycles to redistribute the water and probe solutes. Subsequently, 100 cycles of GCMC/MD simulations were performed. Every GCMC/MD cycle includes (i) 200,000 GCMC steps; (ii) 5,000 step steepest descent minimization and a 100 ps MD equilibration; (iii) 1 ns production MD simulation with Cα protein atoms restrained with a harmonic restraint of 0.12 kcal/mol·Å^2^. The snapshots saved every 10 ps from the 10 × 100 ns of MD trajectories were used to calculate SILCS FragMaps. FragMaps were generated based on the probability distributions of selected probe molecule atoms and the water oxygens calculated on a 1 Å^3^ grid. The probability distributions are then normalized and converted to grid-free energies (GFE) based on a Boltzmann transformation for visualization.

### Structure-Based Hierarchical Virtual Screening

Structure-based virtual screening was performed using the DOCK3.7 program.^59^ All compounds were prepared in the flexibase db2 format with the latest version of the ZINC protocol.^59,60^ The RBD dimer of the spike cryo-EM structure (PDB ID: 7E7B) was used for docking.^39^ Forty-five matching spheres were generated based on the heavy atoms of oleic acid and other nearby spheres calculated by SPHGEN.^61^ The receptor structure was protonated using Reduce.^62^ Grids evaluating the different energy terms were precalculated using CHMEGRID for the van der Waals, QNIFFT for electrostatics, and SOLVMAP for ligand desolvation.^63−66^

MM-GB/SA refinement and rescoring were performed using the Protein Local Optimization Program (PLOP) as described previously.^43,44,67−69^ First, hydrogen atoms of the receptor were added by the all-atom OPLS 2005 force field.^70,71^ Then, the complexes of the receptor and docked ligands were refined and rescored in the Surface Generalized Born implicit solvent with the all-atom OPLS force field.^72,73^ During the refinement, the docked ligand was minimized flexibly in the pocket, whereas the receptor was kept rigid. Finally, the binding energy (*E*_Bind_ = *E*_Complex_ – *E*_Ligand_ – *E*_Receptor_) was calculated by subtracting the optimized free ligand energy in solution (*E*_ligand_) and the free receptor energy in solution (*E*_Receptor_) from the optimized receptor–ligand complex’s energy in solution (*E*_Complex_).

### Protein Expression and Purification

#### Prototypic Spike Protein

To produce S-Trimer fusion protein, cDNA encoding the ectodomain of SARS-CoV-2 S protein (amino acid residues 1 to 1211) with R682S, R683G, R685G, K986P, and V987P mutations was synthesized (Sino Biological) using optimized codons for the human cell. The cDNA was cloned into a pcDNA3.1 vector to allow in-frame fusion of the mutated ectodomain of S protein to Trimer-Tag (residues 458 to 484 from Enterobacteria phage T6 Fibritin protein) followed by 8× histidine tag and Protein C tag. The purified plasmid coding the fusion protein was transfected into Expi293F cells (Thermo Fisher Scientific) using polyethylenimine max (PEI, Polysciences) when the cell density reached 1.5 million per mL. Transfected cells were shaken in SMM 293-TII expression medium (Sino Biological) for 72 h. Twenty-five mM Tris (pH 8.0) and 10 mM CaCl_2_ were added into the centrifugal supernatant of the cell culture medium after the cell and debris were removed. The fusion protein was then purified by anti-ProteinC antibody affinity resin. After washing with buffer A (25 mM Tris, pH 8.0, 150 mM NaCl, 2 mM CaCl_2_), the protein complex was eluted in buffer B (25 mM Tris, pH 8.0, 150 mM NaCl, 5 mM EDTA, 0.1 mg/mL Protein C peptide), concentrated, and purified on a Superose 6 increase 10/300 (GE Healthcare) size-exclusion column equilibrated in buffer C (25 mM HEPES, pH 8.0, and 150 mM NaCl).

#### Omicron Spike Protein

Genes encoding the ectodomain of the Omicron BA.4 spike protein (residues 14–1205) with 6P mutants (F817P, A892P, A899P, A942P, K986P, and V987P) were fused with a C-terminal T4 fibritin trimerization domain, a StrepII tag, and an 8× His tag and cloned into a mammalian cell expression vector pCAGGS. A Kozak sequence and an exogenous signal peptide derived from μ-phosphatase (MGILPSPGMPALLSLVSLLSVLLMGCVAETGT) were added into the N terminus to maximize the protein production as previously reported.^17^ The pCAGGS-S plasmids were transfected using polyethylenimine (PEI) and expressed in HEK293F suspension-cultured cells (Gibco, Cat# 11625-019). Cells were cultured at 37 °C in SMM 293-TII Expression medium (Sino Biological, Cat# M293TII). Cell culture supernatants were collected after a 4-day infection and filtered through 0.22 μm filters. The supernatants containing the spike protein were purified using His-Trap HP columns (GE Healthcare) and the Superose 6 Increase 10/300 GL column (GE Healthcare). Purified proteins were stored in a protein buffer (20 mM Tris-HCl, 150 mM NaCl, pH 8.0).

### Surface Plasmon Resonance (SPR)

The binding affinity between compounds and spike protein was analyzed at 25 °C using the BIAcore T200 (GE Healthcare). PBS-P running buffer (Cytiva, Cat# 28995084) containing 20 mM PBS (pH 7.4), 2.7 mM KCl, 137 mM NaCl, and 0.05% surfactant P20 was used. For chip surface preparation, the SARS-CoV-2 trimeric spike protein was exchanged for PBS buffer via gel filtration and diluted to a final concentration of 50 μg/mL in NaAc buffer (pH 4.1) before immobilization on a CM5 chip through amine coupling. The protein flew through the chip at 10 μL/min in PBS-P buffer until the response unit (RU) reached approximately 12000. The reference channel surface was treated with the same procedure but replacing the ligand with buffer. To ensure full dissolution, the supernatant was taken for gradient dilution after high-speed centrifugation (14000*g*, 15 min) of the highest concentration for each testing compound. SPR measurements of a series of compound concentrations were performed at a 30 μL/min flow rate. The contact time and the dissociation time were 60 and 90 s, respectively. DMSO solvent correction was carried out following the BIAcore T200 guide. The steady-state affinity method incorporated in the BIAEVALUATION 4.1 software was used to display binding curves and calculate equilibrium binding constants (*K*_D_).

### Dynamic Light Scattering

To evaluate aggregation under the SPR assay condition, we performed dynamic light scattering (DLS) using a DynaPro NanoStar instrument (Wyatt Technology) at a laser wavelength of 658 nm and with the detectable particle radius range of 0.2–2500 nm. PBS-P buffer containing 5% DMSO (same as the running buffer used in the SPR assay) prefiltered with 0.22 μm filter was used as the reference sample. Compounds at 25 μM in the PBS-P buffer with 5% DMSO were measured with or without the prior centrifugation (14000*g*, 5 min).

### Cryo-EM Model

#### Cryo-EM Grid Preparation and Data Acquisition

For the spike+SPC-14 system, a total of 10 μL of purified spike protein at a concentration of 3.42 mg/mL was incubated with 2.42 μL of SPC-14 at the concentration of 1 mM at a 1:10 molar ratio on ice for 40 min for the next step of cryo-EM grid preparation. After centrifugation (13500*g*, 4 °C for 5 min), 10 μL of supernatant was diluted into 1.53 mg/mL and applied for the next step of cryo-EM grid preparation. For the apo spike system, a total of 5 μL of purified spike protein at a concentration of 3.42 mg/mL was diluted into 1.71 mg/mL and applied for cryo-EM grid preparation. An aliquot of 4 μL of protein sample of spike and SPC-14 complex was applied onto a glow-discharged 400 mesh grid (Quantifoil Au R1.2/1.3) supported with a thin layer of graphene oxide (GO), blotted with filter paper for 2.0 s and plunge-frozen in liquid ethane using a Thermo Fisher Vitrobot Mark IV. Cryo-EM micrographs were collected on a 300 kV Thermo Fisher Krios G4 electron microscope equipped with a Falcon 4 direct detection camera. The micrographs were collected at a calibrated magnification of 96,000×, yielding a pixel size of 0.86 Å at a counting mode. In total, 3,476 micrographs were collected at an accumulated electron dose of 50 e^–^Å^–2^ s^–1^ on each micrograph that was fractionated into a stack of 32 frames with a defocus range of −1.0 μm to −2.0 μm.

#### EM Data Processing

Beam-induced motion correction was performed on the stack of frames using MotionCorr2.^74^ The contrast transfer function (CTF) parameters were determined by CTFFIND4.^75^ For the spike+SPC-14 complex, a total of 4,841 good micrographs were selected for further data processing using cryoSPARC.^76^ Particles were autopicked by the autopicking program in cryoSPARC, followed by three rounds of reference-free 2D classifications. Next, 140,188 particles were selected from good 2D classes and were subjected to a round of 3D classification using a reconstruction of the spike protein as a starting model. Two converged 3D classes were selected for a final round of 3D refinement. In one class, all three RBDs of spike protein show down conformation. 59,187 particles were included in this class, yielding a final reconstruction at a global resolution of 3.06 Å based on the gold-standard Fourier shell correlation criterion at FSC = 0.143. Some additional density other than the main chain can be observed in the FFA-binding pocket. During model building, the additional density had been identified as the corresponding density of SPC-14. The local resolution was then calculated on the final density map. In another class, one RBD of spike protein shows an up conformation, and the other two RBDs show down conformations. 65,052 particles were included in this class, yielding a final reconstruction at a global resolution of 3.27 Å based on the gold-standard Fourier shell correlation criterion at FSC = 0.143. In this class, credible additional density around the FFA-binding pocket cannot be observed. The local resolution was then calculated on the final density map.

For the apo spike protein system, a total of 3,048 good micrographs were selected for further data processing using cryoSPARC.^76^ Particles were autopicked by the Autopicking program in cryoSPARC, followed by 2 rounds of reference-free 2D classifications. Next, 68,452 particles were selected from good 2D classes and were subjected to two rounds of 3D classification using a reconstruction of the spike protein as a starting model. One converged 3D class was selected for a final round of 3D refinement. In this class, one RBD shows up conformation, the other two RBDs show down conformations. 34,118 particles were included in this class, yielding a final reconstruction at a global resolution of 3.27 Å based on the gold-standard Fourier shell correlation criterion at FSC = 0.143. No additional density other than the main chain can be observed in the apo spike protein system. The local resolution was then calculated on the final density map.

#### Model Building and Refinement

The model of spike+SPC-14 complex and apo spike was built by fitting the model of the structure of apo spike (predicted by AlphaFold2) into the density map using UCSF Chimera,^7,77^ followed by a manual model building in COOT and a real space refinement in PHENIX.^78,79^ The model statistics are listed in Table S5.

### Chemistry

Our in-house compound library for virtual screening was constructed from the ChemDiv database (http://eu.chemdiv.com), Enamine (https://enamine.net), Vitas-M (https://vitasmlab.biz), and ChemBridge (https://www.chembridge.com) based on maximizing structural diversity.^44,45^ The vendors characterized each compound with liquid chromatography–mass spectrometry (LC-MS) and nuclear magnetic resonance (NMR) experiments (see Supporting Information), and the purity of each compound was larger than 90%.

#### Synthesis of SPC-14

Heptanedioic acid anhydride (439.2 mg, 2.75 mmol) and 6-nitroindoline (300.0 mg, 1.83 mmol) were dissolved in dichloromethane (4 mL) in a one-necked flask (10 mL) filled with N_2_. The reaction mixture was stirred at room temperature (rt) for 2 h. Then the mixture was concentrated under reduced pressure and further purified by column chromatography giving the compound SPC-14 (yellow solid, 381.2 mg, 68.1%). ^1^H NMR (600 MHz, DMSO) δ 8.82 (d, *J* = 1.4 Hz, 1H), 7.89 (dd, *J* = 8.2, 2.3 Hz, 1H), 7.47 (d, *J* = 8.2 Hz, 1H), 4.20 (t, *J* = 8.6 Hz, 2H), 3.26 (t, *J* = 8.5 Hz, 2H), 2.48 (t, *J* = 7.4 Hz, 2H), 2.20 (dt, *J* = 16.0, 7.4 Hz, 2H), 1.68–1.45 (m, 4H), 1.44–1.28 (m, 2H).^13^C NMR (101 MHz, DMSO-*d*_6_) δ (ppm) 174.93, 170.80, 147.39, 144.44, 140.89, 125.80, 119.09, 110.25, 48.52, 35.06, 34.07, 28.63, 28.00, 24.86, 23.95.

#### Synthesis of Ester-SPC-15 and SPC-15

To a solution of 7-methoxy-7-oxoheptanoic acid (200 mg, 1.15 mmol) and 6-bromoindoline (189.8 mg, 0.96 mmol) in dimethylformamide (DMF) (5 mL) were added *N*,*N*-diisopropylethylamine (DIEA) (372 mg, 2.88 mmol) and hexafluorophosphate azabenzotriazole tetramethyl uronium (HATU) (548 mg, 1.44 mmol). The reaction was stirred at rt for 16 h. The mixture was quenched with water, extracted by ethyl acetate (EA) (50 mL*3), dried by Na_2_SO_4_, concentrated under vacuum, and purified by column chromatography to obtain a white solid as the target product Ester-SPC-15 (200 mg, 59.8%). MS [M + H]^+^ 354, ^1^H NMR (400 MHz, DMSO) δ 8.24 (s, 1H), 7.19–7.13 (m, 2H), 4.10 (t, *J* = 8.5 Hz, 2H), 3.58 (s, 3H), 3.09 (t, *J* = 8.5 Hz, 2H), 2.44 (t, *J* = 7.2 Hz, 2H), 2.31 (t, *J* = 7.4 Hz, 2H), 1.56 (td, *J* = 7.5, 5.2 Hz, 4H), 1.35–1.28 (m, 2H). Subsequently, Ester-SPC-15 (140 mg, 0.40 mmol) was dissolved in EtOH/H_2_O (5 mL/1 mL), and LiOH (49.8 mg, 1.2 mmol) was added. The reaction was stirred at rt for 16 h. The mixture was quenched with water, adjusted pH to 3 with HCl, extracted by EA (50 mL*3), dried by Na_2_SO_4_, concentrated under vacuum, and purified by prep-HPLC to obtain a white solid as the target product SPC-15 (13.6 mg, 10.1%). MS [M + H]^+^ 340, ^1^H NMR (400 MHz, DMSO) δ 8.43 (s, 1H), 7.12 (dd, *J* = 7.9, 1.7 Hz, 1H), 7.01 (d, *J* = 7.9 Hz, 1H), 4.06 (t, *J* = 8.5 Hz, 2H), 3.14 (t, *J* = 8.4 Hz, 2H), 2.41 (dt, *J* = 14.7, 7.3 Hz, 4H), 1.73 (ddt, *J* = 22.7, 15.2, 7.4 Hz, 4H), 1.47 (dd, *J* = 15.3, 8.0 Hz, 2H).

#### Synthesis of SPC-16

To a solution of 7-methoxy-7-oxoheptanoic acid (200 mg, 1.15 mmol) and 6-methoxyindoline (142.74 mg, 0.96 mmol) in DMF (5 mL) were added DIEA (370.95 mg, 2.87 mmol) and HATU (545.68 mg, 1.44 mmol). The reaction was stirred at rt for 16 h. The mixture was quenched with water, extracted by EA (50 mL*3), dried by Na_2_SO_4_, concentrated under vacuum, and purified by column chromatography to obtain a white solid as the intermediate product (methyl 7-(6-methoxyindolin-1-yl)-7-oxoheptanoate, 250 mg, 85.57%). Then the intermediate product (140 mg, 0.46 mmol) was dissolved in EtOH/H_2_O (5 mL/1 mL). After the addition of LiOH (57.77 mg, 1.38 mmol), the reaction was stirred at rt for 16 h. The mixture was quenched with water, adjusted pH to 3 with HCl, extracted by EA (50 mL*3), dried by Na_2_SO_4_, concentrated under vacuum, and purified by prep-HPLC to obtain a white solid as the target product SPC-16 (36.2 mg, 27.1%). MS [M + H]^+^ 292, ^1^H NMR (400 MHz, DMSO) δ 11.95 (s, 1H), 7.70 (d, *J* = 2.3 Hz, 1H), 7.05 (d, *J* = 8.2 Hz, 1H), 6.50 (dd, *J* = 8.2, 2.5 Hz, 1H), 4.04 (t, *J* = 8.5 Hz, 2H), 3.66 (s, 3H), 2.99 (t, *J* = 8.4 Hz, 2H), 2.38 (t, *J* = 7.3 Hz, 2H), 2.17 (t, *J* = 7.3 Hz, 2H), 1.51–1.46 (m, 4H), 1.30–1.25 (m, 2H).

#### Synthesis of SPC-17

To a solution of 7-methoxy-7-oxoheptanoic acid (250 mg, 1.44 mmol) and 6-(trifluoromethoxy) indoline (242.97 mg, 2 mmol) in DMF (5 mL) were added DIEA (463.69 mg, 3.59 mmol) and HATU (682.1 mg, 1.79 mmol). The reaction was stirred at rt for 16 h. The mixture was quenched with water, extracted by EA (50 mL*3), dried by Na_2_SO_4_, concentrated under vacuum, and purified by column chromatography to obtain a white solid as the intermediate product (methyl 7-oxo-7-(6-(trifluoromethoxy) indolin-1-yl) heptanoate, 245 mg, 57%). Then the intermediate product (150 mg, 0.42 mmol) was dissolved in EtOH/H_2_O (5 mL/1 mL). After the addition of LiOH (52.59 mg, 1.25 mmol), the reaction was stirred at rt for 16 h. The mixture was quenched with water, adjusted pH to 3 with HCl, extracted by EA (50 mL*3), dried by Na_2_SO_4_, concentrated under vacuum, and purified by prep-HPLC to obtain a white solid as the target product SPC-17 (36.2 mg, 25.11%). MS [M + H]^+^ 346, ^1^H NMR (400 MHz, DMSO) δ 11.95 (s, 1H,), 7.98 (s, 1H), 7.27 (d, *J* = 8.1 Hz, 1H), 6.91 (dd, *J* = 8.1, 1.4 Hz, 1H), 4.11 (t, *J* = 8.6 Hz, 2H), 3.10 (t, *J* = 8.5 Hz, 2H), 2.41 (t, *J* = 7.2 Hz, 2H), 2.17 (t, *J* = 7.3 Hz, 2H), 1.51 (ddd, *J* = 22.8, 12.5, 7.5 Hz, 4H), 1.29–1.25 (m, 2H).

#### Synthesis of SPC-18

To a solution of 9-methoxy-9-oxononanoic acid (200 mg, 0.99 mmol) and 6-nitroindoline (135 mg, 0.82 mmol) in DMF (5 mL) were added DIEA (319 mg, 2.47 mmol) and HATU (469 mg, 1.23 mmol). The reaction was stirred at rt for 16 h. The mixture was quenched with water, extracted by EA (50 mL*3), dried by Na_2_SO_4_, concentrated under vacuum, and purified by column chromatography to obtain a yellow solid as the intermediate product (methyl 9-(6-nitroindolin-1-yl)-9-oxononanoate, 230 mg, 79.7%). Then the intermediate product (150 mg, 0.43 mmol) was dissolved in EtOH/H_2_O (5 mL/1 mL), and LiOH (54.2 mg, 1.29 mmol) was added. The reaction was stirred at rt for 2 h. The mixture was quenched with water, pH adjusted to 3 with HCl, extracted by EA (50 mL*3), dried by Na_2_SO_4_, concentrated under vacuum, and purified by prep-HPLC to obtain a yellow solid as the target product SPC-18 (21.2 mg, 14.7%). MS [M + H]^+^ 335, ^1^H NMR (400 MHz, DMSO) δ 11.94 (s, 1H), 8.83 (*J* = 1.9 Hz, 1H), 7.89 (dd, *J* = 8.2, 2.3 Hz, 1H), 7.48 (d, *J* = 8.2 Hz, 1H), 4.20 (t, *J* = 8.6 Hz, 2H), 3.26 (t, *J* = 8.5 Hz, 2H), 2.47 (d, *J* = 7.3 Hz, 2H), 2.20 (t, *J* = 7.3 Hz, 2H), 1.58 (dd, *J* = 14.0, 7.1 Hz, 2H), 1.51–1.48 (m, 2H), 1.31 (s, 6H).

#### Synthesis of SPC-19

To a solution of 10-methoxy-10-oxodecanoic acid (250 mg, 1.16 mmol) and 6-nitroindoline (158 mg, 0.96 mmol) in DMF (5 mL) were added DIEA (372 mg, 2.88 mmol) and HATU (548 mg, 1.44 mmol). The reaction was stirred at rt for 16 h. The mixture was quenched with water, extracted by EA (50 mL*3), dried by Na_2_SO_4_, concentrated under vacuum, and purified by column chromatography to obtain a yellow solid as the intermediate product (methyl 10-(6-nitroindolin-1-yl)-10-oxodecanoate, 340 mg, 97.4%). Then the intermediate product (186 mg, 0.51 mmol) was dissolved in EtOH/H_2_O (5 mL/1 mL), and LiOH (65 mg, 1.54 mmol) was added. The reaction was stirred at rt for 2 h. The mixture was quenched with water, pH adjusted to 3 with HCl, extracted by EA (50 mL*3), dried by Na_2_SO_4_, concentrated under vacuum, and purified by prep-HPLC to obtain a yellow solid as the target product SPC-19 (13 mg, 7.3%). MS [M + H]^+^ 349, ^1^H NMR (400 MHz, DMSO) δ 11.93 (s, 1H), 8.78 (s, 1H), 7.85 (dd, *J* = 8.2, 2.2 Hz, 1H), 7.43 (d, *J* = 8.2 Hz, 1H), 4.16 (t, *J* = 8.6 Hz, 2H), 3.22 (t, *J* = 8.5 Hz, 2H), 2.45–2.43 (m, 2H), 2.15 (t, *J* = 7.4 Hz, 2H), 1.55–1.53 (m, 2H), 1.44 (dd, *J* = 14.0, 7.1 Hz, 2H), 1.28–1.25 (m, 4H), 1.24 (d, *J* = 3.3 Hz, 4H).

#### Synthesis of SPC-20

To a solution of 11-methoxy-11-oxoundecanoic acid (200 mg, 0.87 mmol) and 6-nitroindoline (119 mg, 0.72 mmol) in DMF (5 mL) were added DIEA (279 mg, 2.16 mmol) and HATU (411 mg, 1.08 mmol). The reaction was stirred at rt for 16 h. The mixture was quenched with water, extracted by EA (50 mL*3), dried by Na_2_SO_4_, concentrated under vacuum, and purified by column chromatography to obtain a yellow solid as the intermediate product (methyl 11-(6-nitroindolin-1-yl)-11-oxoundecanoate, 250 mg, 91.5%). Then the intermediate product (176 mg, 0.47 mmol) was dissolved in EtOH/H_2_O (5 mL/1 mL), and LiOH (58.9 mg, 1.4 mmol) was added. The reaction was stirred at rt for 2 h. The mixture was quenched with water, adjusted pH to 3 with HCl, extracted by EA (50 mL*3), dried by Na_2_SO_4_, concentrated under vacuum, and purified by prep-HPLC to obtain a yellow solid as the target product SPC-20 (12 mg, 7.1%). MS [M + H]^+^ 363, ^1^H NMR (400 MHz, DMSO) δ 11.97 (s, 1H), 8.82 (d, *J* = 1.6 Hz, 1H), 7.89 (dd, *J* = 8.2, 2.2 Hz, 1H), 7.47 (d, *J* = 8.2 Hz, 1H), 4.20 (t, *J* = 8.6 Hz, 2H), 3.26 (t, *J* = 8.5 Hz, 2H), 2.47 (d, *J* = 7.2 Hz, 2H), 2.19 (t, *J* = 7.3 Hz, 2H), 1.58 (dd, *J* = 13.9, 7.0 Hz, 2H), 1.49–1.47 (m, 2H), 1.31 (s, 4H), 1.27 (s, 6H).

## Data Availability

The docking poses of 1,000 top-scored compounds and all compounds tested in SPR assay are available at https://www.huanglab.org.cn/for_submission_wq/; Cryo-EM maps of the apo SARS-CoV-2 spike protein and the SPC-14 bound SARS-COV-2 spike protein have been deposited in the Electron Microscopy Data Bank under the accession codes EMD-34464 and EMD-34465, and the Protein Data Bank under the PDB codes 8H3D and 8H3E, respectively.

## Supporting Information Available

Validation report of cryo-EM structure of SARS-CoV-2 apo spike (PDF)
Validation report of cryo-EM structure of SPC-14 bound SARS-CoV-2 spike (PDF)
The structure and activity of all tested compounds; concentration response curves and steady-state binding curves in SPR assay; DLS intensity profile of representative compounds; the cryo-EM data collection, refinement, and validation statistics; ^1^H NMR, ^13^C NMR, and LC-MS spectra of representative compounds (PDF)
