# Supplementary material for: In Silico Discovery of Small Molecule Modulators Targeting the Achilles’ Heel of SARS-CoV-2 Spike Protein
Source: ACS Cent Sci. 2023 Feb 8;9(2):252–65. doi: 10.1021/acscentsci.2c01190 (PMC9924089; doi:10.1021/acscentsci.2c01190)
Supplement: Supplementary file 2 — oc2c01190_si_002.pdf [file oc2c01190_si_002.pdf]

Preliminary Full wwPDB EM Validation Report (i)

Oct 7, 2022 – 05:58 PM JST

Deposition ID : D_1300032715

This wwPDB validation report is NOT for manuscript review

This is a Preliminary Full wwPDB EM Validation Report.

This report is produced by the wwPDB Deposition System during initial deposition but before annotation of the structure.

We welcome your comments at *validation@mail.wwpdb.org*
A user guide is available at
https://www.wwpdb.org/validation/2017/EMValidationReportHelp
with specific help available everywhere you see the (i) symbol.

The types of validation reports are described at
http://www.wwpdb.org/validation/2017/FAQs#types.

Not For Manuscript Review

The following versions of software and data (see references (i)) were used in the production of this report:

EMDB validation analysis	:	0.0.1.dev43
Mogul	:	1.8.5 (274361), CSD as541be (2020)
MolProbity	:	4.02b-467
Percentile statistics	:	20191225.v01 (using entries in the PDB archive December 25th 2019)
MapQ	:	1.9.9
Ideal geometry (proteins)	:	Engh & Huber (2001)
Ideal geometry (DNA, RNA)	:	Parkinson et al. (1996)
Validation Pipeline (wwPDB-VP)	:	2.31.2

1 Overall quality at a glance (i)

The following experimental techniques were used to determine the structure:
ELECTRON MICROSCOPY

The reported resolution of this entry is unknown.

Percentile scores (ranging between 0-100) for global validation metrics of the entry are shown in the following graphic. The table shows the number of entries on which the scores are based.

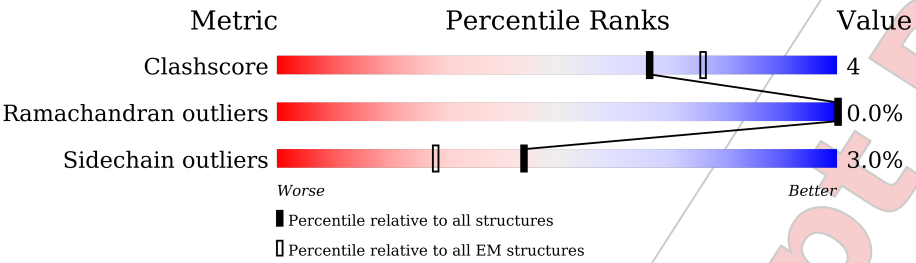

Metric	Whole archive (#Entries)	EM structures (#Entries)
Clashscore	158937	4297
Ramachandran outliers	154571	4023
Sidechain outliers	154315	3826

The table below summarises the geometric issues observed across the polymeric chains and their fit to the map. The red, orange, yellow and green segments of the bar indicate the fraction of residues that contain outliers for >=3, 2, 1 and 0 types of geometric quality criteria respectively. A grey segment represents the fraction of residues that are not modelled. The numeric value for each fraction is indicated below the corresponding segment, with a dot representing fractions <=5% The upper red bar (where present) indicates the fraction of residues that have poor fit to the EM map (all-atom inclusion < 40%). The numeric value is given above the bar.

Mol	Chain	Length	Quality of chain
1	A	1276	11% / 72% 8% 20%
1	B	1276	• / 72% 9% 18%
1	C	1276	72% 9% 19%

Not For Manuscript Review

2 Entry composition (i)

There are 2 unique types of molecules in this entry. The entry contains 24448 atoms, of which 0 are hydrogens and 0 are deuteriums.

In the tables below, the AltConf column contains the number of residues with at least one atom in alternate conformation and the Trace column contains the number of residues modelled with at most 2 atoms.

- Molecule 1 is a protein.

Mol	Chain	Residues	Atoms					AltConf	Trace
			Total	C	N	O	S		
1	A	1023	8005	5119	1326	1524	36	0	0
1	B	1041	8142	5205	1351	1548	38	0	0
1	C	1039	8119	5187	1348	1546	38	0	0

- Molecule 2 is 2-acetamido-2-deoxy-beta-D-glucopyranose (three-letter code: NAG) (formula: $C_8H_{15}NO_6$).

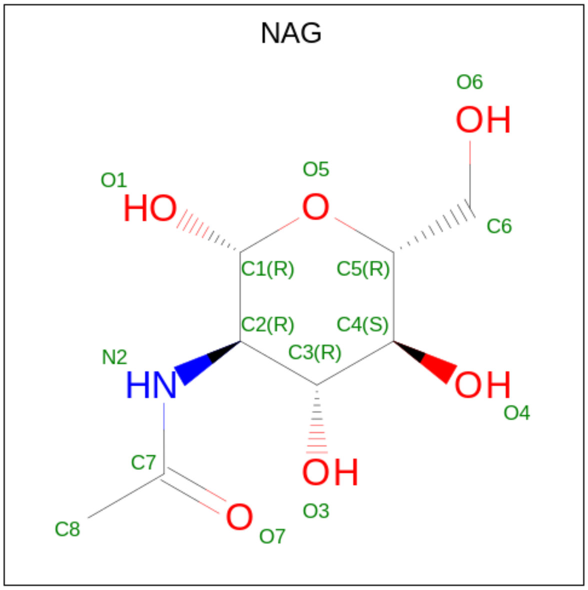

Mol	Chain	Residues	Atoms				AltConf
			Total	C	N	O	
2	A	1	42	24	3	15	0
2	A	1	42	24	3	15	0
2	A	1	42	24	3	15	0

Continued on next page...

Not For Manuscript Review

Continued from previous page...

Mol	Chain	Residues	Atoms	AltConf
2	B	1	Total C N O<br>70 40 5 25	0
2	B	1	Total C N O<br>70 40 5 25	0
2	B	1	Total C N O<br>70 40 5 25	0
2	B	1	Total C N O<br>70 40 5 25	0
2	B	1	Total C N O<br>70 40 5 25	0
2	C	1	Total C N O<br>70 40 5 25	0
2	C	1	Total C N O<br>70 40 5 25	0
2	C	1	Total C N O<br>70 40 5 25	0
2	C	1	Total C N O<br>70 40 5 25	0
2	C	1	Total C N O<br>70 40 5 25	0

Not For Manuscript Review

3 Residue-property plots (i)

These plots are drawn for all protein, RNA, DNA and oligosaccharide chains in the entry. The first graphic for a chain summarises the proportions of the various outlier classes displayed in the second graphic. The second graphic shows the sequence view annotated by issues in geometry and atom inclusion in map density. Residues are color-coded according to the number of geometric quality criteria for which they contain at least one outlier: green = 0, yellow = 1, orange = 2 and red = 3 or more. A red diamond above a residue indicates a poor fit to the EM map for this residue (all-atom inclusion < 40%). Stretches of 2 or more consecutive residues without any outlier are shown as a green connector. Residues present in the sample, but not in the model, are shown in grey.

- Molecule 1:

Chain A:

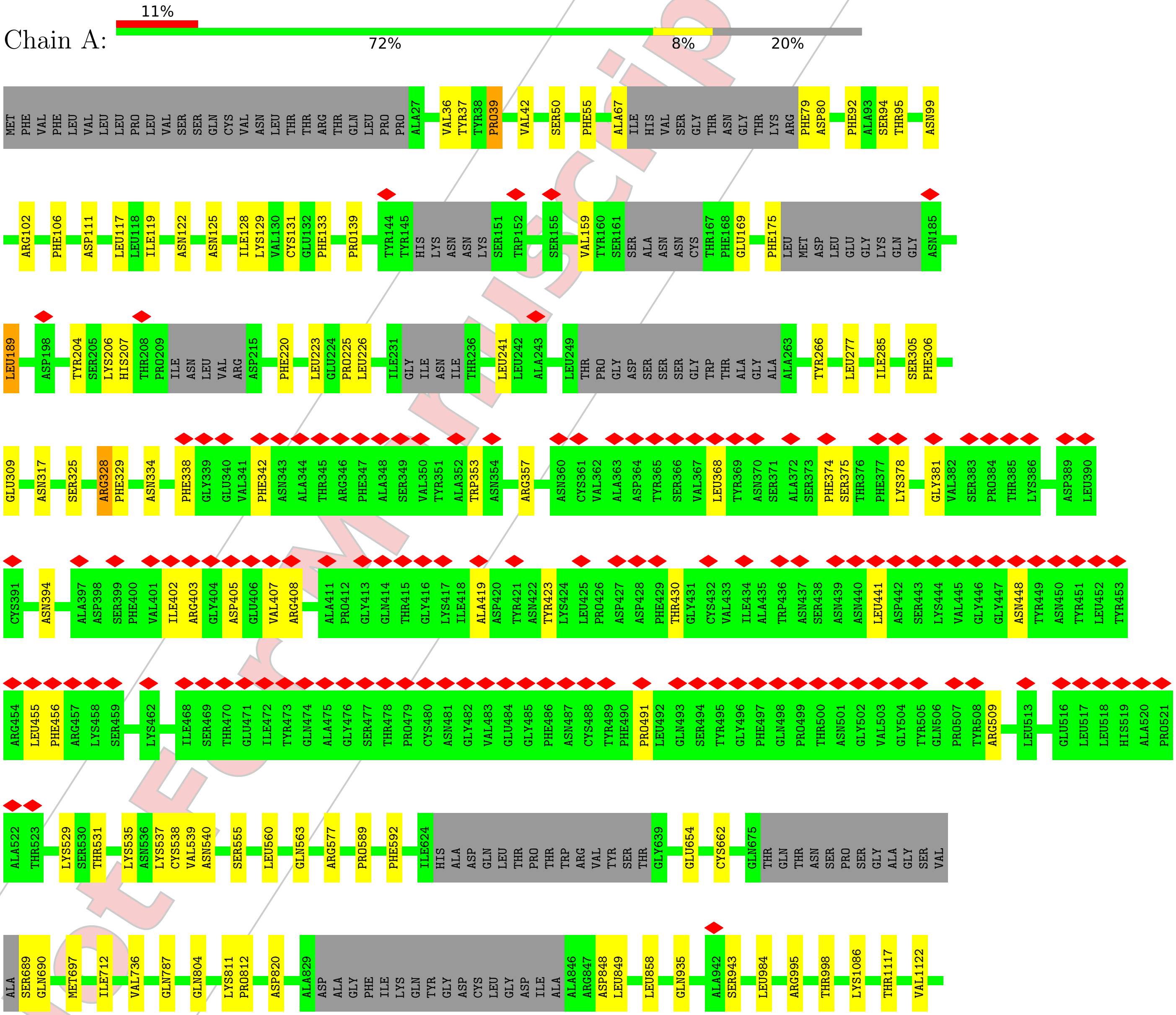

Not For Manuscript Review

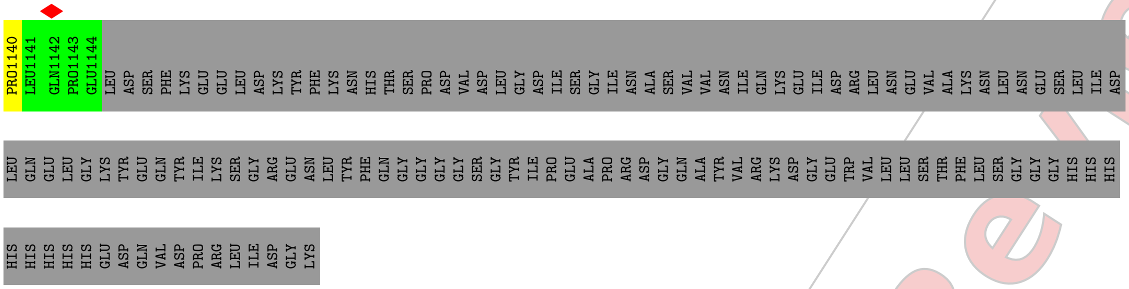

• Molecule 1:

Chain B:

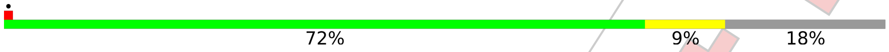

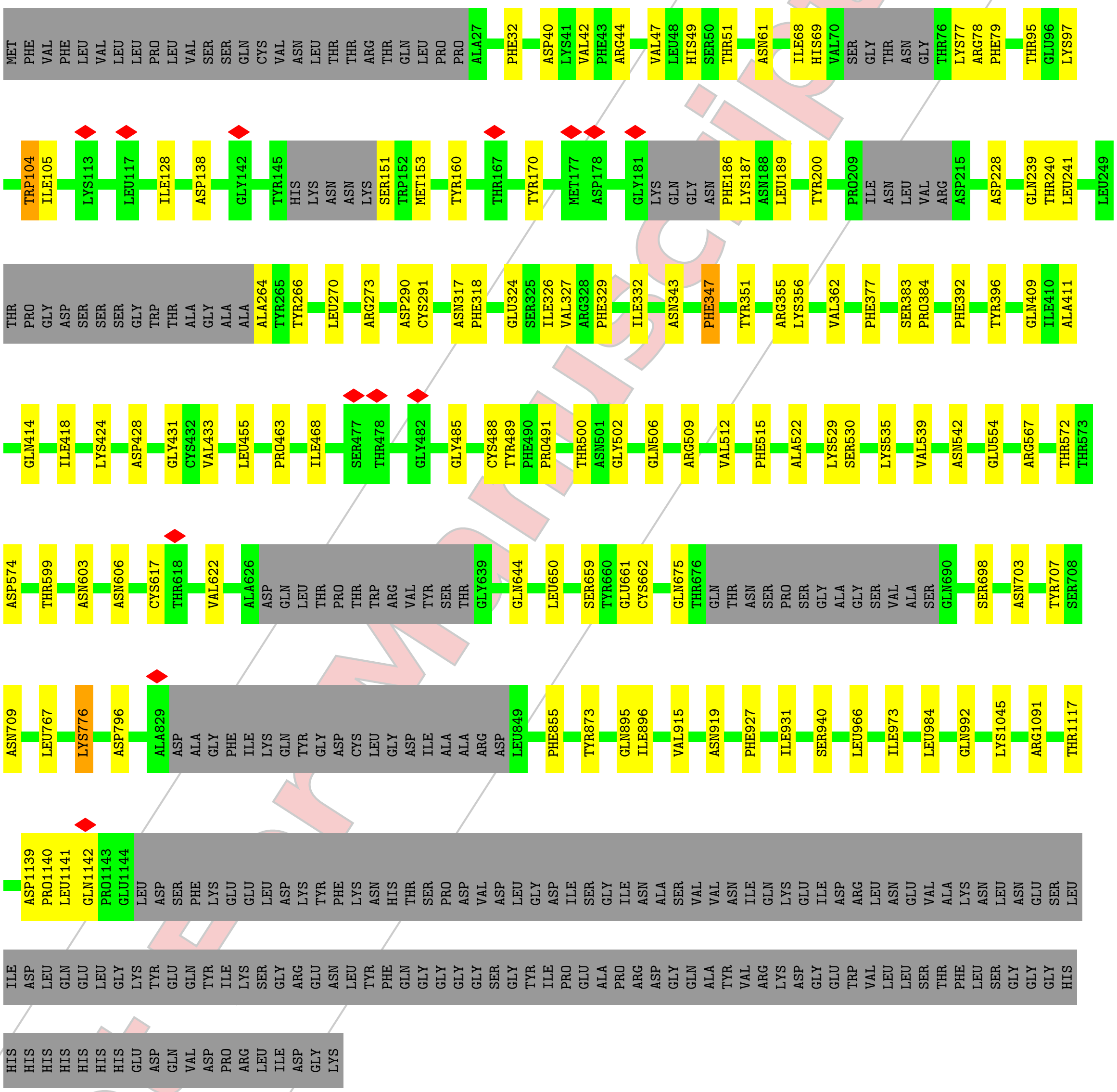

• Molecule 1:

Chain C:

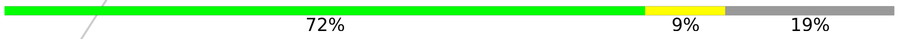

Not For Manuscript Review

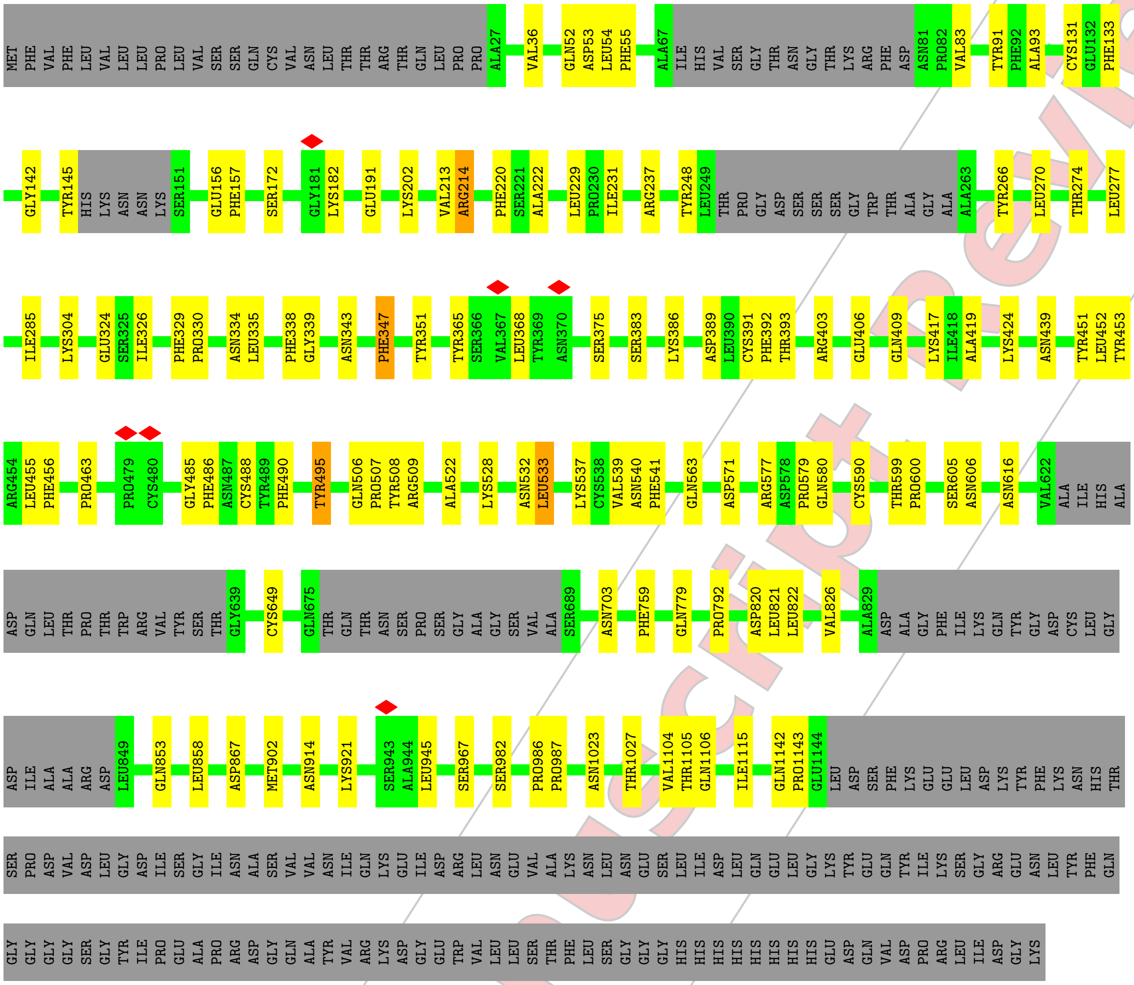

Not For Manuscript Review

4 Experimental information (i)

Property	Value	Source
EM reconstruction method	SINGLE PARTICLE	Depositor
Imposed symmetry	POINT, Not provided	
Number of particles used	Not provided	
Resolution determination method	Not provided	
CTF correction method	Not provided	
Microscope	Not provided	
Voltage (kV)	Not provided	
Electron dose ($e^-/\text{Å}^2$)	Not provided	
Minimum defocus (nm)	Not provided	
Maximum defocus (nm)	Not provided	
Magnification	Not provided	
Image detector	Not provided	
Maximum map value	4.177	Depositor
Minimum map value	-2.003	Depositor
Average map value	-0.001	Depositor
Map value standard deviation	0.099	Depositor
Recommended contour level	0.4	Depositor
Map size (Å)	412.80002, 412.80002, 412.80002	wwPDB
Map dimensions	480, 480, 480	wwPDB
Map angles (°)	90.0, 90.0, 90.0	wwPDB
Pixel spacing (Å)	0.86, 0.86, 0.86	Depositor

Not For Manuscript Review

5 Model quality (i)

5.1 Standard geometry (i)

Bond lengths and bond angles in the following residue types are not validated in this section: NAG

The Z score for a bond length (or angle) is the number of standard deviations the observed value is removed from the expected value. A bond length (or angle) with $|Z| > 5$ is considered an outlier worth inspection. RMSZ is the root-mean-square of all Z scores of the bond lengths (or angles).

Mol	Chain	Bond lengths		Bond angles	
		RMSZ	#|Z| >5	RMSZ	#|Z| >5
1	A	0.27	0/8188	0.49	0/11136
1	B	0.27	0/8329	0.49	0/11330
1	C	0.26	0/8305	0.49	0/11299
All	All	0.27	0/24822	0.49	0/33765

There are no bond length outliers.

There are no bond angle outliers.

There are no chirality outliers.

There are no planarity outliers.

5.2 Too-close contacts (i)

In the following table, the Non-H and H(model) columns list the number of non-hydrogen atoms and hydrogen atoms in the chain respectively. The H(added) column lists the number of hydrogen atoms added and optimized by MolProbity. The Clashes column lists the number of clashes within the asymmetric unit, whereas Symm-Clashes lists symmetry-related clashes.

Mol	Chain	Non-H	H(model)	H(added)	Clashes	Symm-Clashes
1	A	8005	0	7792	51	0
1	B	8142	0	7933	66	0
1	C	8119	0	7917	63	0
2	A	42	0	39	0	0
2	B	70	0	65	3	0
2	C	70	0	65	4	0
All	All	24448	0	23811	175	0

The all-atom clashscore is defined as the number of clashes found per 1000 atoms (including hydrogen atoms). The all-atom clashscore for this structure is 4.

Not For Manuscript Review

All (175) close contacts within the same asymmetric unit are listed below, sorted by their clash magnitude.

Atom-1	Atom-2	Interatomic distance (Å)	Clash overlap (Å)
1:C:339:GLY:HA3	2:C:1147:NAG:HN2	1.32	0.92
1:C:55:PHE:HB3	1:C:270:LEU:HB3	1.57	0.84
1:C:853:GLN:HB3	1:C:858:LEU:HD12	1.71	0.72
1:B:273:ARG:NH2	1:B:290:ASP:OD2	2.25	0.67
1:A:689:SER:OG	1:A:690:GLN:N	2.24	0.67
1:A:129:LYS:HG3	1:A:169:GLU:HG3	1.78	0.66
1:A:342:PHE:O	1:A:509:ARG:NH2	2.29	0.65
1:A:357:ARG:HH22	1:A:394:ASN:HA	1.60	0.65
1:B:69:HIS:HB3	1:B:77:LYS:HZ2	1.63	0.64
1:C:36:VAL:HA	1:C:54:LEU:HD11	1.78	0.64
1:C:277:LEU:HD22	1:C:285:ILE:HD13	1.80	0.63
1:C:36:VAL:HG12	1:C:54:LEU:HD21	1.81	0.62
1:C:36:VAL:HG23	1:C:222:ALA:HA	1.80	0.62
1:C:451:TYR:HB2	1:C:495:TYR:HB2	1.82	0.61
1:B:796:ASP:OD1	1:B:796:ASP:N	2.36	0.59
2:C:1147:NAG:H83	2:C:1147:NAG:H3	1.85	0.59
1:B:97:LYS:HG2	1:B:186:PHE:HA	1.84	0.58
1:A:563:GLN:NE2	1:B:42:VAL:O	2.37	0.58
1:B:661:GLU:OE2	1:B:662:CYS:N	2.37	0.58
1:A:206:LYS:HB3	1:A:223:LEU:HD22	1.86	0.57
1:B:95:THR:O	1:B:264:ALA:N	2.37	0.57
1:B:535:LYS:NZ	1:B:554:GLU:OE2	2.28	0.57
1:C:339:GLY:HA3	2:C:1147:NAG:N2	2.14	0.57
1:A:95:THR:HG22	1:A:189:LEU:HB3	1.86	0.56
1:B:105:ILE:N	1:B:239:GLN:O	2.31	0.56
1:C:424:LYS:HB3	1:C:463:PRO:HA	1.87	0.56
1:B:409:GLN:OE1	1:B:418:ILE:N	2.30	0.56
1:B:574:ASP:N	1:B:574:ASP:OD1	2.37	0.56
1:A:589:PRO:HG3	1:B:855:PHE:CE2	2.41	0.56
1:C:563:GLN:O	1:C:577:ARG:NH2	2.39	0.55
1:B:68:ILE:H	1:B:78:ARG:HB2	1.72	0.55
1:C:52:GLN:HG2	1:C:274:THR:HG22	1.90	0.54
1:A:325:SER:HA	1:A:540:ASN:HB2	1.90	0.54
1:A:139:PRO:HB3	1:A:159:VAL:HA	1.89	0.54
1:A:328:ARG:NH1	1:A:328:ARG:O	2.41	0.53
1:C:403:ARG:NH2	1:C:406:GLU:OE2	2.35	0.53
1:A:106:PHE:HB3	1:A:117:LEU:HB3	1.91	0.53
1:C:213:VAL:HG23	1:C:214:ARG:H	1.73	0.53
1:C:351:TYR:HE1	1:C:452:LEU:HB2	1.74	0.53
1:A:277:LEU:HD13	1:A:285:ILE:HD13	1.91	0.53

Continued on next page...

Not For Manuscript Review

Continued from previous page...

Atom-1	Atom-2	Interatomic distance (Å)	Clash overlap (Å)
1:C:485:GLY:H	1:C:488:CYS:HB2	1.73	0.53
1:B:327:VAL:HG13	1:B:530:SER:HA	1.90	0.52
1:C:326:ILE:HG23	1:C:541:PHE:HA	1.92	0.52
1:A:848:ASP:OD1	1:A:849:LEU:N	2.43	0.52
1:C:439:ASN:HA	1:C:507:PRO:HG2	1.90	0.52
1:B:927:PHE:O	1:B:931:ILE:HG22	2.09	0.52
1:C:1104:VAL:HG23	1:C:1115:ILE:HG12	1.92	0.51
1:A:995:ARG:HA	1:A:998:THR:HG22	1.92	0.51
1:B:411:ALA:HB3	1:B:414:GLN:HG3	1.92	0.51
1:B:40:ASP:OD2	1:B:44:ARG:NH2	2.44	0.51
1:C:605:SER:OG	1:C:606:ASN:N	2.43	0.51
1:C:83:VAL:HG11	1:C:237:ARG:HH11	1.75	0.50
1:A:736:VAL:HG23	1:A:858:LEU:HD23	1.93	0.50
1:B:290:ASP:OD1	1:B:291:CYS:N	2.38	0.50
1:A:662:CYS:HB2	1:A:697:MET:HE3	1.94	0.50
1:C:329:PHE:O	1:C:580:GLN:NE2	2.45	0.49
1:B:707:TYR:HB3	1:C:792:PRO:HG2	1.93	0.49
1:A:328:ARG:HA	1:A:531:THR:HG22	1.94	0.49
1:C:1105:THR:OG1	1:C:1106:GLN:N	2.45	0.49
1:A:804:GLN:OE1	1:A:935:GLN:NE2	2.36	0.49
1:B:1140:PRO:HB2	1:B:1141:LEU:HD22	1.95	0.49
1:C:914:ASN:OD1	1:C:914:ASN:N	2.46	0.49
1:B:485:GLY:H	1:B:488:CYS:HB2	1.77	0.49
1:C:91:TYR:OH	1:C:191:GLU:OE1	2.22	0.49
1:B:428:ASP:OD1	1:B:428:ASP:N	2.36	0.48
1:B:973:ILE:HD11	1:B:992:GLN:HE22	1.78	0.48
1:C:532:ASN:OD1	1:C:533:LEU:N	2.43	0.48
1:A:37:TYR:HE1	1:A:39:PRO:HB3	1.78	0.48
1:C:365:TYR:HB2	1:C:368:LEU:HD12	1.95	0.48
1:A:811:LYS:HG2	1:A:812:PRO:HD2	1.94	0.48
1:A:1086:LYS:HD2	1:A:1122:VAL:HG21	1.95	0.48
1:A:122:ASN:ND2	1:A:125:ASN:OD1	2.36	0.48
1:A:529:LYS:HA	1:A:529:LYS:HD2	1.74	0.47
1:B:47:VAL:HG12	1:B:49:HIS:H	1.79	0.47
1:A:309:GLU:OE1	1:A:309:GLU:N	2.47	0.47
1:B:324:GLU:H	1:B:539:VAL:HG23	1.79	0.47
1:A:712:ILE:HG13	1:B:896:ILE:HG13	1.97	0.47
1:C:409:GLN:HB3	1:C:419:ALA:HB2	1.96	0.47
1:B:915:VAL:O	1:B:919:ASN:ND2	2.48	0.46
1:C:93:ALA:HB3	1:C:266:TYR:HB2	1.97	0.46
1:B:659:SER:HB3	1:B:698:SER:HB2	1.98	0.46

Continued on next page...

Not For Manuscript Review

Continued from previous page...

Atom-1	Atom-2	Interatomic distance (Å)	Clash overlap (Å)
1:C:330:PRO:HB3	1:C:579:PRO:HB2	1.97	0.46
1:C:424:LYS:HG2	1:C:463:PRO:HG3	1.98	0.46
1:C:324:GLU:H	1:C:539:VAL:HG12	1.81	0.46
1:A:226:LEU:HD23	1:A:226:LEU:HA	1.84	0.45
1:A:102:ARG:HB2	1:A:241:LEU:HD11	1.96	0.45
1:B:567:ARG:HA	1:B:572:THR:O	2.16	0.45
1:C:347:PHE:HD2	1:C:509:ARG:HD3	1.81	0.45
1:B:392:PHE:O	1:B:522:ALA:HB1	2.17	0.45
1:A:119:ILE:HG23	1:A:128:ILE:HG12	1.99	0.45
1:A:381:GLY:HA3	1:A:430:THR:HG23	1.99	0.45
1:A:456:PHE:HB2	1:A:491:PRO:HB3	1.98	0.45
1:B:603:ASN:OD1	2:B:1148:NAG:N2	2.49	0.45
1:B:617:CYS:HB2	1:B:644:GLN:HB2	1.98	0.45
1:B:433:VAL:HG22	1:B:512:VAL:HG23	1.98	0.45
1:B:500:THR:O	1:B:500:THR:OG1	2.33	0.45
1:C:347:PHE:CD2	1:C:509:ARG:HD3	2.51	0.45
1:A:419:ALA:HA	1:A:423:TYR:O	2.17	0.44
1:B:49:HIS:NE2	1:B:51:THR:HG22	2.32	0.44
1:B:347:PHE:CE2	1:B:509:ARG:HB3	2.52	0.44
1:B:318:PHE:CZ	1:B:622:VAL:HG12	2.53	0.44
1:B:343:ASN:HB2	2:B:1147:NAG:H2	1.99	0.44
1:B:1117:THR:HG22	1:B:1140:PRO:HD3	2.00	0.44
1:A:560:LEU:O	1:A:577:ARG:NH2	2.50	0.44
1:B:326:ILE:O	1:B:542:ASN:N	2.50	0.44
1:B:709:ASN:OD1	1:B:709:ASN:N	2.51	0.44
1:C:1142:GLN:HB3	1:C:1143:PRO:HD3	1.99	0.44
1:A:67:ALA:HB2	1:A:80:ASP:HB2	1.98	0.43
1:A:455:LEU:N	1:A:491:PRO:O	2.39	0.43
1:B:424:LYS:HB3	1:B:463:PRO:HA	2.00	0.43
1:B:529:LYS:HE2	1:B:529:LYS:HB2	1.58	0.43
1:C:343:ASN:OD1	2:C:1147:NAG:N2	2.51	0.43
1:B:966:LEU:HA	1:B:966:LEU:HD23	1.81	0.43
1:B:1142:GLN:N	1:B:1142:GLN:OE1	2.51	0.43
1:C:83:VAL:HG11	1:C:237:ARG:HD3	2.00	0.43
1:A:402:ILE:HD11	1:A:407:VAL:HG23	2.00	0.43
1:B:455:LEU:N	1:B:491:PRO:O	2.47	0.43
1:B:97:LYS:HZ2	1:B:187:LYS:HG3	1.83	0.43
1:A:375:SER:HA	1:C:486:PHE:HE1	1.83	0.43
1:B:228:ASP:N	1:B:228:ASP:OD1	2.49	0.43
1:B:356:LYS:O	1:B:396:TYR:HB2	2.19	0.43
1:B:984:LEU:HD12	1:B:984:LEU:HA	1.91	0.43

Continued on next page...

Not For Manuscript Review

Continued from previous page...

Atom-1	Atom-2	Interatomic distance (Å)	Clash overlap (Å)
1:C:304:LYS:HE2	1:C:304:LYS:HB3	1.86	0.43
1:B:431:GLY:HA2	1:B:515:PHE:CE2	2.53	0.43
1:C:820:ASP:OD1	1:C:821:LEU:N	2.51	0.43
1:A:787:GLN:OE1	1:C:703:ASN:ND2	2.52	0.43
1:C:537:LYS:HG3	1:C:539:VAL:HG13	2.00	0.43
1:B:61:ASN:HB2	2:B:1145:NAG:H2	2.01	0.43
1:A:368:LEU:HD13	1:A:374:PHE:HZ	1.82	0.42
1:A:654:GLU:OE1	1:A:654:GLU:N	2.49	0.42
1:B:535:LYS:HA	1:B:535:LYS:HD2	1.82	0.42
1:A:37:TYR:H	1:A:55:PHE:HE1	1.67	0.42
1:B:138:ASP:N	1:B:138:ASP:OD1	2.53	0.42
1:A:403:ARG:HD2	1:A:405:ASP:OD1	2.20	0.42
1:A:984:LEU:HD23	1:A:984:LEU:HA	1.91	0.42
1:A:1117:THR:HG22	1:A:1140:PRO:HD3	2.01	0.42
1:C:826:VAL:HB	1:C:945:LEU:HD12	2.01	0.42
1:A:943:SER:O	1:A:943:SER:OG	2.35	0.42
1:C:409:GLN:OE1	1:C:419:ALA:N	2.42	0.42
1:B:502:GLY:O	1:B:506:GLN:NE2	2.53	0.41
1:C:335:LEU:HD12	1:C:338:PHE:HZ	1.85	0.41
1:B:97:LYS:HZ3	1:B:186:PHE:N	2.18	0.41
1:B:189:LEU:HD12	1:B:189:LEU:HA	1.89	0.41
1:C:202:LYS:HB3	1:C:202:LYS:HE2	1.81	0.41
1:C:921:LYS:HA	1:C:921:LYS:HD3	1.87	0.41
1:C:986:PRO:N	1:C:987:PRO:HD2	2.35	0.41
1:B:128:ILE:HD12	1:B:170:TYR:HD2	1.85	0.41
1:B:776:LYS:HB3	1:B:776:LYS:HE3	1.60	0.41
1:C:417:LYS:HA	1:C:417:LYS:HD3	1.94	0.41
1:C:822:LEU:O	1:C:826:VAL:HG12	2.19	0.41
1:A:131:CYS:HB2	1:A:133:PHE:CZ	2.56	0.41
1:C:455:LEU:HD23	1:C:456:PHE:CE2	2.55	0.41
1:A:305:SER:OG	1:A:306:PHE:N	2.54	0.41
1:A:537:LYS:HG3	1:A:539:VAL:HG13	2.03	0.41
1:C:393:THR:HB	1:C:522:ALA:HA	2.03	0.41
1:C:599:THR:HA	1:C:600:PRO:HD3	1.90	0.41
1:B:383:SER:HA	1:B:384:PRO:HD3	1.94	0.40
1:C:131:CYS:H	1:C:133:PHE:HE2	1.69	0.40
1:C:506:GLN:HE21	1:C:508:TYR:HE1	1.68	0.40
1:A:204:TYR:HD1	1:A:225:PRO:HA	1.86	0.40
1:B:650:LEU:HD12	1:B:650:LEU:HA	1.91	0.40
1:C:229:LEU:HB2	1:C:231:ILE:HD11	2.04	0.40
1:A:441:LEU:HD12	1:A:441:LEU:HA	1.92	0.40

Continued on next page...

Not For Manuscript Review

Continued from previous page...

Atom-1	Atom-2	Interatomic distance (Å)	Clash overlap (Å)
1:B:332:ILE:HG22	1:B:362:VAL:HG21	2.03	0.40
1:B:468:ILE:HA	1:B:468:ILE:HD12	1.88	0.40
1:B:767:LEU:HD23	1:B:767:LEU:HA	1.96	0.40
1:C:338:PHE:CD1	1:C:338:PHE:N	2.89	0.40
1:A:99:ASN:HB3	1:A:102:ARG:NH2	2.36	0.40
1:B:200:TYR:CE2	1:B:228:ASP:HB2	2.56	0.40
1:C:142:GLY:HA3	1:C:156:GLU:HB2	2.04	0.40
1:C:383:SER:HB3	1:C:386:LYS:HG3	2.02	0.40
1:B:104:TRP:HA	1:B:240:THR:HA	2.03	0.40
1:C:131:CYS:HB2	1:C:133:PHE:CE2	2.56	0.40
1:C:182:LYS:HE2	1:C:182:LYS:HB3	1.77	0.40
1:C:1023:ASN:O	1:C:1027:THR:HG23	2.21	0.40

There are no symmetry-related clashes.

5.3 Torsion angles (i)

5.3.1 Protein backbone (i)

In the following table, the Percentiles column shows the percent Ramachandran outliers of the chain as a percentile score with respect to all PDB entries followed by that with respect to all EM entries.

The Analysed column shows the number of residues for which the backbone conformation was analysed, and the total number of residues.

Mol	Chain	Analysed	Favoured	Allowed	Outliers	Percentiles	
1	A	1001/1276 (78%)	946 (94%)	54 (5%)	1 (0%)	51	51
1	B	1023/1276 (80%)	950 (93%)	73 (7%)	0	100	100
1	C	1025/1276 (80%)	958 (94%)	67 (6%)	0	100	100
All	All	3049/3828 (80%)	2854 (94%)	194 (6%)	1 (0%)	100	100

All (1) Ramachandran outliers are listed below:

Mol	Chain	Res	Type
1	A	39	PRO

Not For Manuscript Review

5.3.2 Protein sidechains (i)

In the following table, the Percentiles column shows the percent sidechain outliers of the chain as a percentile score with respect to all PDB entries followed by that with respect to all EM entries.

The Analysed column shows the number of residues for which the sidechain conformation was analysed, and the total number of residues.

Mol	Chain	Analysed	Rotameric	Outliers	Percentiles	
1	A	892/1106 (81%)	866 (97%)	26 (3%)	42	42
1	B	908/1106 (82%)	881 (97%)	27 (3%)	41	41
1	C	906/1106 (82%)	877 (97%)	29 (3%)	39	39
All	All	2706/3318 (82%)	2624 (97%)	82 (3%)	44	41

All (82) residues with a non-rotameric sidechain are listed below:

Mol	Chain	Res	Type
1	A	36	VAL
1	A	42	VAL
1	A	50	SER
1	A	79	PHE
1	A	92	PHE
1	A	94	SER
1	A	111	ASP
1	A	175	PHE
1	A	189	LEU
1	A	207	HIS
1	A	220	PHE
1	A	266	TYR
1	A	317	ASN
1	A	328	ARG
1	A	329	PHE
1	A	334	ASN
1	A	338	PHE
1	A	353	TRP
1	A	378	LYS
1	A	408	ARG
1	A	448	ASN
1	A	535	LYS
1	A	538	CYS
1	A	555	SER
1	A	592	PHE
1	A	820	ASP

Continued on next page...

Not For Manuscript Review

Continued from previous page...

Mol	Chain	Res	Type
1	B	32	PHE
1	B	79	PHE
1	B	104	TRP
1	B	151	SER
1	B	153	MET
1	B	160	TYR
1	B	241	LEU
1	B	266	TYR
1	B	270	LEU
1	B	317	ASN
1	B	329	PHE
1	B	347	PHE
1	B	351	TYR
1	B	355	ARG
1	B	377	PHE
1	B	489	TYR
1	B	599	THR
1	B	606	ASN
1	B	675	GLN
1	B	703	ASN
1	B	776	LYS
1	B	873	TYR
1	B	895	GLN
1	B	940	SER
1	B	1045	LYS
1	B	1091	ARG
1	B	1139	ASP
1	C	53	ASP
1	C	145	TYR
1	C	157	PHE
1	C	172	SER
1	C	214	ARG
1	C	220	PHE
1	C	248	TYR
1	C	334	ASN
1	C	347	PHE
1	C	375	SER
1	C	389	ASP
1	C	391	CYS
1	C	392	PHE
1	C	453	TYR
1	C	490	PHE

Continued on next page...

Not For Manuscript Review

Continued from previous page...

Mol	Chain	Res	Type
1	C	495	TYR
1	C	528	LYS
1	C	533	LEU
1	C	540	ASN
1	C	571	ASP
1	C	590	CYS
1	C	616	ASN
1	C	649	CYS
1	C	759	PHE
1	C	779	GLN
1	C	867	ASP
1	C	902	MET
1	C	967	SER
1	C	982	SER

Sometimes sidechains can be flipped to improve hydrogen bonding and reduce clashes. There are no such sidechains identified.

5.3.3 RNA (i)

There are no RNA molecules in this entry.

5.4 Non-standard residues in protein, DNA, RNA chains (i)

There are no non-standard protein/DNA/RNA residues in this entry.

5.5 Carbohydrates (i)

There are no monosaccharides in this entry.

5.6 Ligand geometry (i)

13 ligands are modelled in this entry.

In the following table, the Counts columns list the number of bonds (or angles) for which Mogul statistics could be retrieved, the number of bonds (or angles) that are observed in the model and the number of bonds (or angles) that are defined in the Chemical Component Dictionary. The Link column lists molecule types, if any, to which the group is linked. The Z score for a bond length (or angle) is the number of standard deviations the observed value is removed from the expected value. A bond length (or angle) with $|Z| > 2$ is considered an outlier worth inspection. RMSZ is the root-mean-square of all Z scores of the bond lengths (or angles).

Not For Manuscript Review

Mol	Type	Chain	Res	Link	Bond lengths			Bond angles		
					Counts	RMSZ	#|Z| > 2	Counts	RMSZ	#|Z| > 2
2	NAG	A	1149	1	14,14,15	0.18	0	17,19,21	0.44	0
2	NAG	C	1146	1	14,14,15	0.22	0	17,19,21	0.47	0
2	NAG	C	1149	1	14,14,15	0.19	0	17,19,21	0.38	0
2	NAG	C	1145	1	14,14,15	0.34	0	17,19,21	0.40	0
2	NAG	B	1149	1	14,14,15	0.25	0	17,19,21	0.36	0
2	NAG	A	1148	1	14,14,15	0.25	0	17,19,21	0.36	0
2	NAG	B	1147	1	14,14,15	0.28	0	17,19,21	0.47	0
2	NAG	C	1147	1	14,14,15	0.57	0	17,19,21	1.27	1 (5%)
2	NAG	C	1148	1	14,14,15	0.29	0	17,19,21	0.56	0
2	NAG	B	1145	1	14,14,15	0.28	0	17,19,21	0.36	0
2	NAG	B	1148	1	14,14,15	0.39	0	17,19,21	0.48	0
2	NAG	B	1146	1	14,14,15	0.20	0	17,19,21	0.39	0
2	NAG	A	1145	1	14,14,15	0.27	0	17,19,21	0.52	0

In the following table, the Chirals column lists the number of chiral outliers, the number of chiral centers analysed, the number of these observed in the model and the number defined in the Chemical Component Dictionary. Similar counts are reported in the Torsion and Rings columns. '-' means no outliers of that kind were identified.

Mol	Type	Chain	Res	Link	Chirals	Torsions	Rings
2	NAG	A	1149	1	-	2/6/23/26	0/1/1/1
2	NAG	C	1146	1	-	2/6/23/26	0/1/1/1
2	NAG	C	1149	1	-	4/6/23/26	0/1/1/1
2	NAG	C	1145	1	-	4/6/23/26	0/1/1/1
2	NAG	B	1149	1	-	0/6/23/26	0/1/1/1
2	NAG	A	1148	1	-	3/6/23/26	0/1/1/1
2	NAG	B	1147	1	-	1/6/23/26	0/1/1/1
2	NAG	C	1147	1	-	5/6/23/26	0/1/1/1
2	NAG	C	1148	1	-	3/6/23/26	0/1/1/1
2	NAG	B	1145	1	-	2/6/23/26	0/1/1/1
2	NAG	B	1148	1	-	2/6/23/26	0/1/1/1
2	NAG	B	1146	1	-	3/6/23/26	0/1/1/1
2	NAG	A	1145	1	-	3/6/23/26	0/1/1/1

There are no bond length outliers.

All (1) bond angle outliers are listed below:

Mol	Chain	Res	Type	Atoms	Z	Observed(^{o})	Ideal(^{o})
2	C	1147	NAG	C2-N2-C7	4.33	129.07	122.90

Not For Manuscript Review

There are no chirality outliers.

All (34) torsion outliers are listed below:

Mol	Chain	Res	Type	Atoms
2	C	1149	NAG	O5-C5-C6-O6
2	A	1148	NAG	O5-C5-C6-O6
2	B	1145	NAG	O5-C5-C6-O6
2	C	1146	NAG	O5-C5-C6-O6
2	A	1149	NAG	O5-C5-C6-O6
2	C	1146	NAG	C4-C5-C6-O6
2	C	1149	NAG	C4-C5-C6-O6
2	C	1145	NAG	O5-C5-C6-O6
2	A	1148	NAG	C4-C5-C6-O6
2	B	1145	NAG	C4-C5-C6-O6
2	A	1145	NAG	C4-C5-C6-O6
2	A	1149	NAG	C4-C5-C6-O6
2	C	1145	NAG	C4-C5-C6-O6
2	B	1146	NAG	C8-C7-N2-C2
2	B	1146	NAG	O7-C7-N2-C2
2	C	1145	NAG	C8-C7-N2-C2
2	C	1145	NAG	O7-C7-N2-C2
2	C	1147	NAG	C8-C7-N2-C2
2	C	1147	NAG	O7-C7-N2-C2
2	C	1149	NAG	C8-C7-N2-C2
2	C	1149	NAG	O7-C7-N2-C2
2	C	1147	NAG	O5-C5-C6-O6
2	A	1145	NAG	O5-C5-C6-O6
2	C	1148	NAG	C4-C5-C6-O6
2	B	1148	NAG	O5-C5-C6-O6
2	C	1148	NAG	O5-C5-C6-O6
2	B	1146	NAG	O5-C5-C6-O6
2	A	1148	NAG	C1-C2-N2-C7
2	C	1148	NAG	C3-C2-N2-C7
2	C	1147	NAG	C4-C5-C6-O6
2	B	1148	NAG	C1-C2-N2-C7
2	A	1145	NAG	C3-C2-N2-C7
2	B	1147	NAG	C3-C2-N2-C7
2	C	1147	NAG	C3-C2-N2-C7

There are no ring outliers.

4 monomers are involved in 7 short contacts:

Mol	Chain	Res	Type	Clashes	Symm-Clashes
2	B	1147	NAG	1	0

Continued on next page...

Not For Manuscript Review

Continued from previous page...

Mol	Chain	Res	Type	Clashes	Symm-Clashes
2	C	1147	NAG	4	0
2	B	1145	NAG	1	0
2	B	1148	NAG	1	0

5.7 Other polymers (i)

There are no such residues in this entry.

5.8 Polymer linkage issues (i)

There are no chain breaks in this entry.

Not For Manuscript Review

6 Map visualisation (i)

This section contains visualisations of the EMDB entry D_1300032715. These allow visual inspection of the internal detail of the map and identification of artifacts.

Images derived from a raw map, generated by summing the deposited half-maps, are presented below the corresponding image components of the primary map to allow further visual inspection and comparison with those of the primary map.

6.1 Orthogonal projections (i)

6.1.1 Primary map

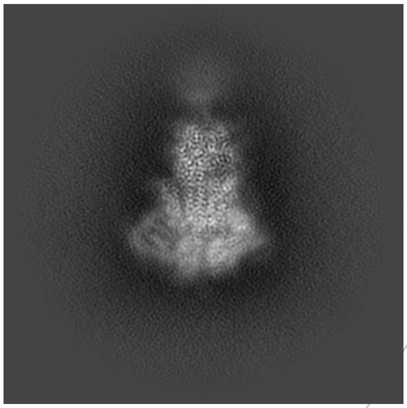

X

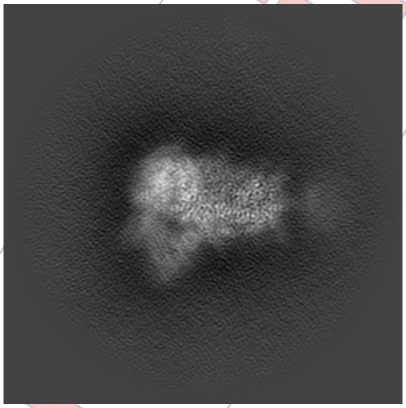

Y

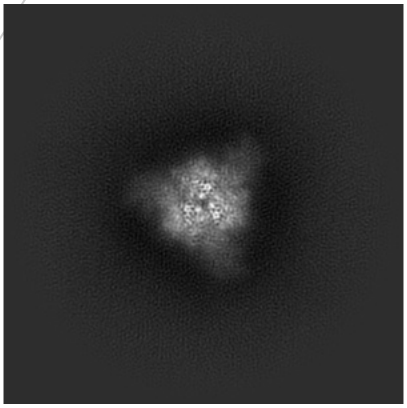

Z

6.1.2 Raw map

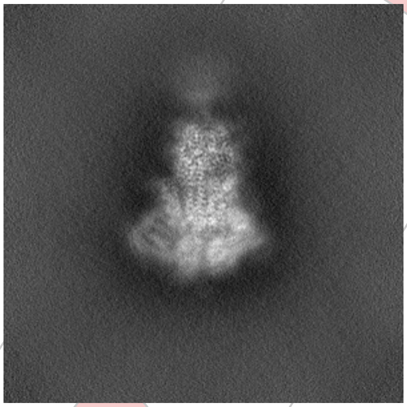

X

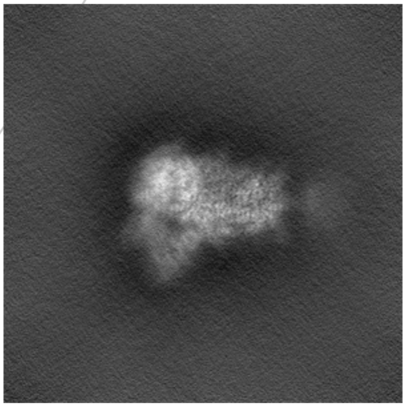

Y

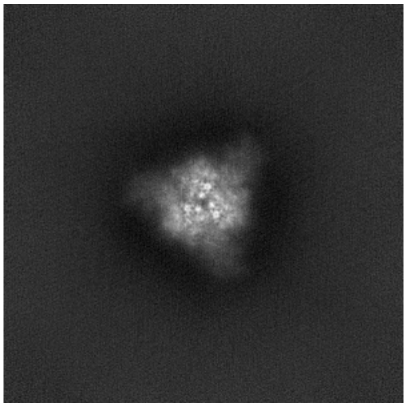

Z

The images above show the map projected in three orthogonal directions.

Not For Manuscript Review

Not For Manuscript Review

6.2 Central slices (i)

6.2.1 Primary map

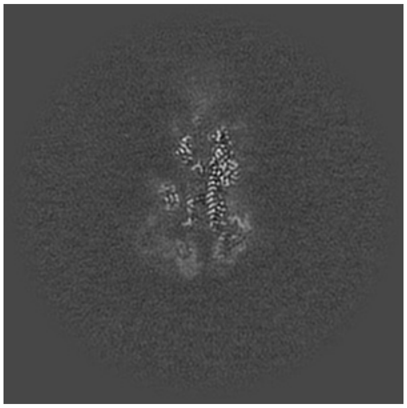
X Index: 240

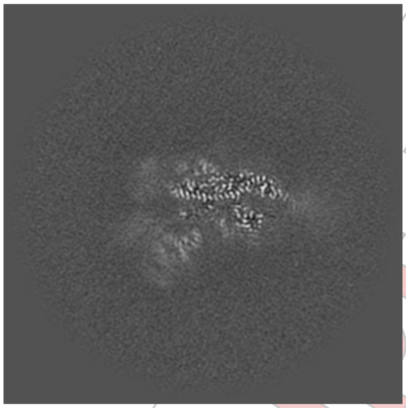
Y Index: 240

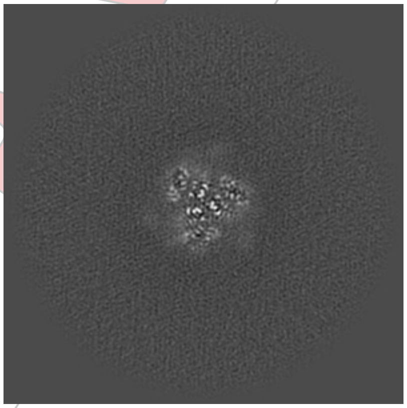
Z Index: 240

6.2.2 Raw map

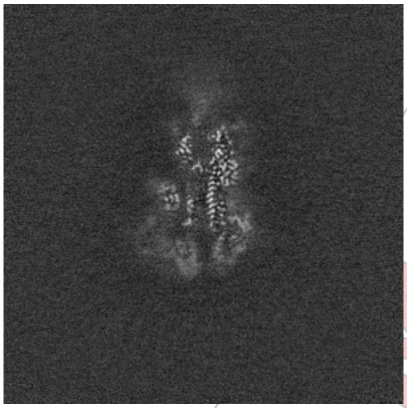
X Index: 240

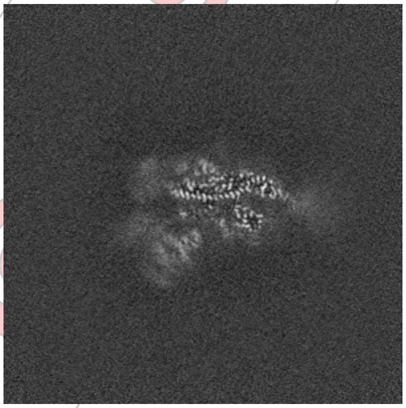
Y Index: 240

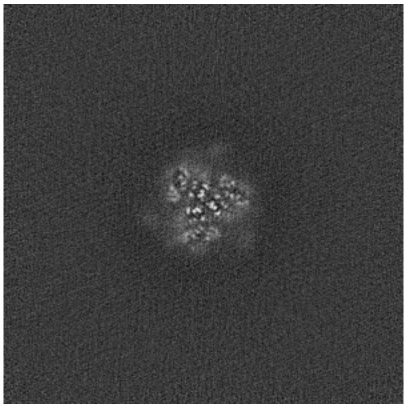
Z Index: 240

The images above show central slices of the map in three orthogonal directions.

6.3 Largest variance slices ⓘ

6.3.1 Primary map

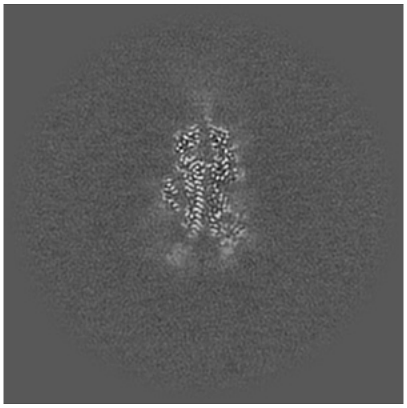
X Index: 232

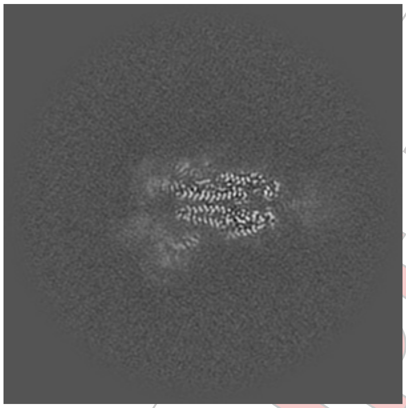
Y Index: 236

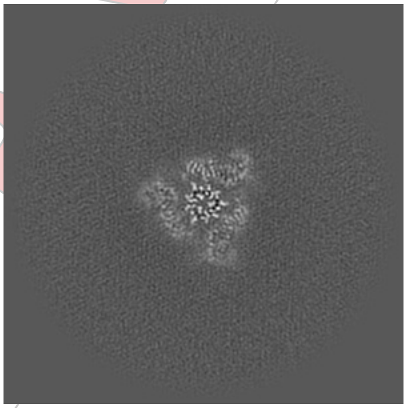
Z Index: 222

6.3.2 Raw map

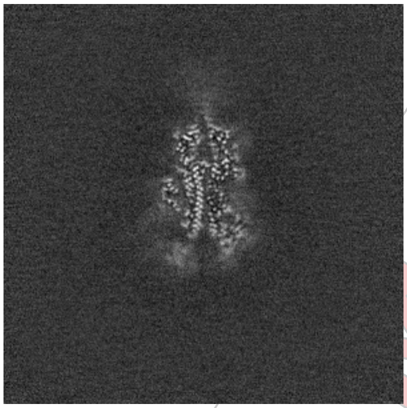
X Index: 233

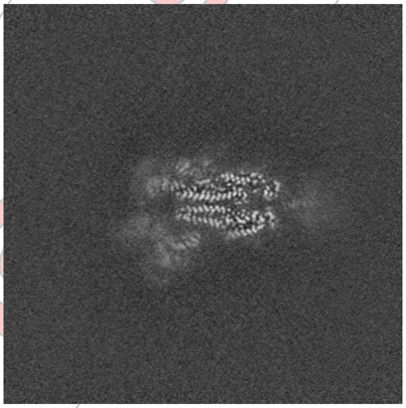
Y Index: 236

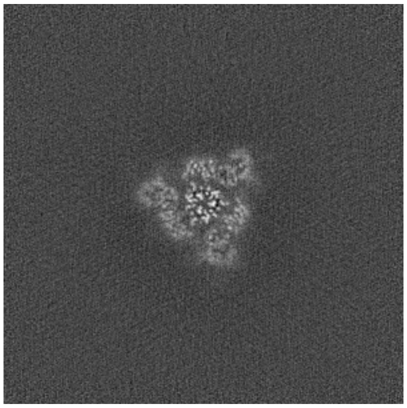
Z Index: 221

The images above show the largest variance slices of the map in three orthogonal directions.

Not For Manuscript Review

6.4 Orthogonal surface views (i)

6.4.1 Primary map

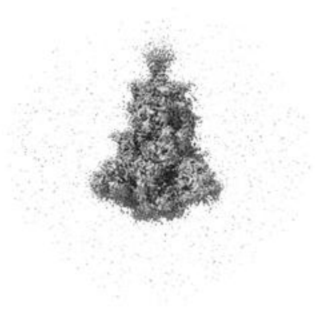

X

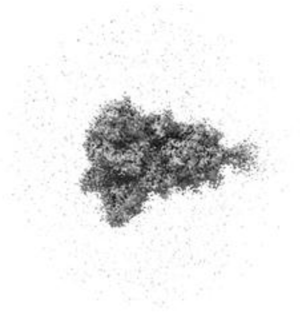

Y

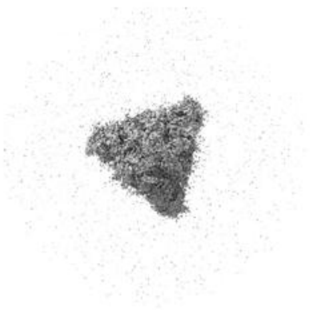

Z

The images above show the 3D surface view of the map at the recommended contour level 0.4. These images, in conjunction with the slice images, may facilitate assessment of whether an appropriate contour level has been provided.

6.4.2 Raw map

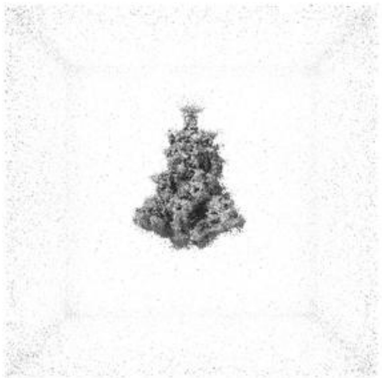

X

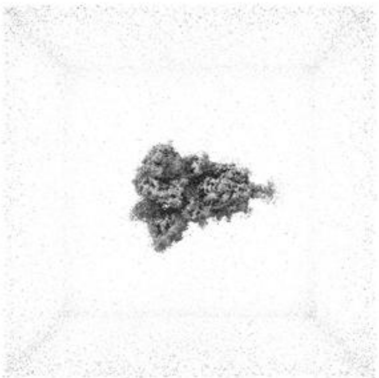

Y

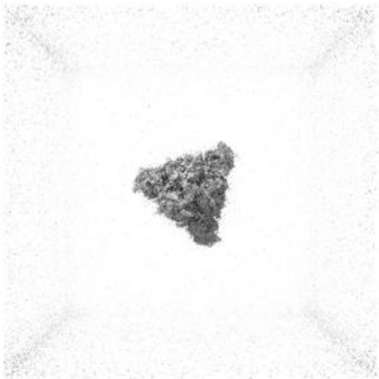

Z

These images show the 3D surface of the raw map. The raw map's contour level was selected so that its surface encloses the same volume as the primary map does at its recommended contour level.

6.5 Mask visualisation (i)

This section was not generated. No masks/segmentation were deposited.

Not For Manuscript Review

7 Map analysis (i)

This section contains the results of statistical analysis of the map.

7.1 Map-value distribution (i)

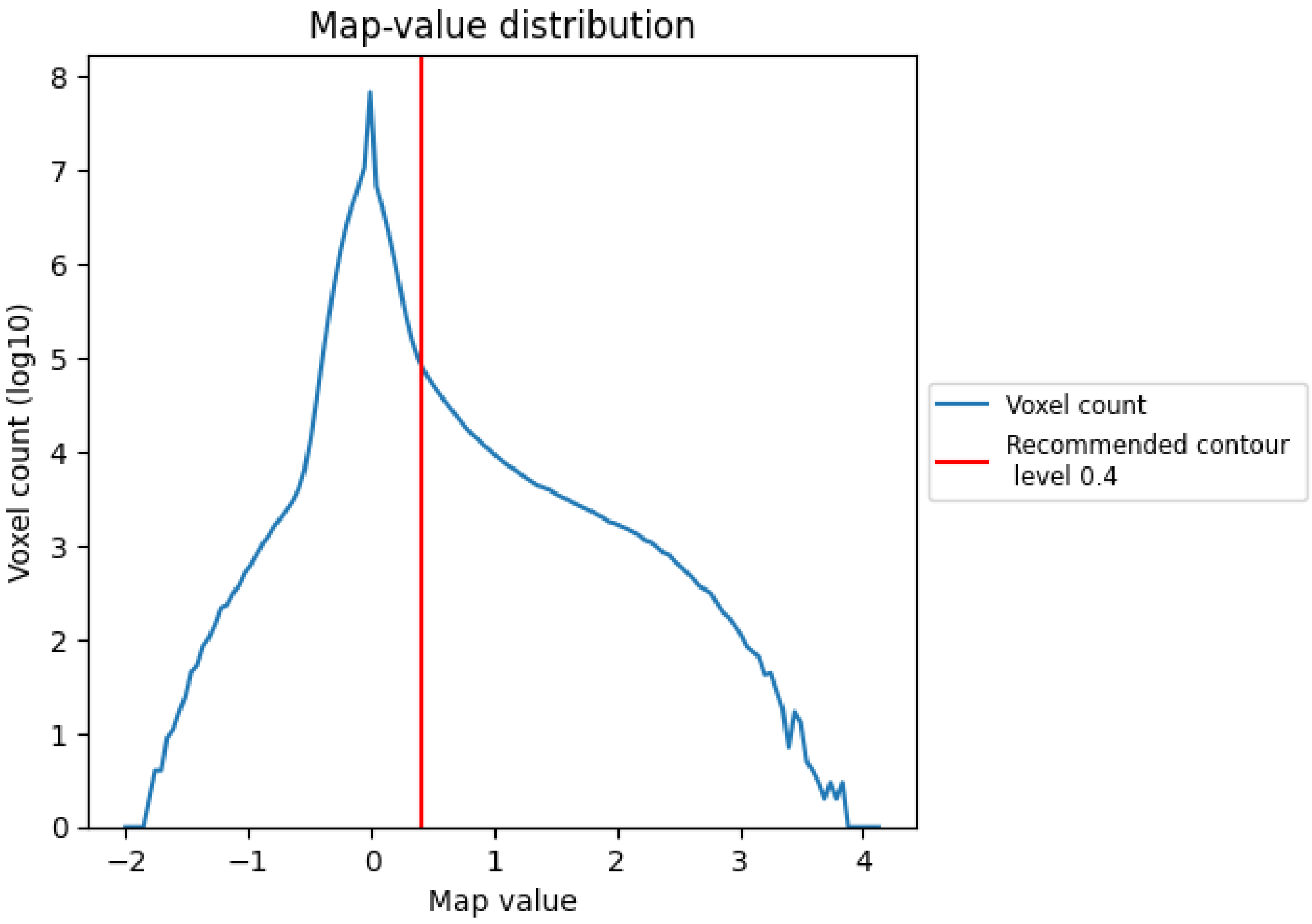

The map-value distribution is plotted in 128 intervals along the x-axis. The y-axis is logarithmic. A spike in this graph at zero usually indicates that the volume has been masked.

Not For Review

7.2 Volume estimate (i)

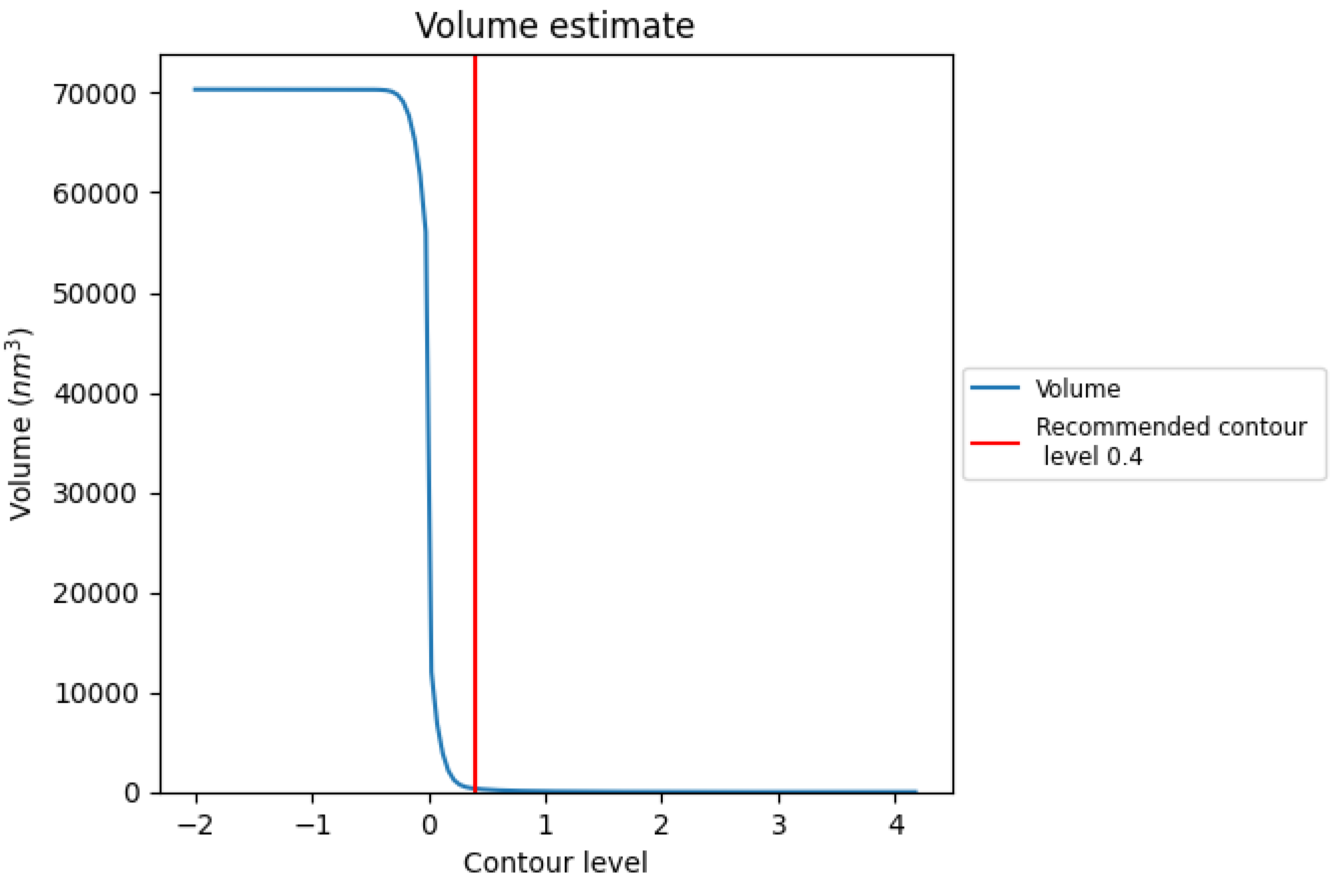

The volume at the recommended contour level is 333 nm^3; this corresponds to an approximate mass of 301 kDa.

The volume estimate graph shows how the enclosed volume varies with the contour level. The recommended contour level is shown as a vertical line and the intersection between the line and the curve gives the volume of the enclosed surface at the given level.

Not For Manuscript Review

7.3 Rotationally averaged power spectrum ⓘ

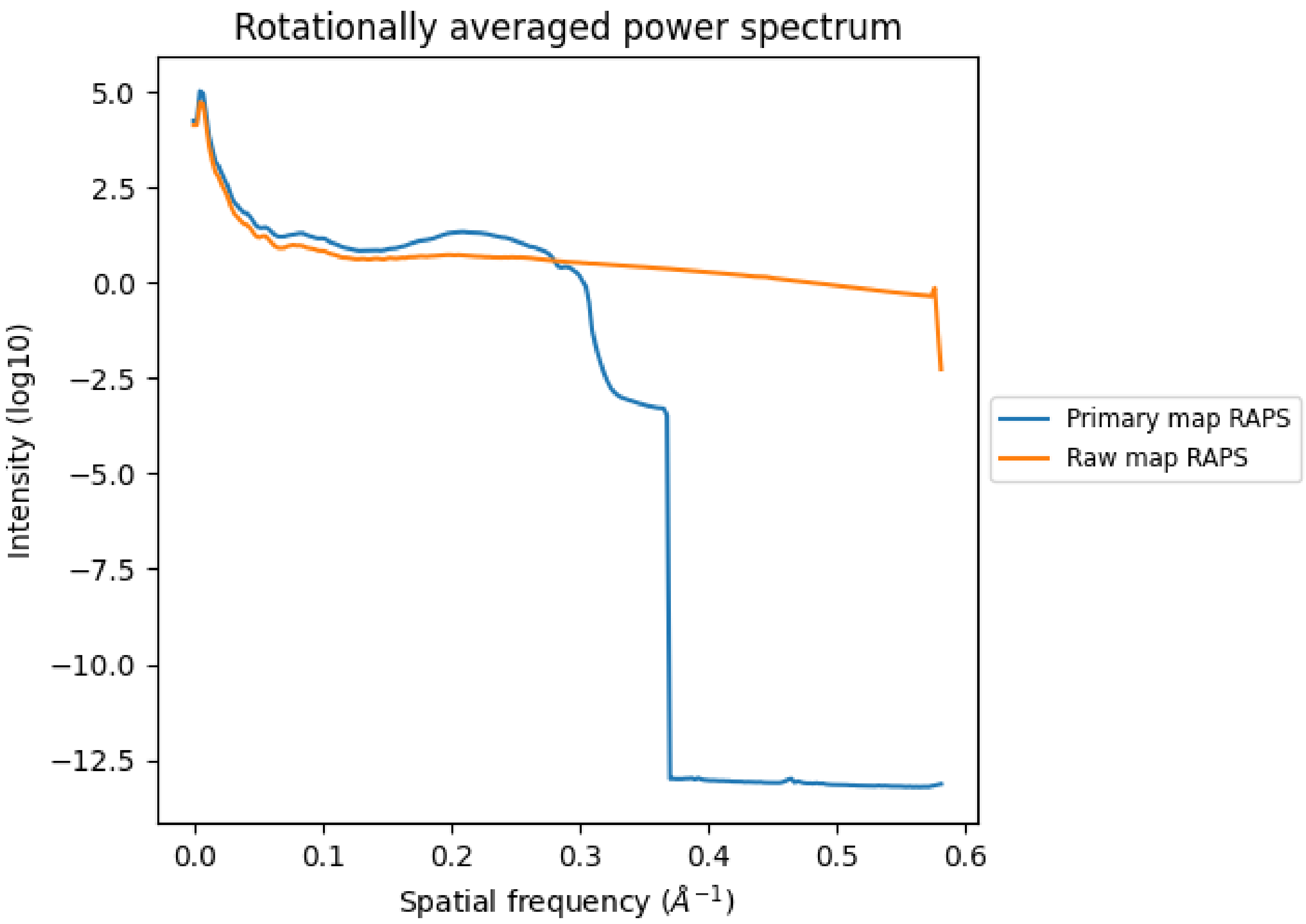

Not For Manuscript Review

8 Fourier-Shell correlation (i)

This section was not generated. No FSC curve or half-maps provided.

Not For Manuscript Review

9 Map-model fit (i)

This section contains information regarding the fit between EMDB map D_1300032715 and PDB model D_1300032715. Per-residue inclusion information can be found in section 3 on page 5.

9.1 Map-model overlay (i)

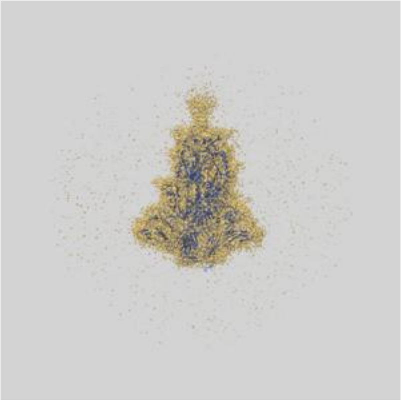

X

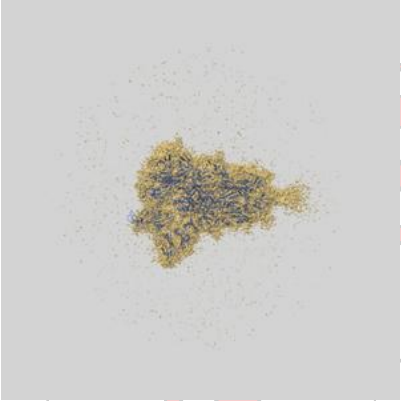

Y

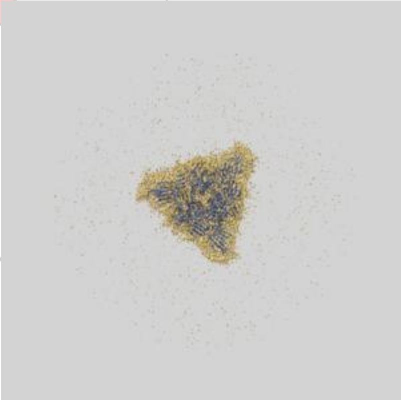

Z

The images above show the 3D surface view of the map at the recommended contour level 0.4 at 50% transparency in yellow overlaid with a ribbon representation of the model coloured in blue. These images allow for the visual assessment of the quality of fit between the atomic model and the map.

Not For Manuscript Review

9.2 Q-score mapped to coordinate model (i)

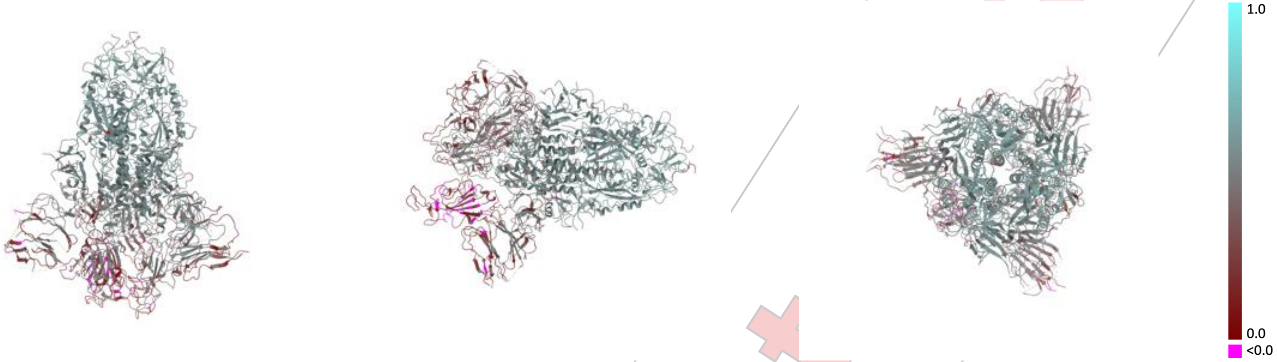

X Y Z

The images above show the model with each residue coloured according its Q-score. This shows their resolvability in the map with higher Q-score values reflecting better resolvability. Please note: Q-score is calculating the resolvability of atoms, and thus high values are only expected at resolutions at which atoms can be resolved. Low Q-score values may therefore be expected for many entries.

9.3 Atom inclusion mapped to coordinate model (i)

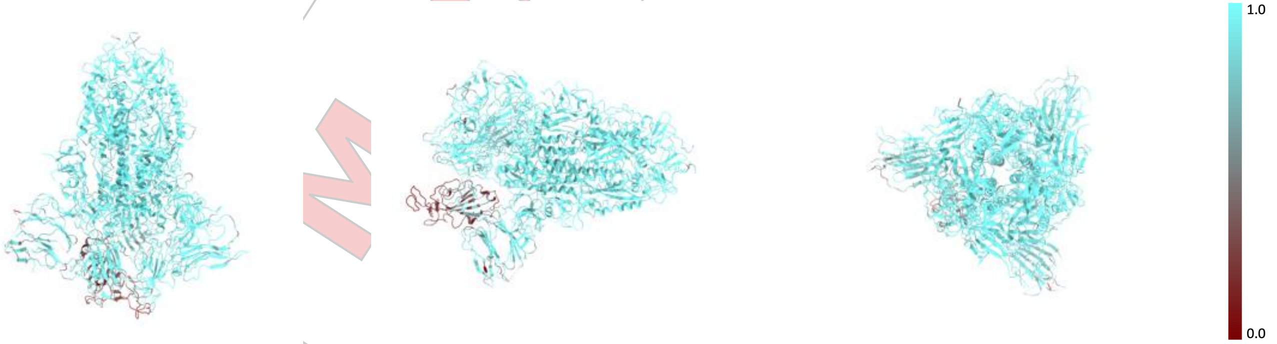

X Y Z

The images above show the model with each residue coloured according to its atom inclusion. This shows to what extent they are inside the map at the recommended contour level (0.4).

Not For Manuscript Review

9.4 Atom inclusion (i)

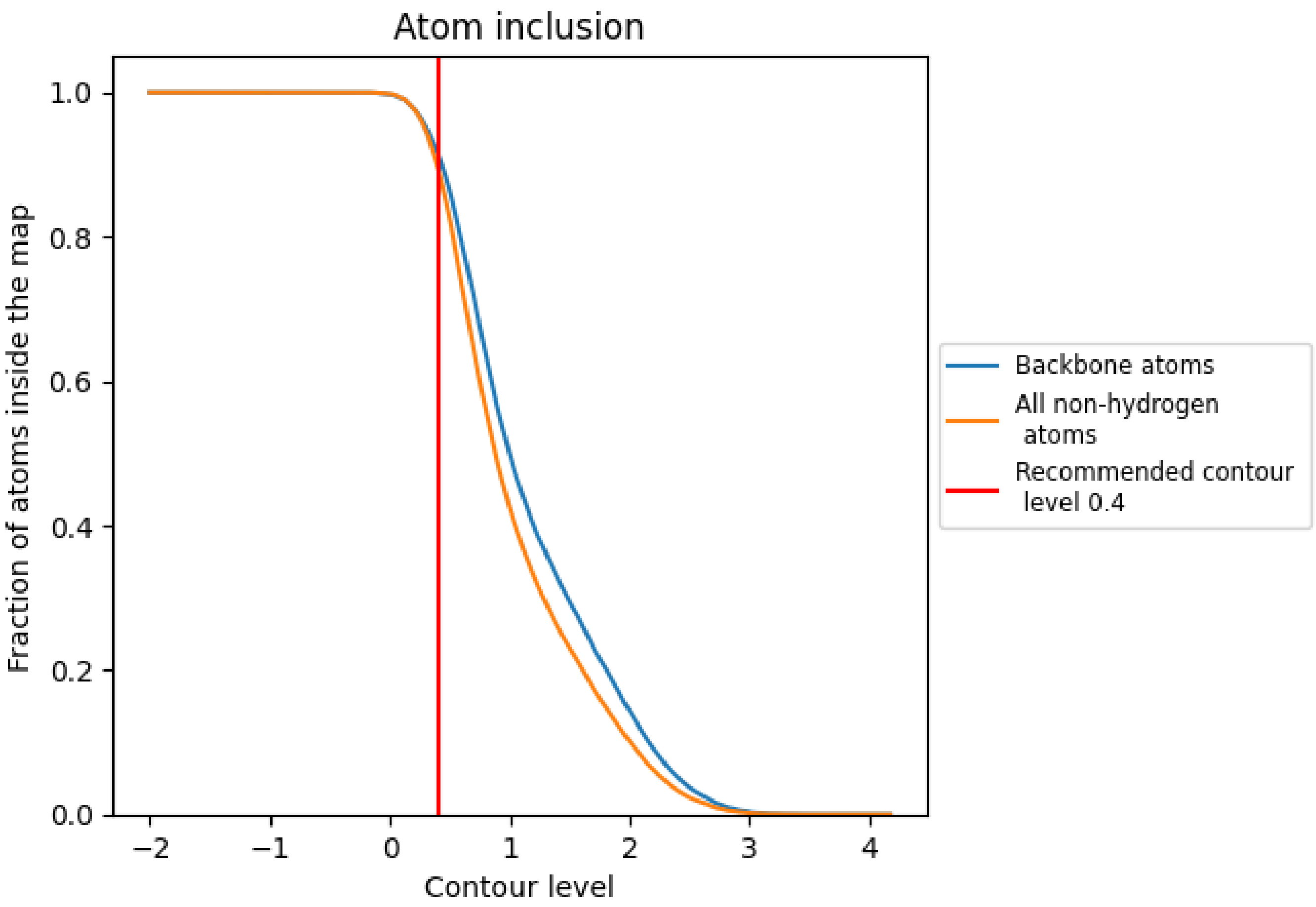

At the recommended contour level, 92% of all backbone atoms, 90% of all non-hydrogen atoms, are inside the map.

Not For Manuscript Review

9.5 Map-model fit summary (i)

The table lists the average atom inclusion at the recommended contour level (0.4) and Q-score for the entire model and for each chain.

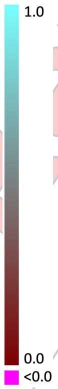

Chain	Atom inclusion	Q-score
All	0.8959	0.4480
A	0.8197	0.4150
B	0.9257	0.4640
C	0.9410	0.4650

Not For Manuscript Review
