# Supplementary material for: In Silico Discovery of Small Molecule Modulators Targeting the Achilles’ Heel of SARS-CoV-2 Spike Protein
Source: ACS Cent Sci. 2023 Feb 8;9(2):252–65. doi: 10.1021/acscentsci.2c01190 (PMC9924089; doi:10.1021/acscentsci.2c01190)

Supporting Information

for

In Silico Discovery of Small Molecule Modulators Targeting the Achilles' Heel of SARS-CoV-2 Spike Protein

Qing Wang [1,2‡], Fanhao Meng [3‡], Yuting Xie [2], Wei Wang [2], Yumin Meng [4], Linjie Li [4], Tao Liu [5], Jianxun Qi [4], Xiaodan Ni [3], Sanduo Zheng [2,5], Jianhui Huang [1] and Niu Huang [2,5,*]*

[1]School of Pharmaceutical Science and Technology, Tianjin University, Tianjin 300072, China

[2]National Institute of Biological Sciences, No. 7 Science Park Road, Zhongguancun Life Science Park, Beijing 102206, China

[3]Shuimu Biosciences, No. 7 Science Park Road, Zhongguancun Life Science Park, Beijing 102206, China

[4]CAS Key Laboratory of Pathogen Microbiology and Immunology, Institute of Microbiology, Chinese Academy of Sciences, Beijing 100101, China

[5]Tsinghua Institute of Multidisciplinary Biomedical Research, Tsinghua University, Beijing 102206, China

*Correspondence should be addressed to J.H. (jhuang@tju.edu.cn) and N.H. (huangniu@nibs.ac.cn)

Content

Table S1. Fifty-four compounds selected for SPR assay after the hierarchical virtual screening. S3

Table S2. Binding affinities of commercially available analogs of SPC-3 and SPC-5 ....S7

Table S3. Binding affinities of commercially available analogs of SPC-6..S9

Table S4. Aggregation evaluation of representative compounds using dynamic light scattering S10

Table S5. Cryo-EM data collection, refinement and validation statistics...S12

Figure S1. Docking poses of commercially available analogs of SPC-2 against the FFA-binding pocket...S13

Concentration response curves and steady-state binding curves in SPR assay S14

^{1}H NMR spectra and ^{13}C NMR/LC-MS spectra of synthesized compounds S32

^{1}H NMR spectra and LC-MS spectra of commercially available compounds S48

Table S1. Fifty-four compounds selected for SPR assay after the hierarchical virtual screening.

Response rank	Vendor ID (Vendor)	Response at 50 μM (%)	K_D (μM)[a]	MM-GB/SA rank	Smiles
1 (SPC-3)	Z92036426 (Enamine)	51.8	19.8	5	Cn1c(nnc1SCc2nc(no2)c3ccccc3Cl)c4ccccc4F
2 (SPC-5)	7966009 (ChemBridge)	46.1	31.4	7	Cn1c(nnc1SCc2nc(no2)c3ccc(cc3)OC)c4ccccc4
3 (SPC-1)	Z25218156 (Enamine)	46	15.5	425	COc1cccc(c1)C(=O)COC(=O)c2ccc(cc2O)O
4 (SPC-2)	Z356729938 (Enamine)	40.2	18.7	935	Cc1nc(cs1)CSCCC(=O)N2CCc3c2cc(cc3)[N+](=O)[O-]
5 (SPC-6)	STK861013 (Vitas-M)	35.9	54.1	302	COc1cccc(c1)c2nc(on2)CCC(=O)Nc3cccnc3
6 (SPC-4)	23835750 (ChemBridge)	30.4	25.1	878	c1(oc(c2c(Cl)cccc2)cc1)C(=O)N(CCc1cn(nc1)C)C
7	STL416744 (Vitas-M)	30.3	164	4	Cn1c(nnc1SCc2nc(no2)c3ccccc3)c4ccco4
8	F6412-1980 (Life chemicals)	27.1	n.d.	127	S(=O)(=O)(c1sccc1)NCC#CCOc1cc(C(F)(F)F)ccc1
9	9502 (Maybridge)	26.3	n.d.	192	Cc1c(ccc(n1)C(F)(F)F)c2nnc(o2)SCc3ccc(cc3)OC(F)(F)F
10	C592-3799 (Chemdiv)	25.5	n.d.	82	Cn1c(nnc1SCC(=O)NCCO)CSc2nc3ccccc3s2
11	0606-5952 (Chemdiv)	24.2	n.d.	556	CCCCCCCCOc1ccc(cc1)OC(=O)c2ccco2
12	0054-0201 (Chemdiv)	23.5	n.d.	582	CCCCCC(=O)Oc1ccc(cc1)/N=N/c2ccc(cc2)C#N
13	C711-0480 (Chemdiv)	23.4	n.d.	31	COC(=O)c1ccc2c(c1)C(=O)/C(=C/c3ccc(o3)c4cccc(c4)C(F)(F)F)/O2
14	6536 (Maybridge)	23.3	n.d.	139	CC(C)(C)c1cc2nc(nc(n2n1)N)SC
15	F6064-2602 (Life chemicals)	22.3	n.d.	2	n1c(C2CN(C(=O)CCn3c(=O)[nH]c(=O)cc3)C2)onc1c1cc(Cl)ccc1
16	0584-0466 (Chemdiv)	21.5	n.d.	383	Cc1cccc(c1)N2C(=O)c3ccc(cc3C2=O)Oc4ccc(cc4)NC(=O)C
17	T6080959 (Enamine)	21.2	n.d.	34	COc1ccc(cc1)c2nnc(o2)SCc3nc(no3)c4ccco4
18	F2496-3133	21.1	n.d.	24	[nH]1c(=O)n(ccc1=O)CCC(=O)OCc1cc(on1)

	(Life chemicals)				c1occc1
19	2166-0281 (Chemdiv)	21	n.d.	934	CCN(CC)c1ccc(cc1)/C=N/c2ccc(cc2)S(=O)(=O)N
20	AQ-776/42801545 (Specs)	19.5	n.d.	534	CC(=O)Nc1ccc(cc1)c2cn3cc(ccc3n2)Sc4ccccc4
21	AE-641/08724023 (Specs)	19	n.d.	576	c1cc(ccc1C#N)/N=C/c2cc(ccc2Cl)[N+](=O)[O-]
22	F2493-3278 (Life chemicals)	18.5	n.d.	56	[nH]1c(=O)n(ccc1=O)CCC(=O)NCc1cc(on1)c1c(F)cccc1
23	T6037875 (Enamine)	18.3	n.d.	734	Cc1c(sc(n1)C)c2nnc(o2)SCc3c(oc(n3)c4cccs4)C
24	T6388553 (Enamine)	18.3	n.d.	200	Cc1cc(c(n1C)C)C(=O)CSc2nnc(o2)c3nnc(o3)c4ccccc4
25	2082-0203 (Chemdiv)	17.9	n.d.	32	CC(=O)Nc1ccc(cc1)C2=N/C(=C/c3cccc(c3)OC)/C(=O)O2
26	AP-501/43286897	17.8	n.d.	137	COc1ccc(cc1)/C=C/c2nn3c(nnc3s2)Cc4ccccc4F
27	T6013936 (Enamine)	17.1	n.d.	43	c1ccc(cc1)c2nnc(o2)SCCCSc3nnc(o3)c4ccccc4
28	18297954 (ChemBridge)	16.5	n.d.	602	n1c(noc1CCc1nc(no1)c1cc(ccc1)C)C1COCC1
29	F2496-3295 (Life chemicals)	16.2	n.d.	23	c1(c2cc(no2)COC(=O)c2cc3nc[nH]c3cc2)oc2c(c1)cccc2
30	5629-0125 (Chemdiv)	16	n.d.	298	c1cc(cc(c1)[N+](=O)[O-])c2ccc(o2)/C=c\3/c(=O)n4c(s3)nc(n4)c5cccs5
31	42995570 (ChemBridge)	16	n.d.	49	N1C(=O)NC(C1=O)CC(=O)NCc1sc(nc1)c1cc(OC)ccc1
32	F5449-0108 (Life chemicals)	15.8	n.d.	458	c1(cc(=O)c(co1)OC)C(=O)Nc1ccc(C(=O)OCCCC)cc1
33	11547730 (ChemBridge)	14.9	n.d.	135	c1(nc(oc1)COc1c(c(F)ccc1)F)C(=O)N(CCCO)C
34	8002-0592 (Chemdiv)	14.4	n.d.	38	c1cc(ccc1OC(=O)c2c(nsc2Cl)Cl)OC(=O)c3c(nsc3Cl)Cl
35	T6141058 (Enamine)	14.3	n.d.	50	c1cc(sc1)c2nc(on2)CSc3nnc(s3)NC4CC4
36	F1905-6556 (Life	14.1	n.d.	377	c1(cc(cnc1)CN)c1ccccc1

	chemicals)				
37	F2496-3101 (Life chemicals)	14	n.d.	42	c1(cc(no1)COC(=O)CSc1ccncc1)c1c(F)cccc1
38	F3139-1680 (Life chemicals)	13.9	n.d.	729	c1(c(cc(cc1)O)O)C(=O)COc1ccc(C(=O)OCC C)cc1
39	K822-0272 (Chemdiv)	13.8	n.d.	584	CC(=O)n1ccc2c1cc(cc2)C(=O)CSc3nnc(n3C)c4cccnc4
40	0782-7025 (Chemdiv)	12.8	n.d.	694	CCCCCCCOc1ccc(cc1)C2=N/C(=C/c3ccco3)/C(=O)O2
41	6420-3607 (Chemdiv)	11.9	n.d.	120	c1ccc2c(c1)ncc(n2)c3ccc(cc3)OCC(=O)c4ccc s4
42	F6514-4048 (Life chemicals)	10.5	n.d.	69	n1(nnc(c1)COc1ccccc1)C1CN(C(=O)Cc2cnc cc2)C1
43	F2496-0660 (Life chemicals)	10.1	n.d.	63	c1(cc(no1)COC(=O)CC1CCCCC1)c1ccc(cc1)OC
44	F6064-2993 (Life chemicals)	9.8	n.d.	52	n1c(C2CN(C(=O)NCCOC)C2)onc1c1cc(Cl)c cc1
45	F6064-7687 (Life chemicals)	9.3	n.d.	62	n1c(c2c(=O)[nH]ccc2)noc1C1CN(C(=O)Cc2 noc3c2cccc3)C1
46	F0682-1220 (Life chemicals)	9	n.d.	267	n1(nnc2c1ncnc2SCC(=O)OCc1ccccc1)c1ccc(cc1)OC
47	F3382-3571 (Life chemicals)	8.9	n.d.	374	c12n(nc(c1)c1c(OC)cccc1)ccn(c2=O)Cc1nc(no1)c1ncccc1
48	AJ-292/419 45407(Spec s)	7.7	n.d.	6	CCC(=O)Nc1ccc(cc1)C(=O)OCC(=O)c2ccc(c(c2)Cl)Cl
49	AJ-292/415 48204(Spec s)	7	n.d.	209	CC(=O)Nc1ccccc1C(=O)OCC(=O)c2ccc(cc2)OC(=O)c3ccccc3
50	AK-918/42 180074(Spe cs)	5.7	n.d.	37	CCOc1ccc(cc1)C(=O)COC(=O)c2ccc(cc2)N C(=O)C
51	AN-652/43 024838(Spe cs)	5.6	n.d.	521	CCCCOC(=O)c1ccc(cc1)OC(=O)c2cccc3c2c ccc3
52	AK-918/41 968204(Spe cs)	5.4	n.d.	308	c1cc(oc1)C(=O)Oc2ccc(cc2)C(=O)COC(=O) c3ccc(cc3Cl)Cl

53	F2496-3037 (Life chemicals)	3.6	n.d.	306	c1(c2c(cc(cc2)F)F)cc(no1)COC(=O)c1cc2nc[nH]c2cc1
54	T6363271 (Enamine)	2.9	n.d.	74	Cc1ccc(cc1)c2nc(no2)CSc3nnc(o3)c4ccco4

[a]n.d. means not determined.

Table S2. Binding affinities of commercially available analogs of SPC-3 and SPC-5.

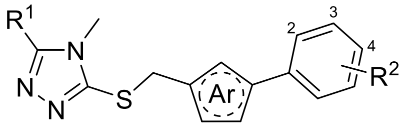

Name	R^1	Ar	R^2	K_D (μM)
SPC-21	H	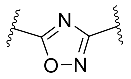	3-CF_3	1.7
SPC-22	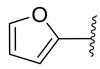	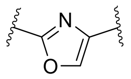	H	333
SPC-23	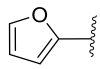	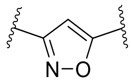	H	2.0
SPC-24	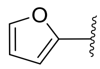	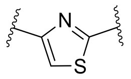	H	32.3
SPC-25	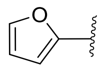	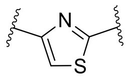	3-CH_3	25.3
SPC-26	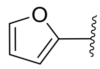	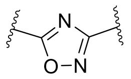	H	164
SPC-27	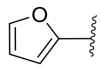	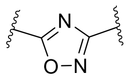	3-Cl	13.4
SPC-28	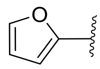	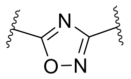	4-Cl	26.6
SPC-29	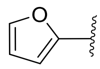	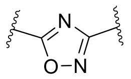	4-CH_3	69.3
SPC-30	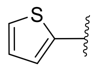	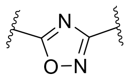	4-Cl	10.7
SPC-31	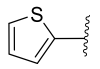	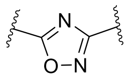	4-CH_3	72.7

SPC-32	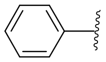	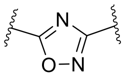	4-Cl	8.1
SPC-5	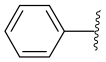	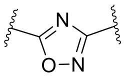	4-OCH_3	31.4
SPC-33	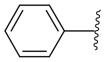	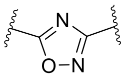	2-CH_3	21.1
SPC-3	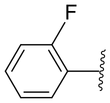	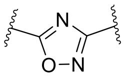	2-Cl	19.8
SPC-34	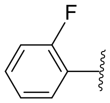	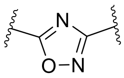	4-CH_2CH_3	74.6
SPC-35	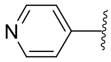	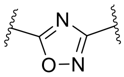	4-F	32.0
SPC-36	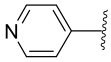	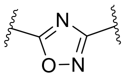	4-CH_3	54.0
SPC-37	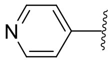	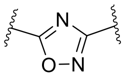	4-OCH_3	7.1
SPC-38	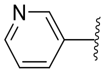	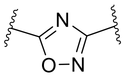	4-F	111

Table S3. Binding affinities of commercially available analogs of SPC-6.

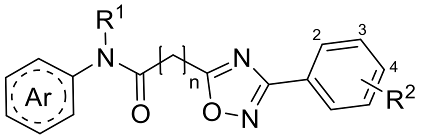

Name	Ar	R^1	n	R^2	K_D (μM)
SPC-6	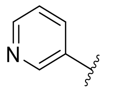	H	2	3-OMe	54.1
SPC-39	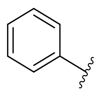	H	2	3-OMe	24.6
SPC-40	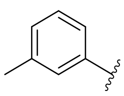	H	2	3-OMe	37.9
SPC-41	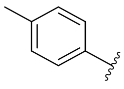	H	2	3-OMe	37.9
SPC-42	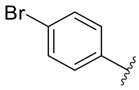	$-CH_3$	2	4-OMe	22.7
SPC-43	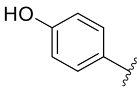	H	3	H	9.8

Table S4. Aggregation evaluation of representative compounds using dynamic light scattering (DLS).

Structure	DLS, no centrifugation[b]	DLS, with centrifugation[c]
**PBS-P running buffer[a]**	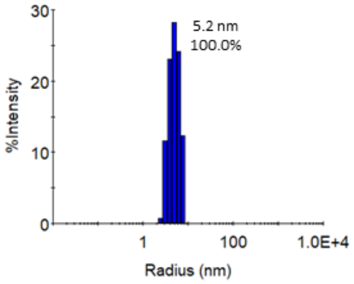	
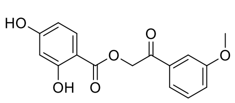<br>SPC-1	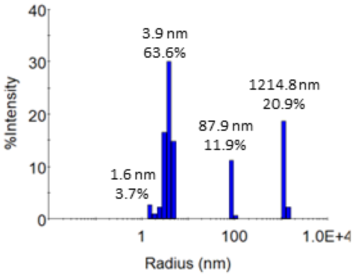	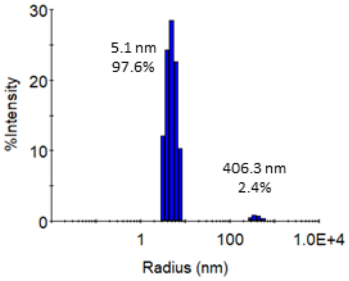
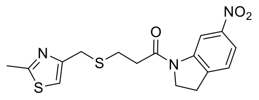<br>SPC-2	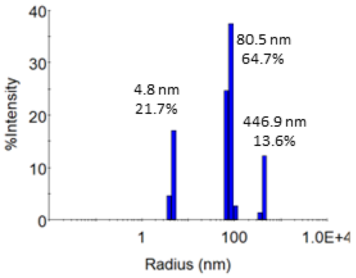	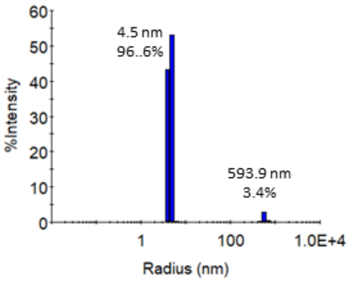
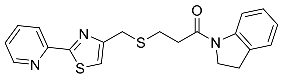<br>SPC-13	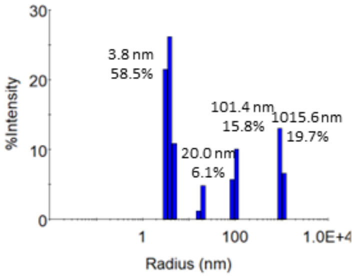	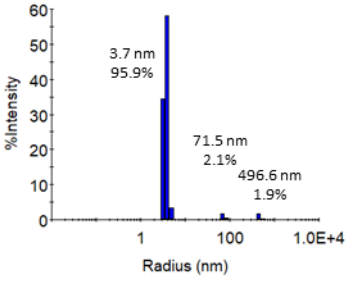
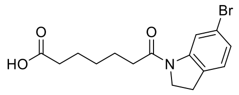<br>SPC-15	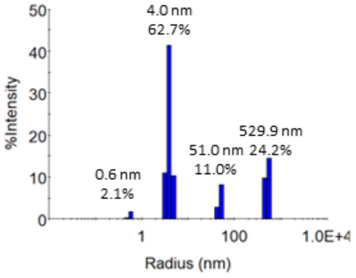	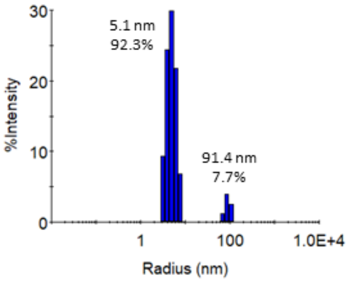

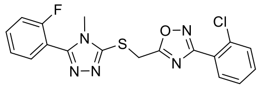

SPC-3

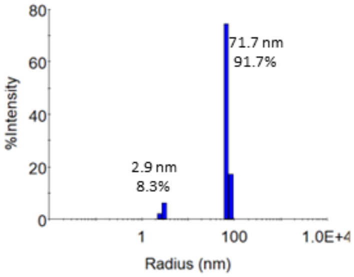

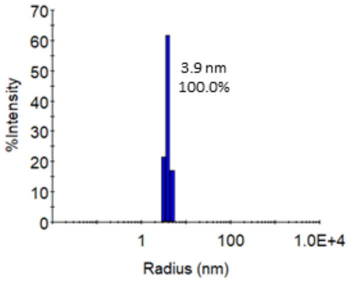

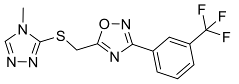

SPC-21

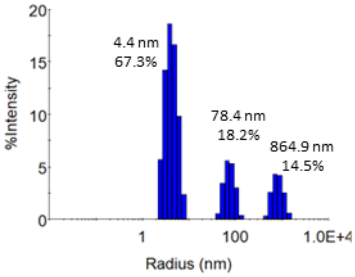

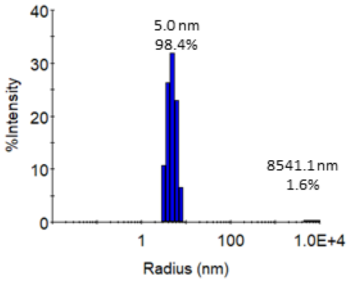

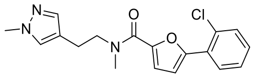

SPC-4

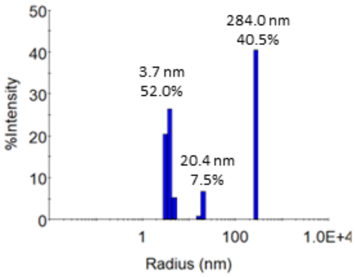

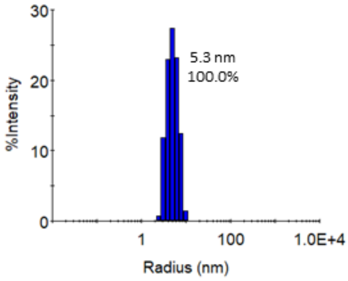

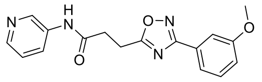

SPC-6

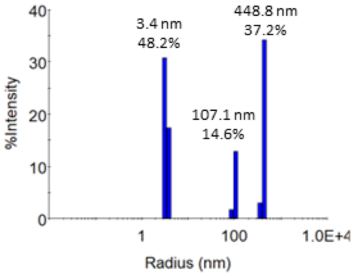

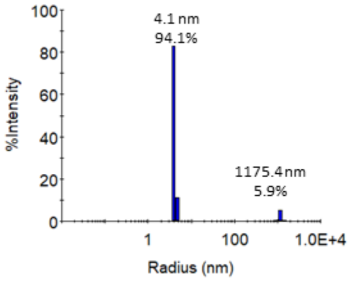

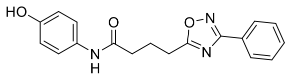

SPC-43

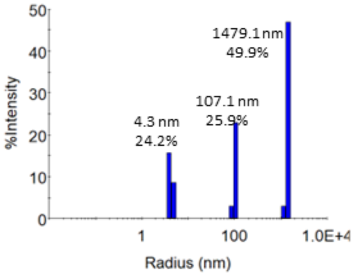

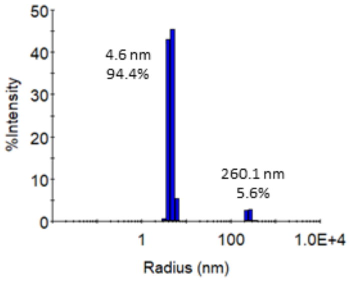

[a]PBS-P running buffer containing 20 mM PBS (pH 7.4), 2.7 mM KCl, 137 mM NaCl, 0.05% surfactant P20, and 5% DMSO was used as the reference sample, with an average radius of 5.2 nm.

[b]DLS intensity profile of compound at 25 µM in PBS-P buffer with 5% DMSO.

[c]After centrifuging the sample (compound at 25 µM in PBS-P buffer with 5% DMSO), particle radius distribution of the supernatant was measured by DLS.

Table S5. Cryo-EM data collection, refinement and validation statistics.

	Map of SARS-CoV-2 spike protein and SPC-14 complex	Map of apo SARS-CoV-2 spike protein
**Data collection and processing**		
Magnification	96k	96k
Voltage (kV)	300	300
Electron exposure ($e^{-}/Å^2$)	50	50
Defocus range (μm)	-1.0~-2.0	-1.0~-2.0
Pixel size (Å)	0.86	0.86
Symmetry imposed	C3	C1
Initial particle images (no.)	127,775	68,452
Final particle images (no.)	59,187	34,118
Map1 resolution (Å)	3.06	3.27
FSC threshold	0.143	0.143
Map1 resolution range (Å)	2.6-8	2.8-8
**Refinement**		
Initial model used (PDB code)	AF2 predicted	AF2 predicted
Model resolution (Å)	3.06	3.27
FSC threshold	0.143	0.143
Model resolution range (Å)	2.6-8	2.8-8
Map sharpening B factor ($Å^2$)	109.0	84.1
Model composition		
Protein residues	3159	3103
R.m.s. deviations		
Bond lengths (Å)	0.003	0.006
Bond angles (°)	0.455	0.631
Validation		
Clashscore	9.97	4.99
Poor rotamers (%)	0.11	0.22
Ramachandran plot		
Favored (%)	94.55	93.57
Allowed (%)	5.45	6.43
Disallowed (%)	0.00	0.00

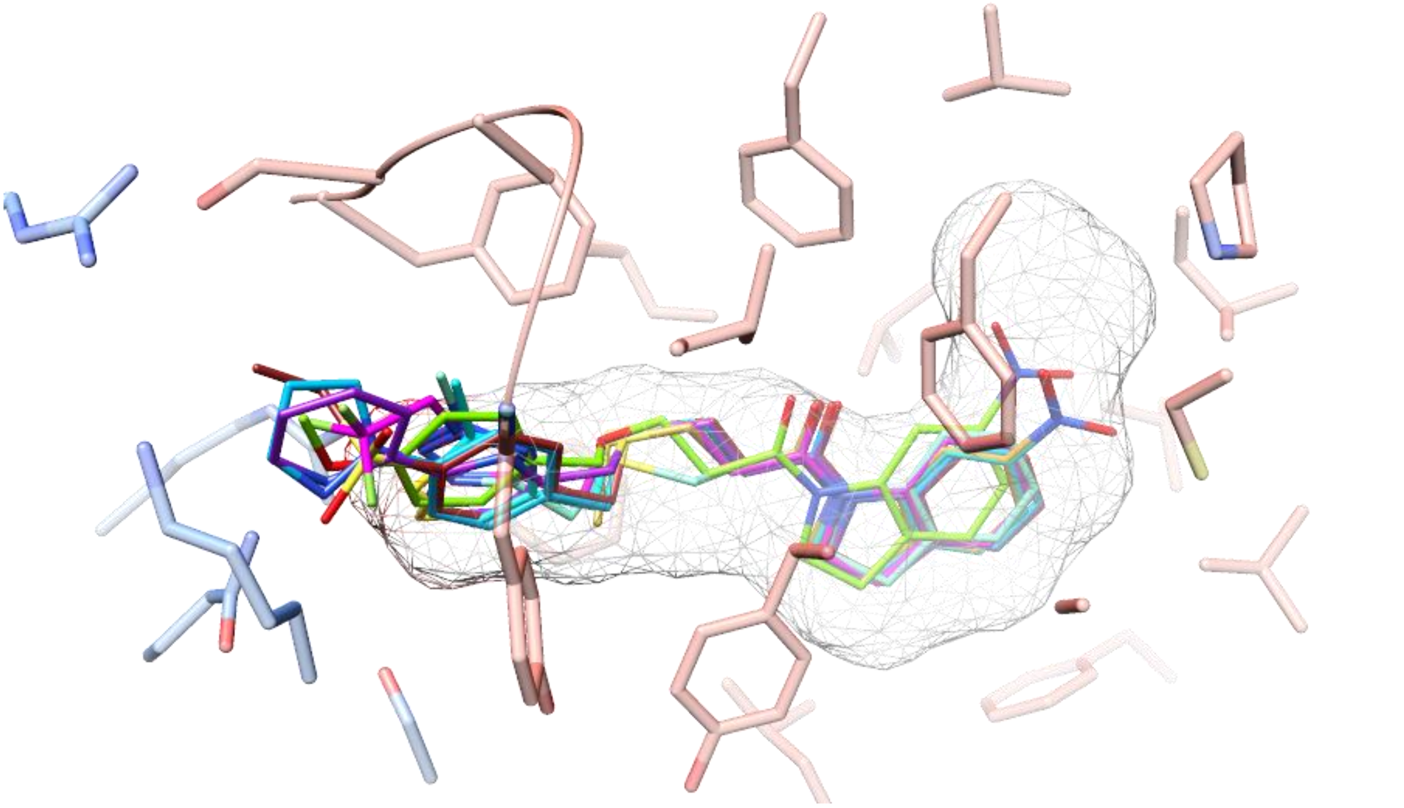

Figure S1. Docking poses of commercially available analogs of SPC-2 against the FFA-binding pocket of SARS-CoV-2 spike protein.

Concentration response curves and steady-state binding curves in SPR assay. For clear representations, spikes at the beginning and end of an injection were removed and refitted.

Reference compounds:

LA

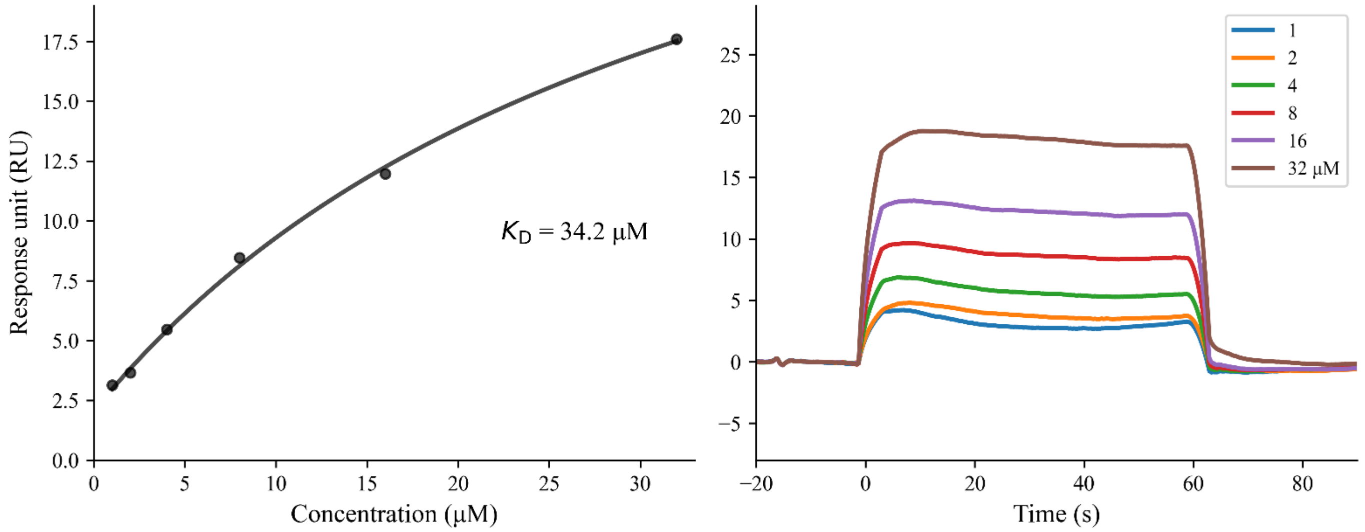

OA

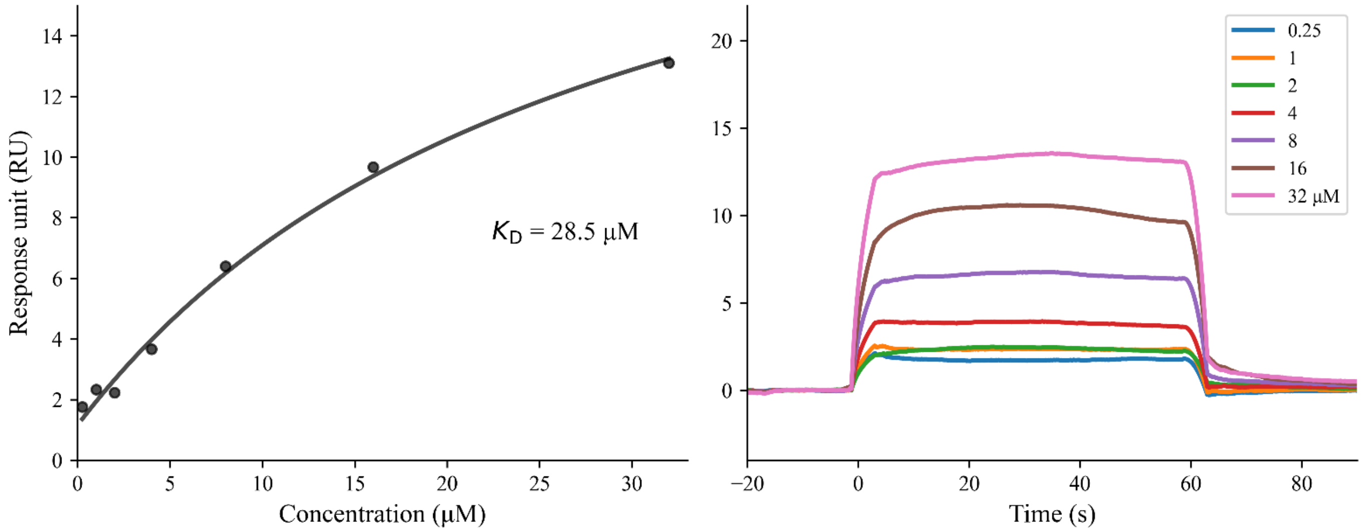

ATRA

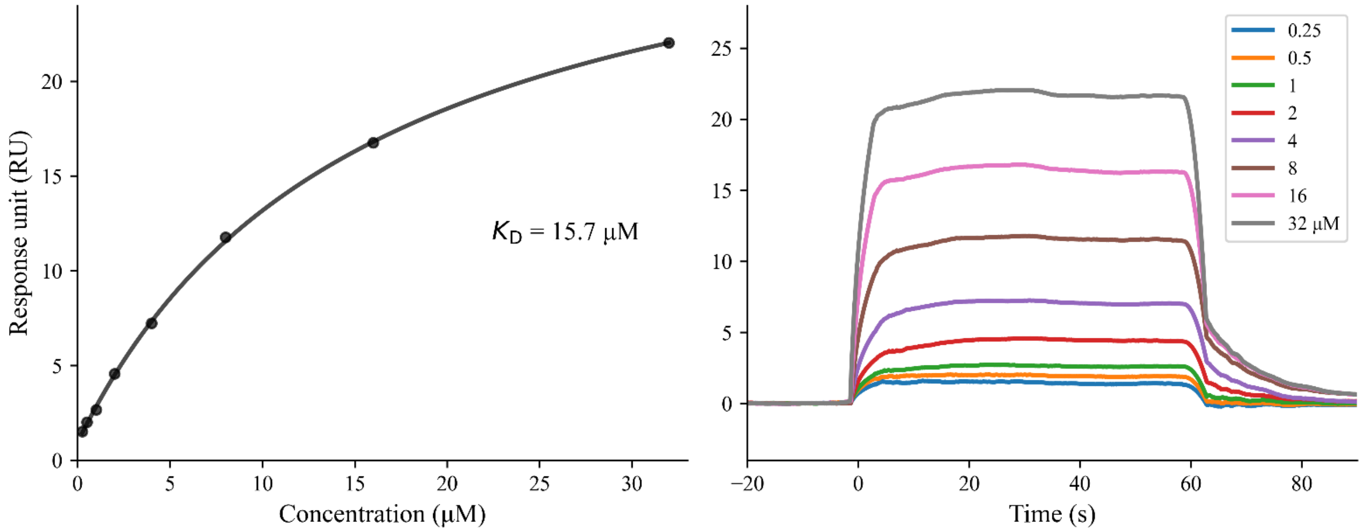

Commercially available compounds:

SPC-1

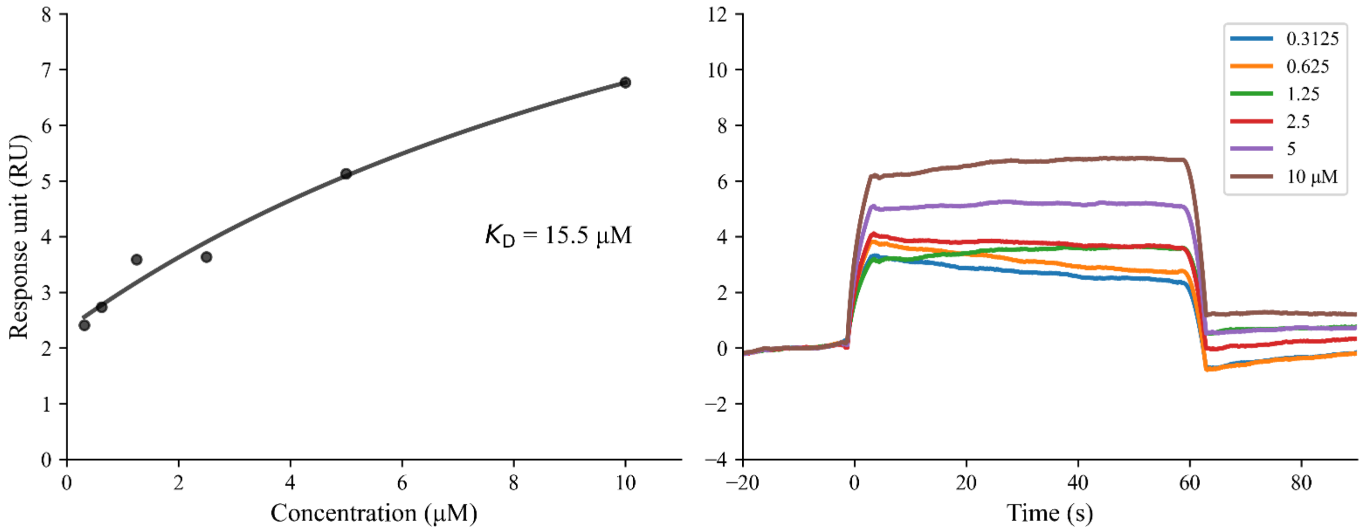

SPC-2

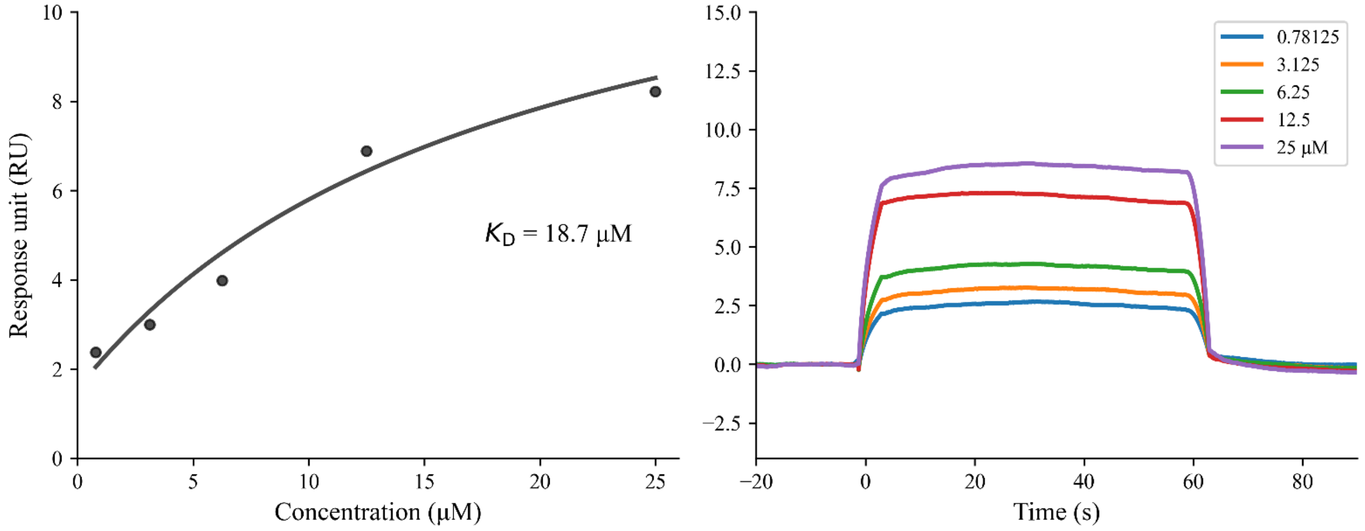

SPC-3

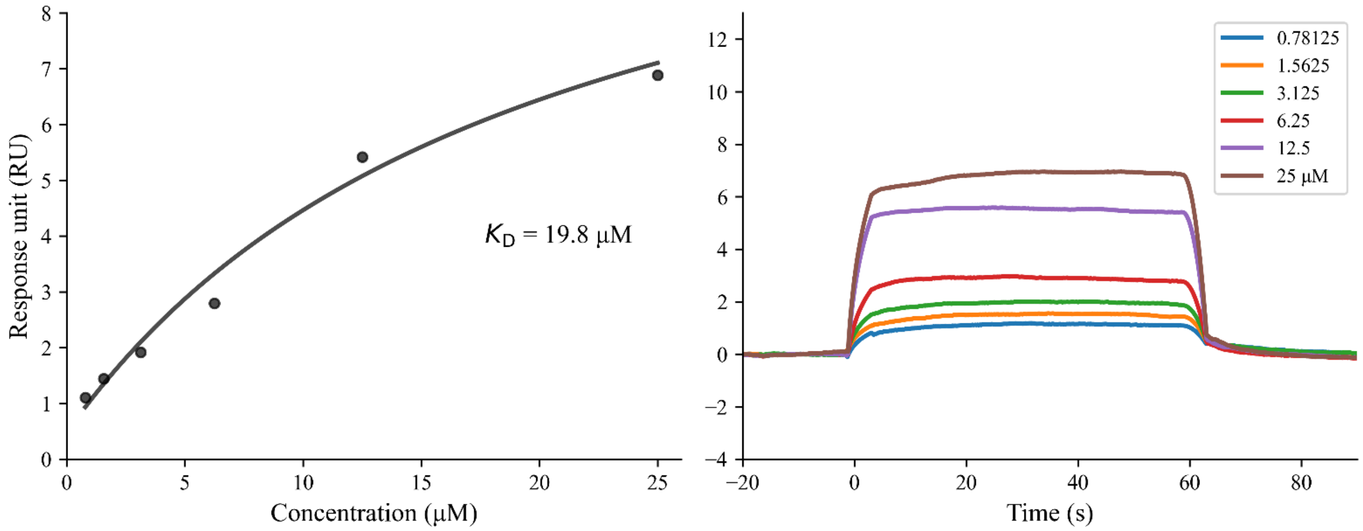

SPC-4

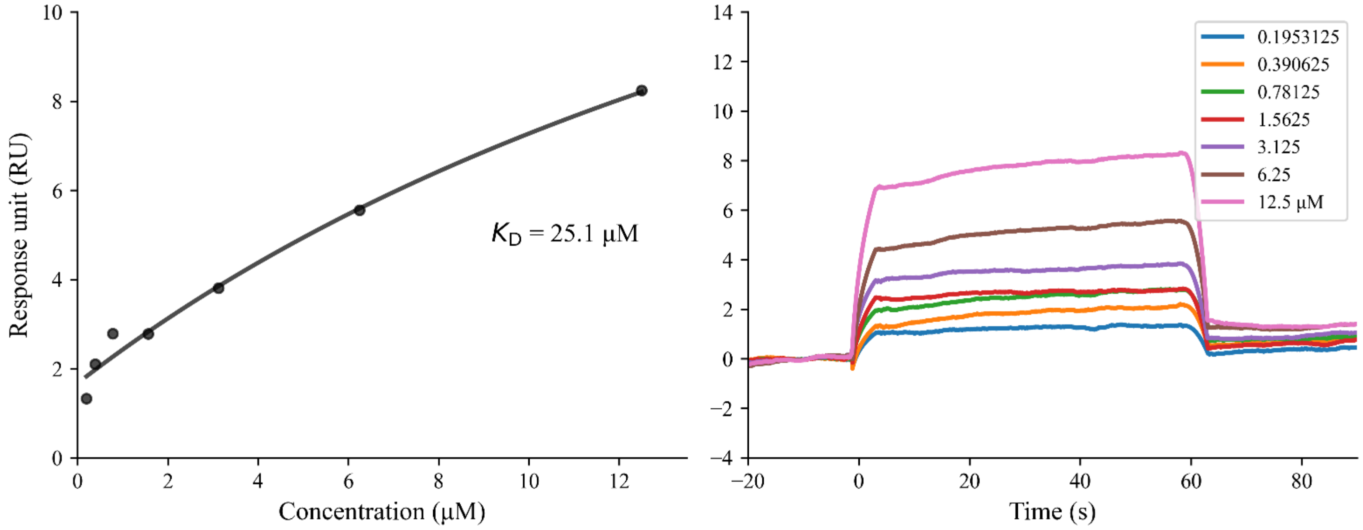

SPC-5

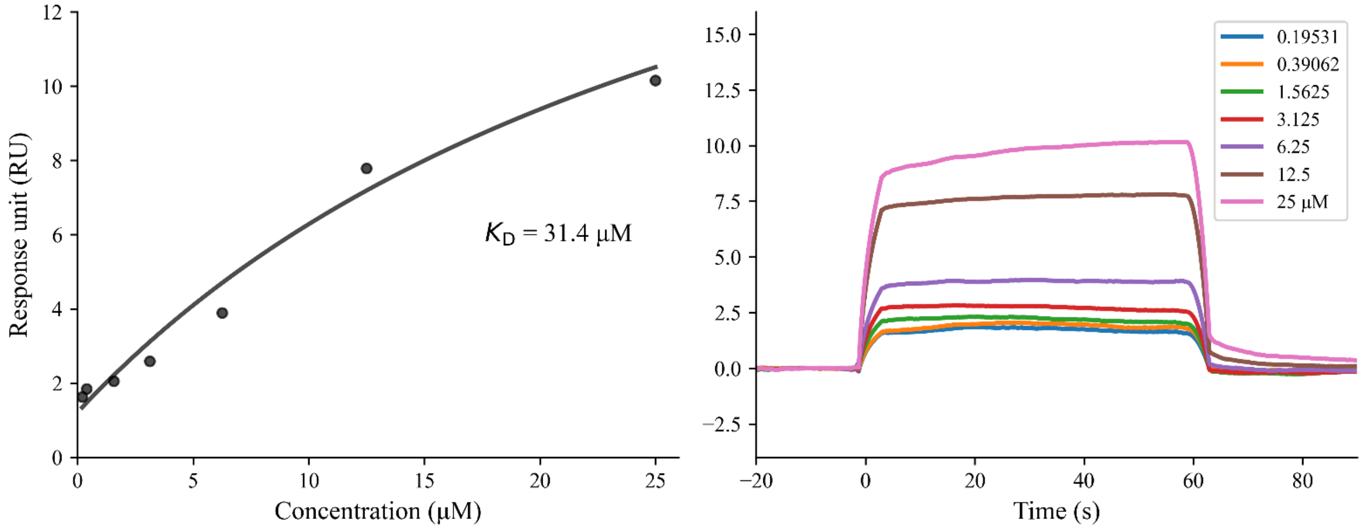

SPC-6

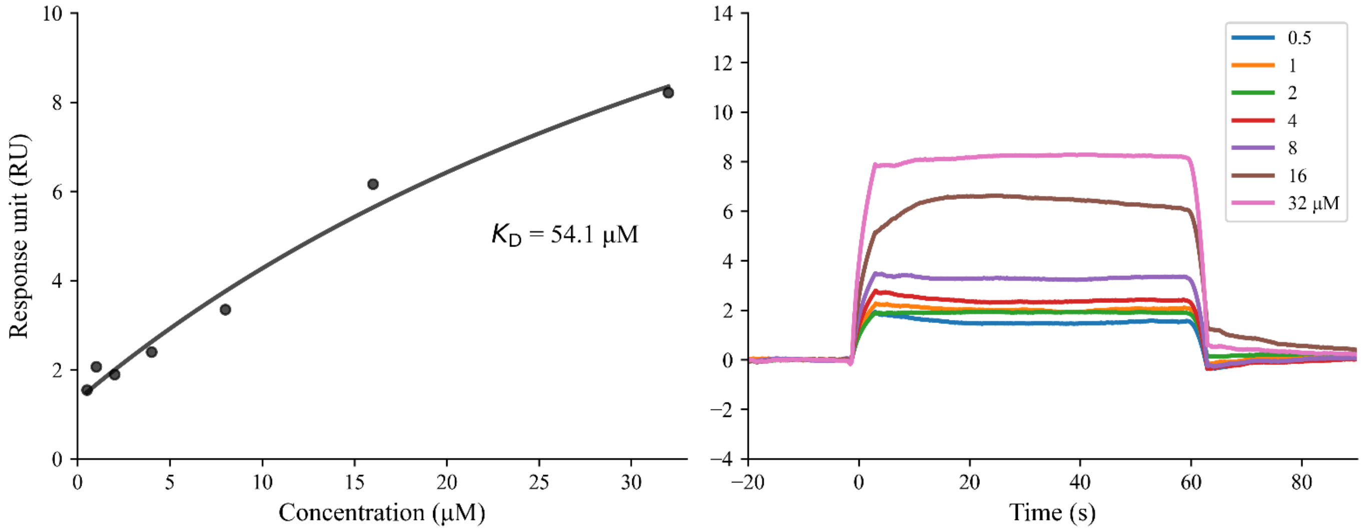

SPC-7

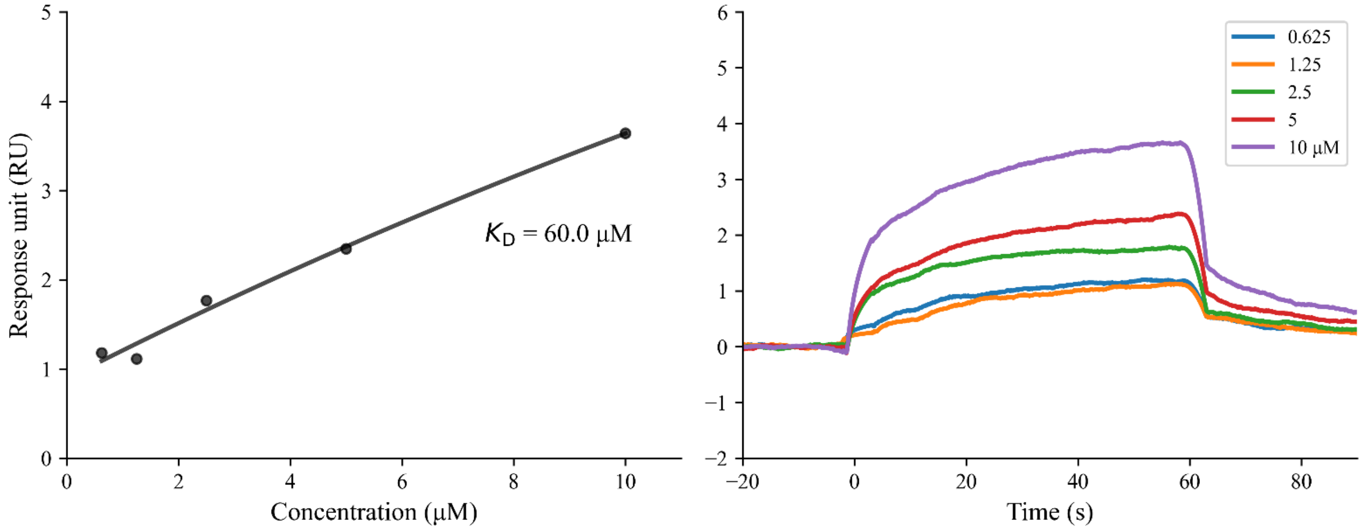

SPC-8

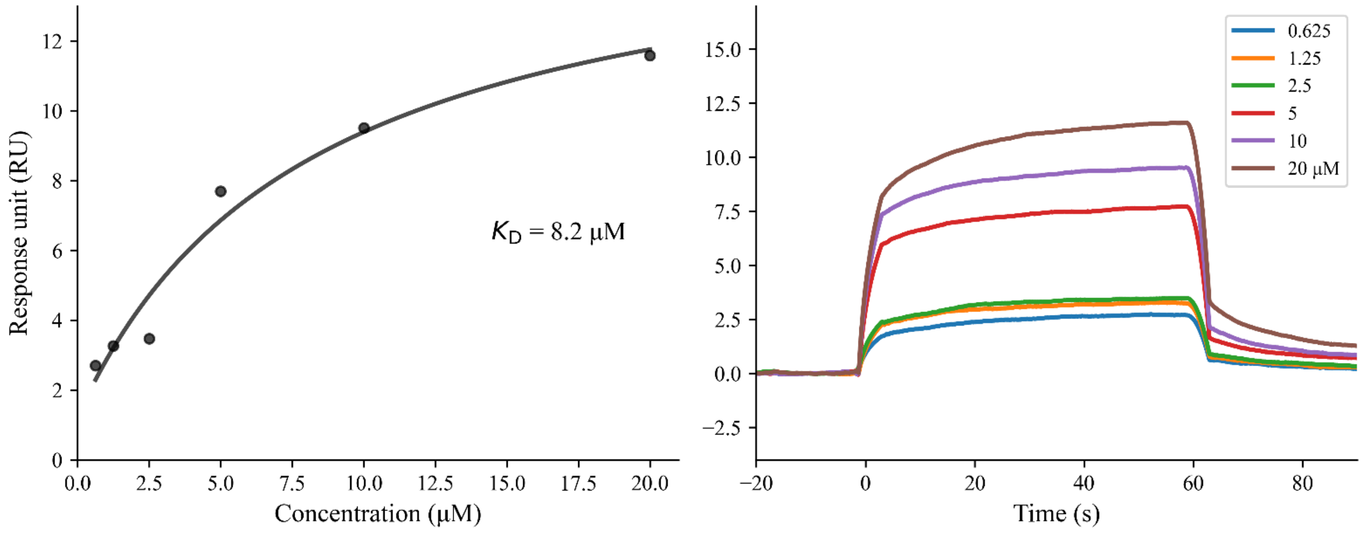

SPC-9

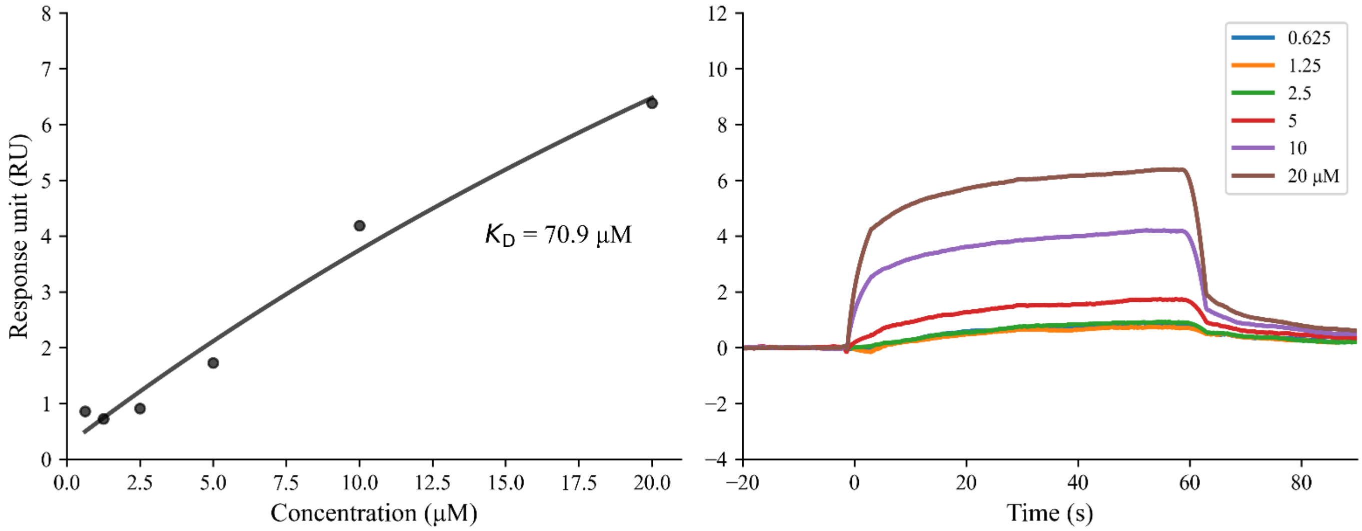

SPC-10

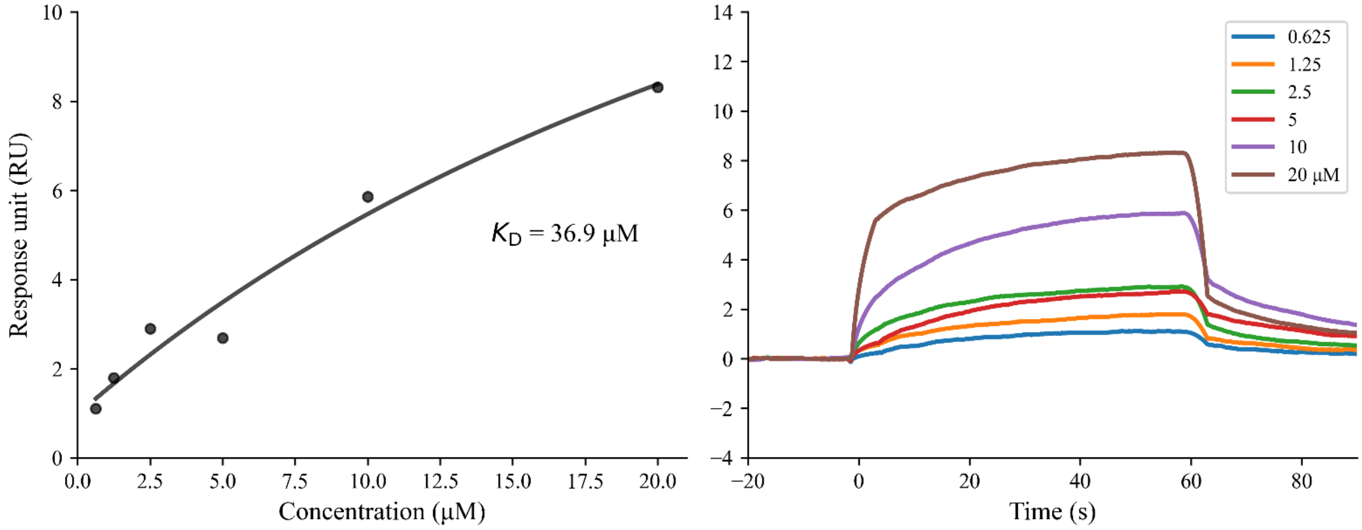

SPC-11

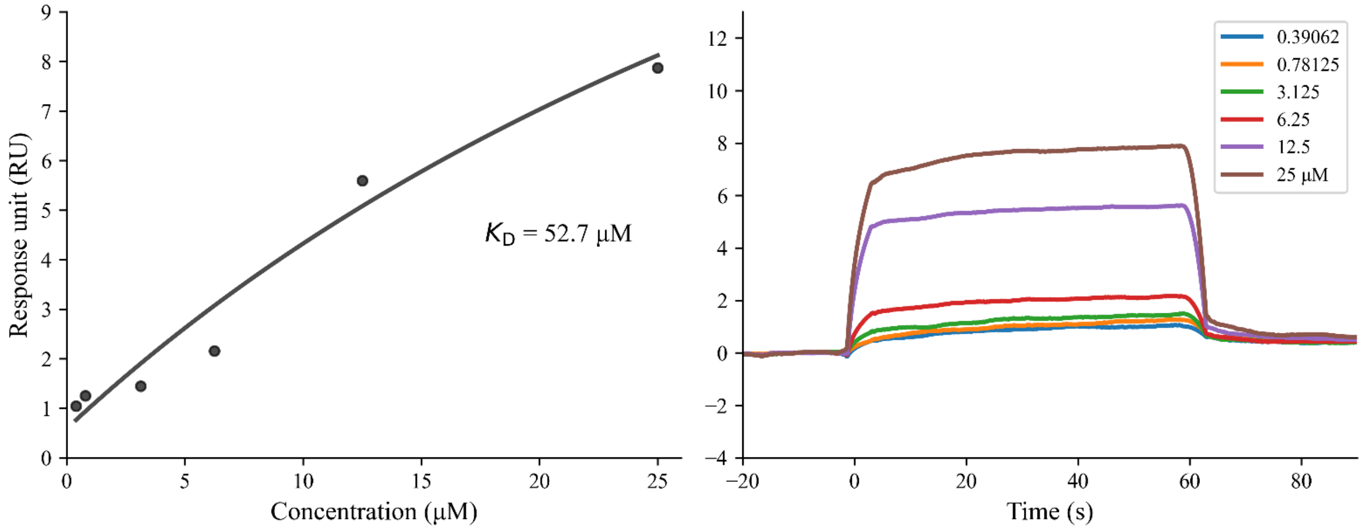

SPC-12

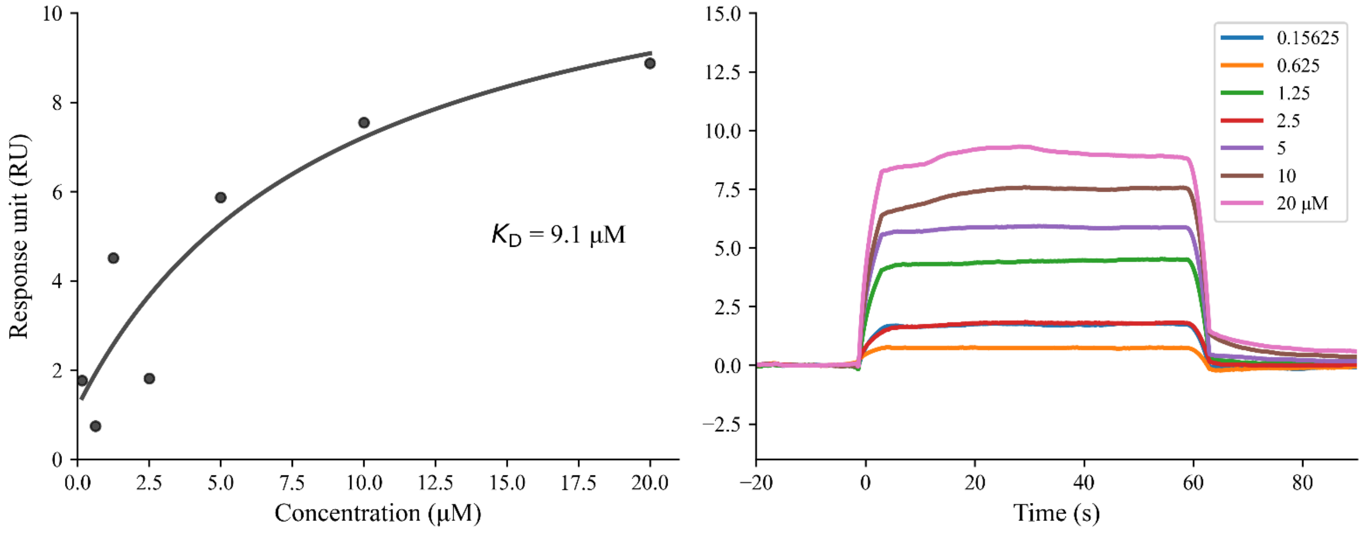

SPC-13

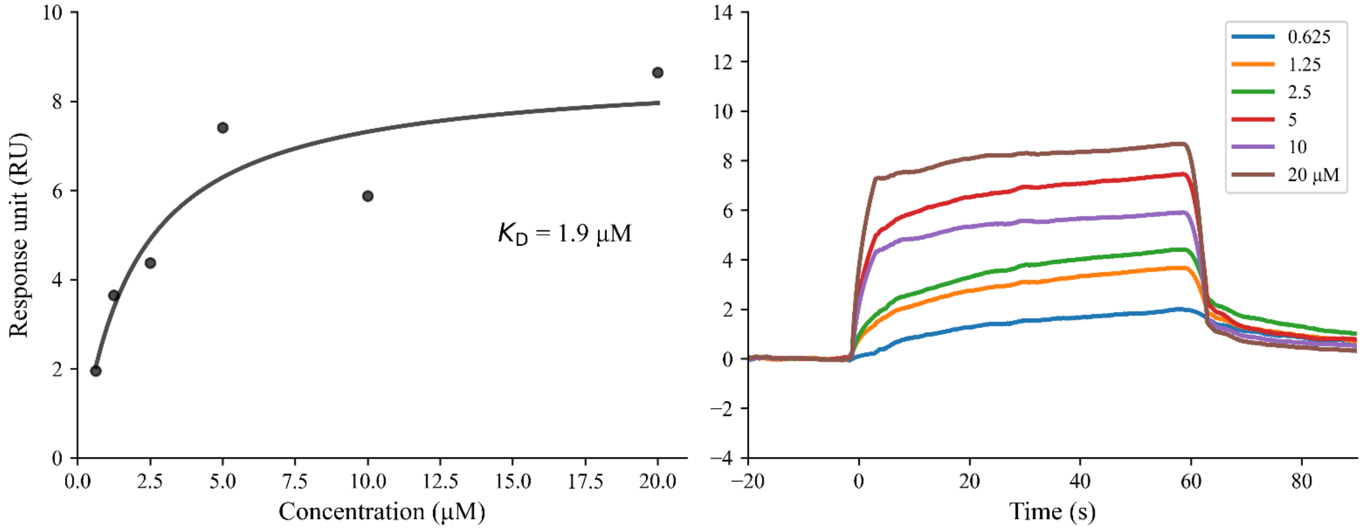

SPC-21

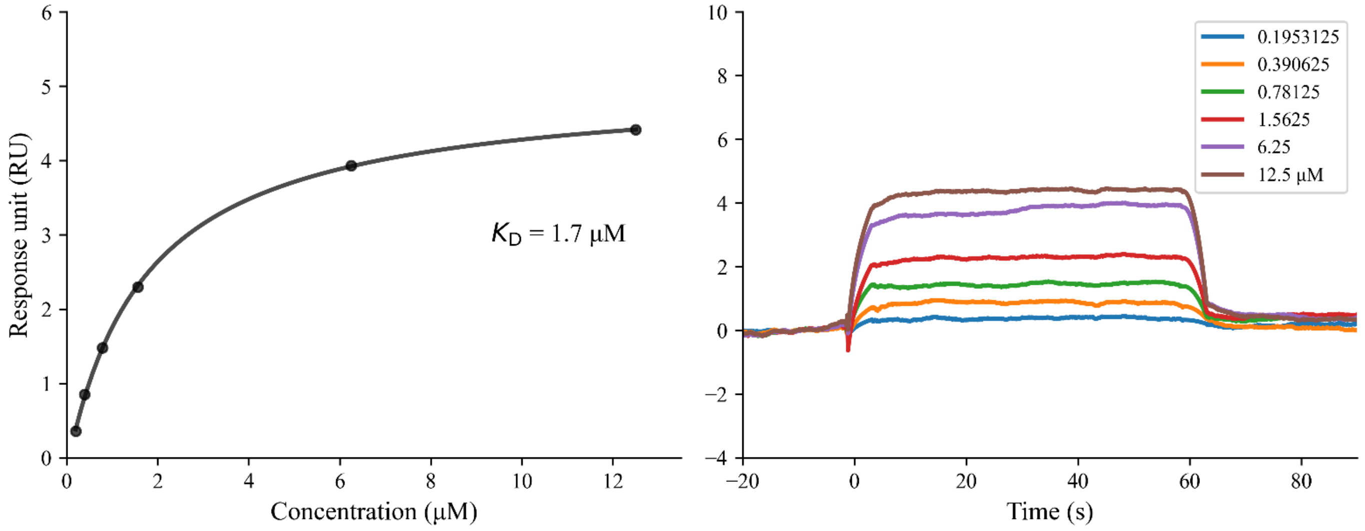

SPC-22

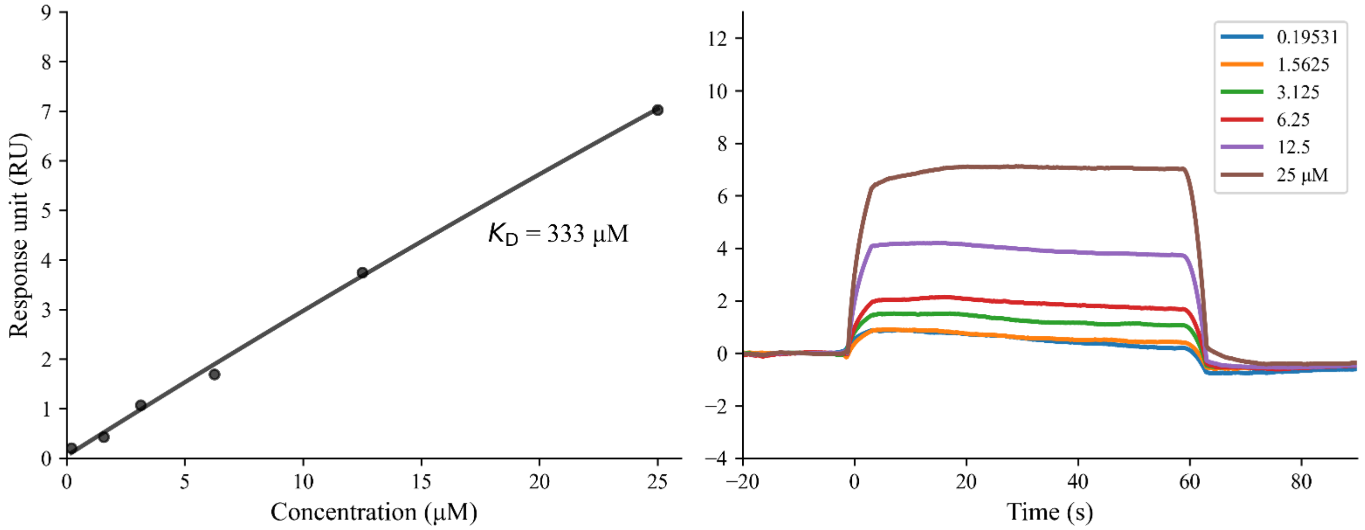

SPC-23

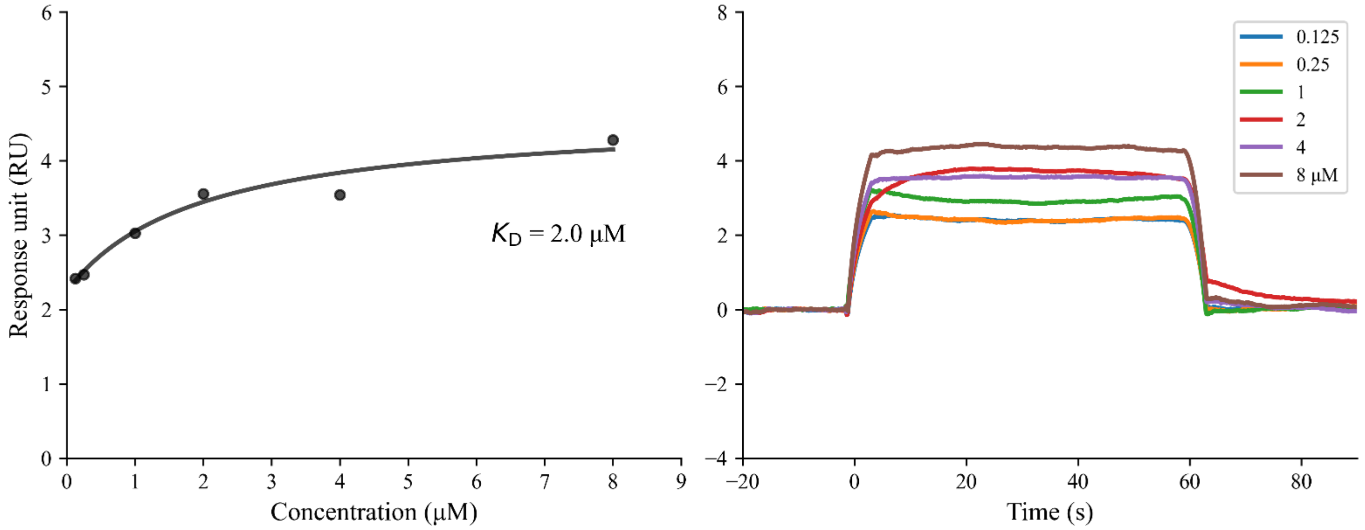

SPC-24

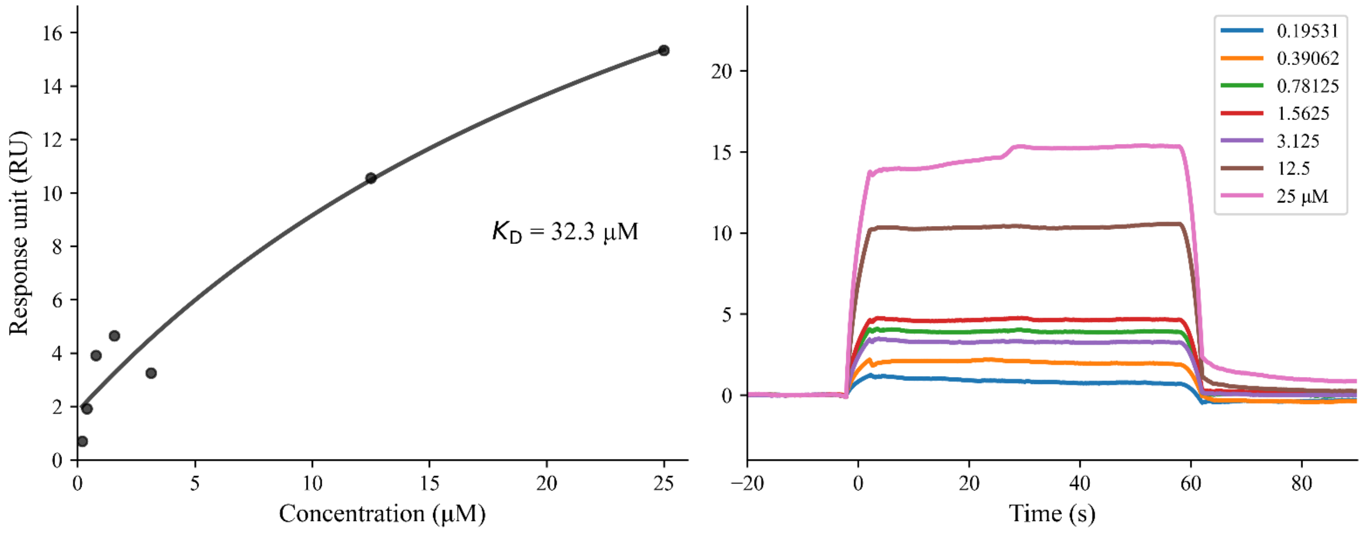

SPC-25

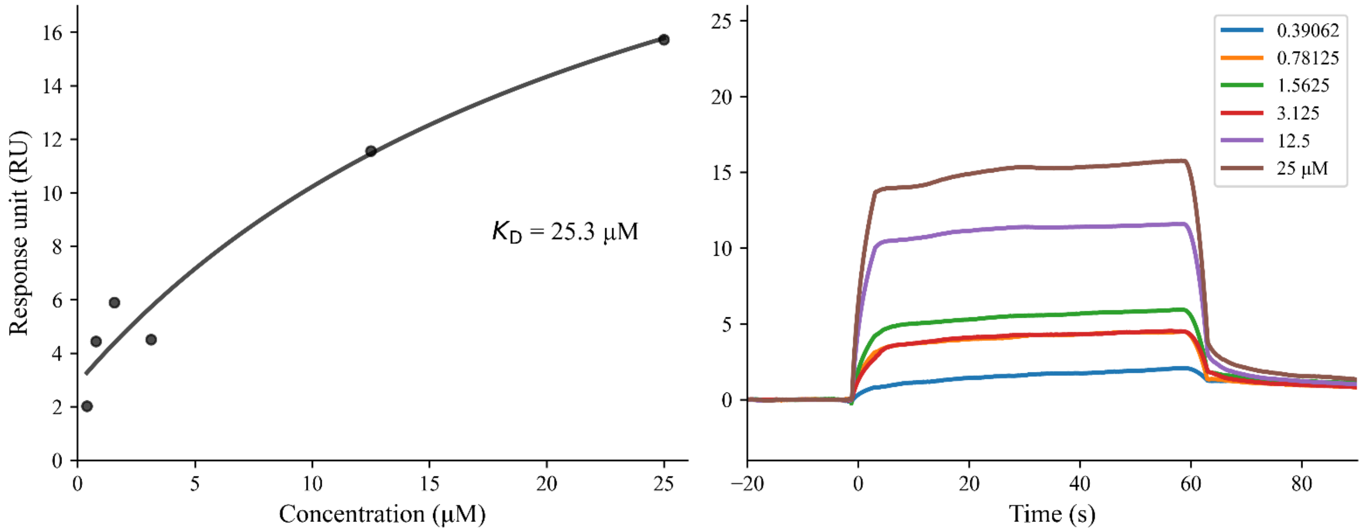

SPC-26

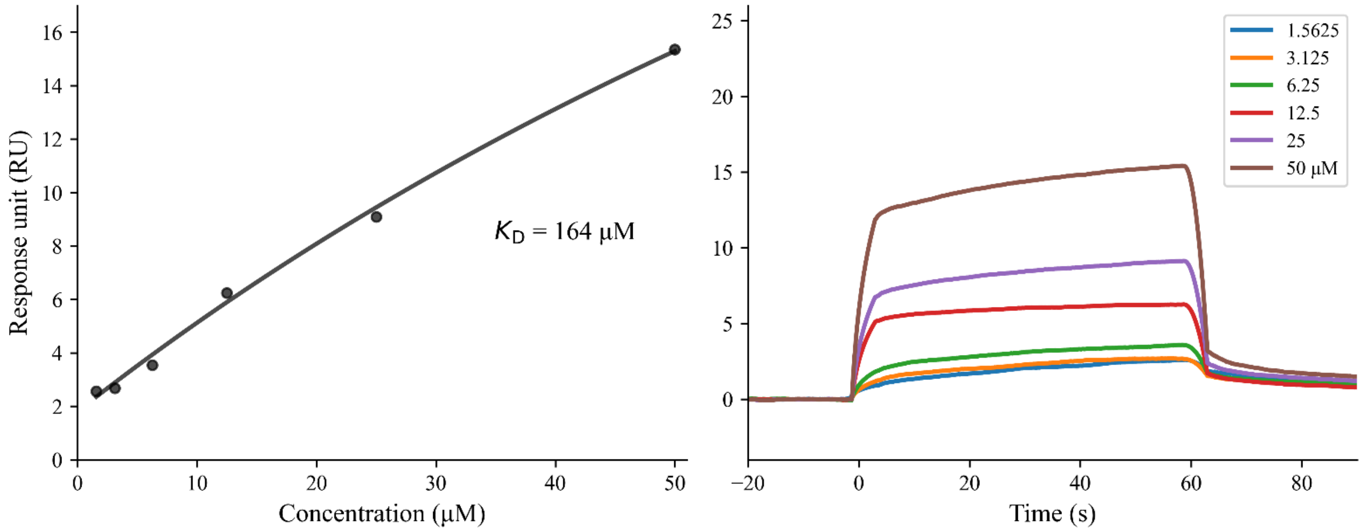

SPC-27

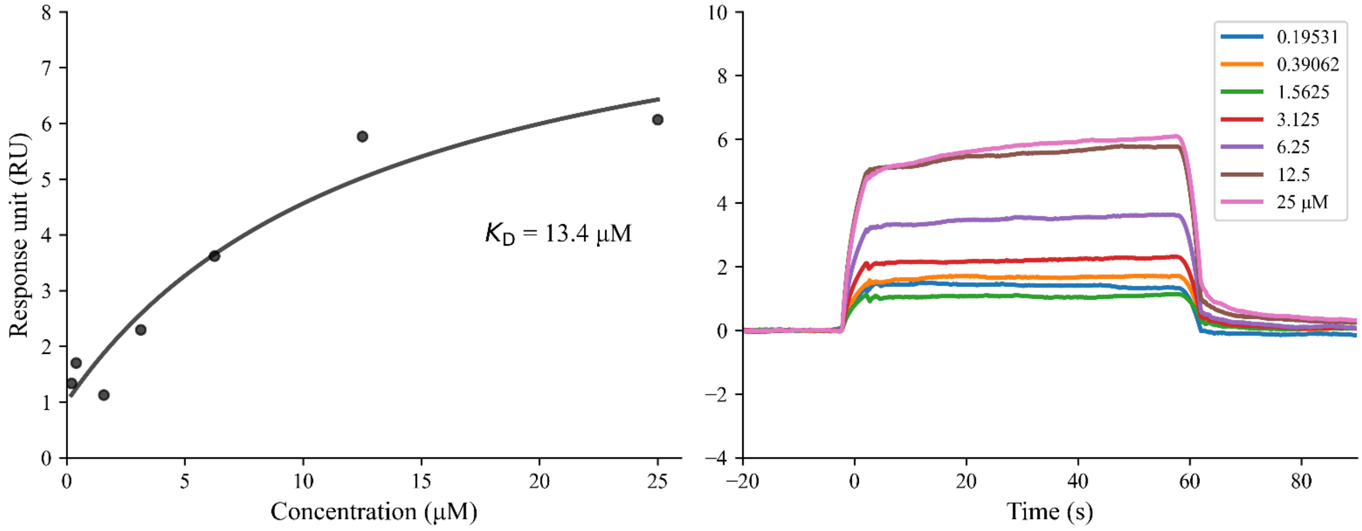

SPC-28

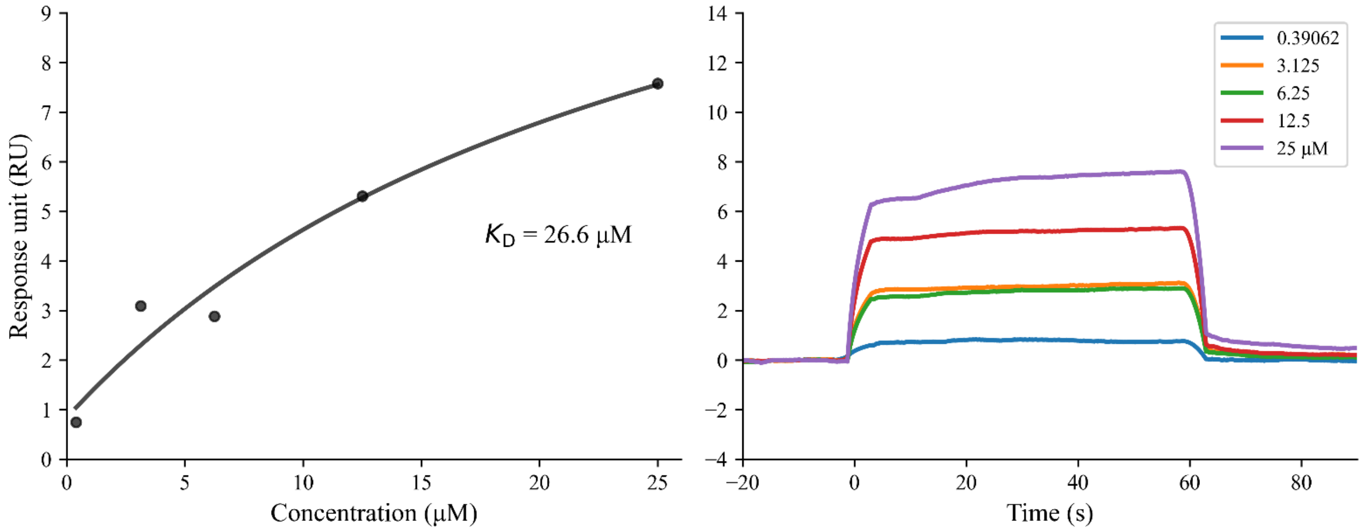

SPC-29

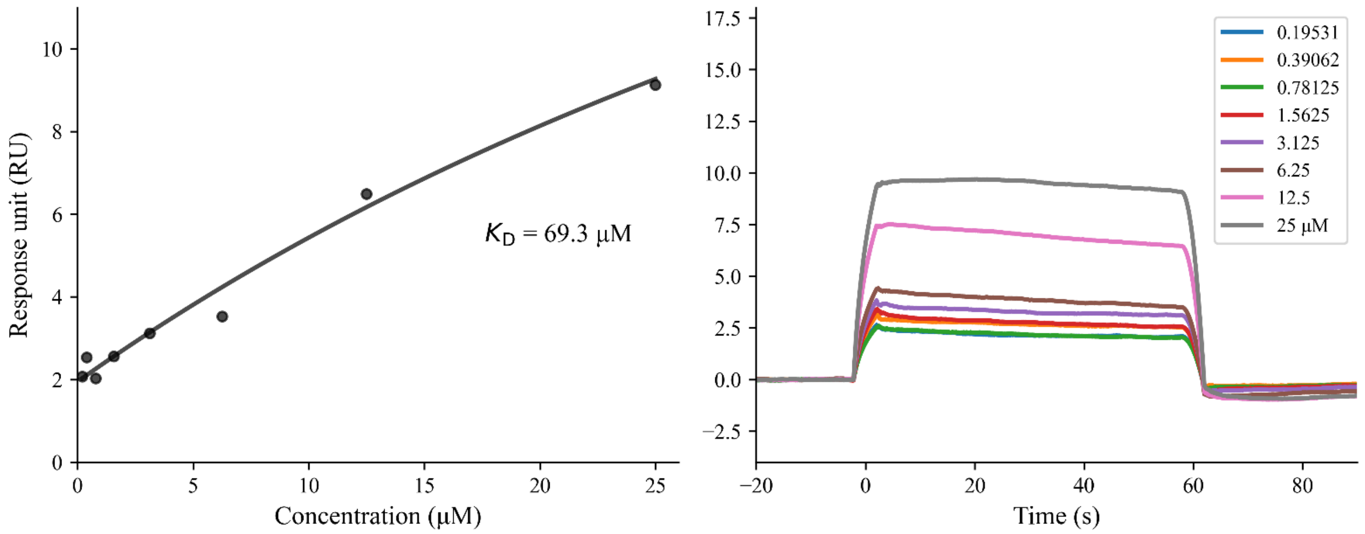

SPC-30

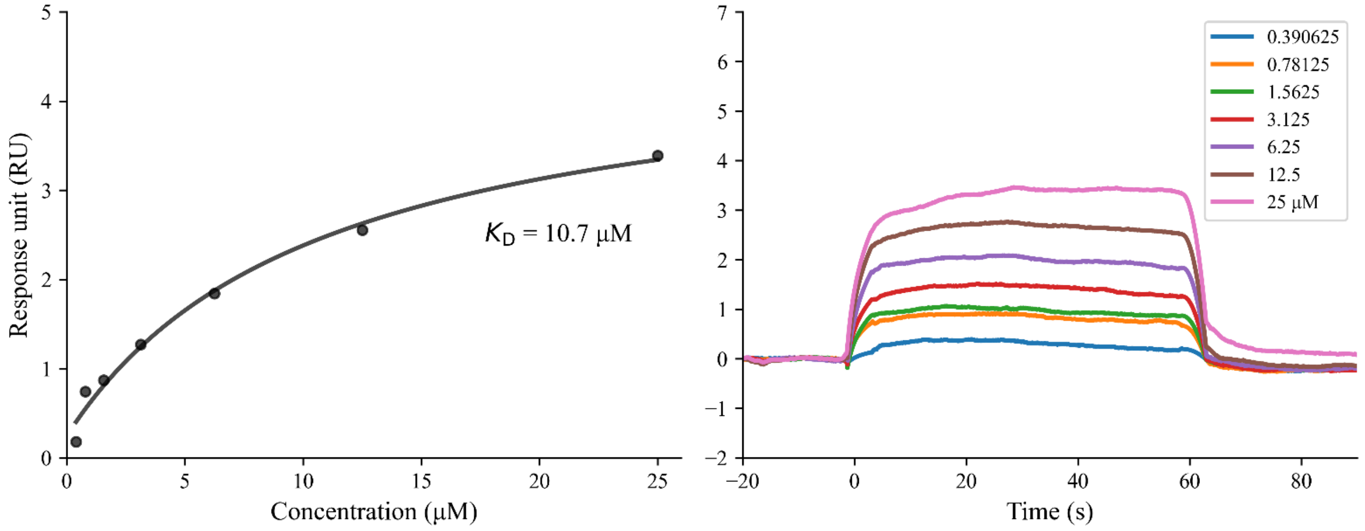

SPC-31

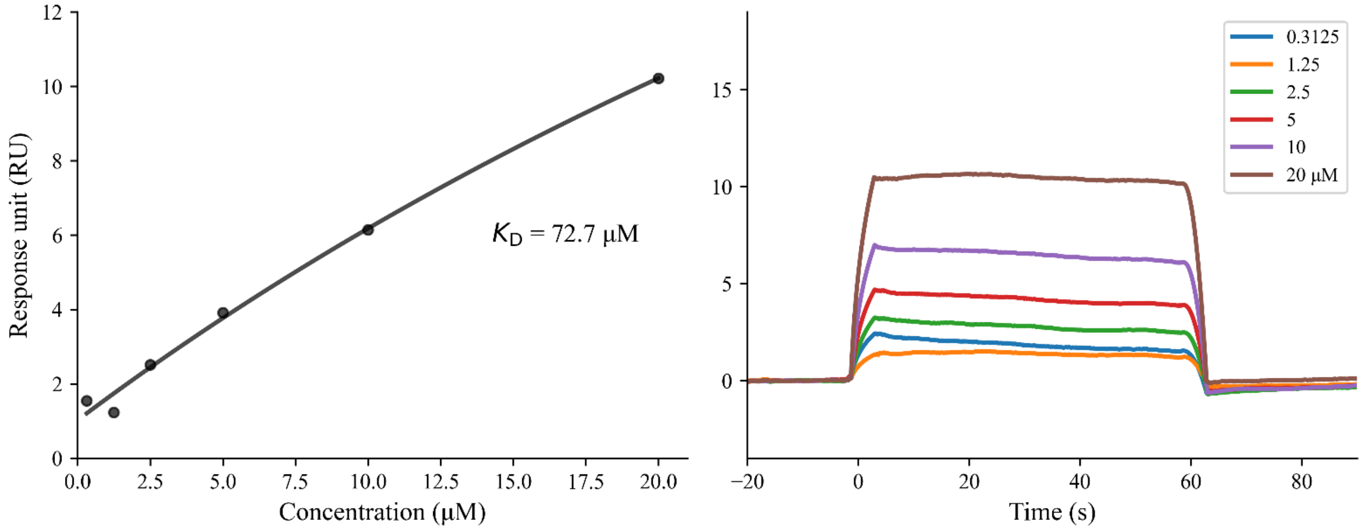

SPC-32

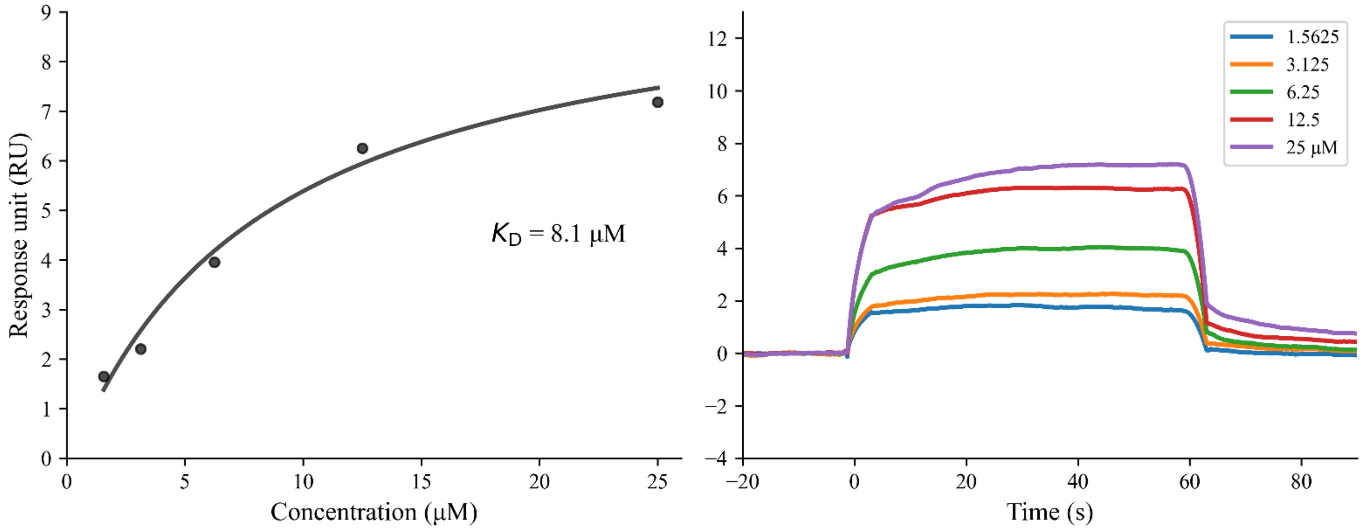

SPC-33

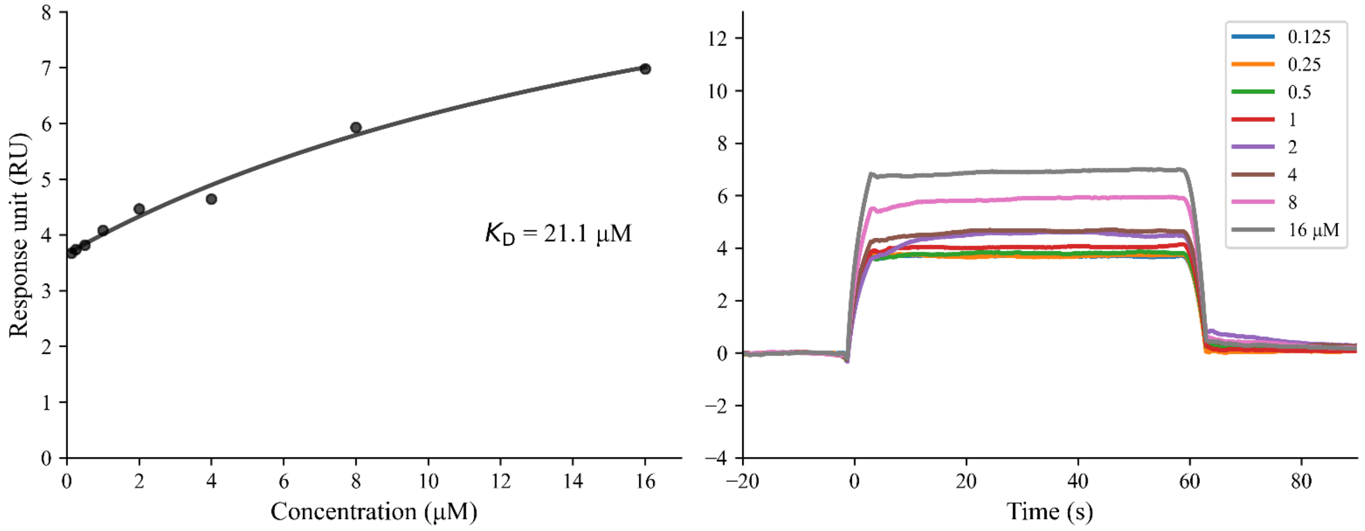

SPC-34

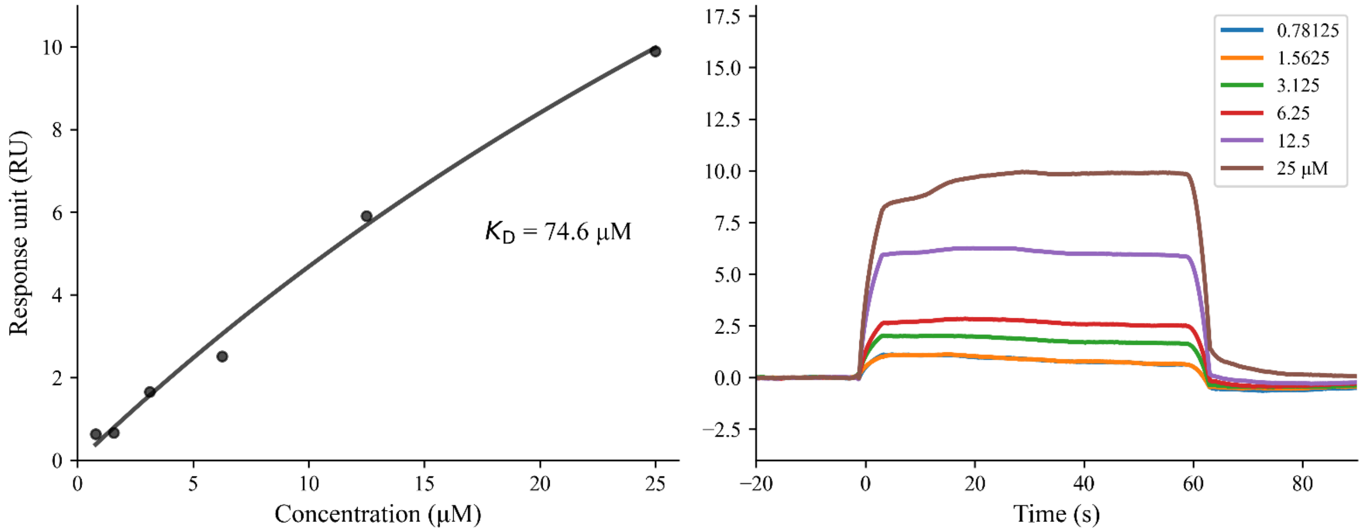

SPC-35

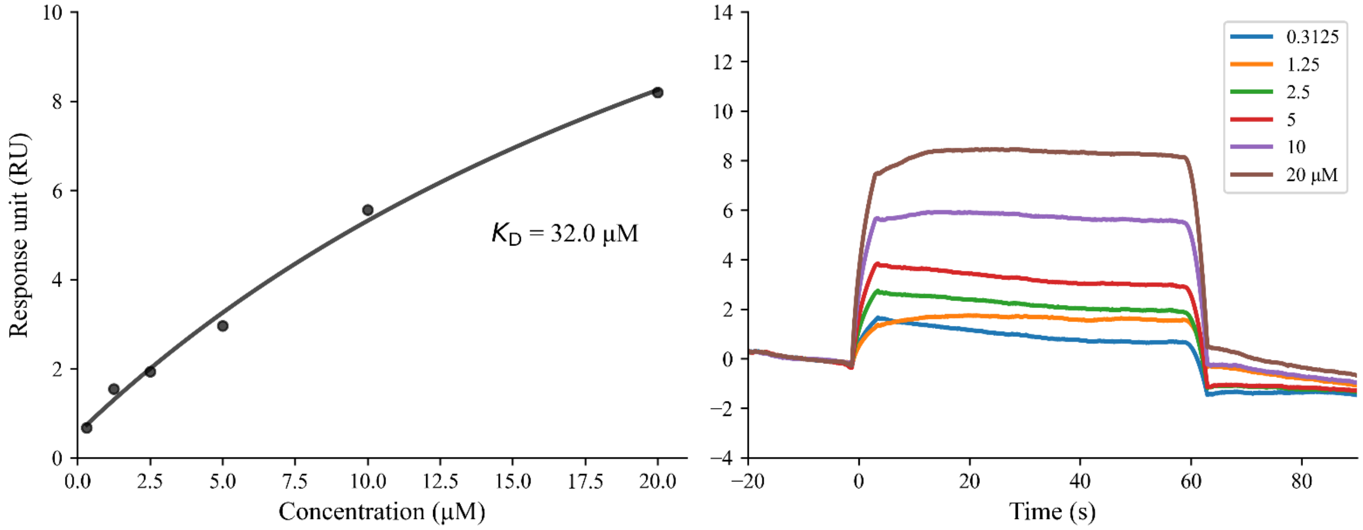

SPC-36

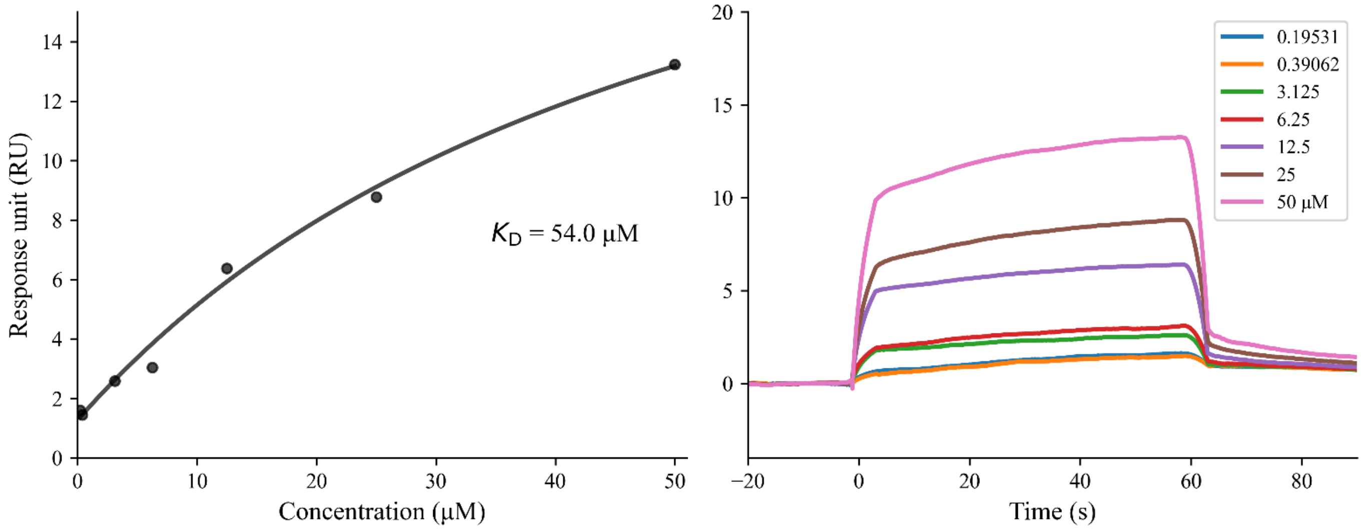

SPC-37

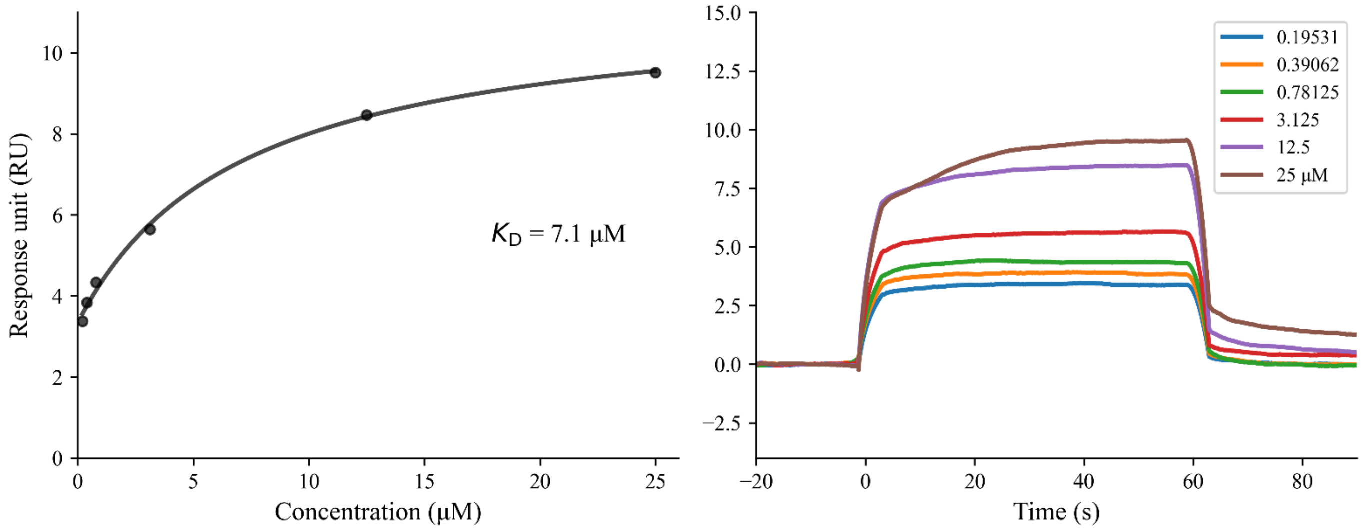

SPC-38

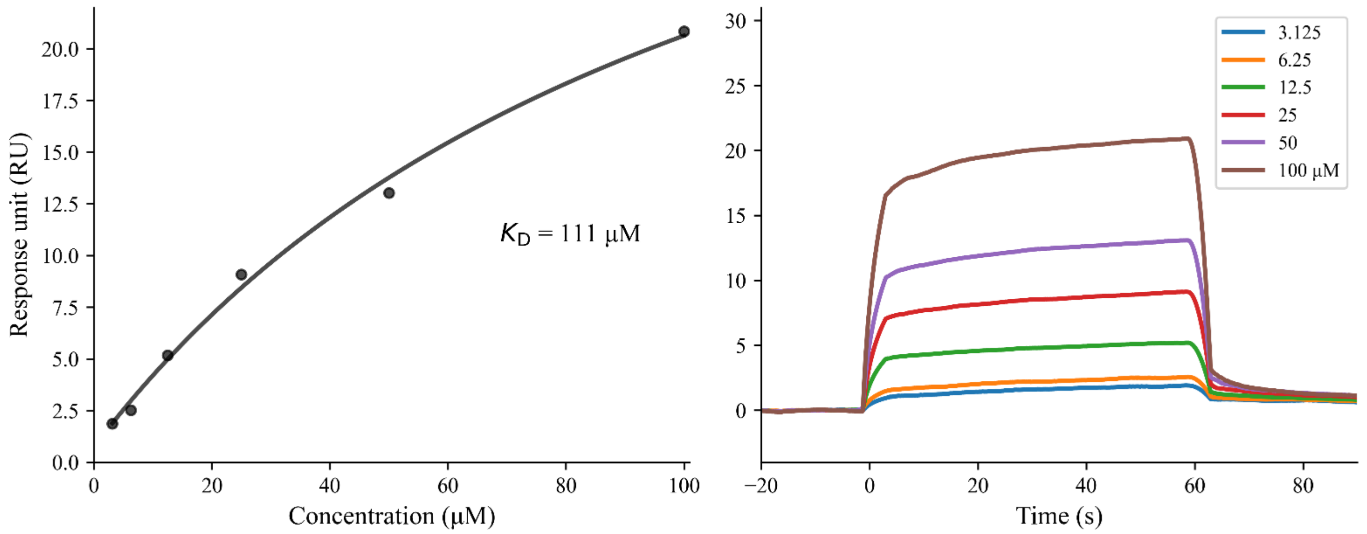

SPC-39

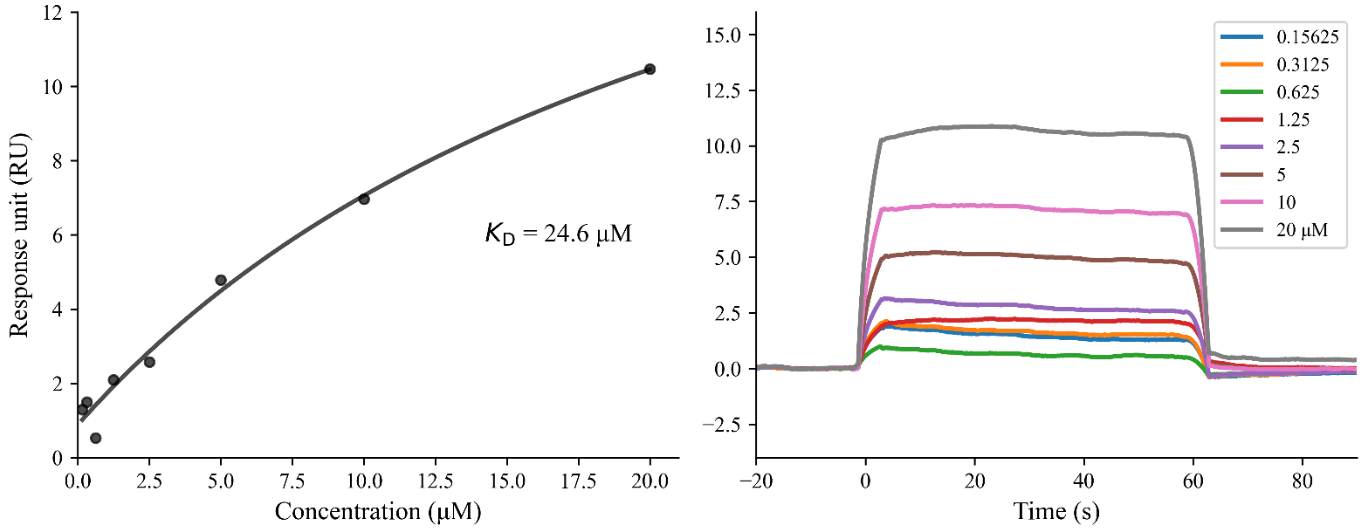

SPC-40

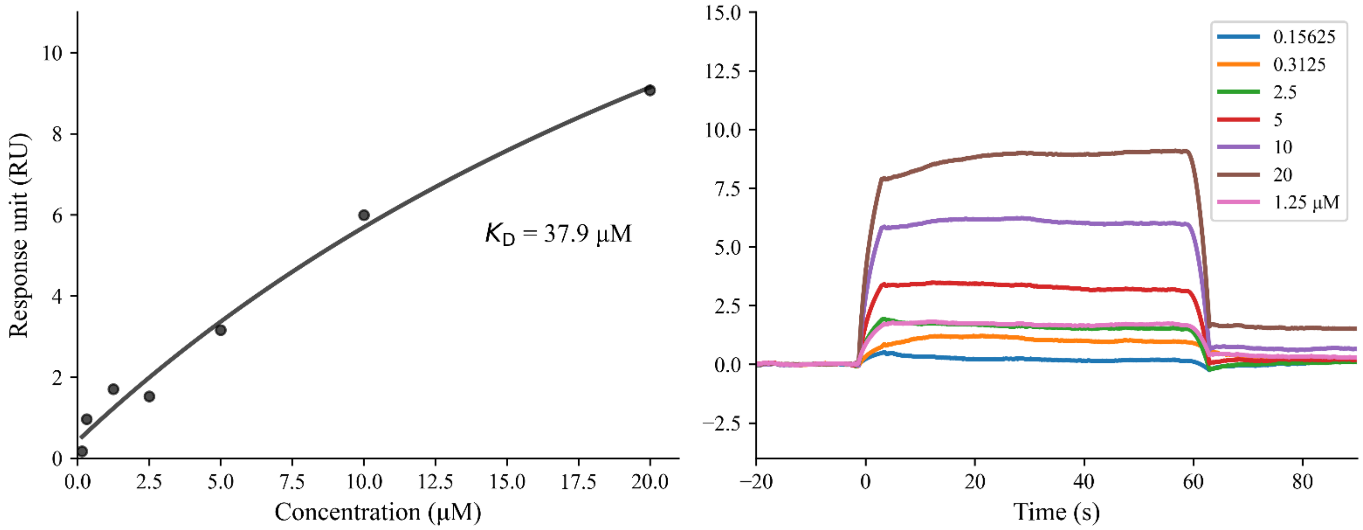

SPC-41

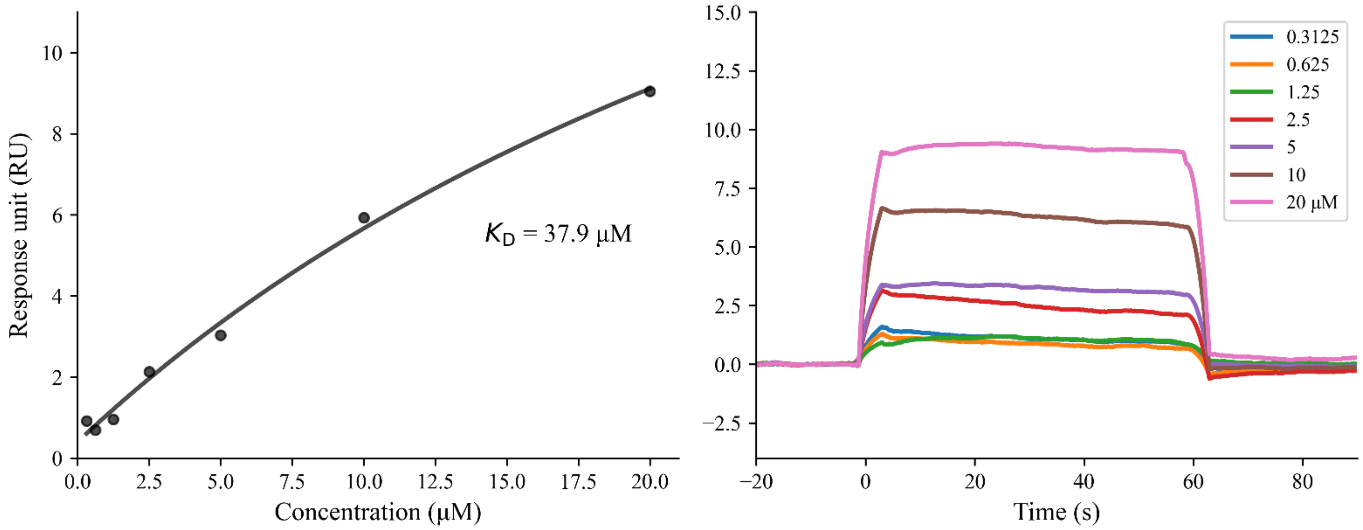

SPC-42

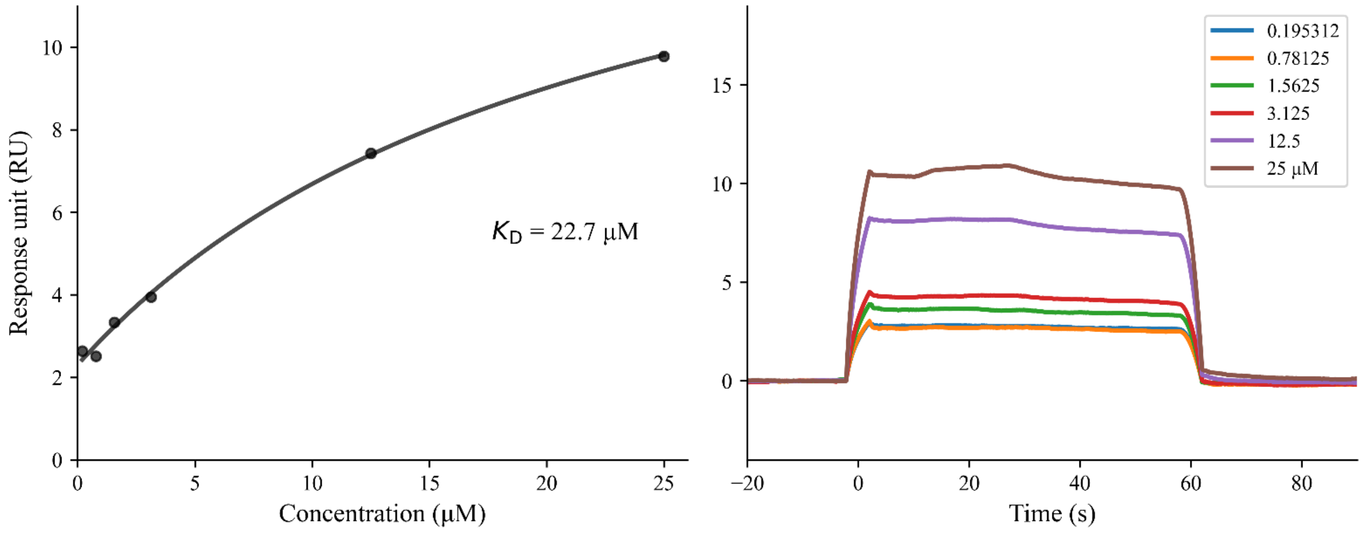

SPC-43

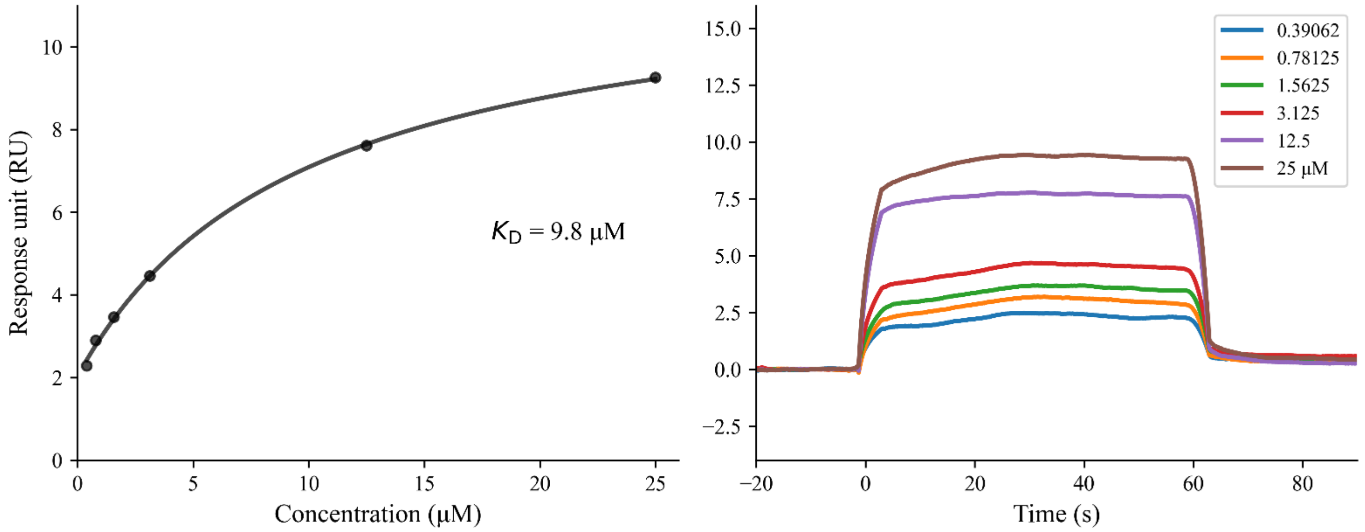

Synthesized compounds:

SPC-14

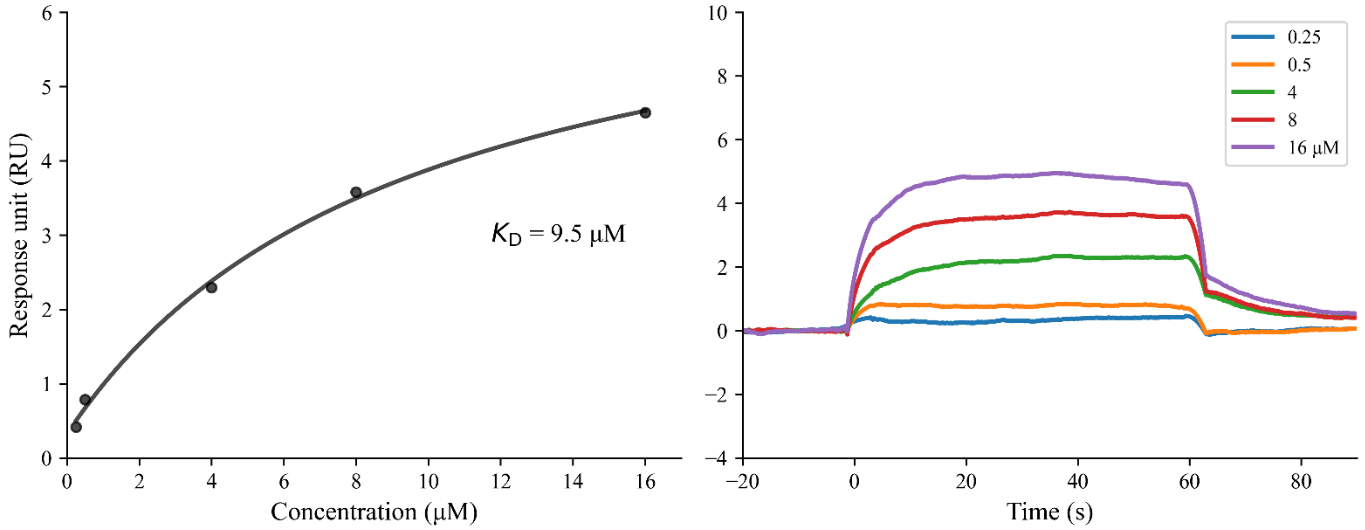

SPC-15

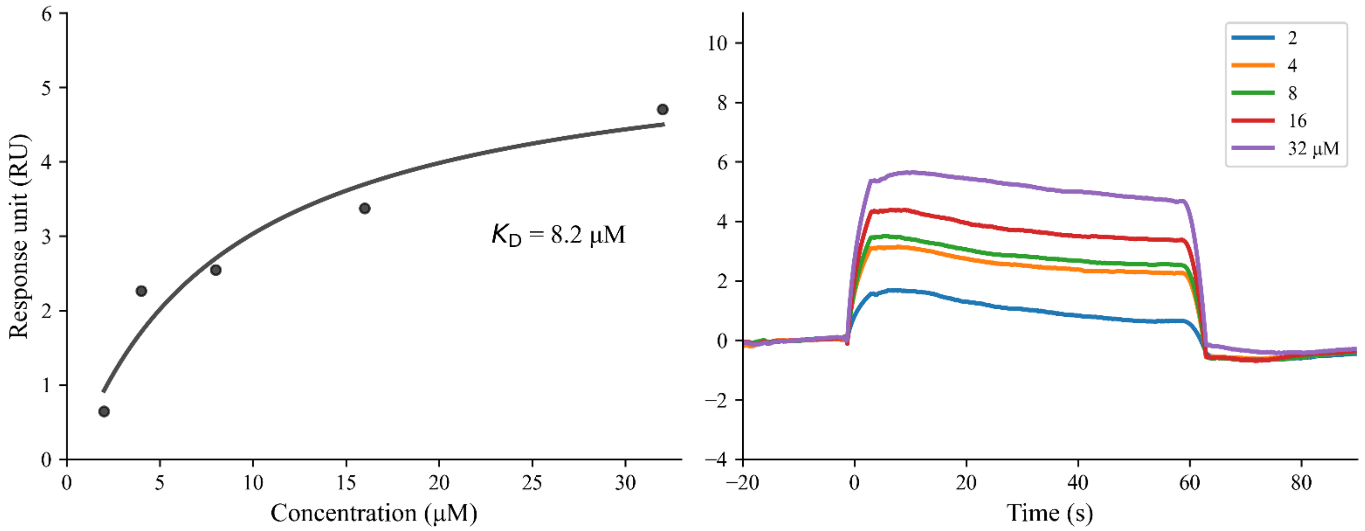

Ester-SPC-15

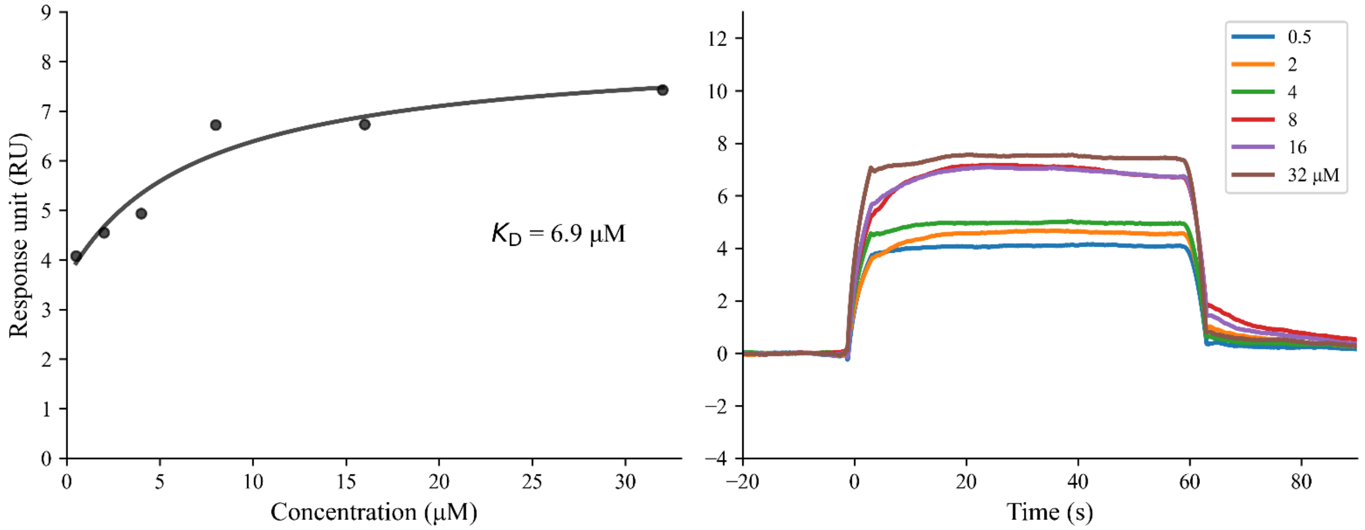

SPC-16

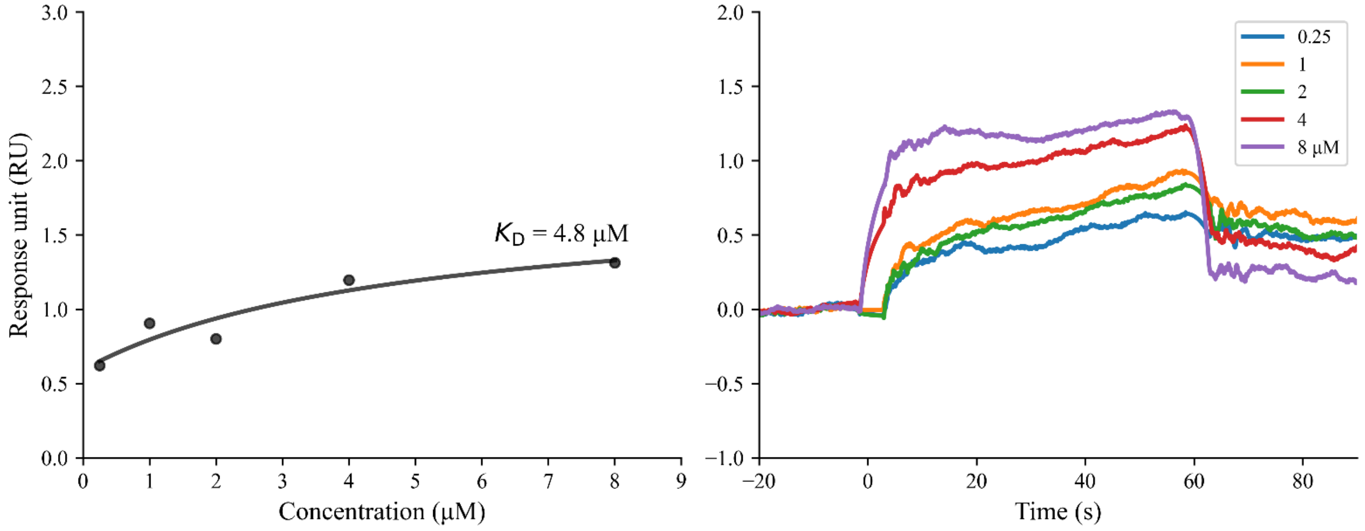

SPC-17

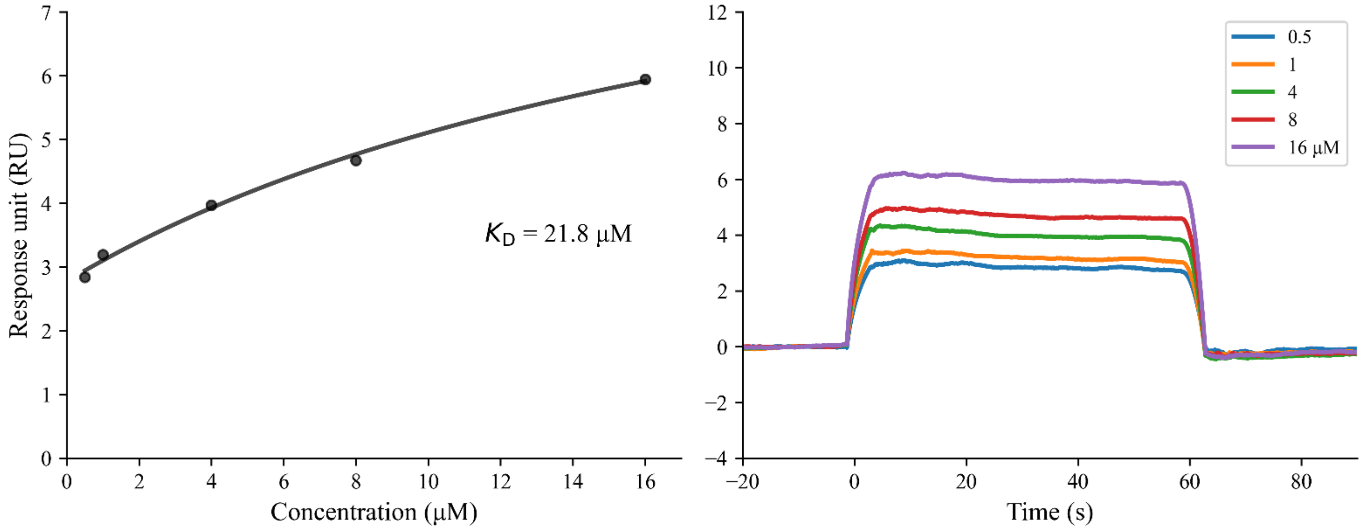

SPC-18

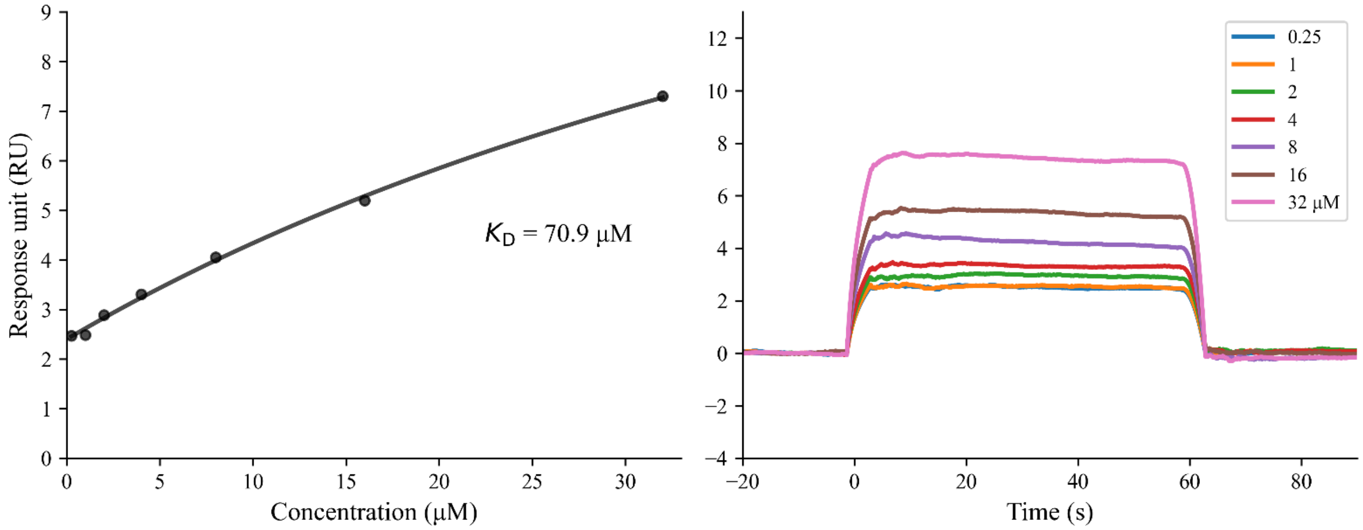

SPC-19

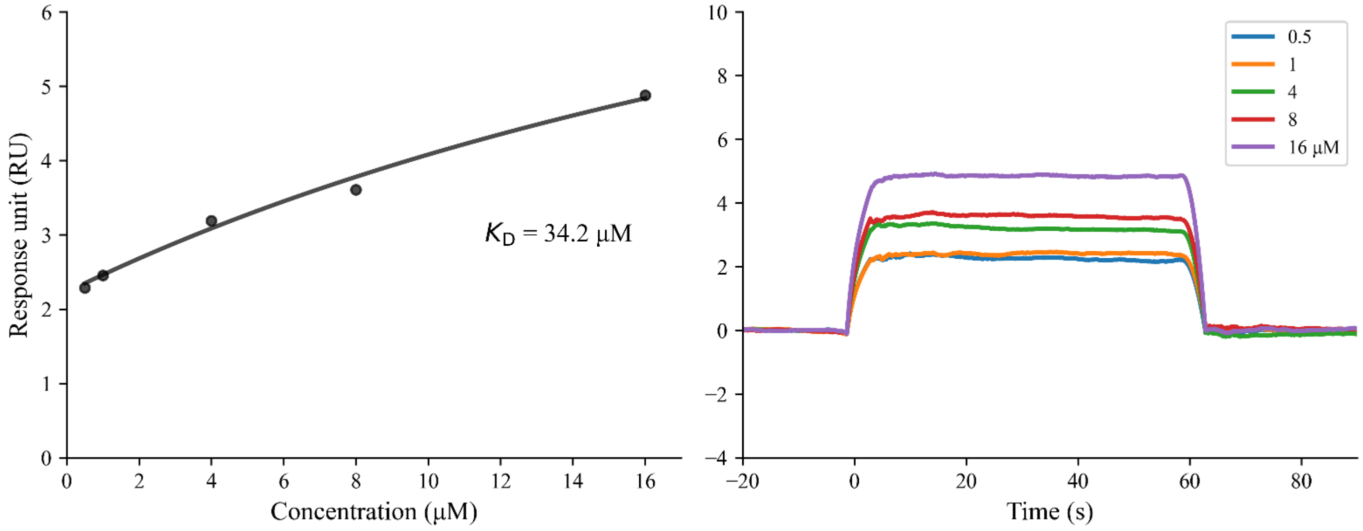

SPC-20

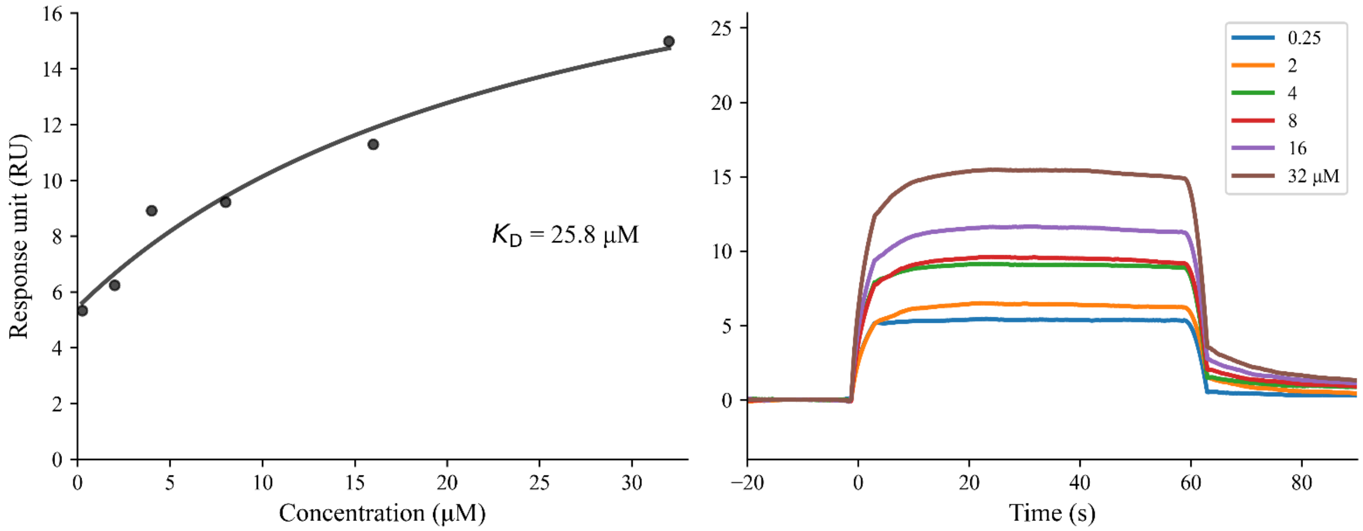

SPC-14, Omicron BA.4

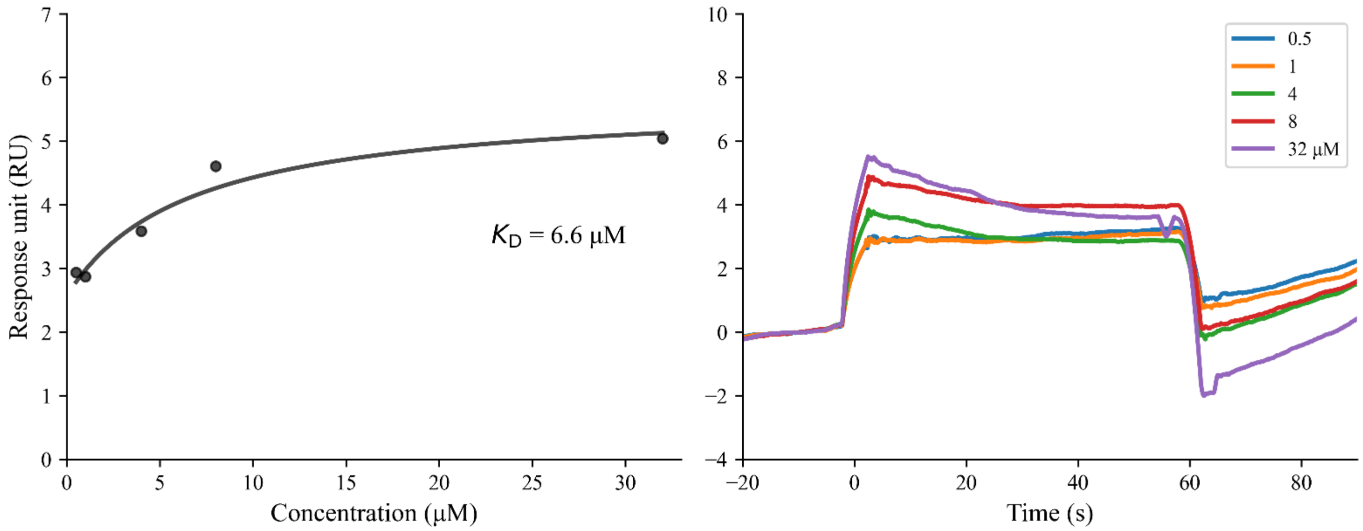

SPC-15, Omicron BA.4

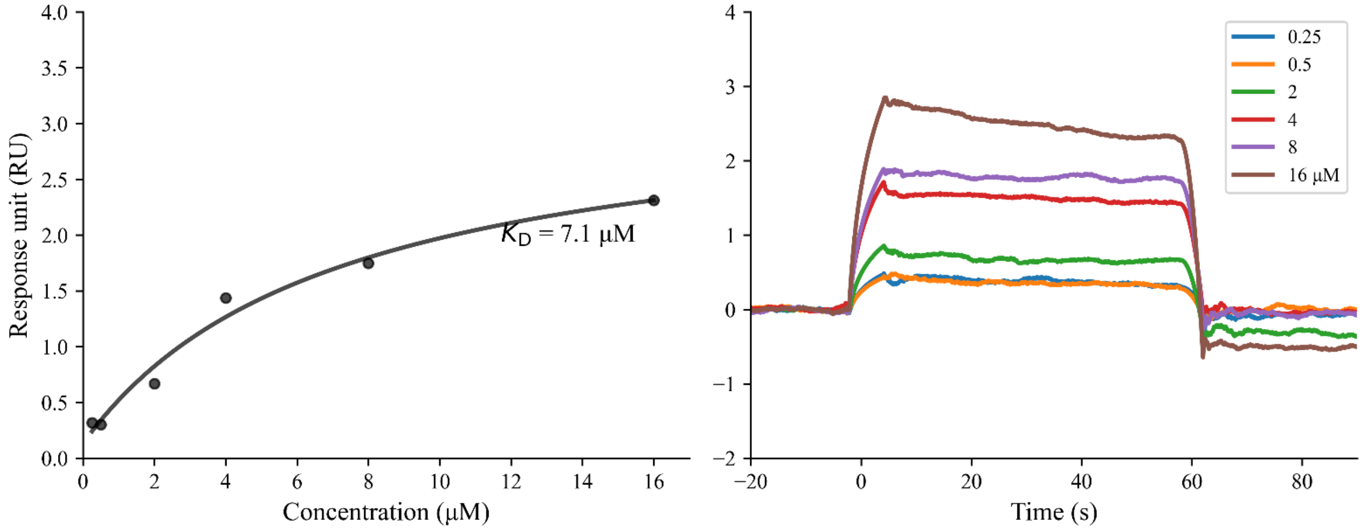

Ester-SPC-15, Omicron BA.4

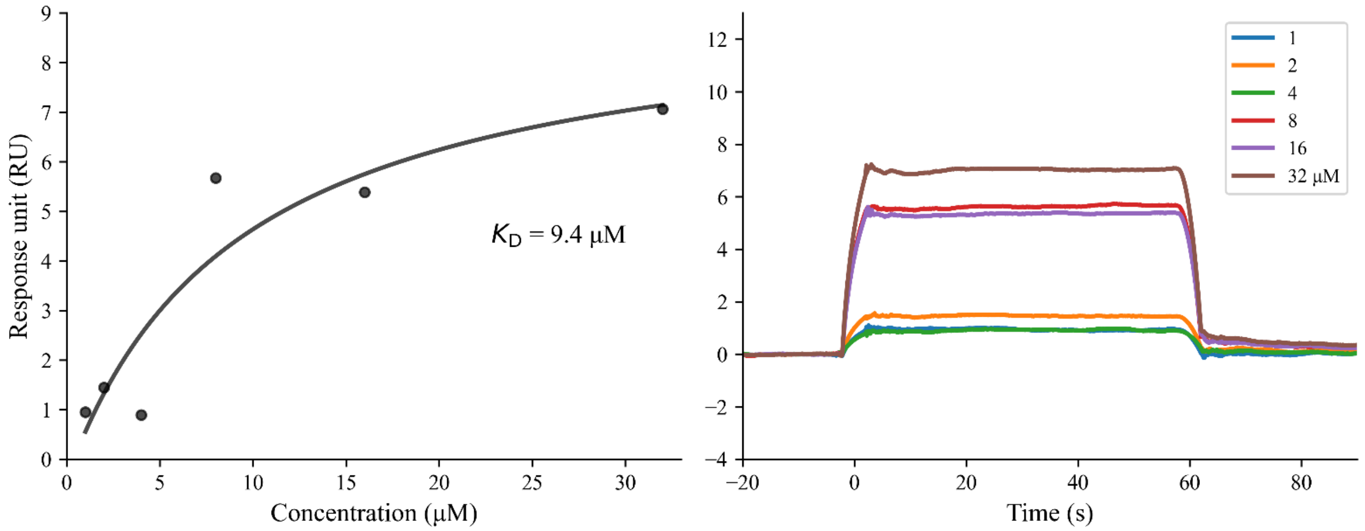

^{1}H NMR spectra and ^{13}C NMR/LC-MS spectra of synthesized compounds.

SPC-14

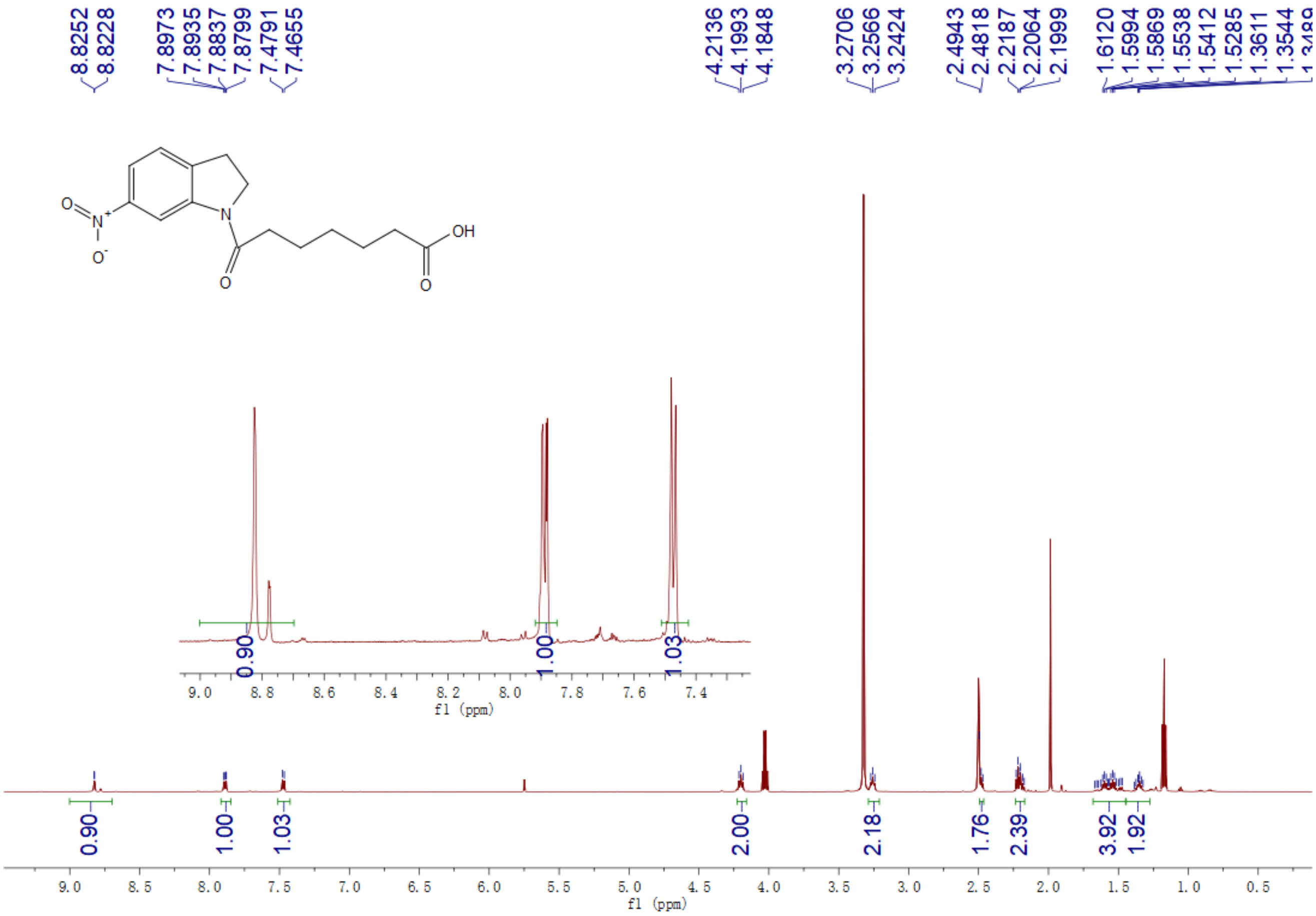

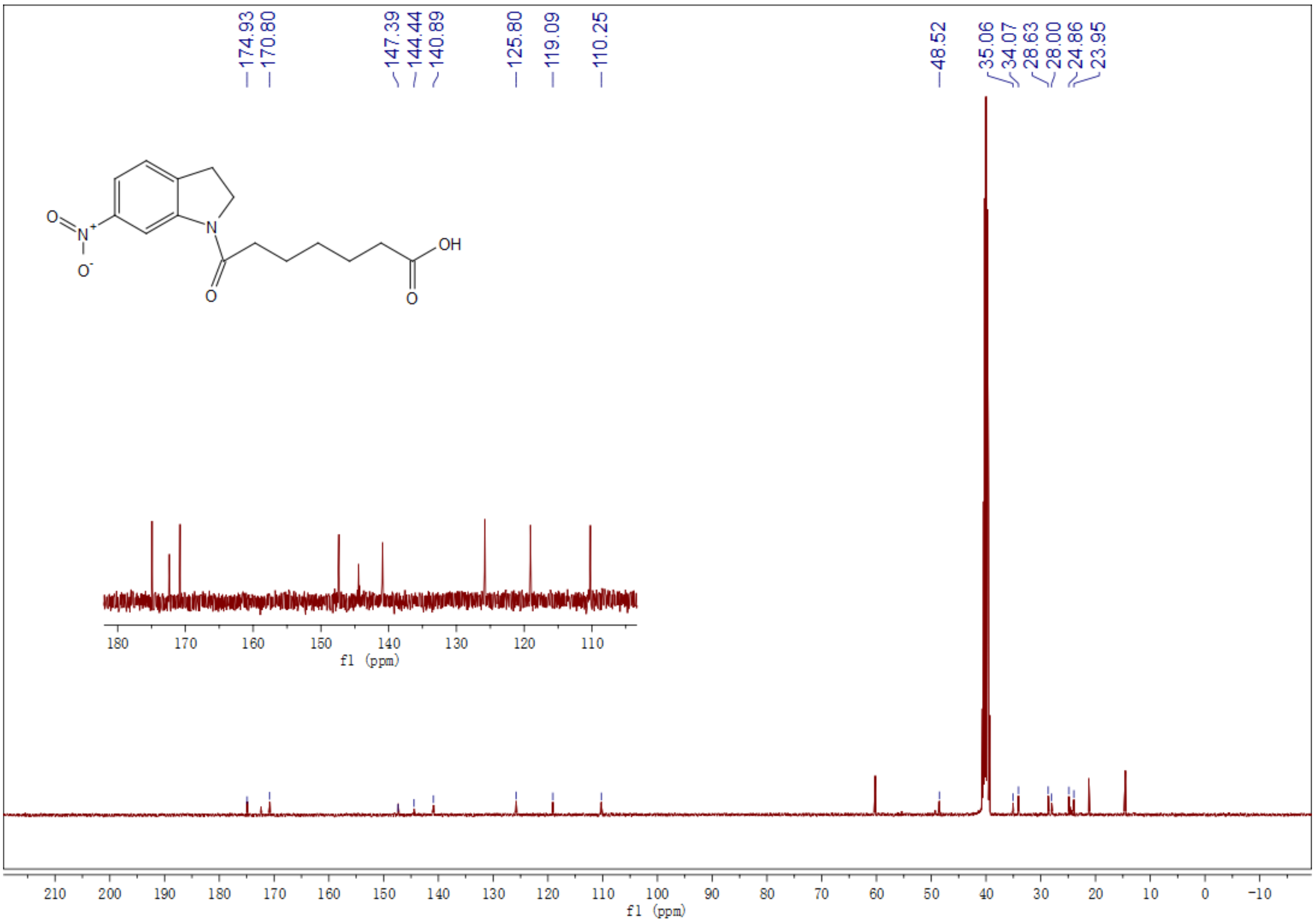

174.93
170.80
147.39
144.44
140.89
125.80
119.09
110.25
48.52
35.06
34.07
28.63
28.00
24.86
23.95
f1 (ppm)

SPC-15

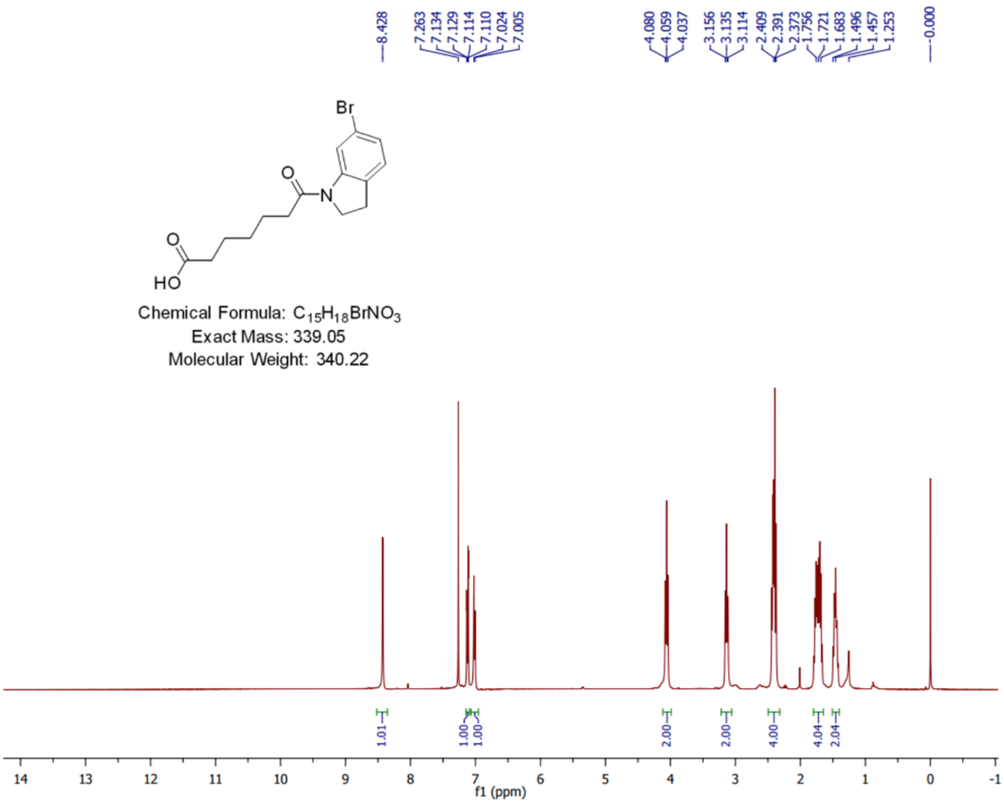

Chemical Formula: $C_{15}H_{18}BrNO_3$
Exact Mass: 339.05
Molecular Weight: 340.22
f1 (ppm)

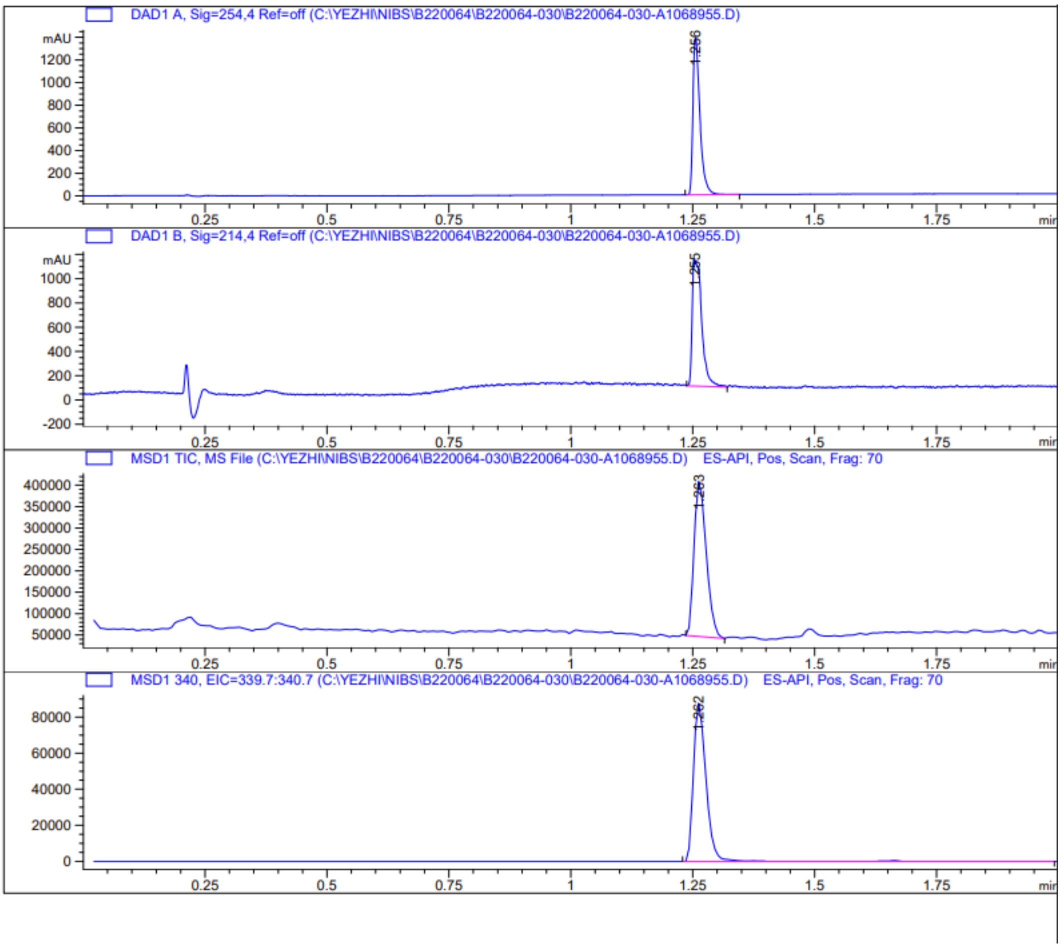

DAD1 A, Sig=254,4 Ref=off (C:\YEZHI\NIBS\B220064\B220064-030\B220064-030-A1068955.D)
mAU
1.256
DAD1 B, Sig=214,4 Ref=off (C:\YEZHI\NIBS\B220064\B220064-030\B220064-030-A1068955.D)
1.255
MSD1 TIC, MS File (C:\YEZHI\NIBS\B220064\B220064-030\B220064-030-A1068955.D) ES-API, Pos, Scan, Frag: 70
1.263
MSD1 340, EIC=339.7:340.7 (C:\YEZHI\NIBS\B220064\B220064-030\B220064-030-A1068955.D) ES-API, Pos, Scan, Frag: 70
1.262

MS Spectrum

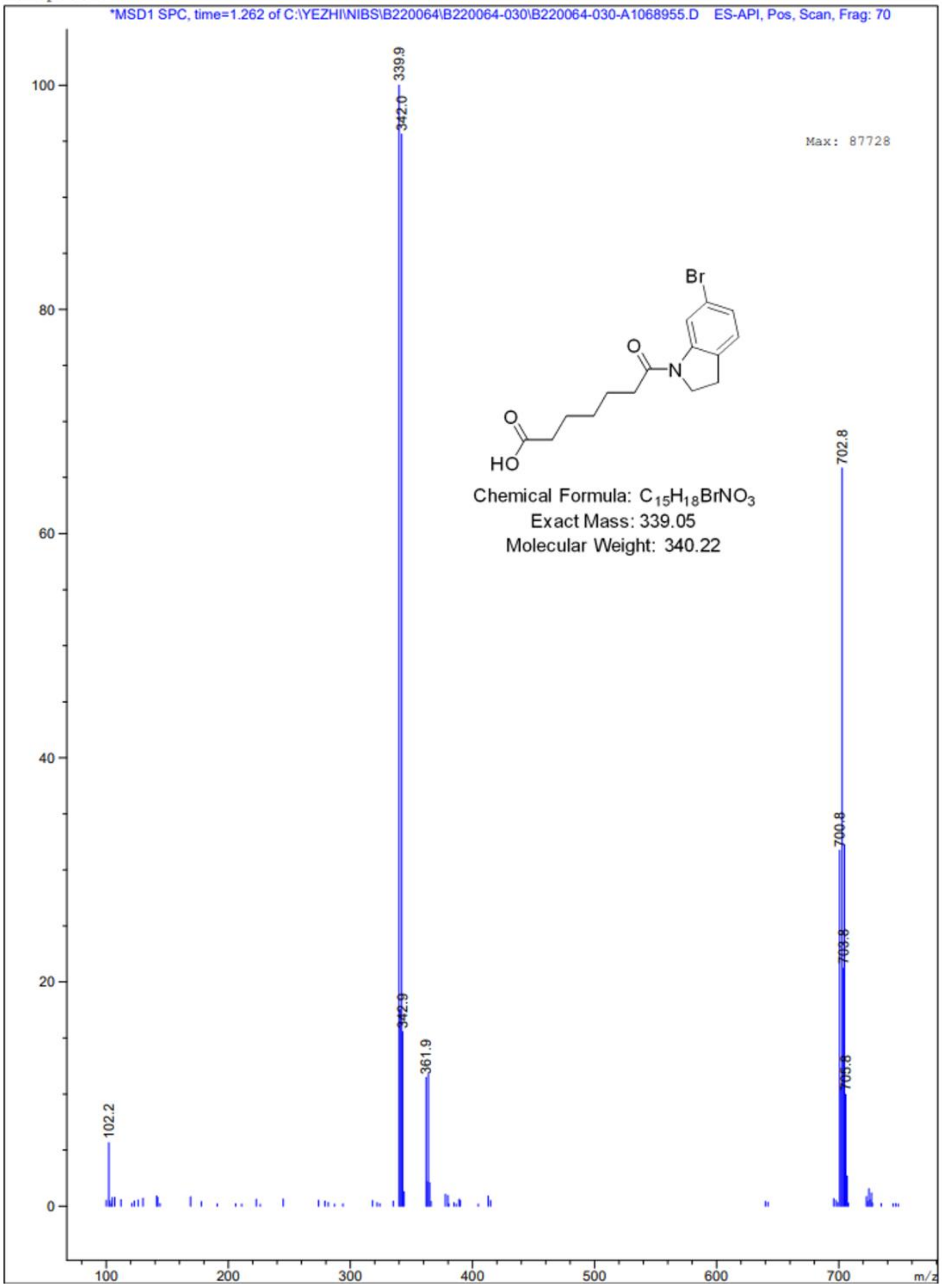

*MSD1 SPC, time=1.262 of C:\YEZHI\NIBS\B220064\B220064-030\B220064-030-A1068955.D ES-API, Pos, Scan, Frag: 70
Max: 87728
339.9
342.0
342.9
361.9
102.2
702.8
700.8
703.8
705.8
Br
O
N
O
HO
Chemical Formula: C15H18BrNO3
Exact Mass: 339.05
Molecular Weight: 340.22
m/z

Ester-SPC-15

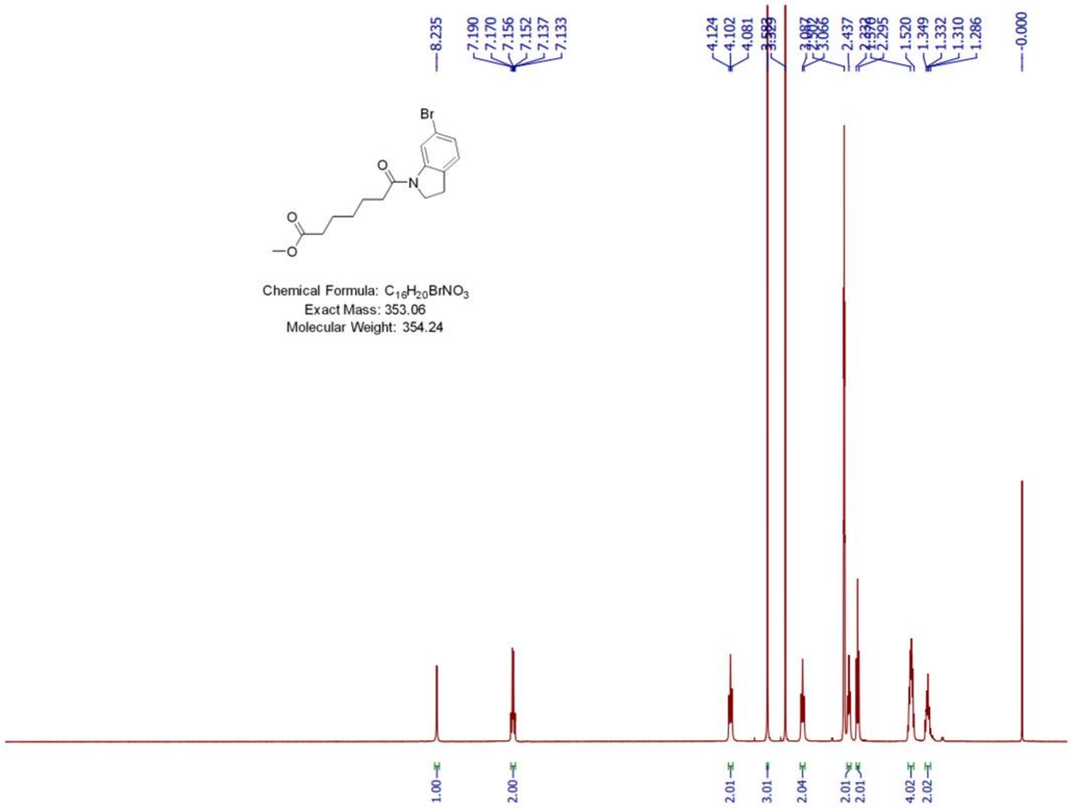

Chemical Formula: $C_{16}H_{20}BrNO_3$
Exact Mass: 353.06
Molecular Weight: 354.24

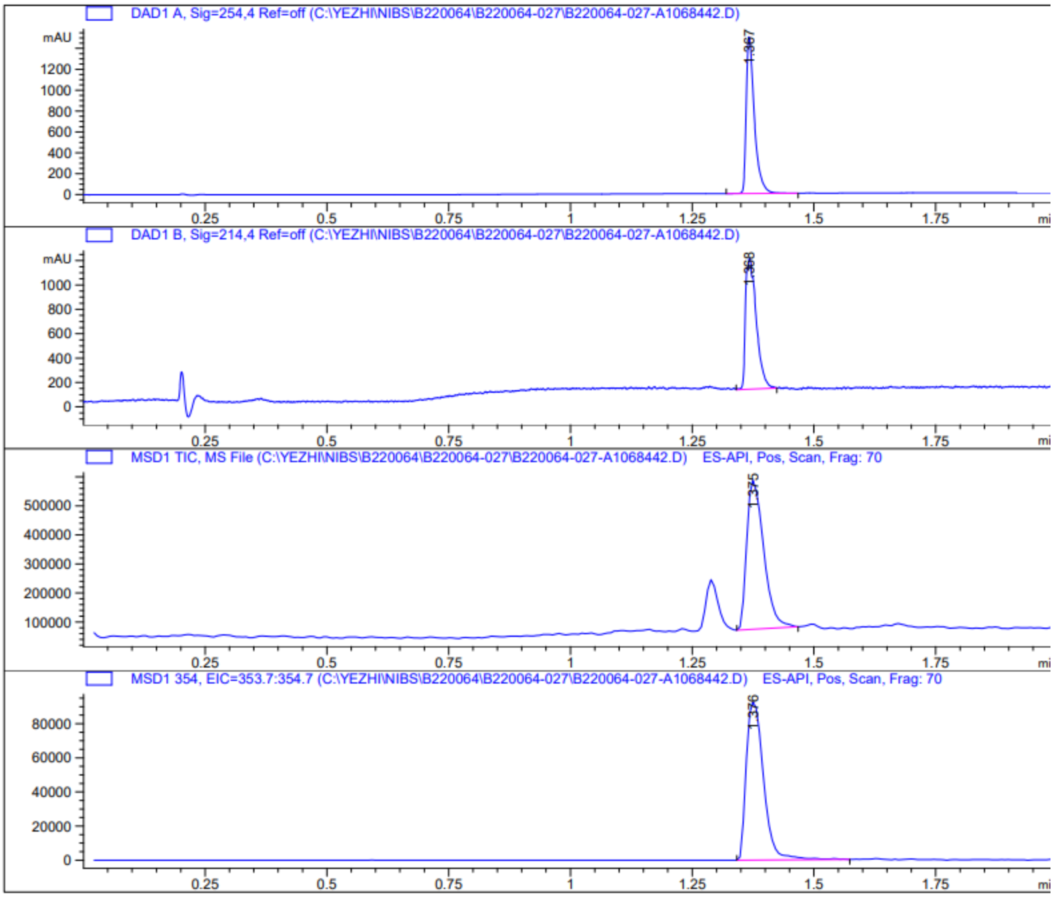

DAD1 A, Sig=254,4 Ref=off (C:\YEZHI\NIBS\B220064\B220064-027\B220064-027-A1068442.D)
DAD1 B, Sig=214,4 Ref=off (C:\YEZHI\NIBS\B220064\B220064-027\B220064-027-A1068442.D)
MSD1 TIC, MS File (C:\YEZHI\NIBS\B220064\B220064-027\B220064-027-A1068442.D) ES-API, Pos, Scan, Frag: 70
MSD1 354, EIC=353.7:354.7 (C:\YEZHI\NIBS\B220064\B220064-027\B220064-027-A1068442.D) ES-API, Pos, Scan, Frag: 70

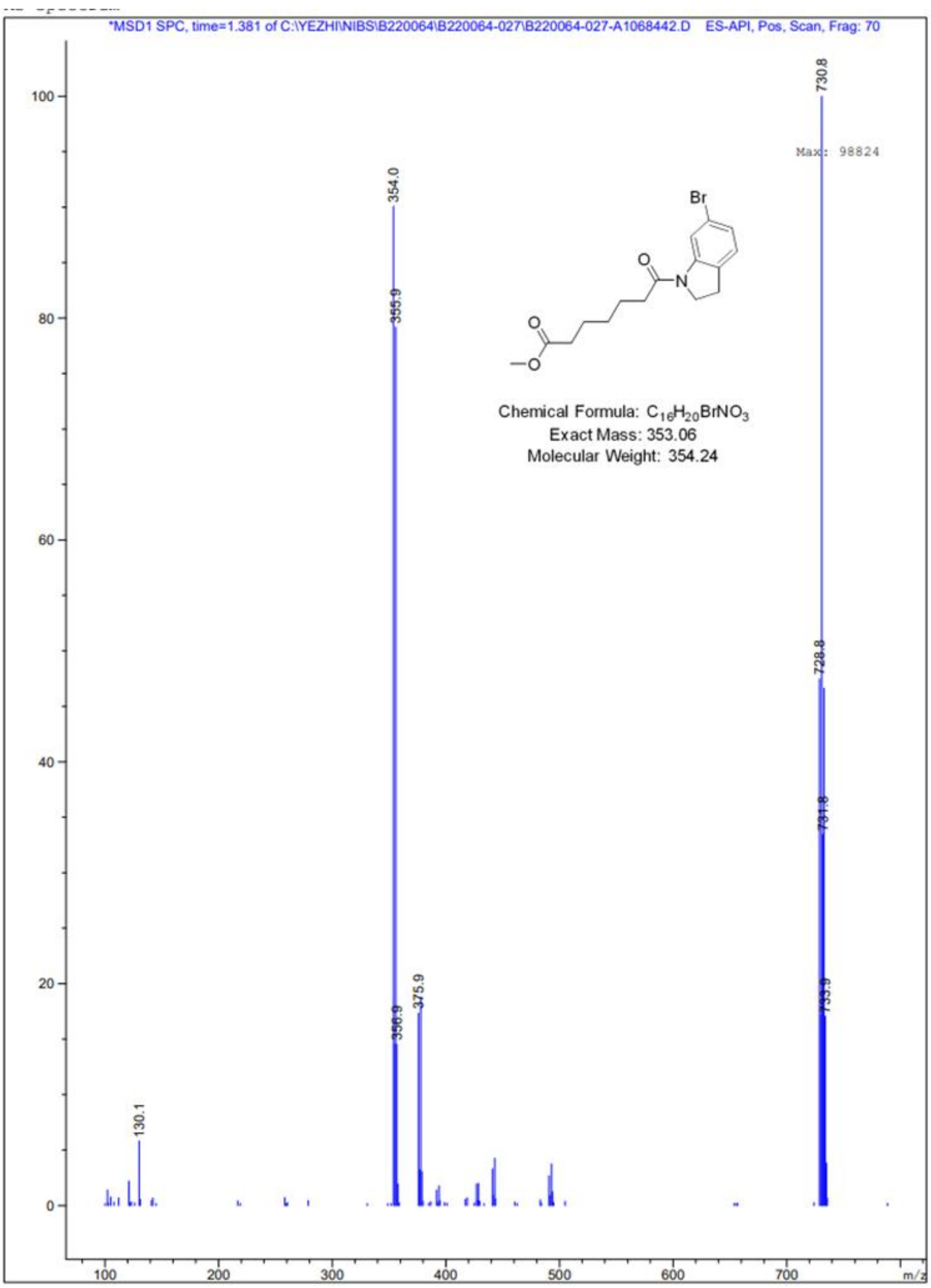
*MSD1 SPC, time=1.381 of C:\YEZHI\NIBS\B220064\B220064-027\B220064-027-A1068442.D ES-API, Pos, Scan, Frag: 70
Max: 98824
730.8
354.0
355.9
728.8
731.8
375.9
356.9
733.9
130.1
Br
O
N
O
O
Chemical Formula: C16H20BrNO3
Exact Mass: 353.06
Molecular Weight: 354.24
m/z

SPC-16

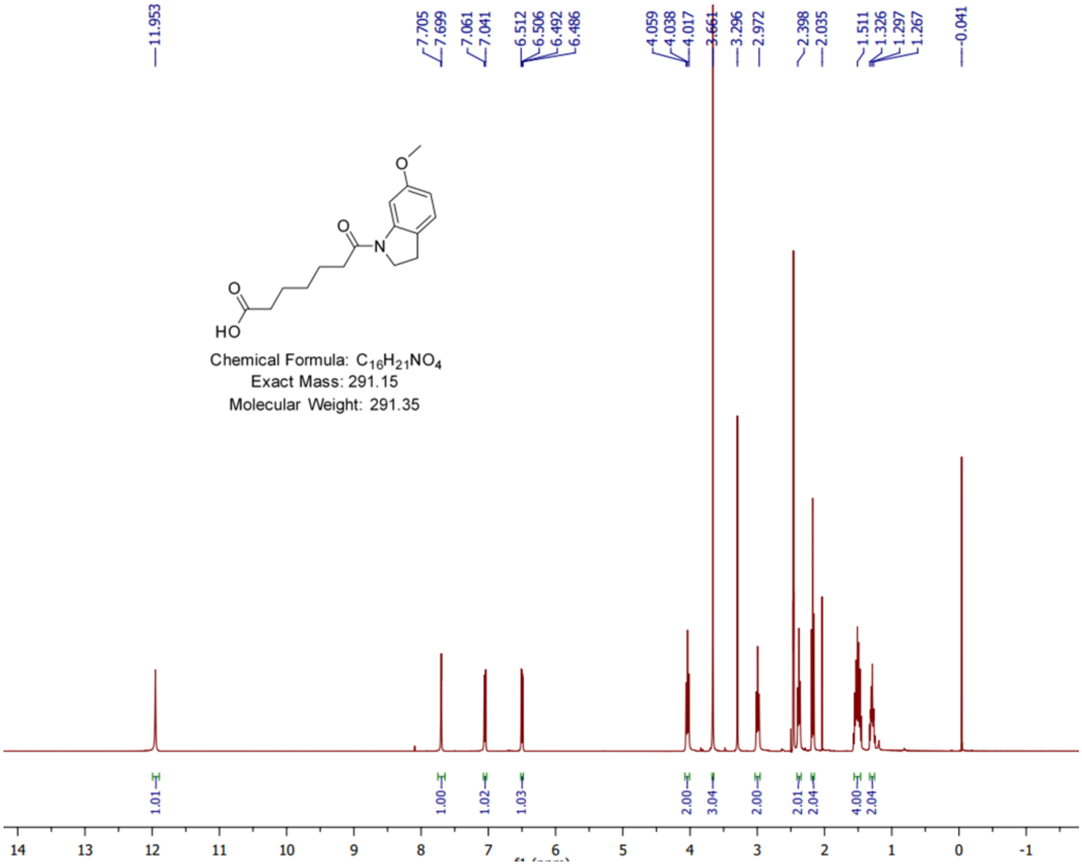

Chemical Formula: $C_{16}H_{21}NO_4$
Exact Mass: 291.15
Molecular Weight: 291.35

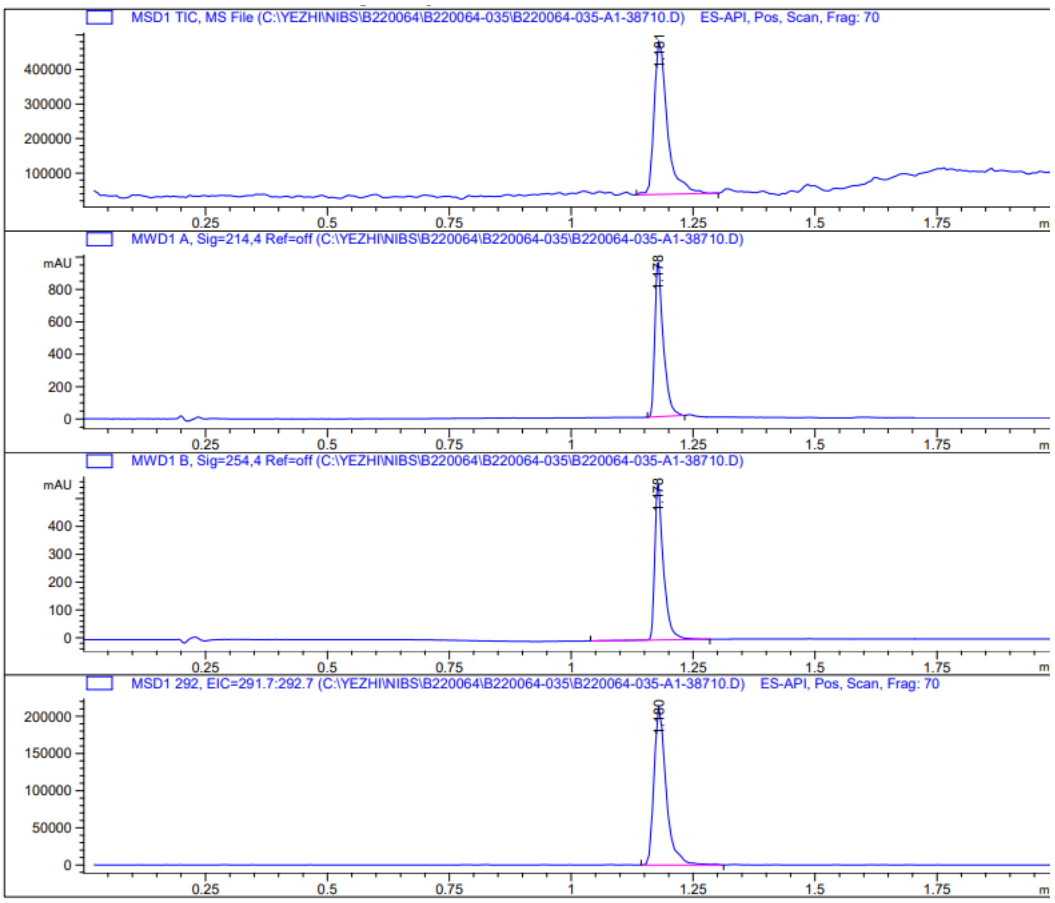

MSD1 TIC, MS File (C:\YEZHI\NIBS\B220064\B220064-035\B220064-035-A1-38710.D) ES-API, Pos, Scan, Frag: 70
MWD1 A, Sig=214,4 Ref=off (C:\YEZHI\NIBS\B220064\B220064-035\B220064-035-A1-38710.D)
MWD1 B, Sig=254,4 Ref=off (C:\YEZHI\NIBS\B220064\B220064-035\B220064-035-A1-38710.D)
MSD1 292, EIC=291.7:292.7 (C:\YEZHI\NIBS\B220064\B220064-035\B220064-035-A1-38710.D) ES-API, Pos, Scan, Frag: 70

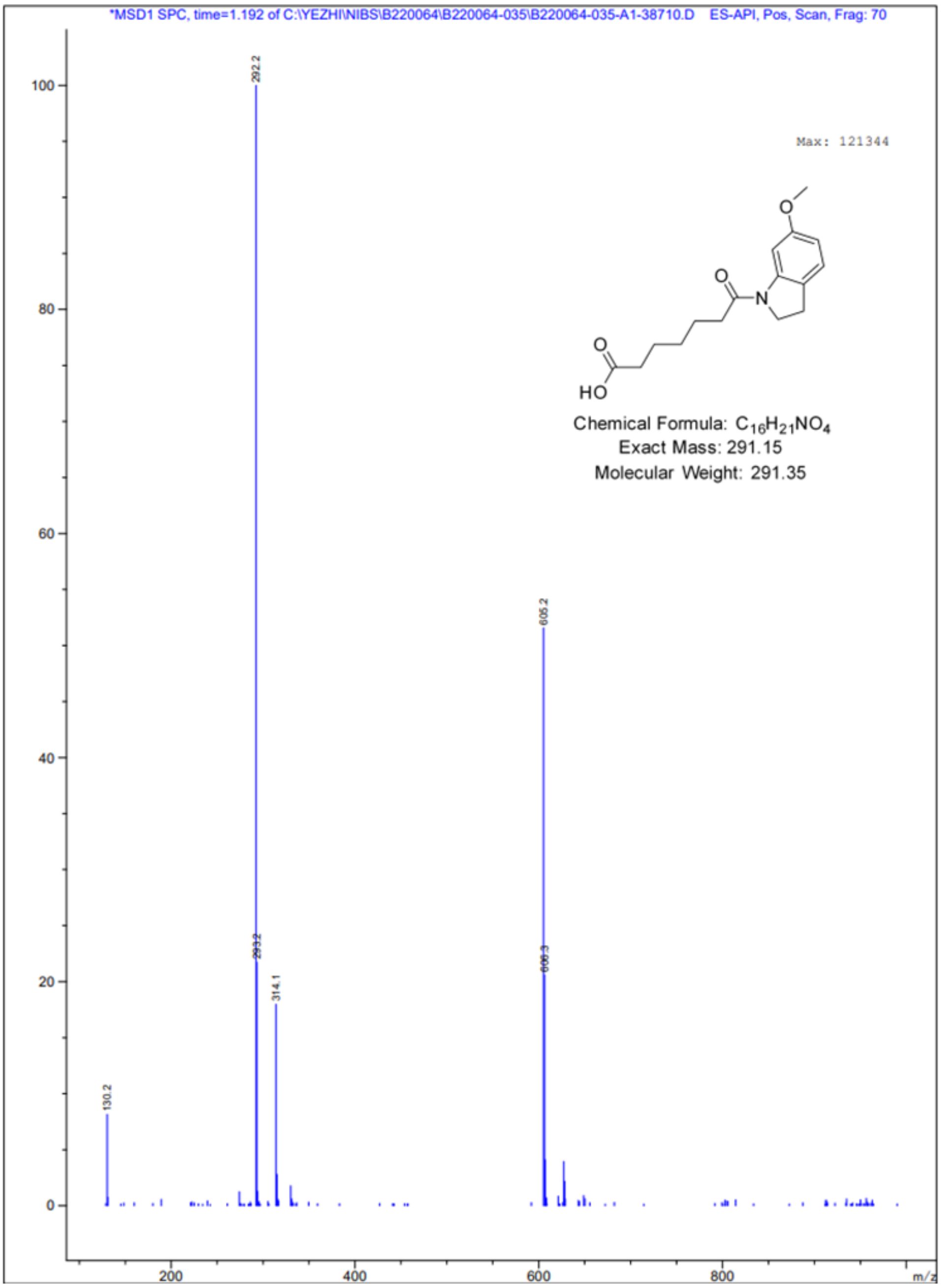

*MSD1 SPC, time=1.192 of C:\YEZHI\NIBS\B220064\B220064-035\B220064-035-A1-38710.D ES-API, Pos, Scan, Frag: 70
Max: 121344
292.2
293.2
314.1
130.2
605.2
606.3
Chemical Formula: C16H21NO4
Exact Mass: 291.15
Molecular Weight: 291.35
m/z

SPC-17

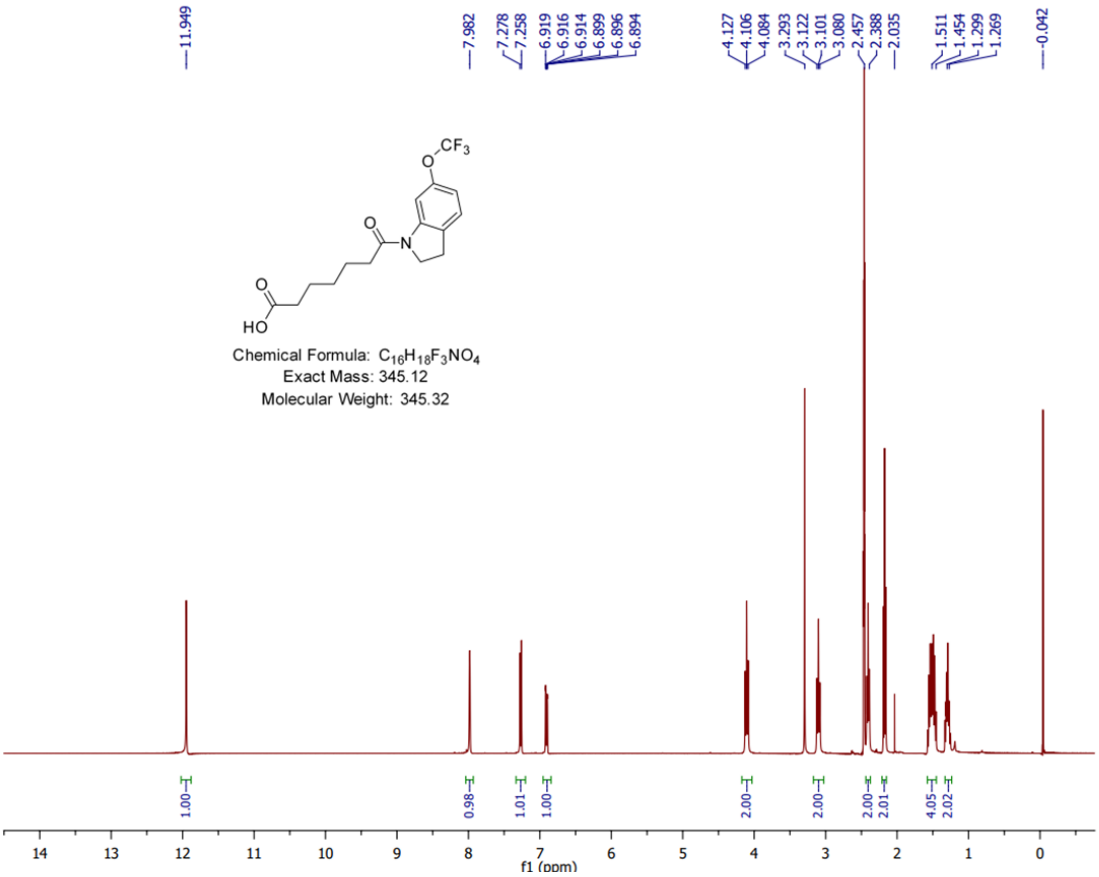
Chemical Formula: $C_{16}H_{18}F_3NO_4$
Exact Mass: 345.12
Molecular Weight: 345.32
f1 (ppm)

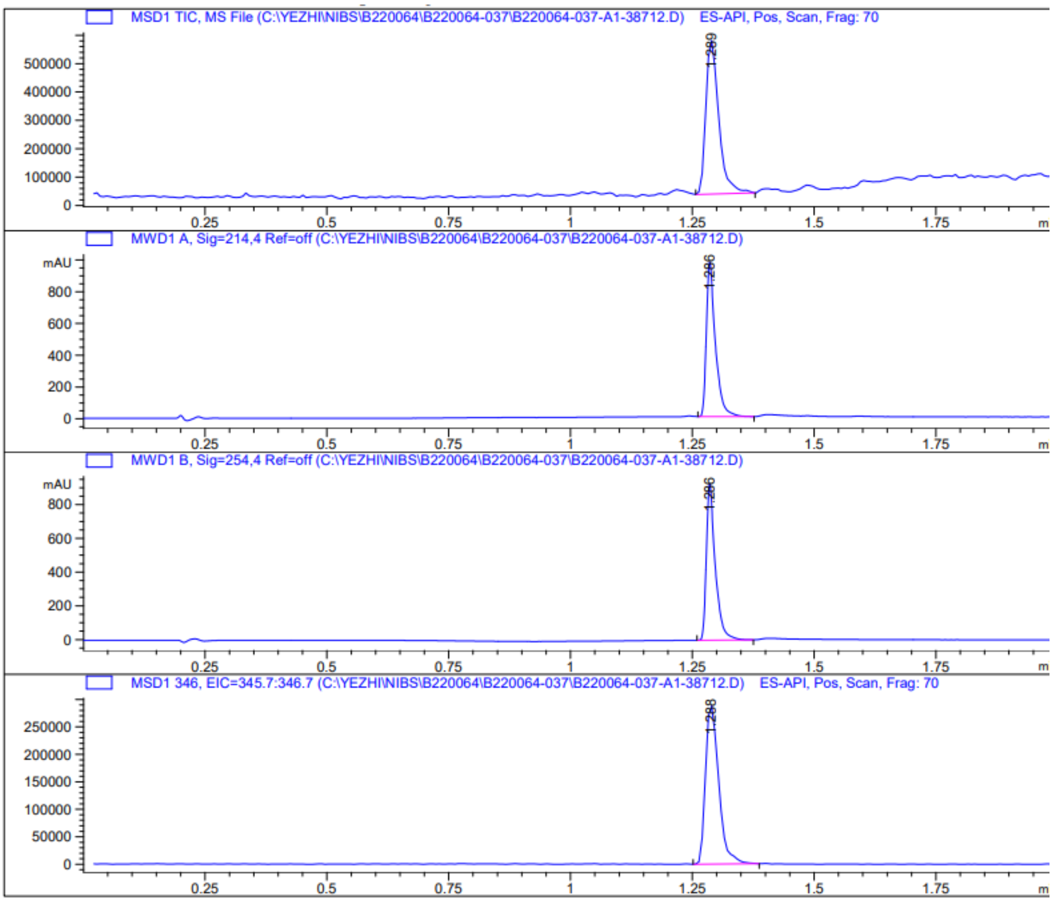
MSD1 TIC, MS File (C:\YEZHI\NIBS\B220064\B220064-037\B220064-037-A1-38712.D) ES-API, Pos, Scan, Frag: 70
MWD1 A, Sig=214,4 Ref=off (C:\YEZHI\NIBS\B220064\B220064-037\B220064-037-A1-38712.D)
MWD1 B, Sig=254,4 Ref=off (C:\YEZHI\NIBS\B220064\B220064-037\B220064-037-A1-38712.D)
MSD1 346, EIC=345.7:346.7 (C:\YEZHI\NIBS\B220064\B220064-037\B220064-037-A1-38712.D) ES-API, Pos, Scan, Frag: 70

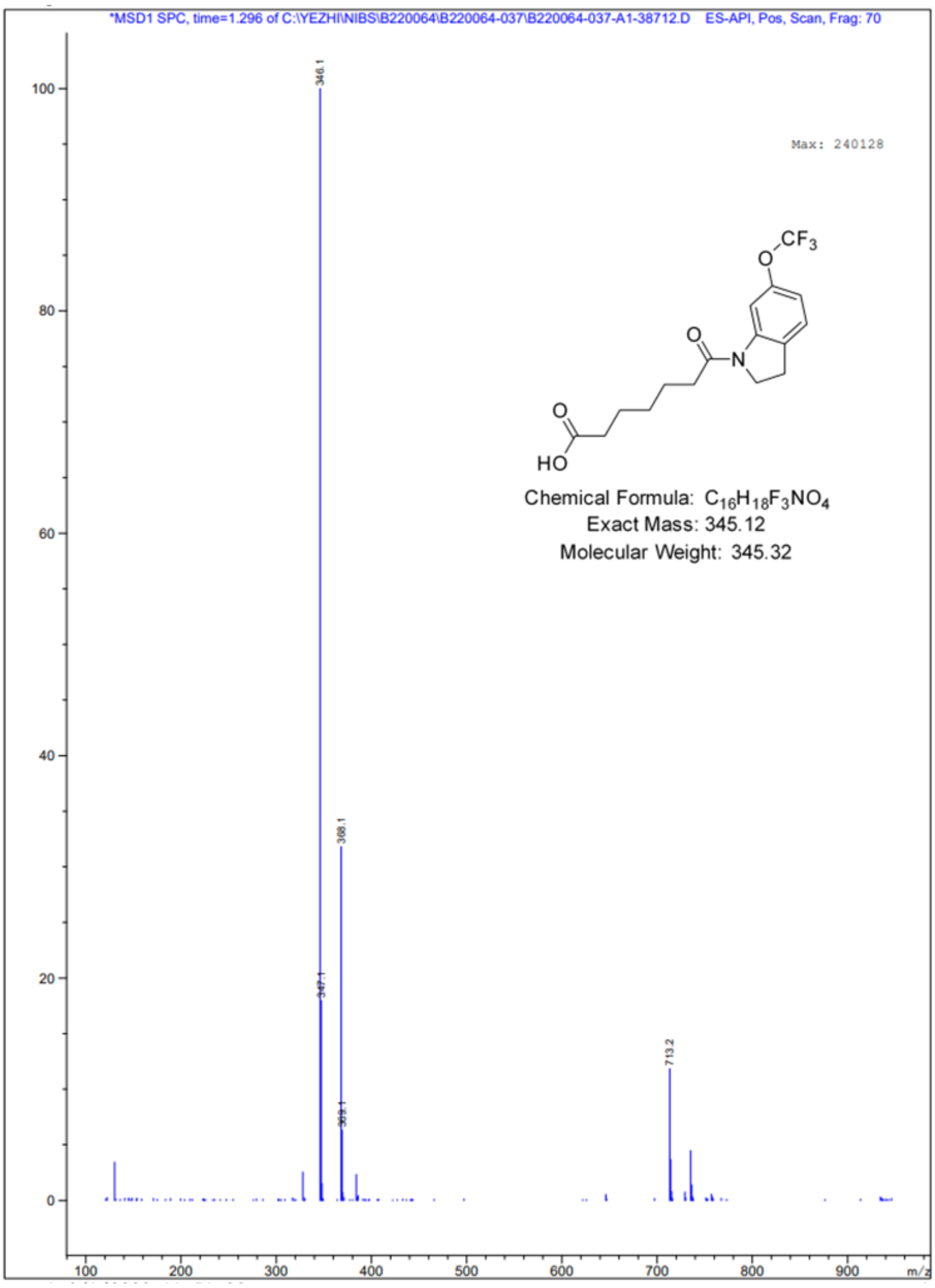
*MSD1 SPC, time=1.296 of C:\YEZHI\NIBS\B220064\B220064-037\B220064-037-A1-38712.D ES-API, Pos, Scan, Frag: 70
Max: 240128
346.1
368.1
347.1
369.1
713.2
m/z
Chemical Formula: C16H18F3NO4
Exact Mass: 345.12
Molecular Weight: 345.32

SPC-18

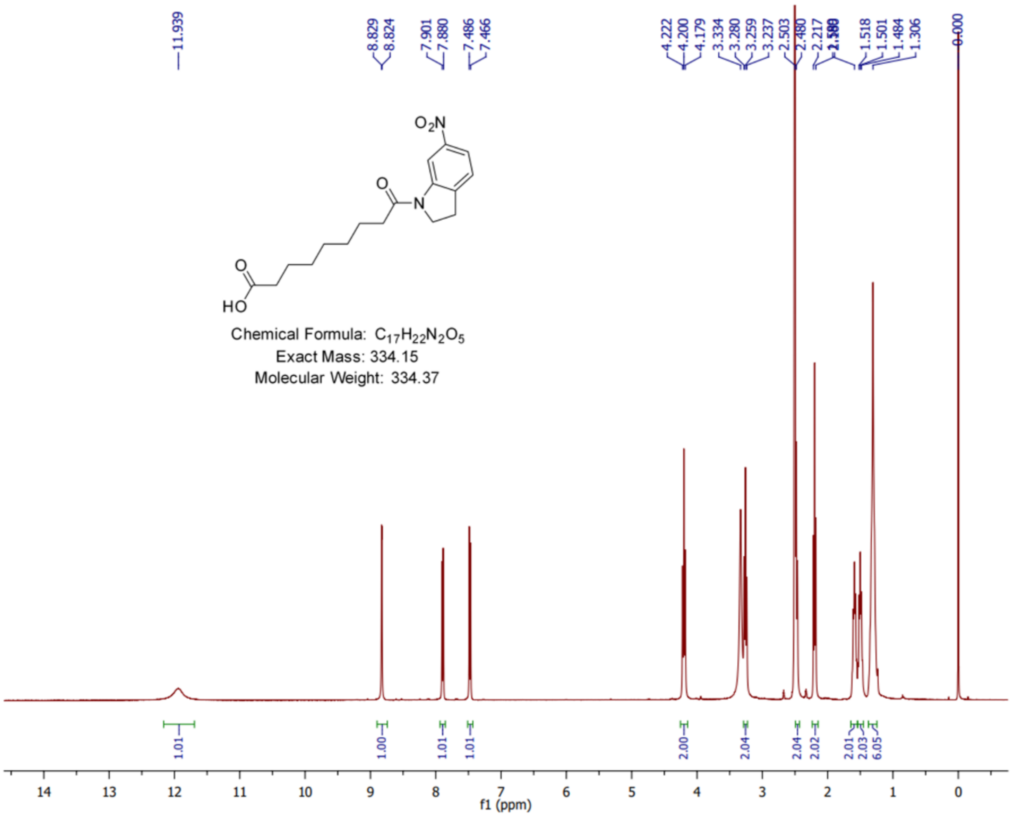

Chemical Formula: $C_{17}H_{22}N_2O_5$
Exact Mass: 334.15
Molecular Weight: 334.37
f1 (ppm)

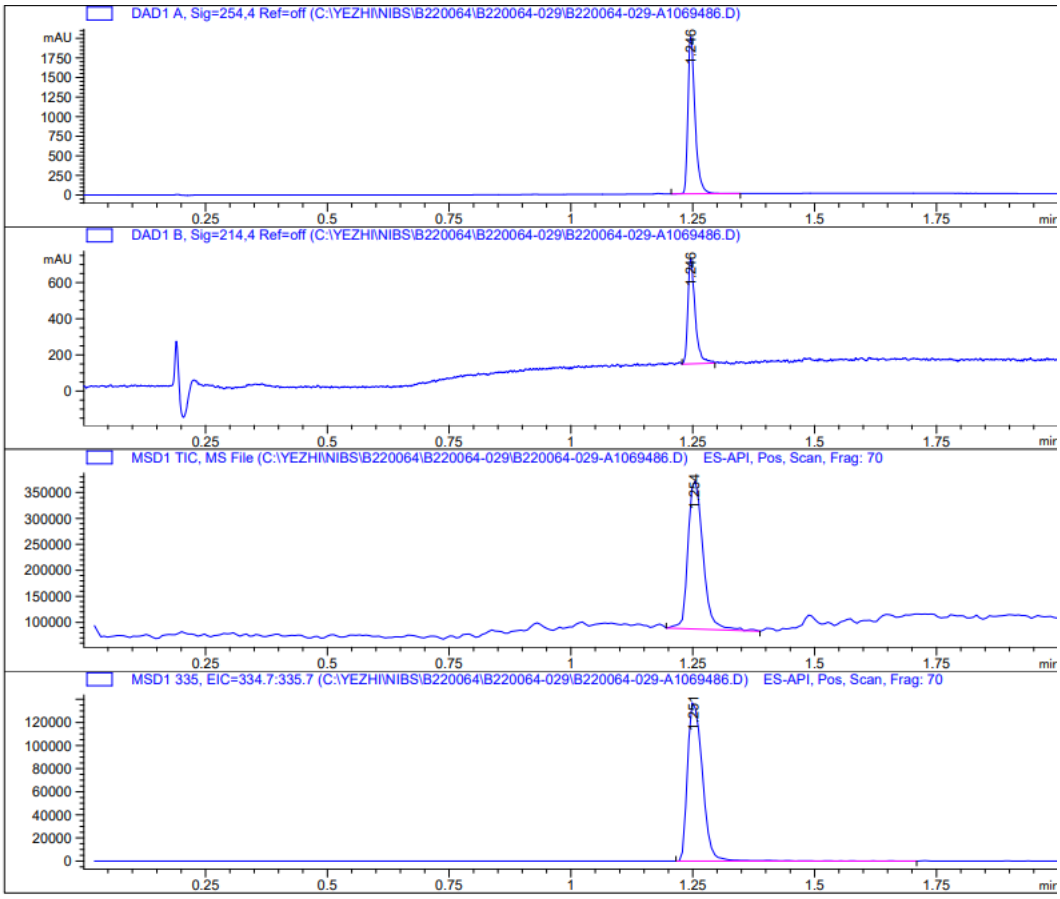

DAD1 A, Sig=254,4 Ref=off (C:\YEZHI\NIBS\B220064\B220064-029\B220064-029-A1069486.D)
DAD1 B, Sig=214,4 Ref=off (C:\YEZHI\NIBS\B220064\B220064-029\B220064-029-A1069486.D)
MSD1 TIC, MS File (C:\YEZHI\NIBS\B220064\B220064-029\B220064-029-A1069486.D) ES-API, Pos, Scan, Frag: 70
MSD1 335, EIC=334.7:335.7 (C:\YEZHI\NIBS\B220064\B220064-029\B220064-029-A1069486.D) ES-API, Pos, Scan, Frag: 70

MS Spectrum

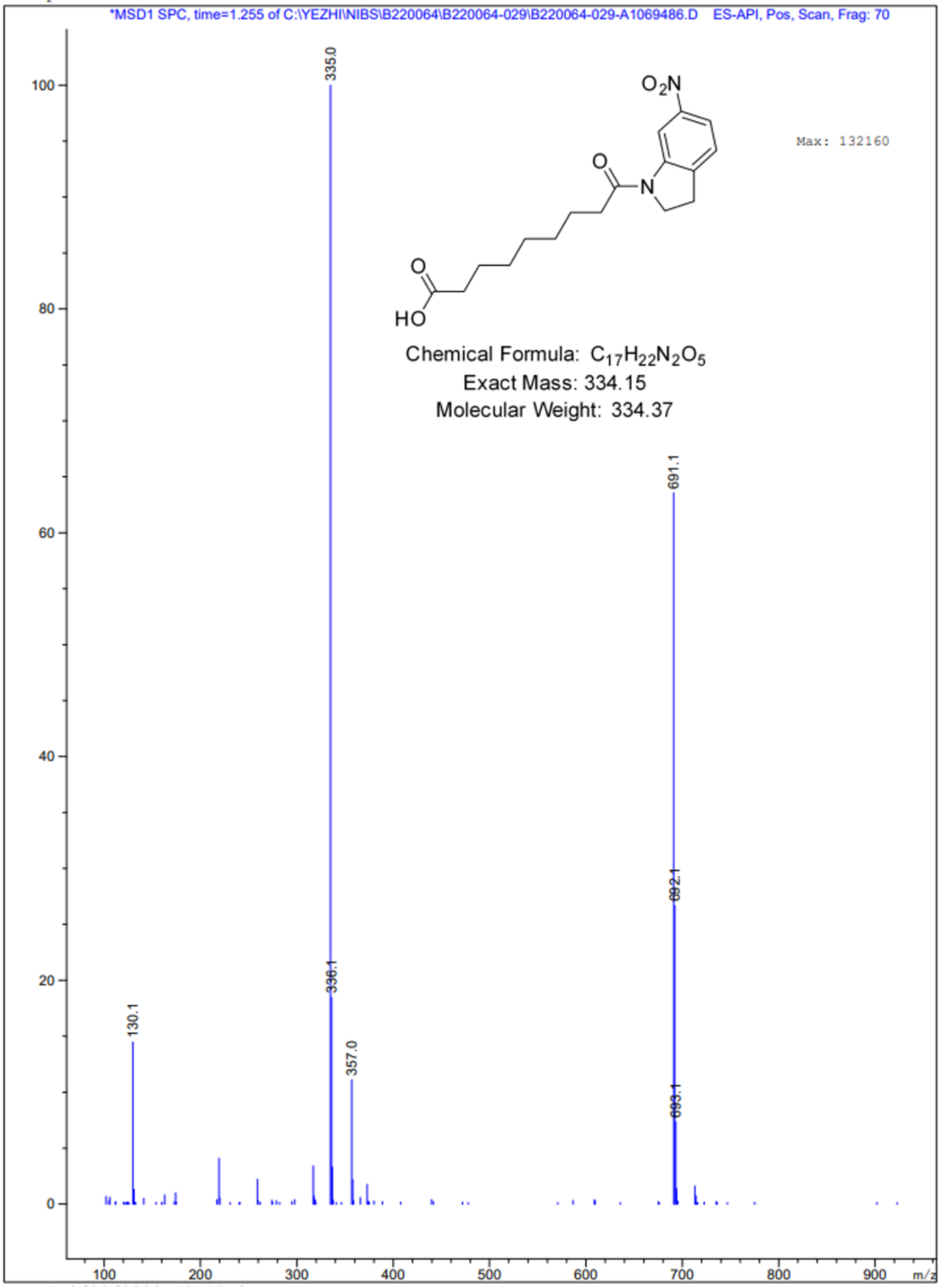
*MSD1 SPC, time=1.255 of C:\YEZHI\NIBS\B220064\B220064-029\B220064-029-A1069486.D ES-API, Pos, Scan, Frag: 70
Max: 132160
O_2N
O
N
O
HO
Chemical Formula: $C_{17}H_{22}N_2O_5$
Exact Mass: 334.15
Molecular Weight: 334.37
335.0
691.1
692.1
336.1
130.1
357.0
693.1
m/z

SPC-19

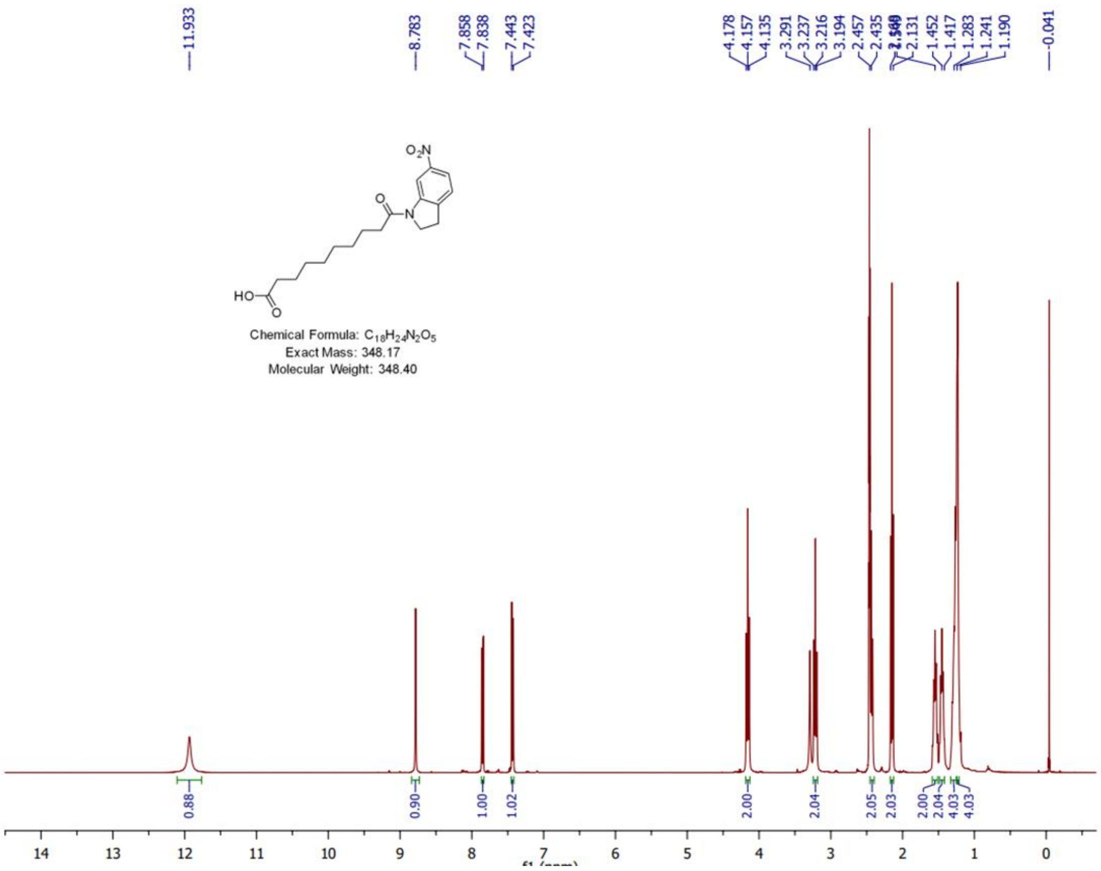

O2N
O
N
HO
O
Chemical Formula: C18H24N2O5
Exact Mass: 348.17
Molecular Weight: 348.40

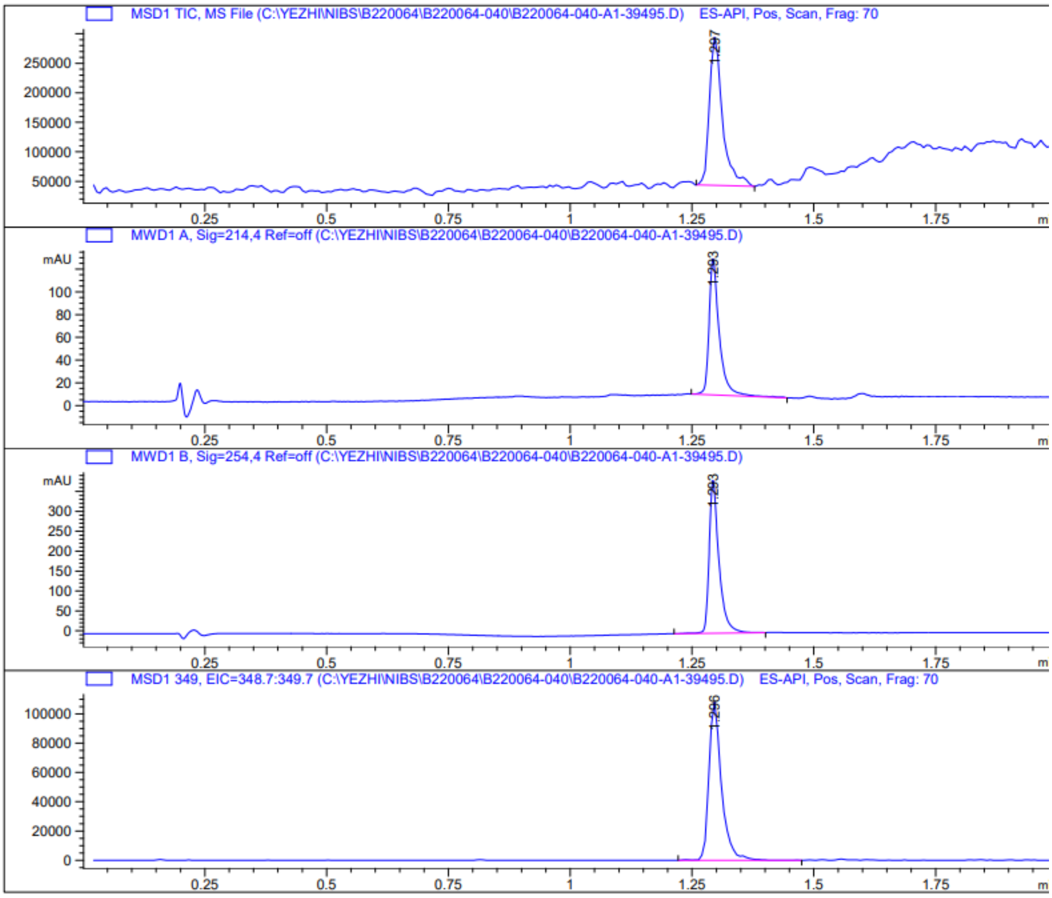

MSD1 TIC, MS File (C:\YEZHI\NIBS\B220064\B220064-040\B220064-040-A1-39495.D) ES-API, Pos, Scan, Frag: 70
MWD1 A, Sig=214,4 Ref=off (C:\YEZHI\NIBS\B220064\B220064-040\B220064-040-A1-39495.D)
MWD1 B, Sig=254,4 Ref=off (C:\YEZHI\NIBS\B220064\B220064-040\B220064-040-A1-39495.D)
MSD1 349, EIC=348.7:349.7 (C:\YEZHI\NIBS\B220064\B220064-040\B220064-040-A1-39495.D) ES-API, Pos, Scan, Frag: 70

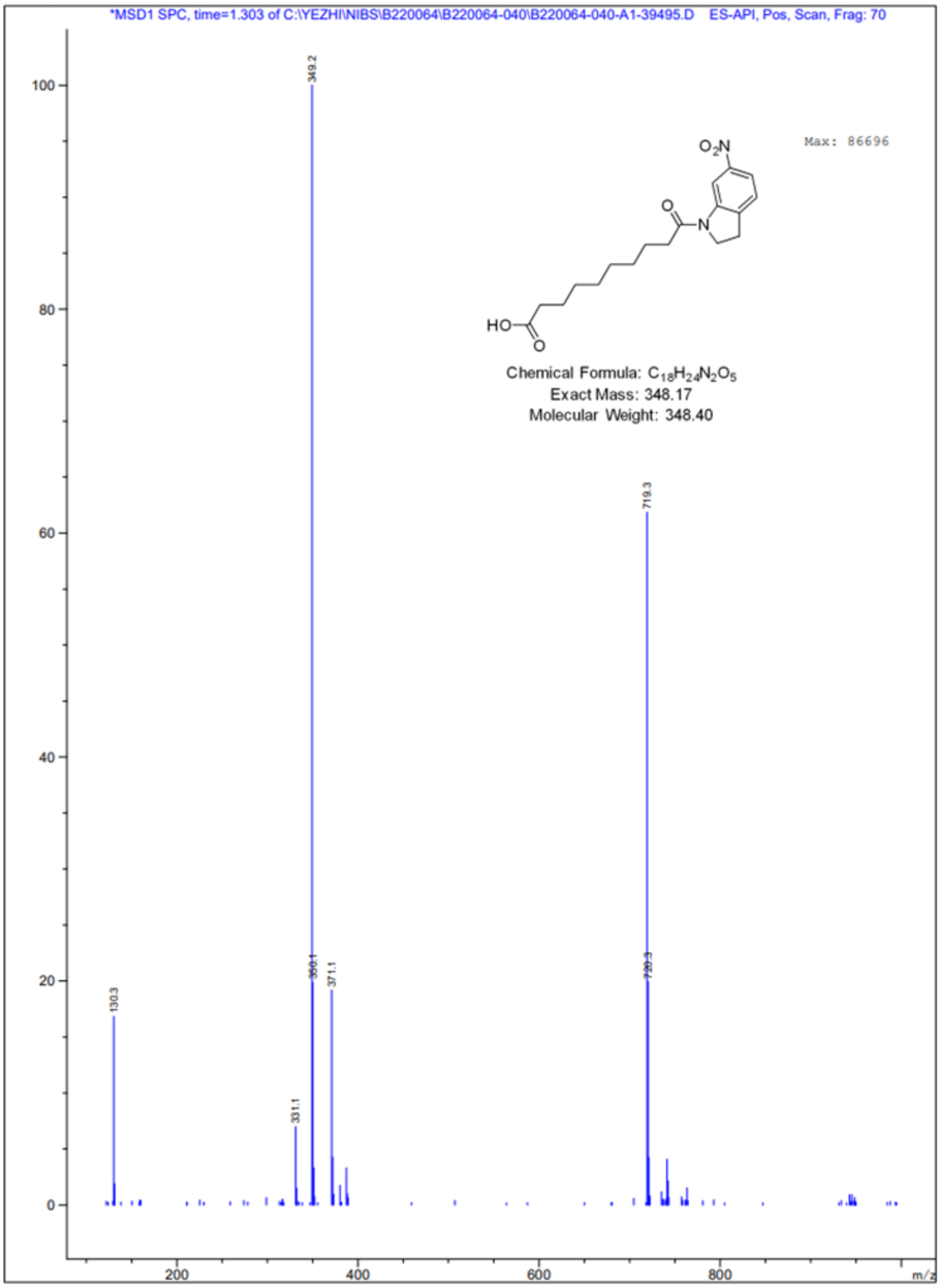
*MSD1 SPC, time=1.303 of C:\YEZHI\NIBS\B220064\B220064-040\B220064-040-A1-39495.D ES-API, Pos, Scan, Frag: 70
Max: 86696
Chemical Formula: C18H24N2O5
Exact Mass: 348.17
Molecular Weight: 348.40
349.2
719.3
350.1
371.1
720.3
130.3
331.1
m/z

SPC-20

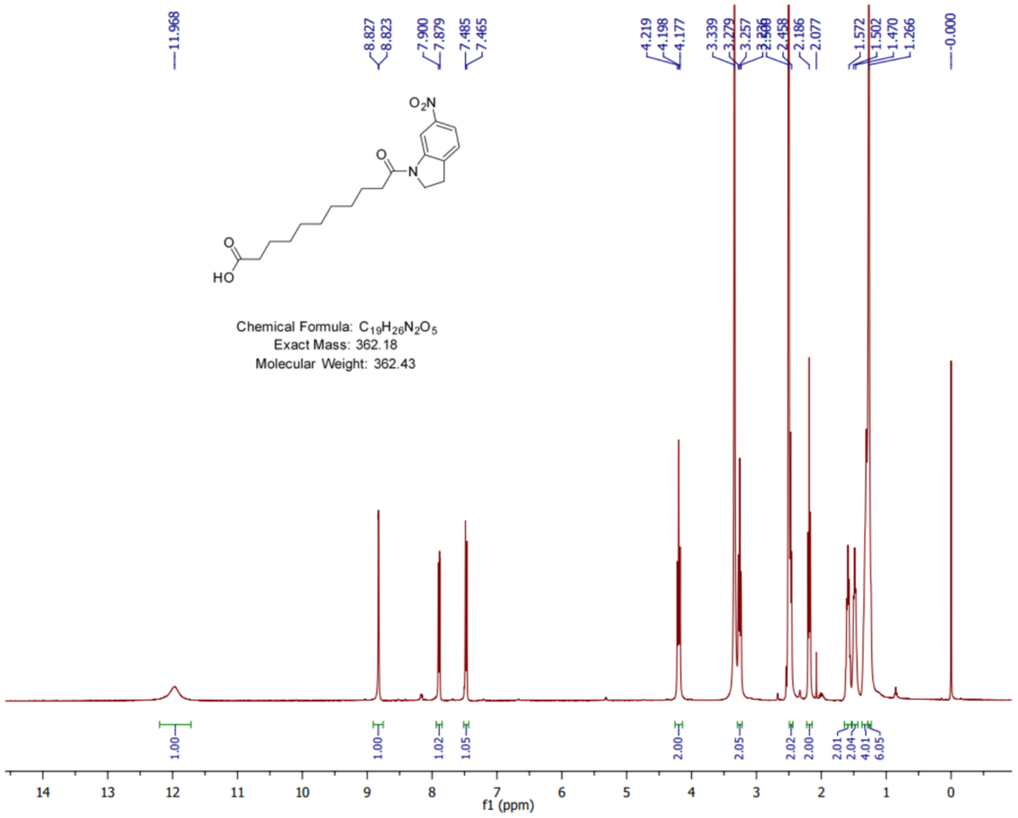

Chemical Formula: $C_{19}H_{26}N_2O_5$
Exact Mass: 362.18
Molecular Weight: 362.43
f1 (ppm)

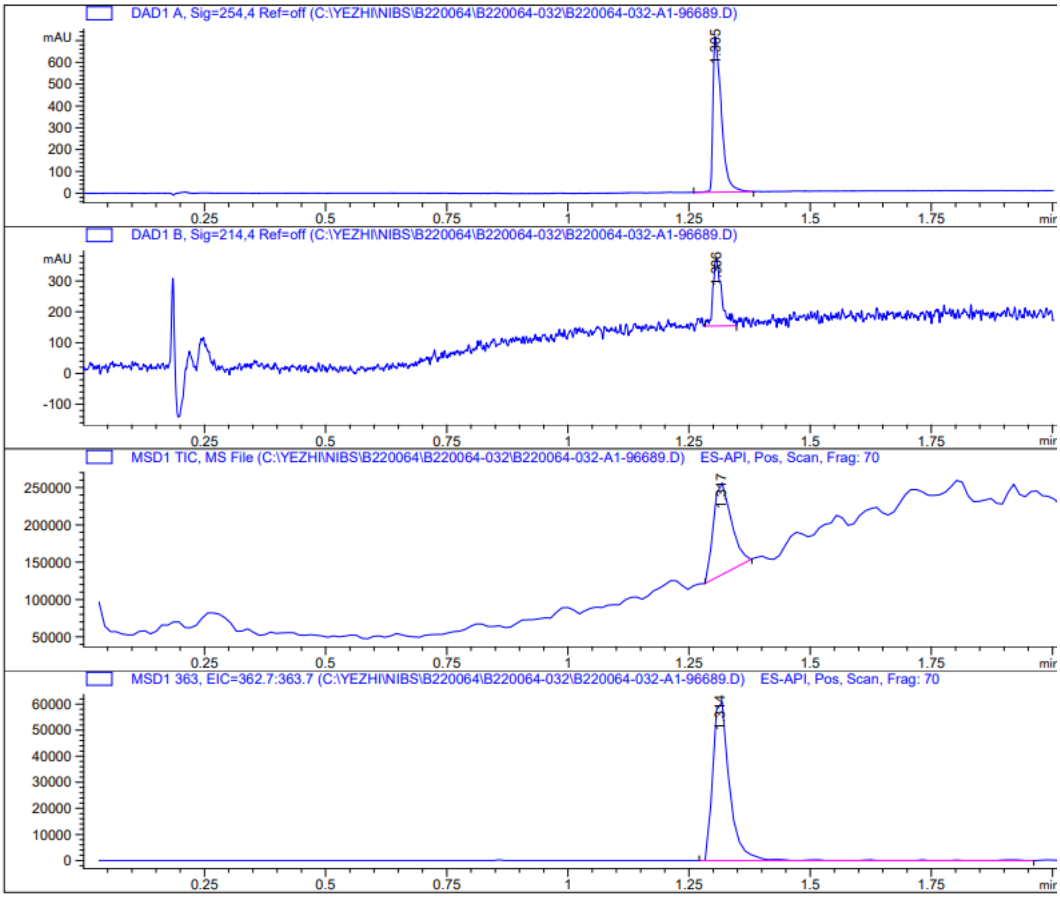

DAD1 A, Sig=254,4 Ref=off (C:\YEZHI\NIBS\B220064\B220064-032\B220064-032-A1-96689.D)
DAD1 B, Sig=214,4 Ref=off (C:\YEZHI\NIBS\B220064\B220064-032\B220064-032-A1-96689.D)
MSD1 TIC, MS File (C:\YEZHI\NIBS\B220064\B220064-032\B220064-032-A1-96689.D) ES-API, Pos, Scan, Frag: 70
MSD1 363, EIC=362.7:363.7 (C:\YEZHI\NIBS\B220064\B220064-032\B220064-032-A1-96689.D) ES-API, Pos, Scan, Frag: 70

MS Spectrum

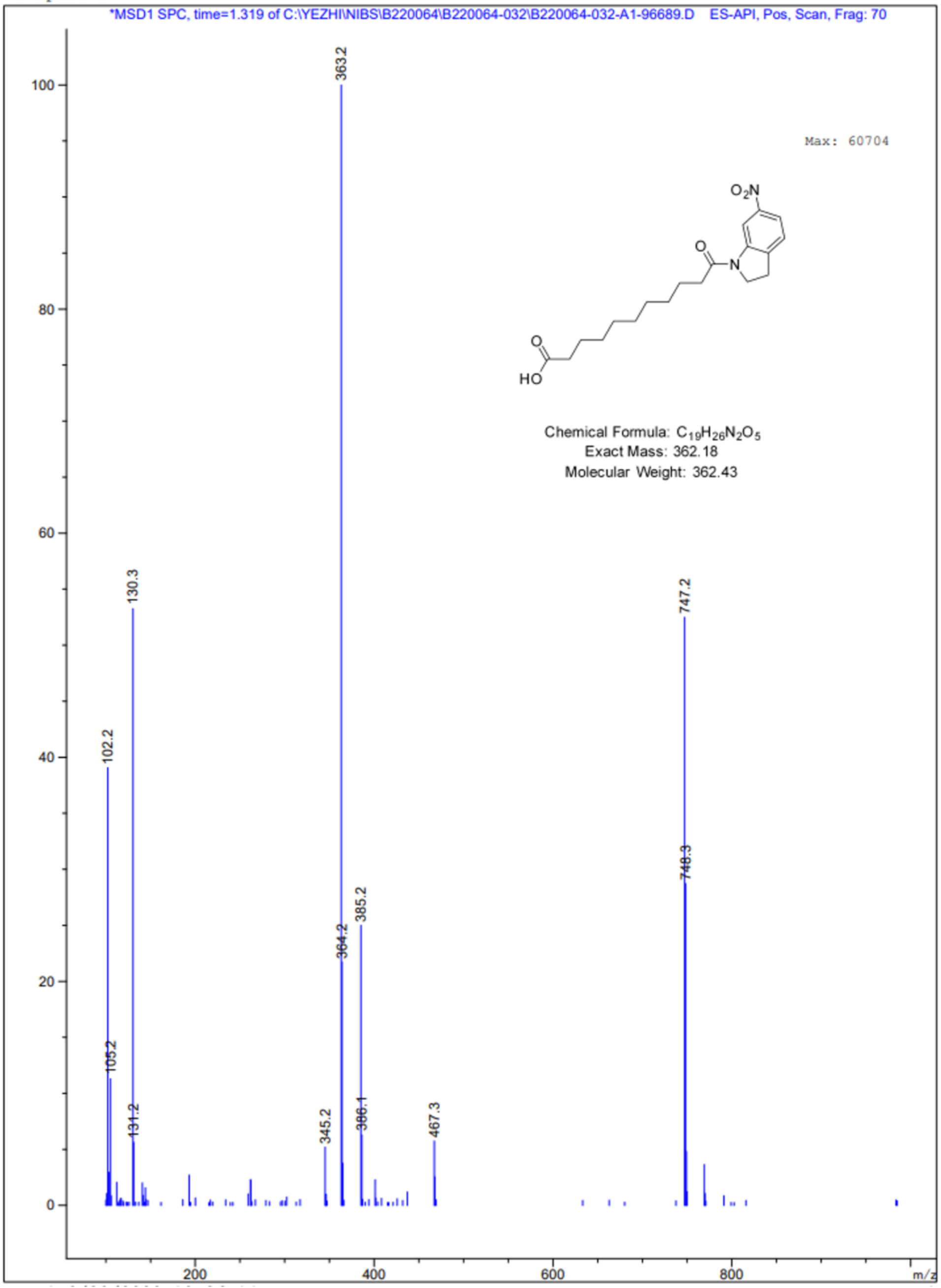

*MSD1 SPC, time=1.319 of C:\YEZHI\NIBS\B220064\B220064-032\B220064-032-A1-96689.D ES-API, Pos, Scan, Frag: 70
Max: 60704
363.2
130.3
747.2
102.2
748.3
384.2
385.2
105.2
131.2
345.2
386.1
467.3
O2N
O
N
O
HO
Chemical Formula: C19H26N2O5
Exact Mass: 362.18
Molecular Weight: 362.43
100
80
60
40
20
0
200
400
600
800
m/z

^{1}H NMR spectra and LC-MS spectra of commercially available compounds.

SPC-1 (Z25218156, Enamine)

```
MaxPeak:  100.00%
Ret_Time: 1.360 min
```

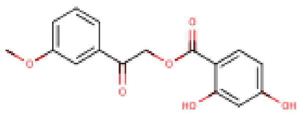

Mol Wt 302.28

Exact Mass 302.08

```
#  Time   Area%
-----------------
1  1.360  100.00
```

RT 1.377

RT 1.380

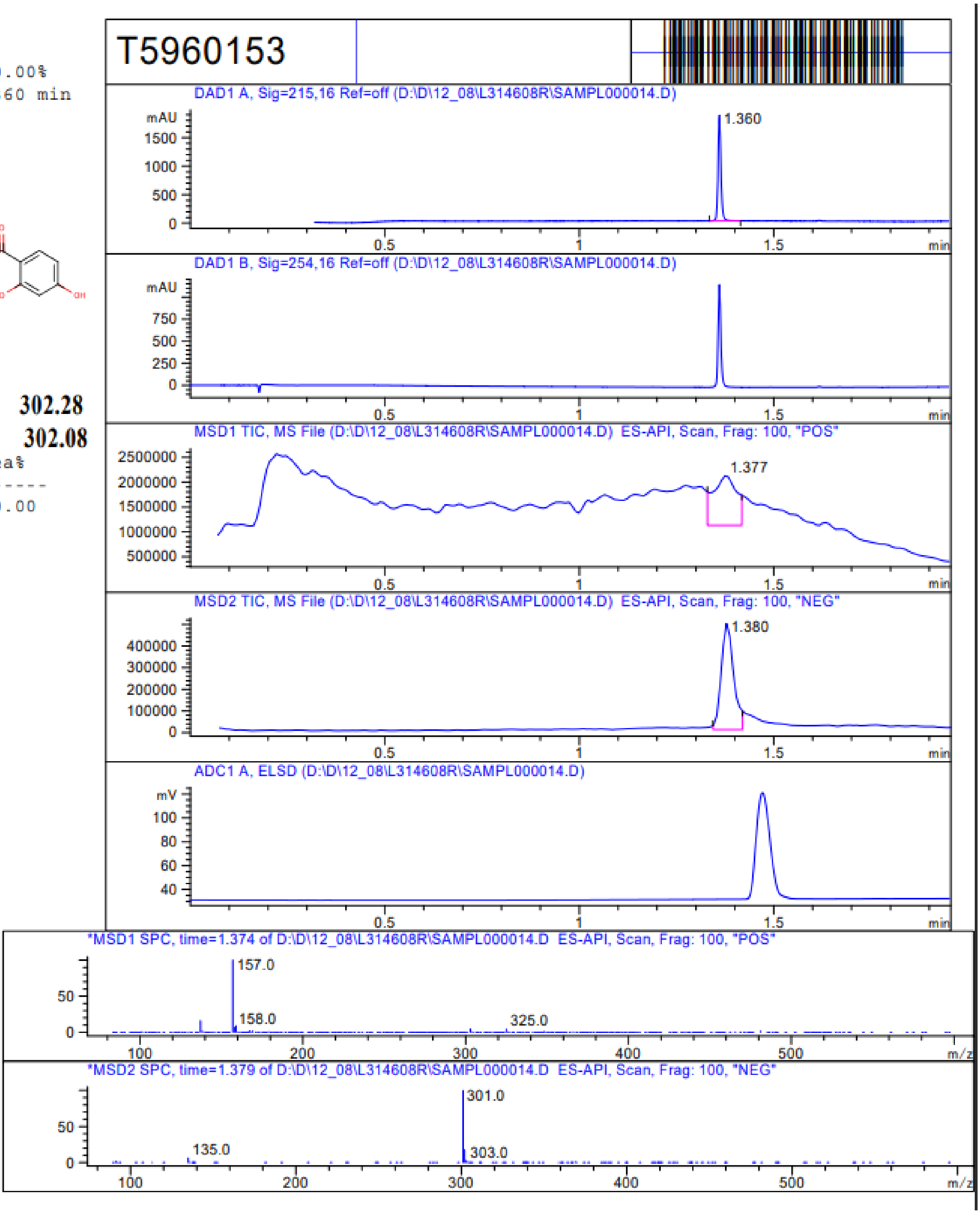

SPC-2 (Z356729938, Enamine)

MaxPeak: 98.70%
Ret_Time: 1.199 min

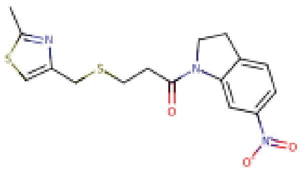

Mol Wt 363.45
Exact Mass 363.08

#	Time	Area%
1	0.996	1.30
2	1.199	98.70

RT 1.208

RT 1.208

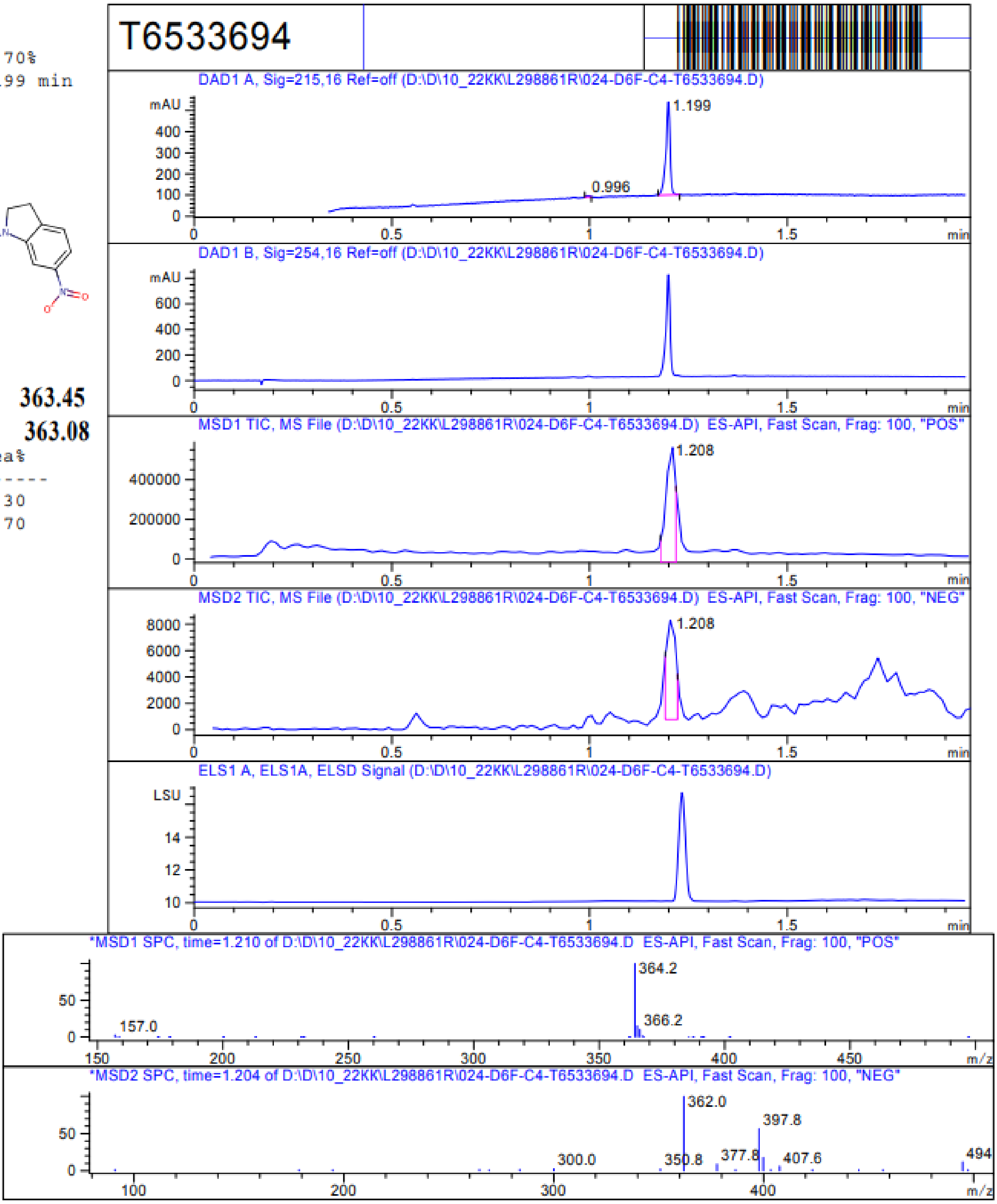

SPC-3 (Z92036426, Enamine)

MaxPeak: 100.00%
Ret_Time: 1.411 min

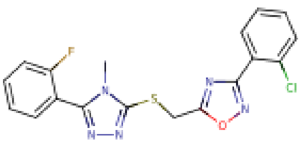

Mol Wt 401.84

Exact Mass 401.06

#	Time	Area%
1	1.411	100.00

RT 1.428

RT 1.435

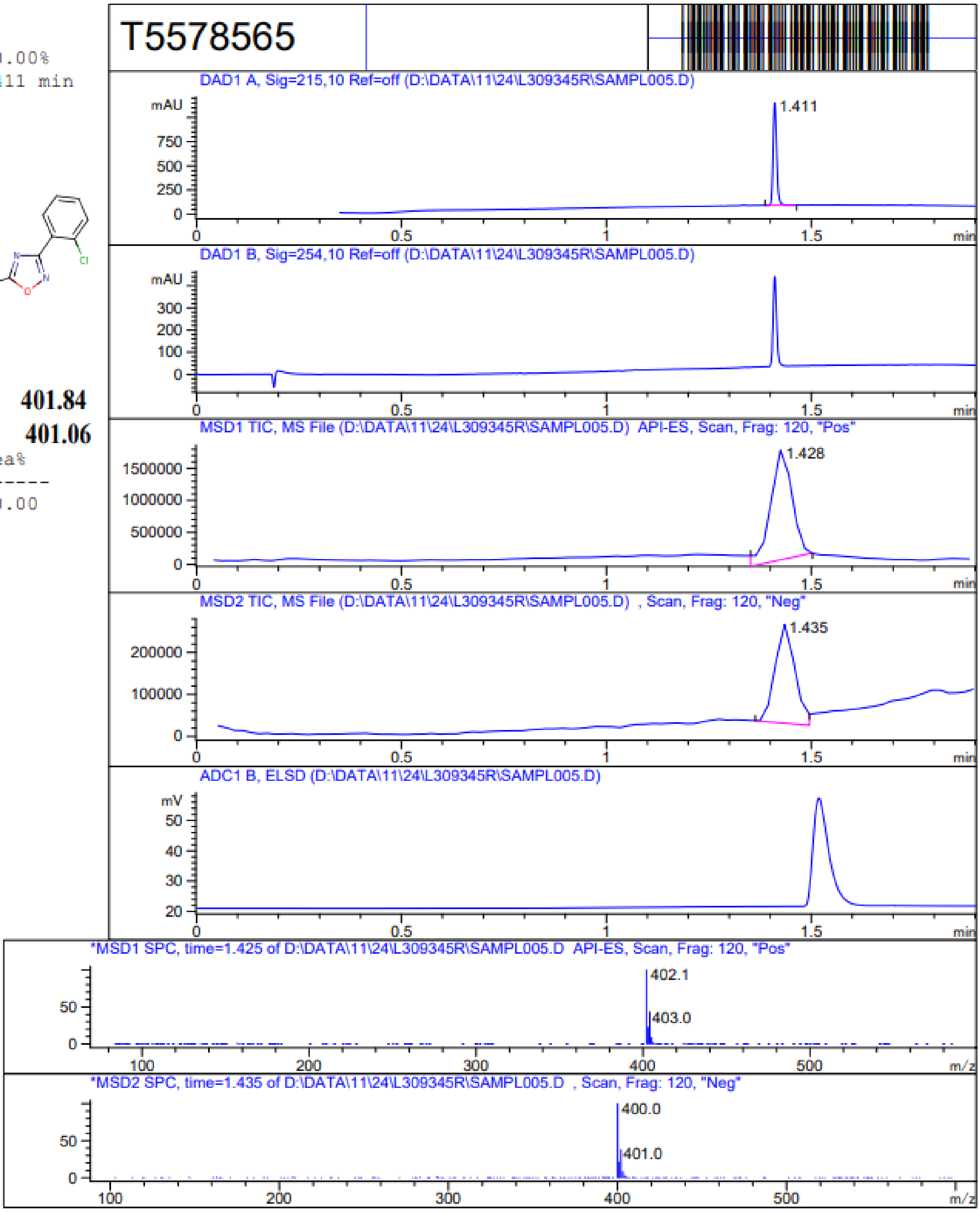

SPC-4 (23835750, ChemBridge)

FC941028234

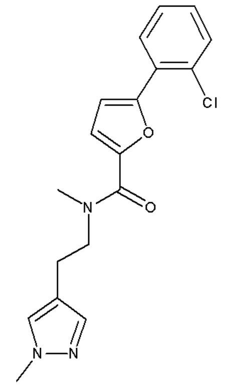

ID	23835750	343.8161	$C_{18}H_{18}ClN_3O_2$

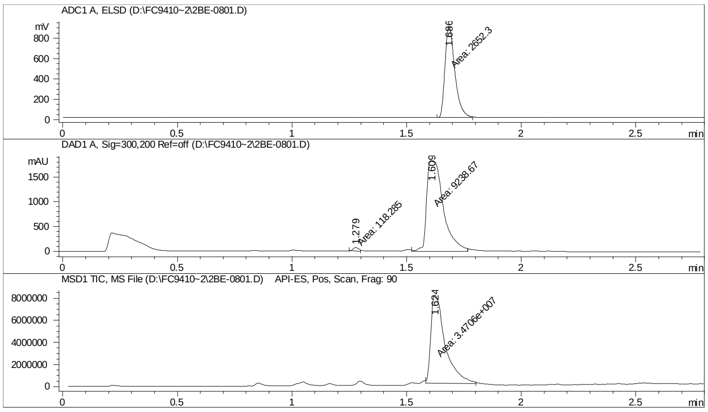

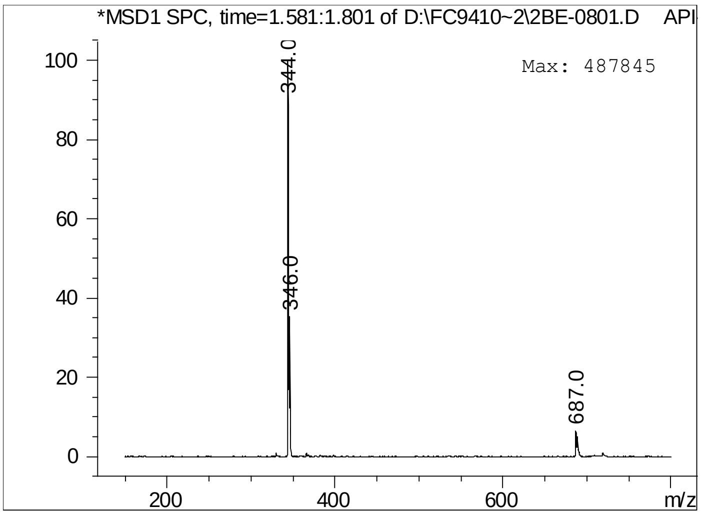

SPC-5 (7966009, ChemBridge)

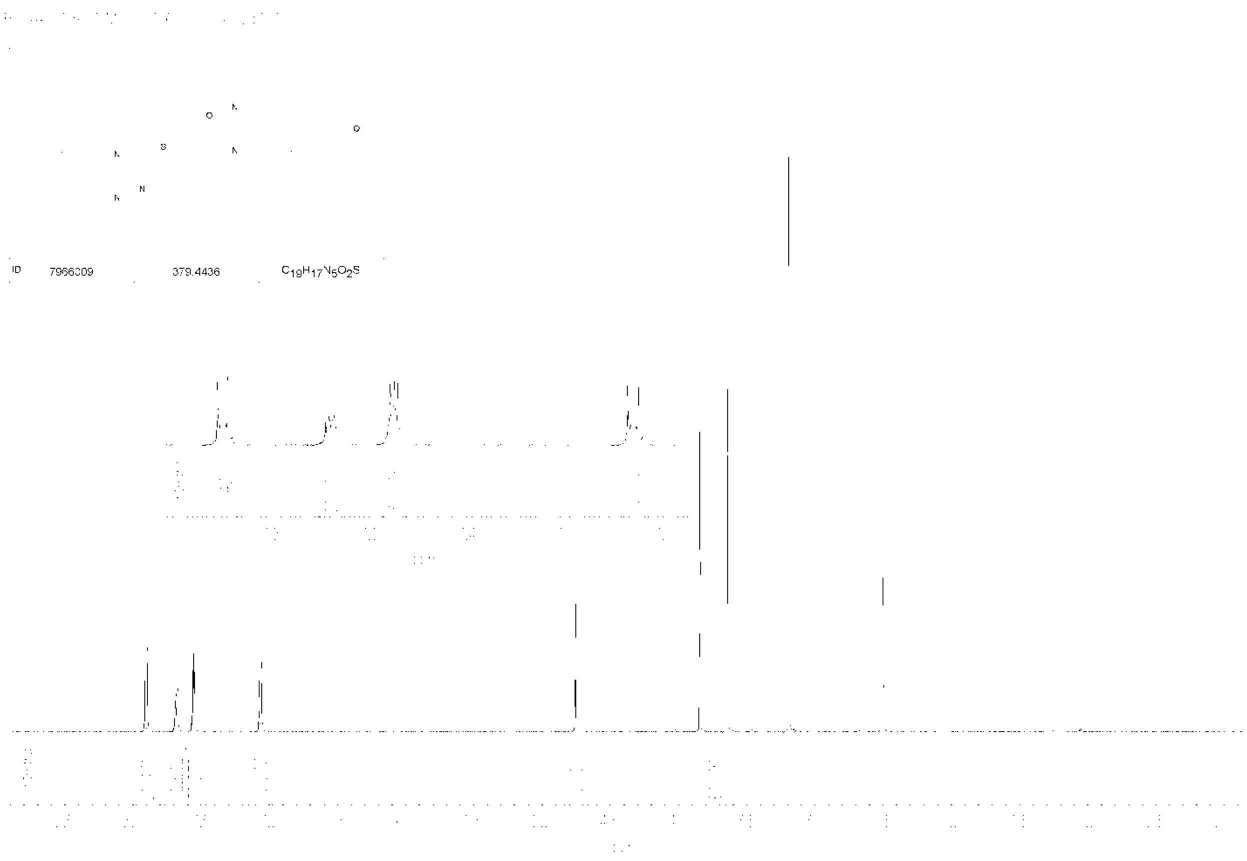

SPC-6 (STK861013, Vitas-M)

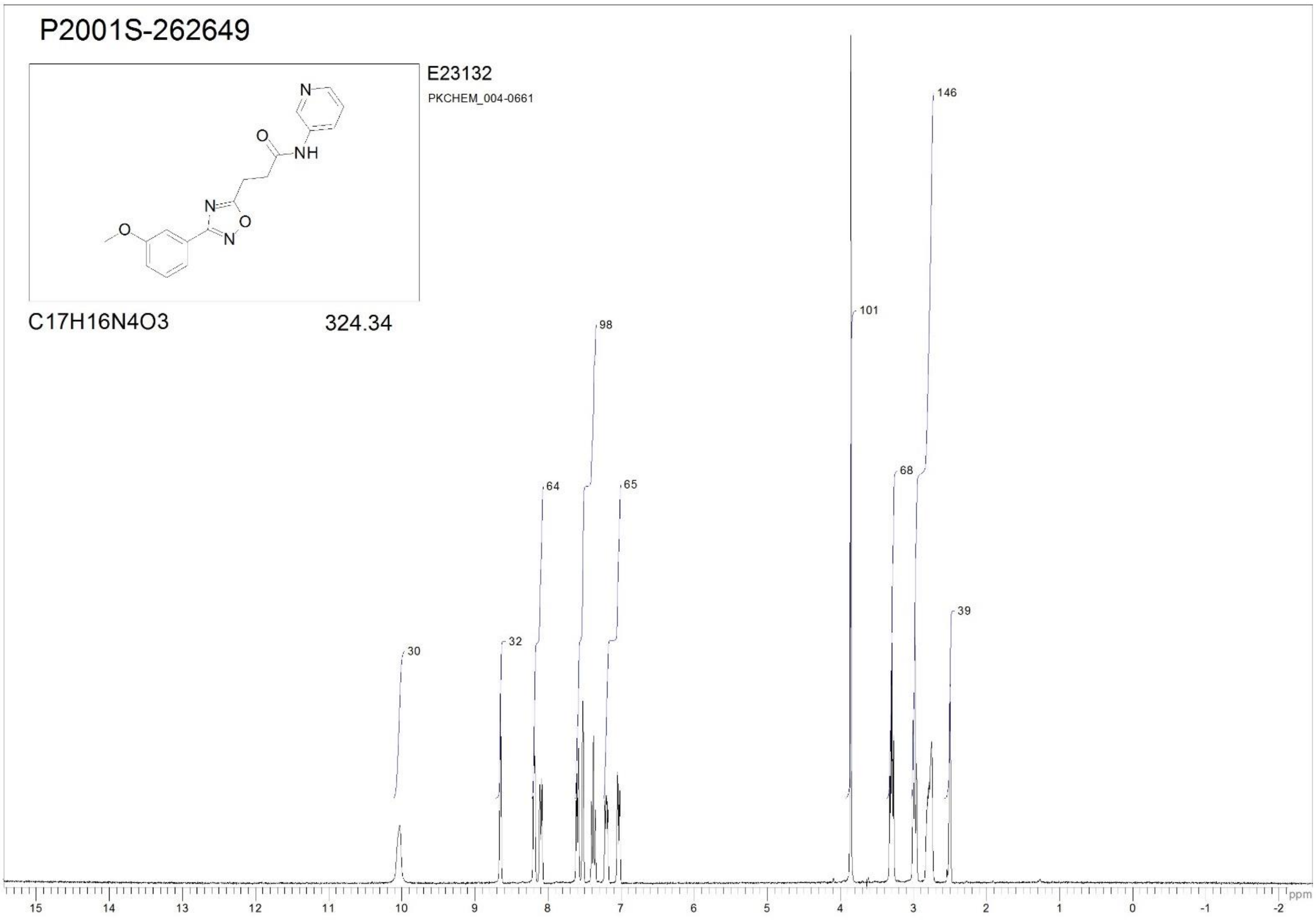

SPC-7 (Z226758636, Enamine)

MaxPeak: 100.00%
Ret_Time: 1.280 min

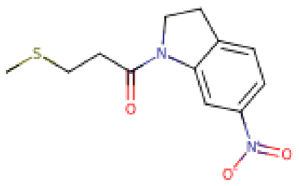

Mol Wt 266.32
Exact Mass 266.08

#	Time	Area%
1	1.280	100.00

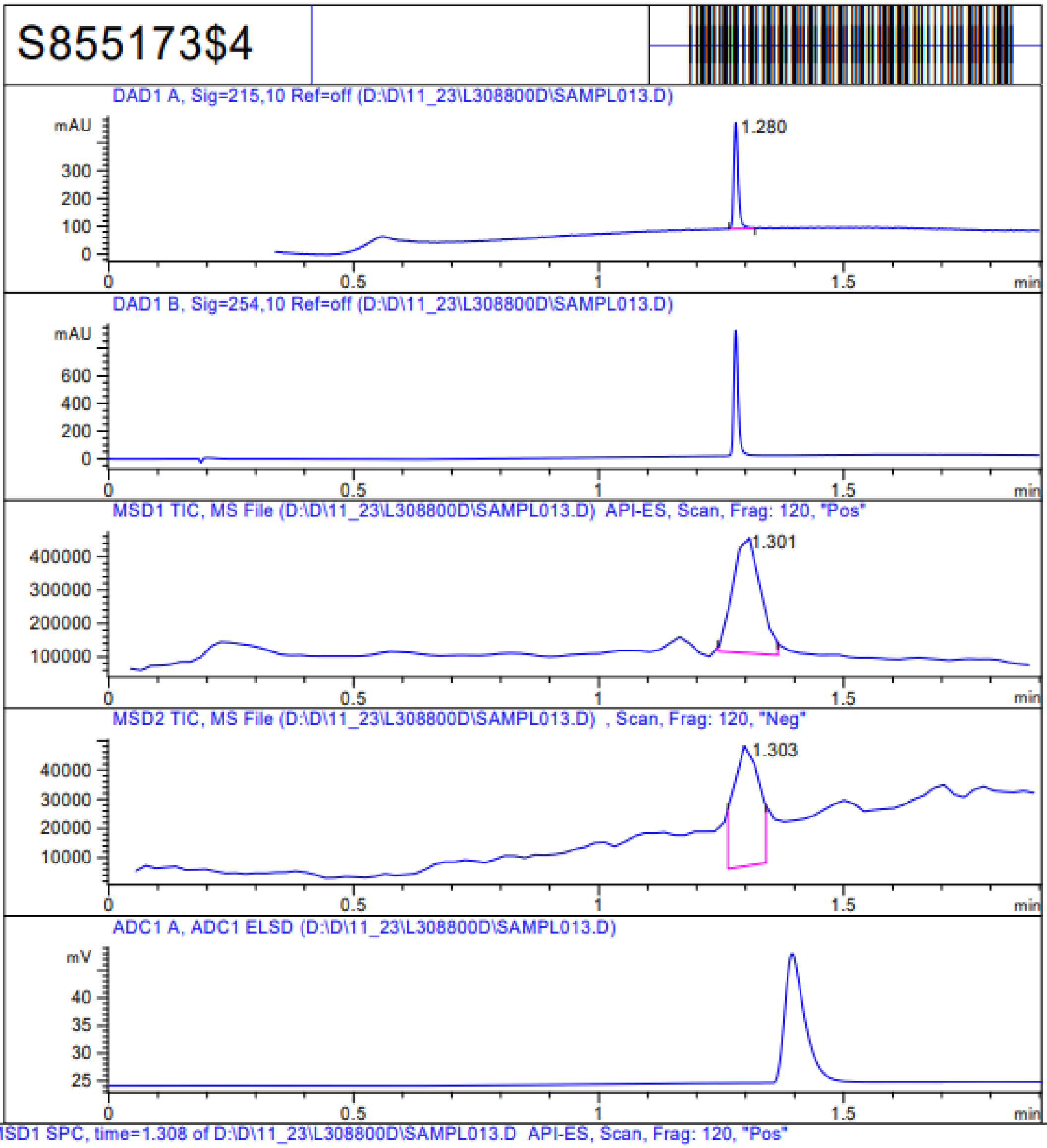

RT 1.301

RT 1.303

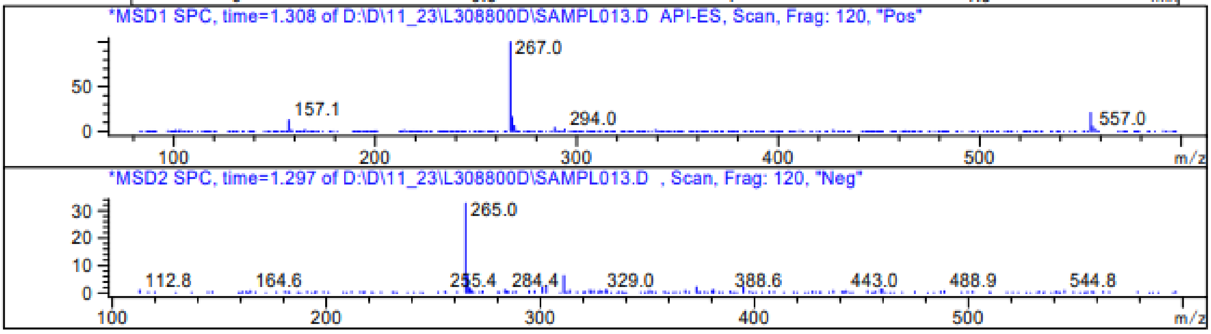

S855173$4

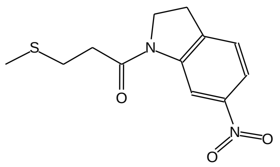

S855173$4 C12H14N2O3S 266.31

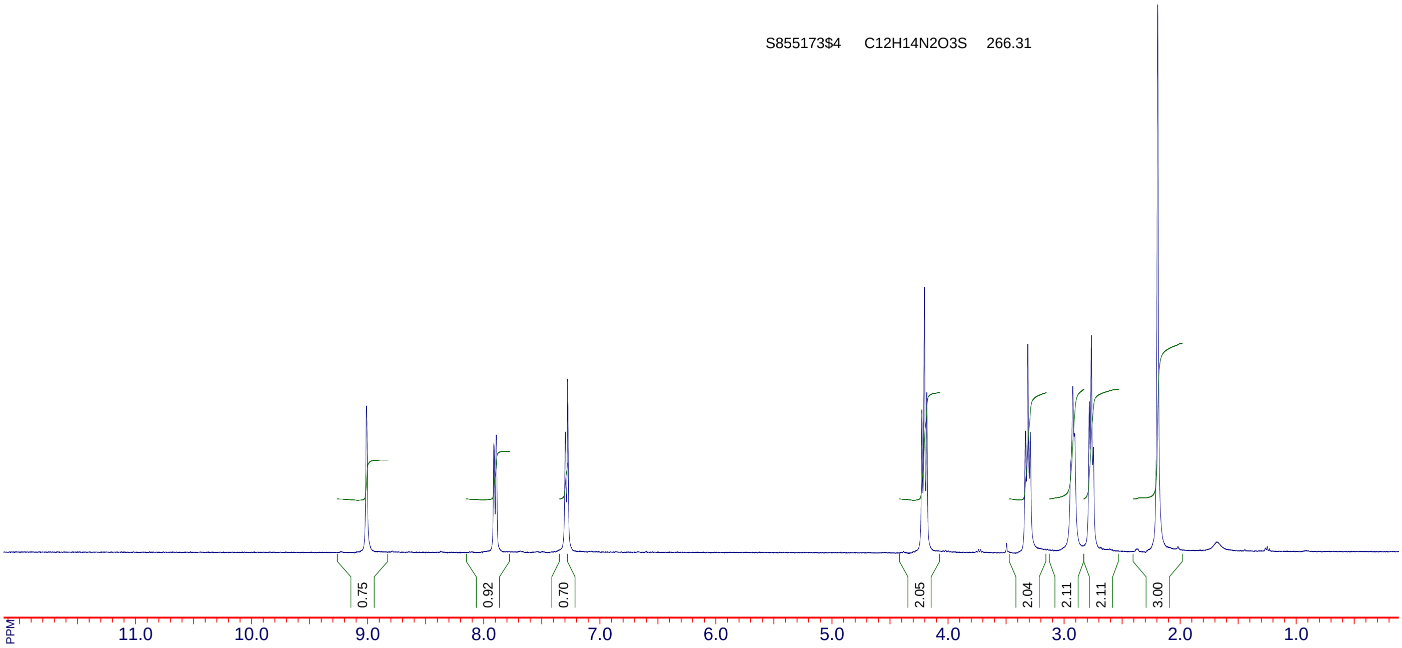

File name: S855173$4	Honchar	SF: 399.9736 MHz	NSC: 0	PW: 11.60 usec, RG: 40	SI: 65536
Date: 23-Nov-2020	Solvent: cdcl3	SW: 7599 Hz	TE: 294 K	AQ: 2.11 sec, RD: 0.00 sec	S855173$4

SPC-8 (Z1231090949, Enamine)

MaxPeak: 100.00%
Ret_Time: 1.463 min

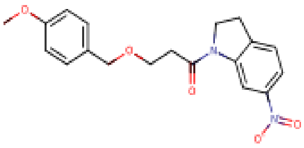

Mol Wt 356.37
Exact Mass 356.15

#	Time	Area%
1	1.463	100.00

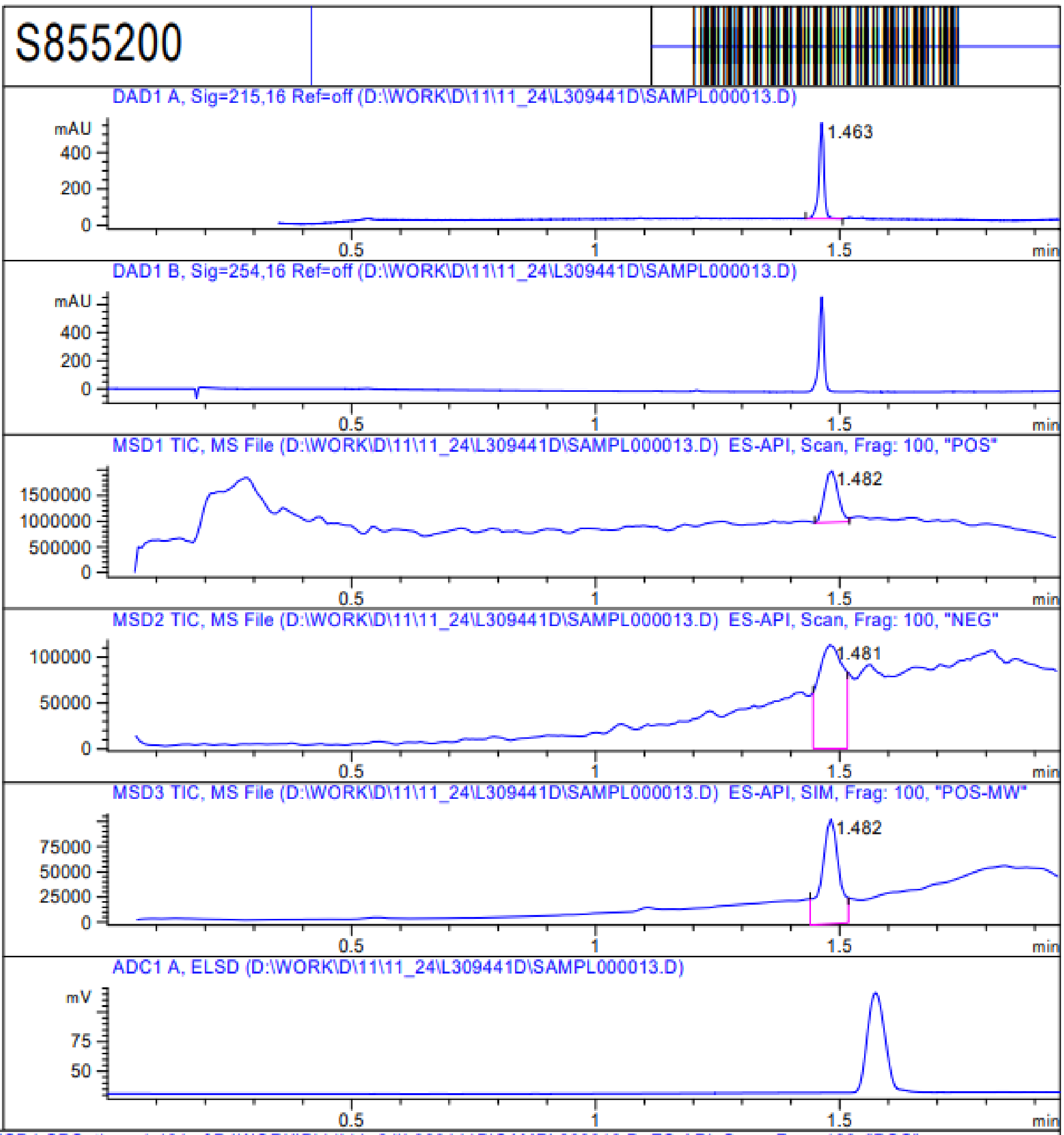

RT 1.482

RT 1.481

RT 1.482

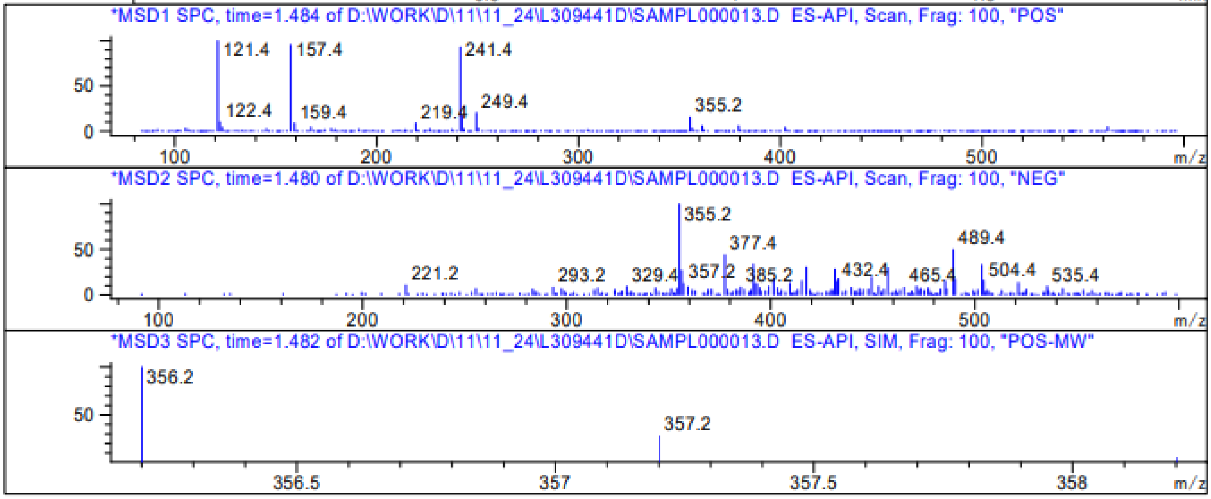

S855200

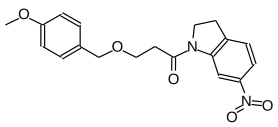

S855200 C19H20N2O5 356.38

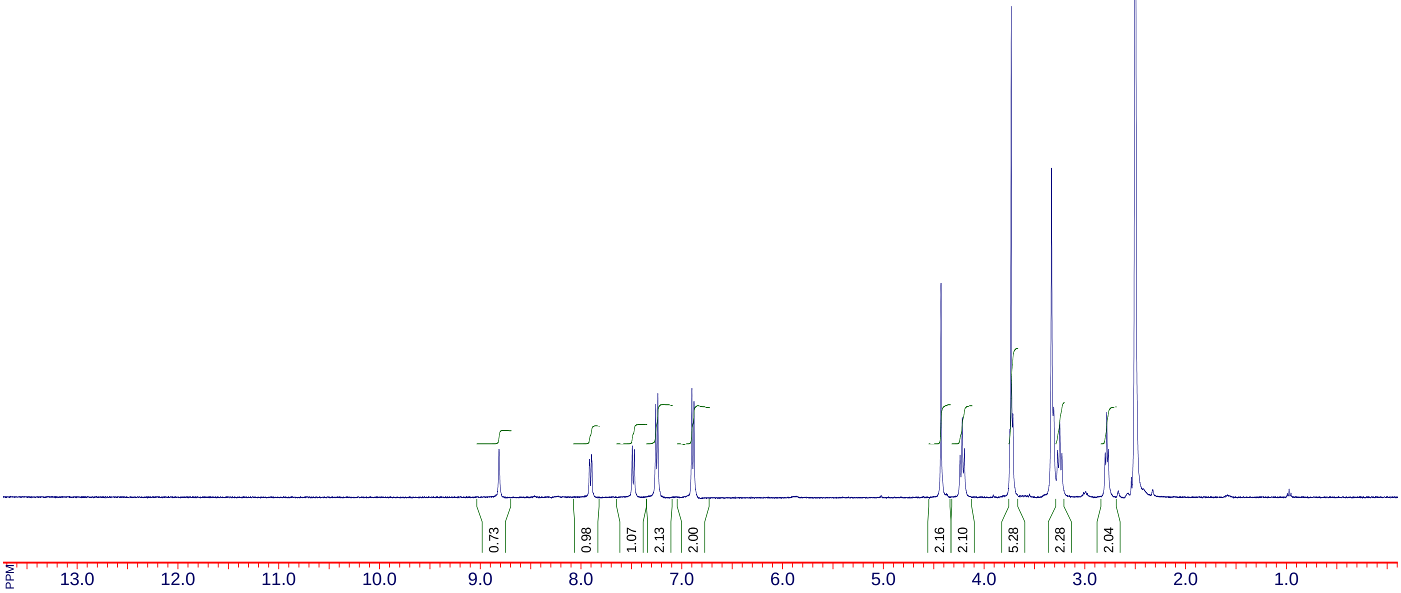

**File name: S855200**	Honchar	SF: 399.9755 MHz	NSC: 0	PW: 11.60 usec, RG: 40	SI: 65536
Date: 16-Nov-2020	Solvent: dmso	SW: 7599 Hz	TE: 294 K	AQ: 2.11 sec, RD: 0.00 sec	S855200

SPC-9 (Z2146217256, Enamine)

MaxPeak: 100.00%
Ret_Time: 1.252 min

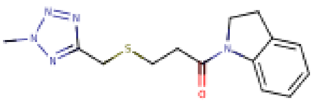

Mol Wt 303.38

Exact Mass 303.13

#	Time	Area%
1	1.252	100.00

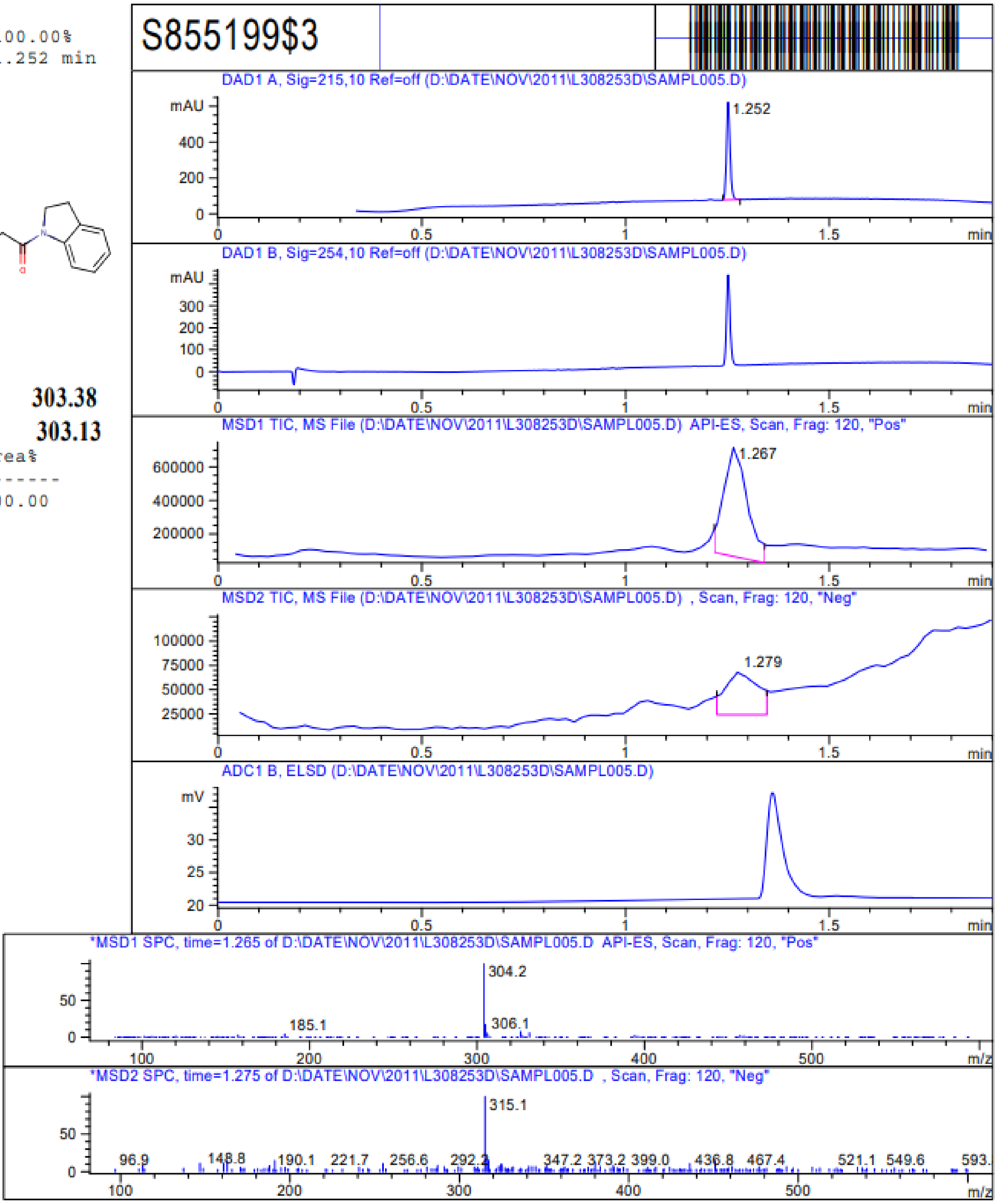

RT 1.267

RT 1.279

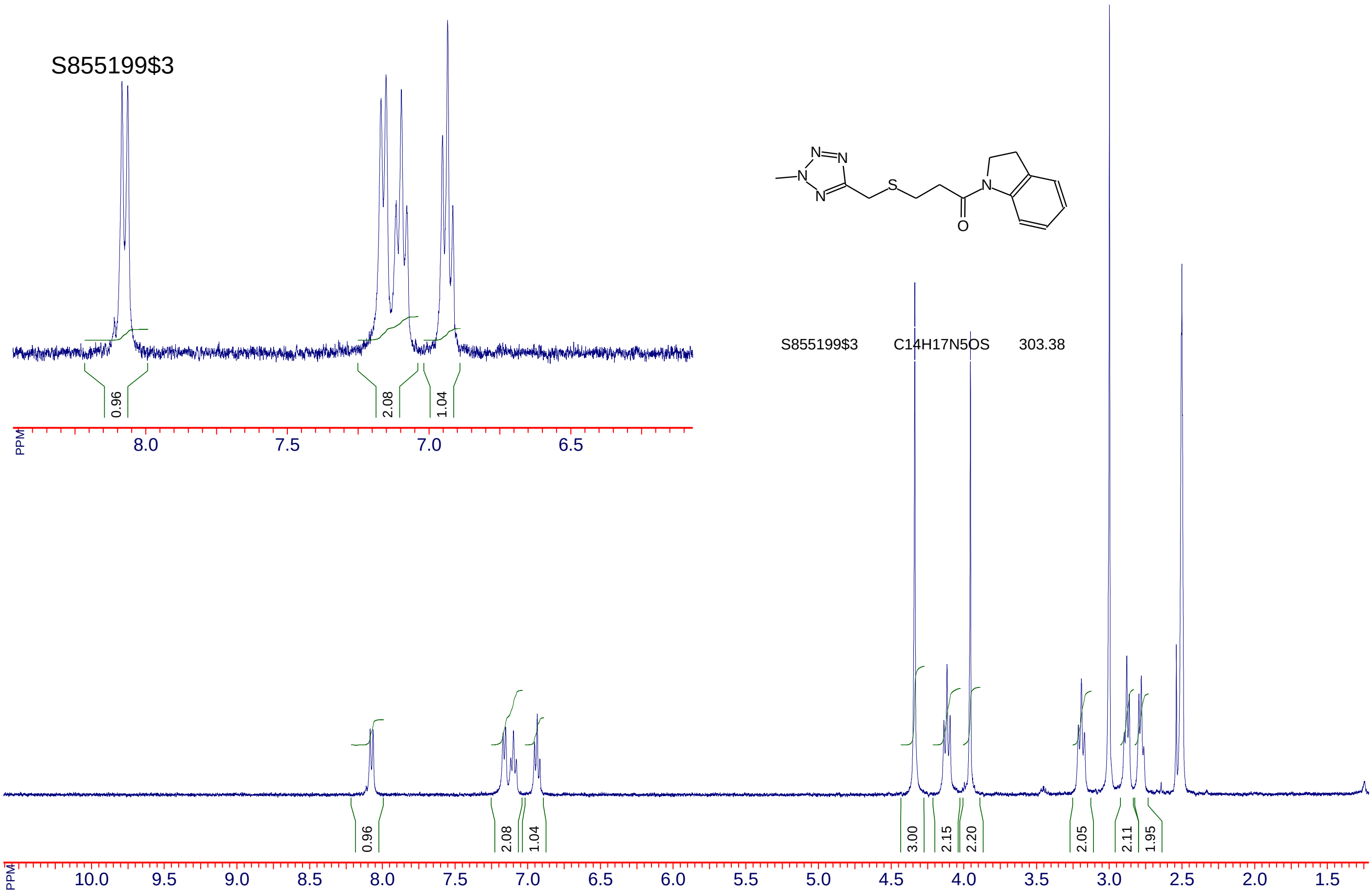

**File name: S855199$3**	Operator: Victorova	SF: 399.9755 MHz	NSC: 0	PW: 11.60 usec, RG: 34	SI: 65536
Date: 23-Nov-2020	Solvent: dmso+ccl4	SW: 7599 Hz	TE: 294 K	AQ: 2.11 sec, RD: 0.00 sec	

SPC-10 (Z2179428345, Enamine)

MaxPeak: 98.04%
Ret_Time: 1.282 min

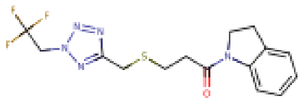

Mol Wt 371.38
Exact Mass 371.12

#	Time	Area%
1	1.282	98.04
2	1.382	1.96

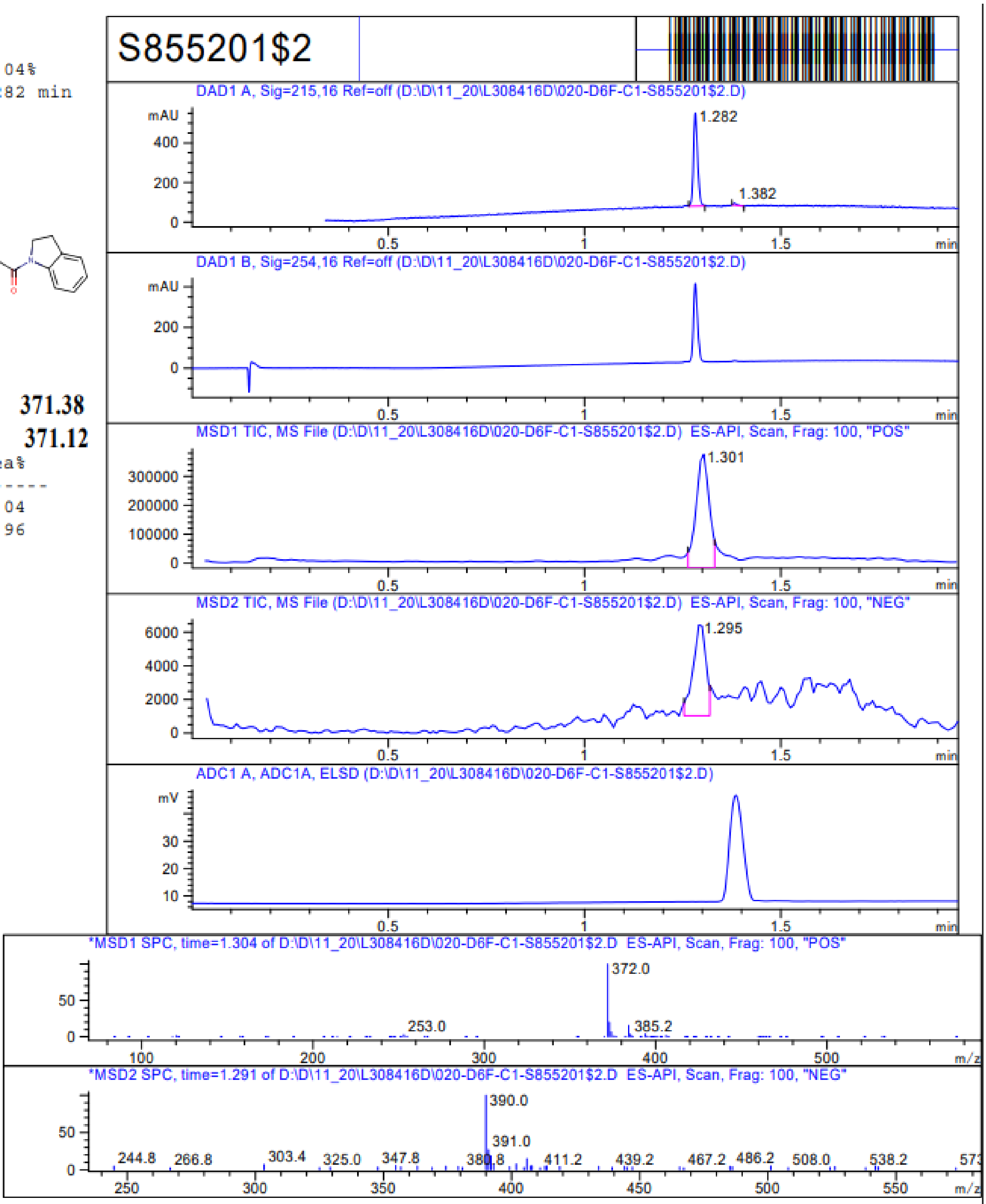

RT 1.301

RT 1.295

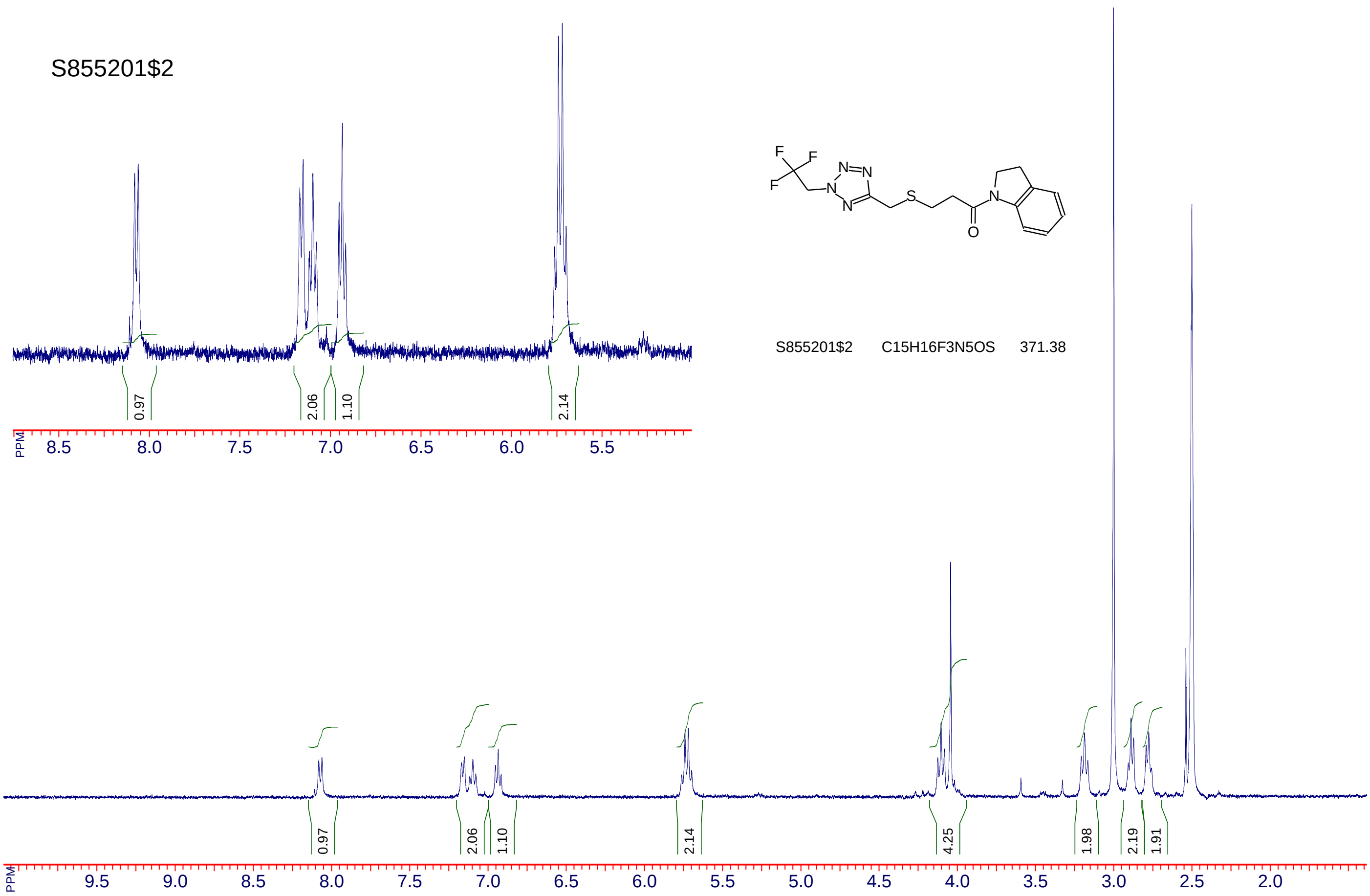

**File name: S855201$2**	Operator: Victorova	SF: 399.9755 MHz	NSC: 0	PW: 11.60 usec, RG: 34	SI: 65536
Date: 23-Nov-2020	Solvent: dmso+ccl4	SW: 7599 Hz	TE: 294 K	AQ: 2.11 sec, RD: 0.00 sec	

SPC-11 (Z2366227978, Enamine)

MaxPeak: 90.02%
Ret_Time: 1.405 min

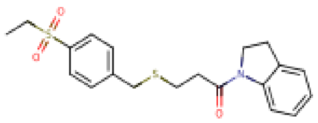

Mol Wt 389.53
Exact Mass 389.14

#	Time	Area%
1	1.290	2.90
2	1.375	7.08
3	1.405	90.02

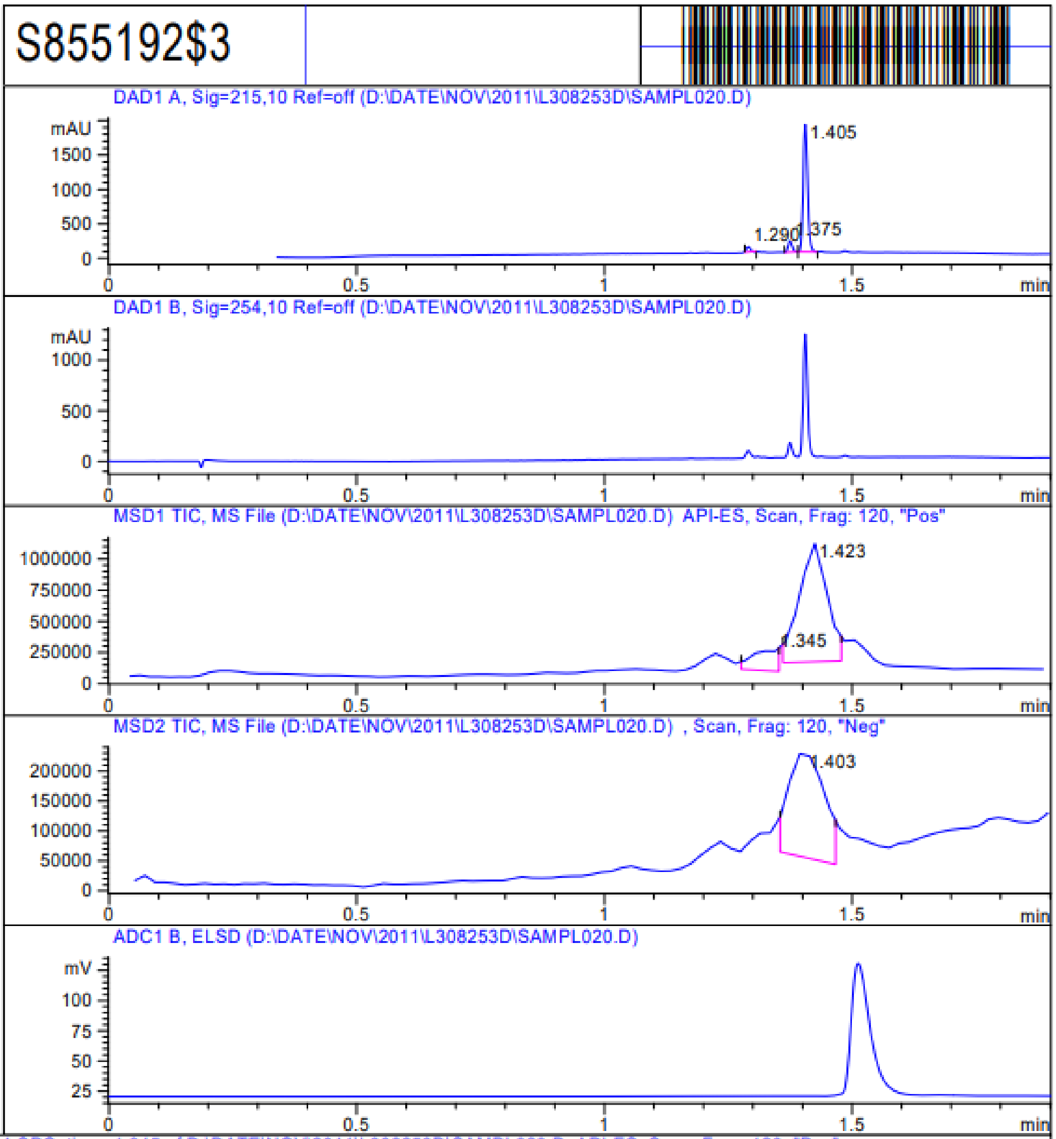

RT 1.345

RT 1.423

RT 1.403

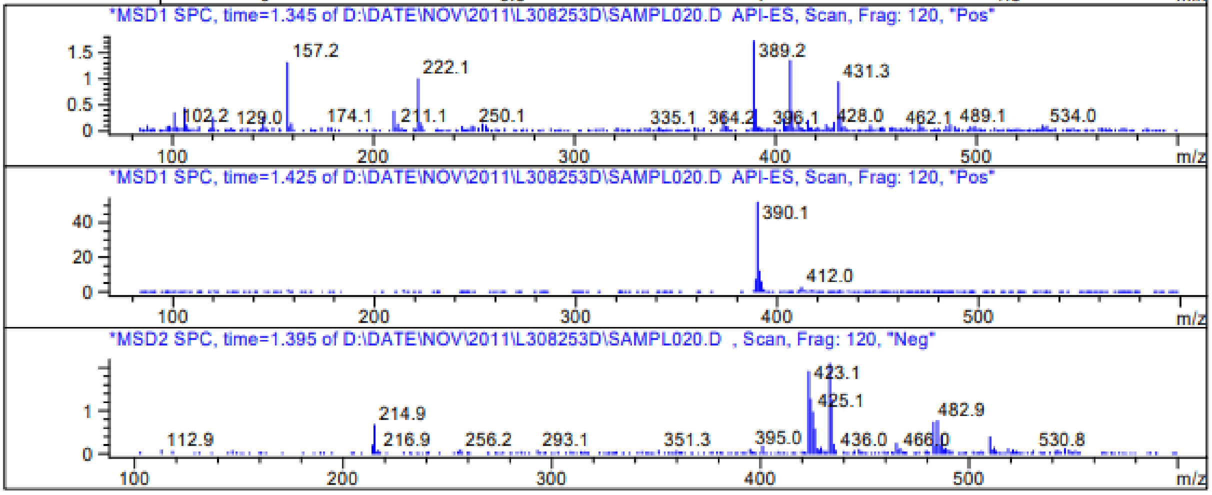

H2764240

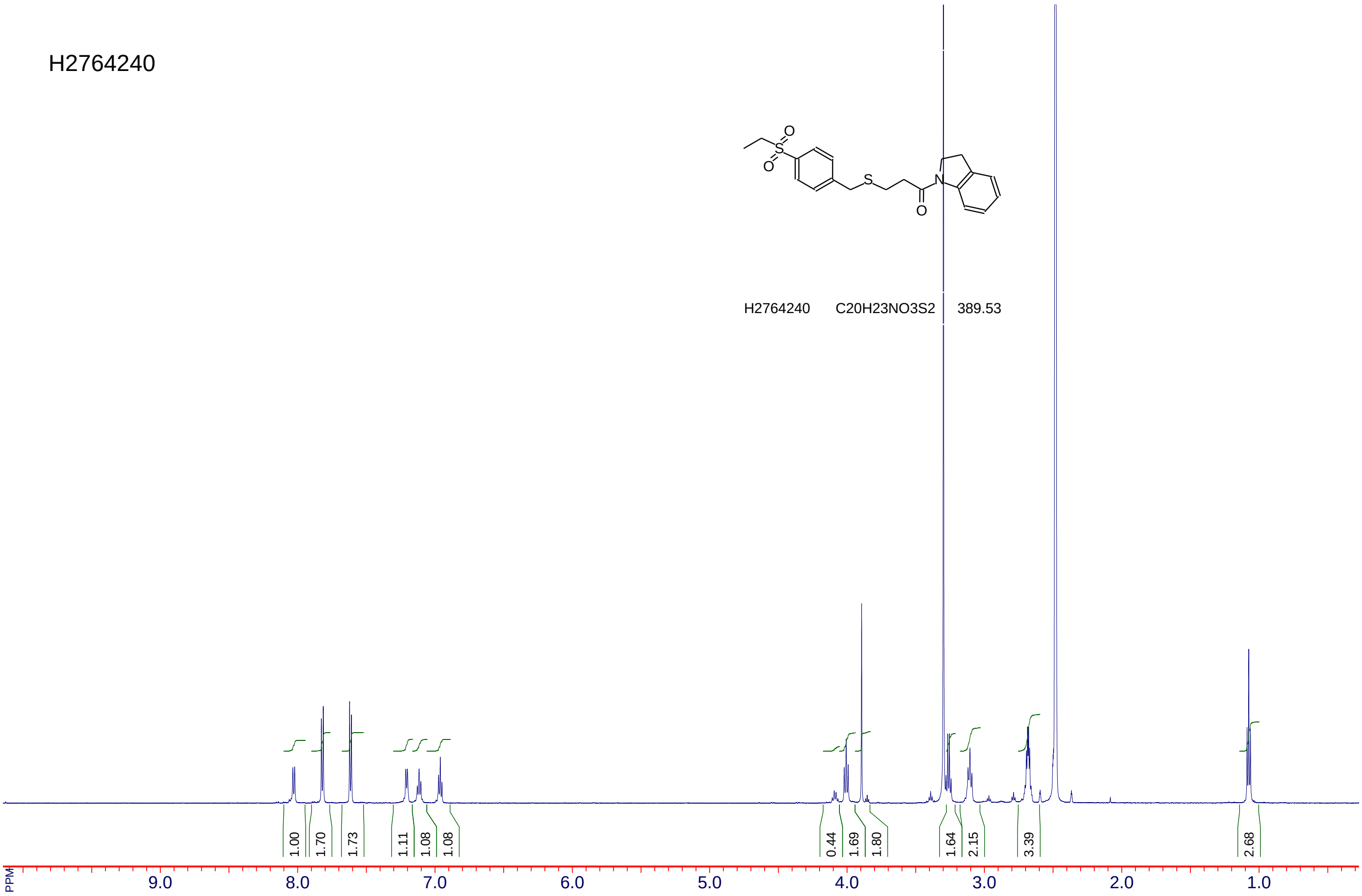

File name: H2764240	Honchar	SF: 599.7811 MHz	NSC: 0	PW: 4.40 usec, RG: 46	SI: 131072
Date: 04-Dec-2020	Solvent: dmso	SW: 11364 Hz	TE: 298 K	AQ: 2.82 sec, RD: 0.00 sec	H2764240

SPC-12 (Z327257512, Enamine)

MaxPeak: 100.00%
Ret_Time: 1.349 min

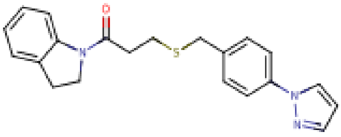

Mol Wt 363.48
Exact Mass 363.17

#	Time	Area%
1	1.349	100.00

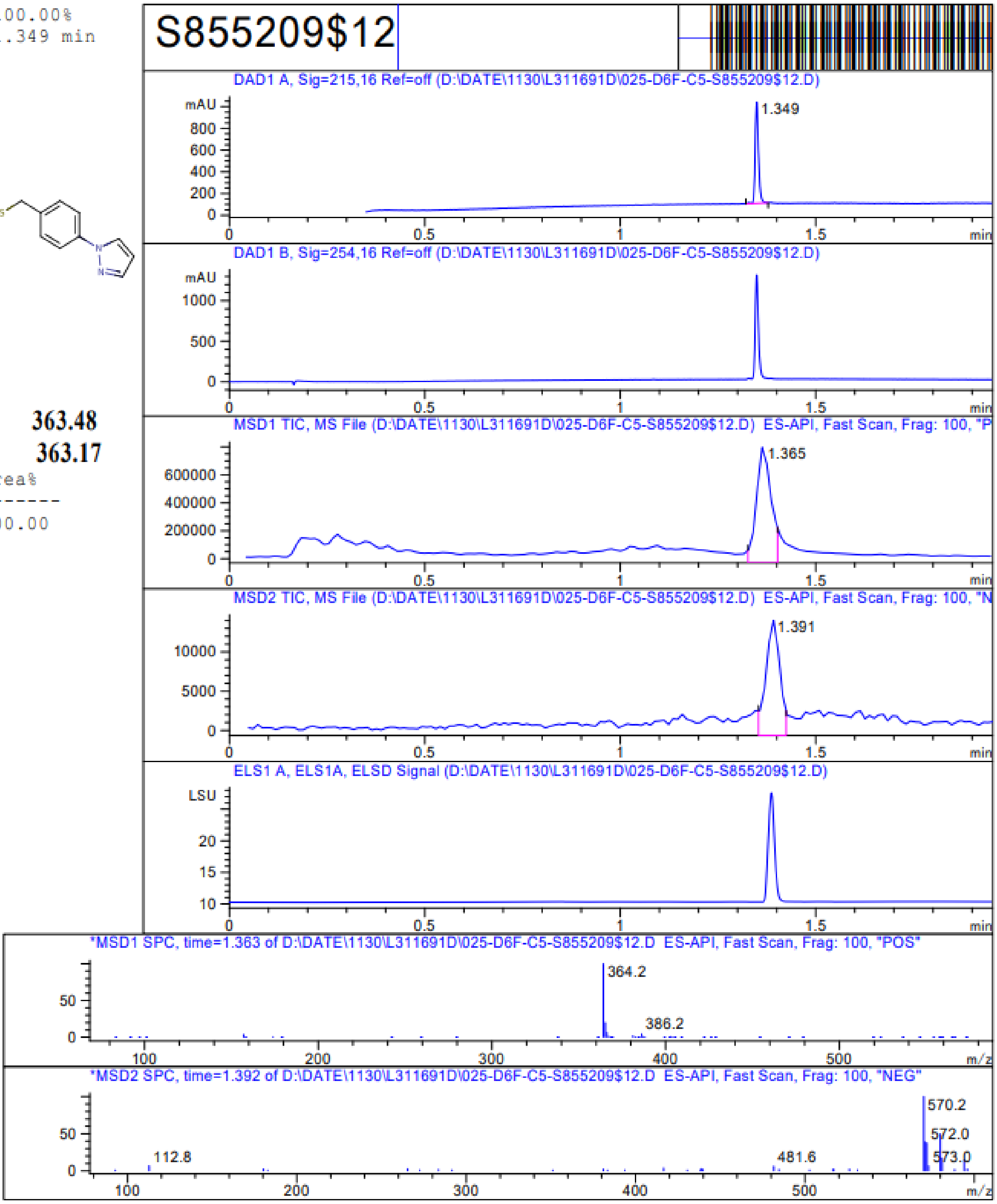

RT 1.365

RT 1.391

S855209$12

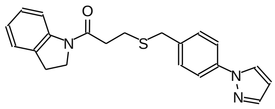

S855209$12 C21H21N3OS 363.48

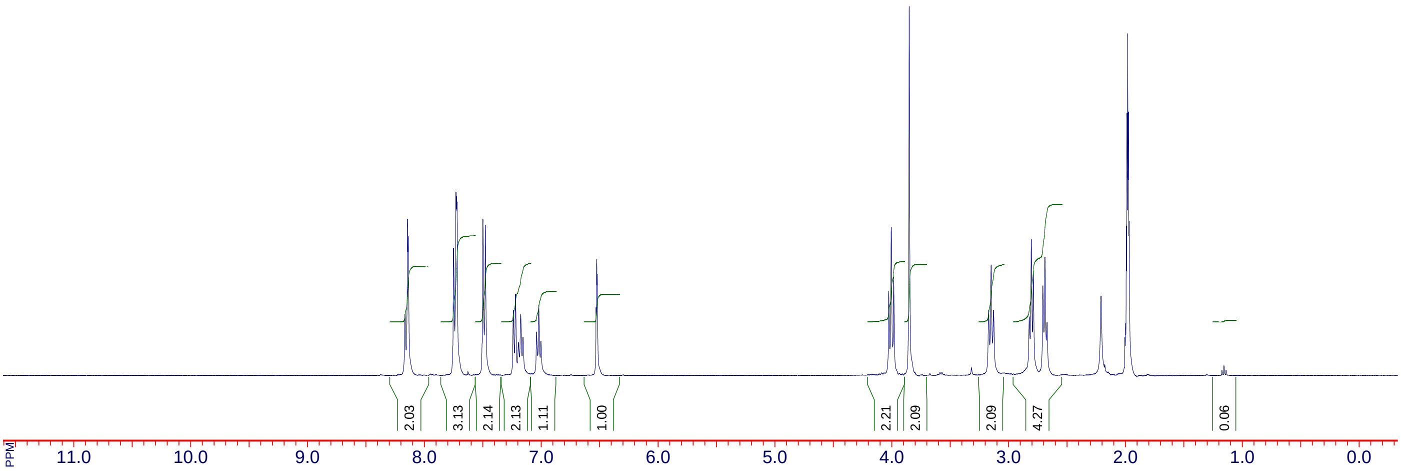

**File name: S855209$12**	Honchar	SF: 399.9757 MHz	NSC: 0	PW: 11.60 usec, RG: 40	SI: 65536
Date: 01-Dec-2020	Solvent: CD3CN	SW: 7599 Hz	TE: 294 K	AQ: 2.11 sec, RD: 0.00 sec	S855209$12

SPC-13 (Z785702604, Enamine)

```
MaxPeak:  100.00%
Ret_Time: 1.501 min
```

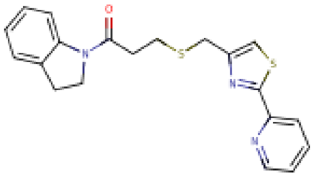

Mol Wt 381.51

Exact Mass 381.12

#	Time	Area%
1	1.501	100.00

RT 1.526

RT 1.527

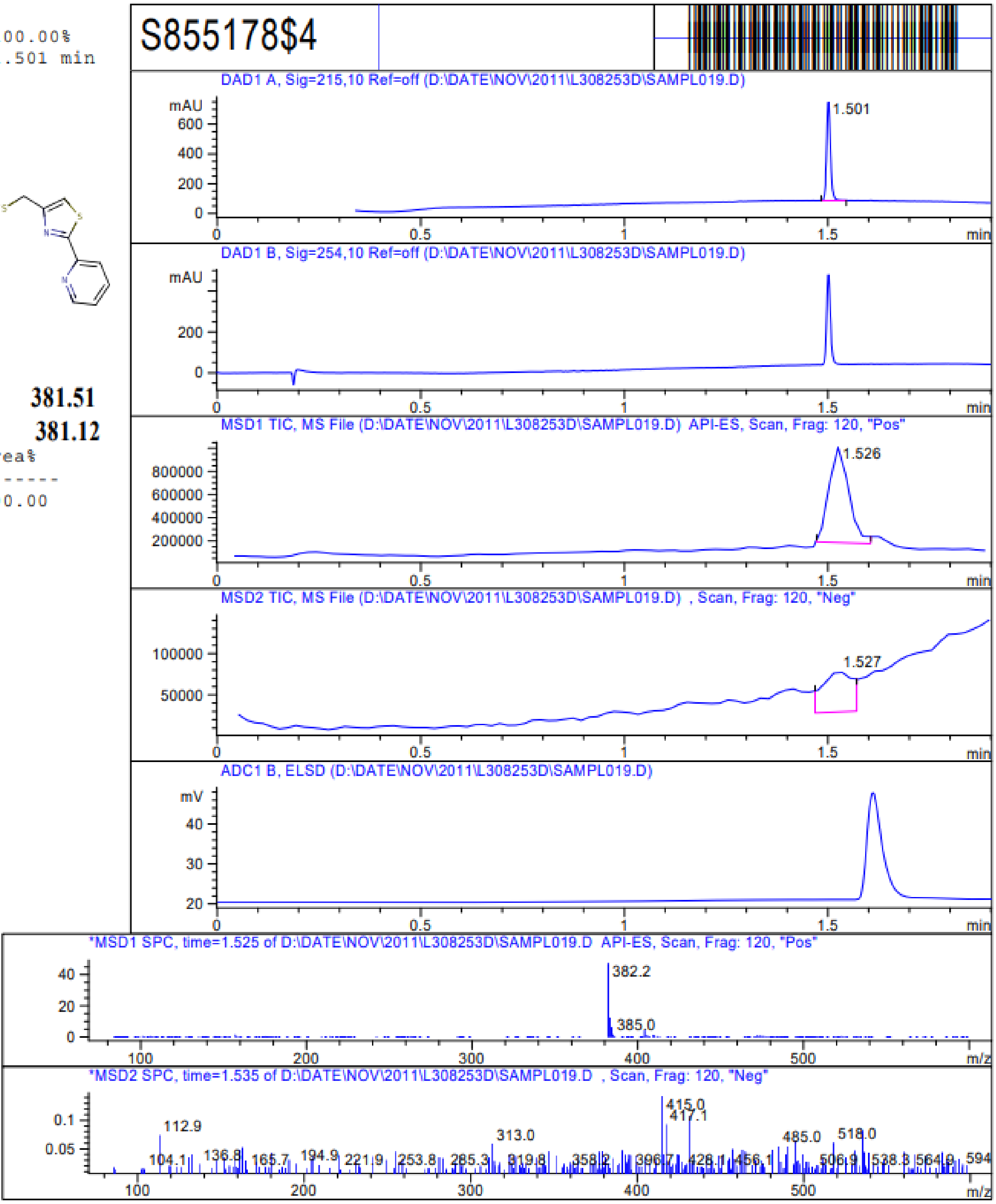

S855178$4

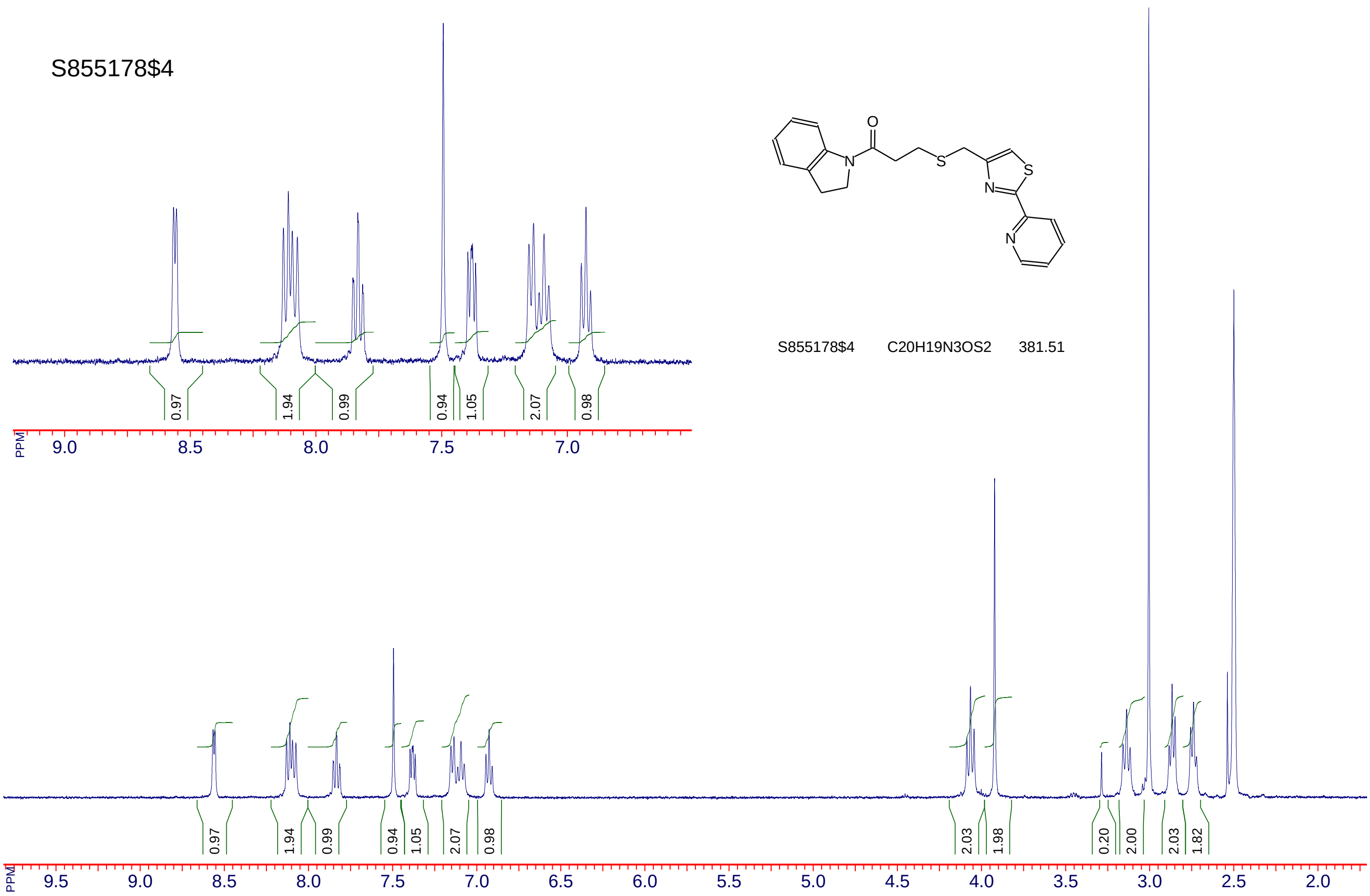

**File name: S855178$4**	Operator: Victorova	SF: 399.9755 MHz	NSC: 0	PW: 11.60 usec, RG: 34	SI: 65536
Date: 23-Nov-2020	Solvent: dmso+ccl4	SW: 7599 Hz	TE: 294 K	AQ: 2.11 sec, RD: 0.00 sec	

SPC-21 (Z224653792, Enamine)

MaxPeak: 100.00%
Ret_Time: 1.215 min

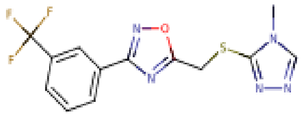

Mol Wt 341.31
Exact Mass 341.06

#	Time	Area%
1	1.215	100.00

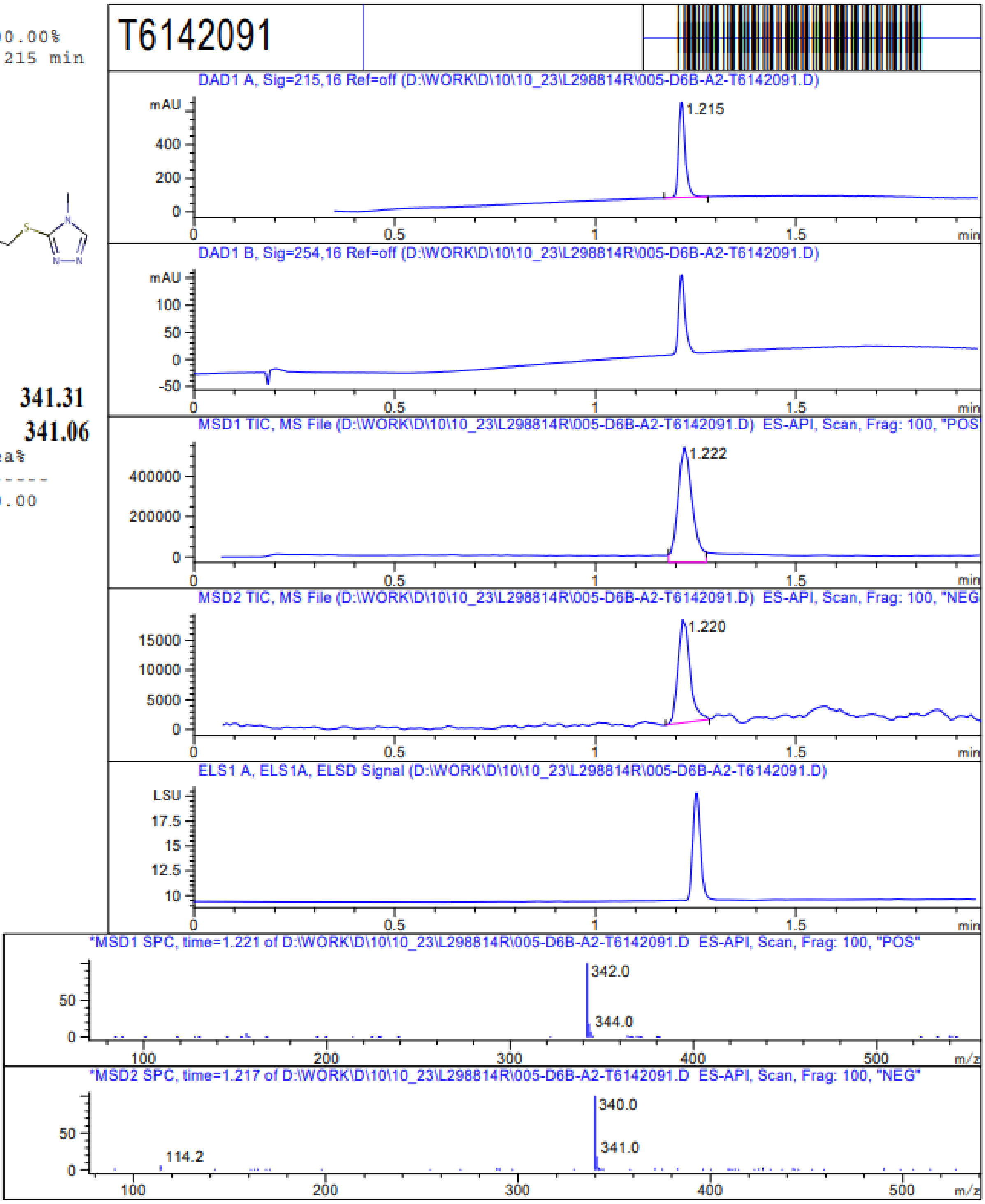

RT 1.222

RT 1.220

SPC-22 (Z17623220, Enamine)

MaxPeak: 90.19%
Ret_Time: 1.264 min

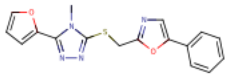

Mol Wt 338.38
Exact Mass 338.09

#	Time	Area%
1	1.264	90.19
2	1.389	6.78
3	1.542	3.03

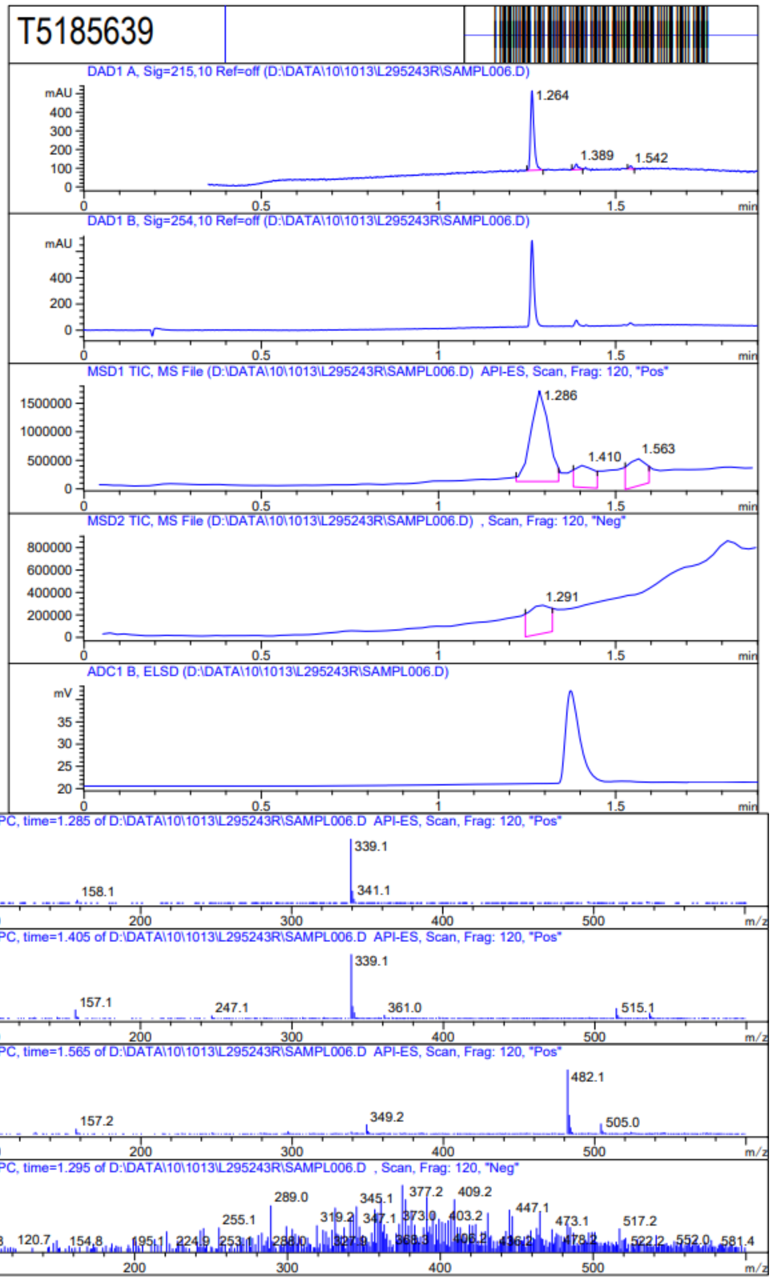

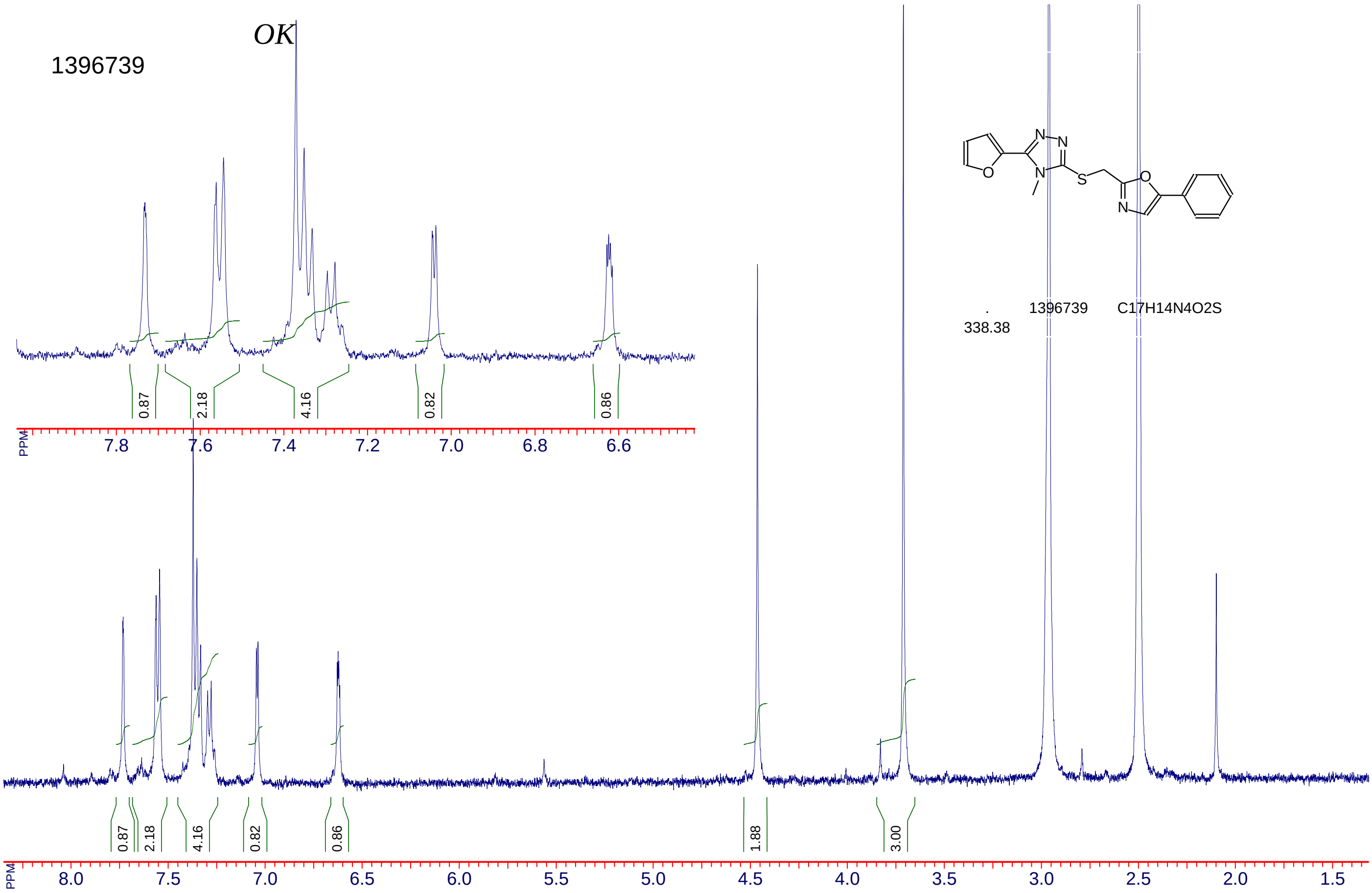

**File name: 1396739**	Vladimirova	SF: 399.9602 MHz	NSC: 0	PW: 7.00 usec, RG: 44	SI: 65536
Date: 24-Jul-2006	Solvent:	SW: 6364 Hz	TE: 295 K	AQ: 1.50 sec, RD: 0.00 sec	*1396739*

SPC-23 (Z97427814, Enamine)

MaxPeak: 94.58%
Ret_Time: 1.309 min

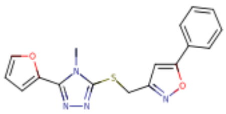

Mol Wt 338.38
Exact Mass 338.09

#	Time	Area%
1	1.287	3.48
2	1.309	94.58
3	1.406	1.93

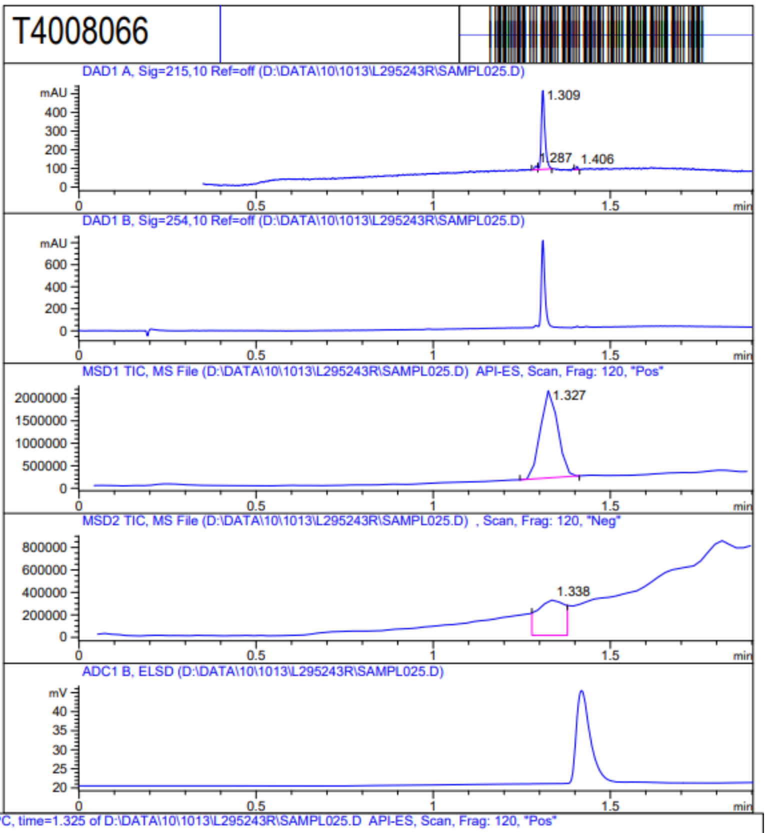

RT 1.327

RT 1.338

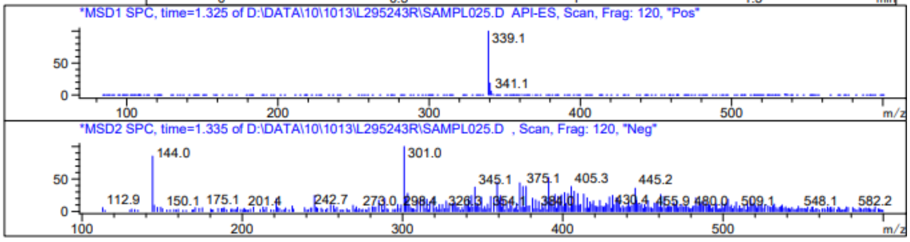

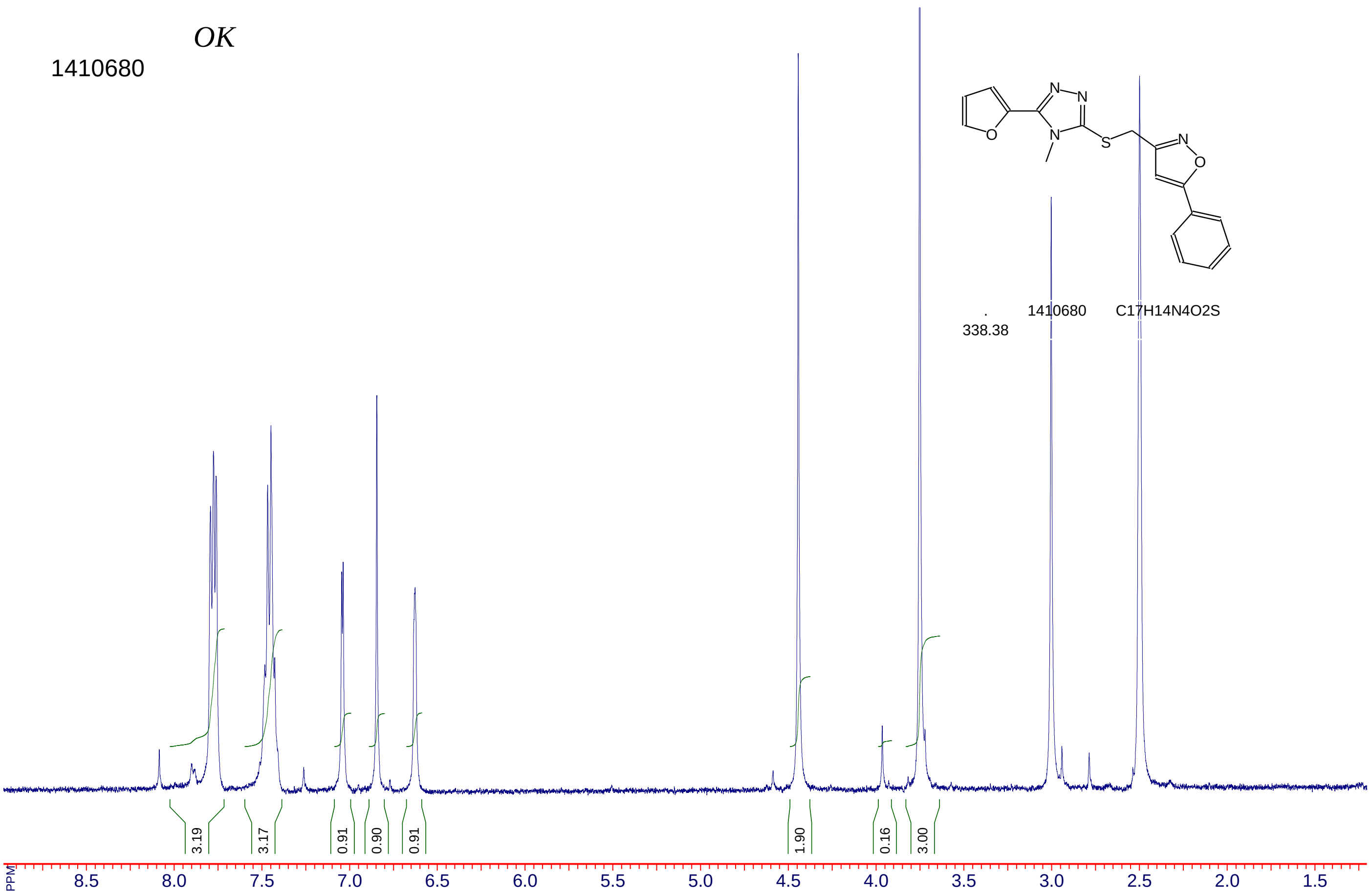

**File name: 1410680**	Vladimirova	SF: 399.9602 MHz	NSC: 0	PW: 7.00 usec, RG: 44	SI: 65536
Date: 10-Aug-2006	Solvent:	SW: 6364 Hz	TE: 295 K	AQ: 1.50 sec, RD: 0.00 sec	*1410680*

SPC-24 (STK438942, Vitas-M)

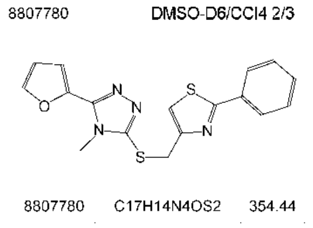

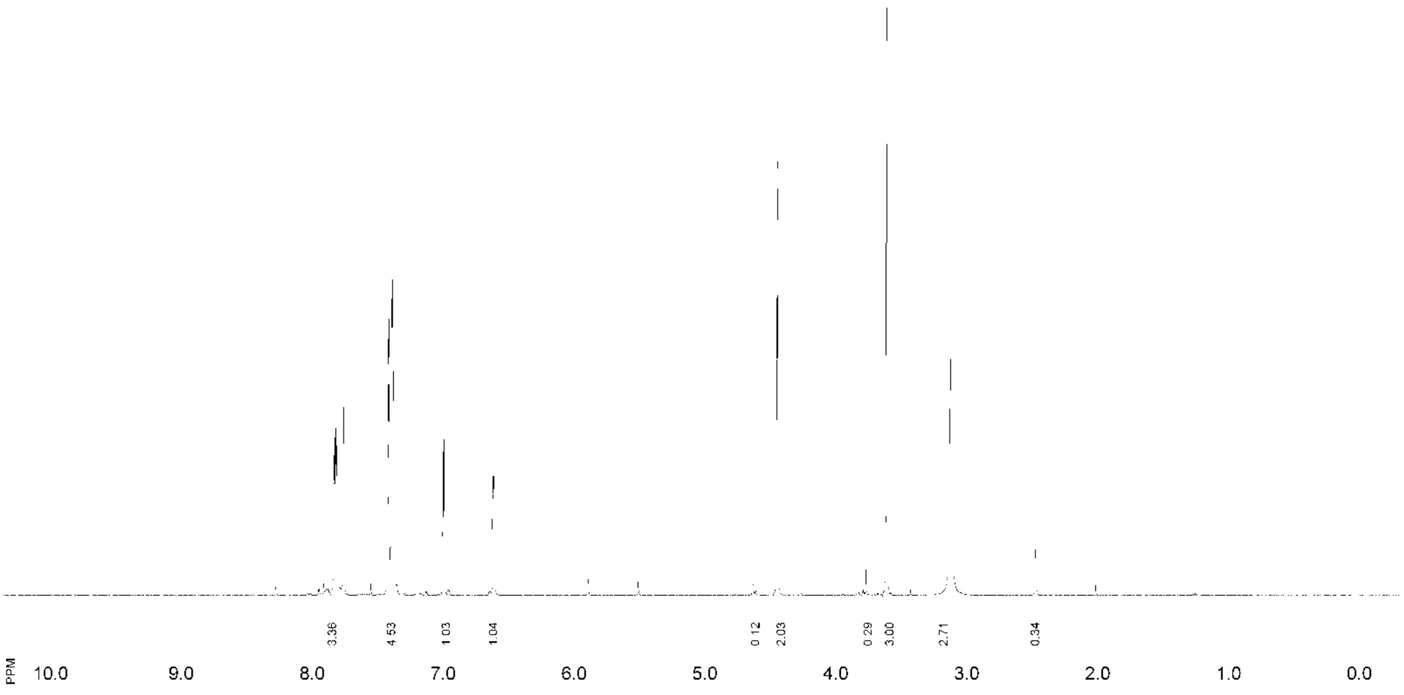

SPC-25 (STK439840, Vitas-M)

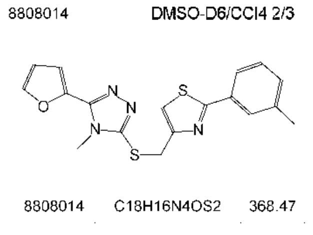

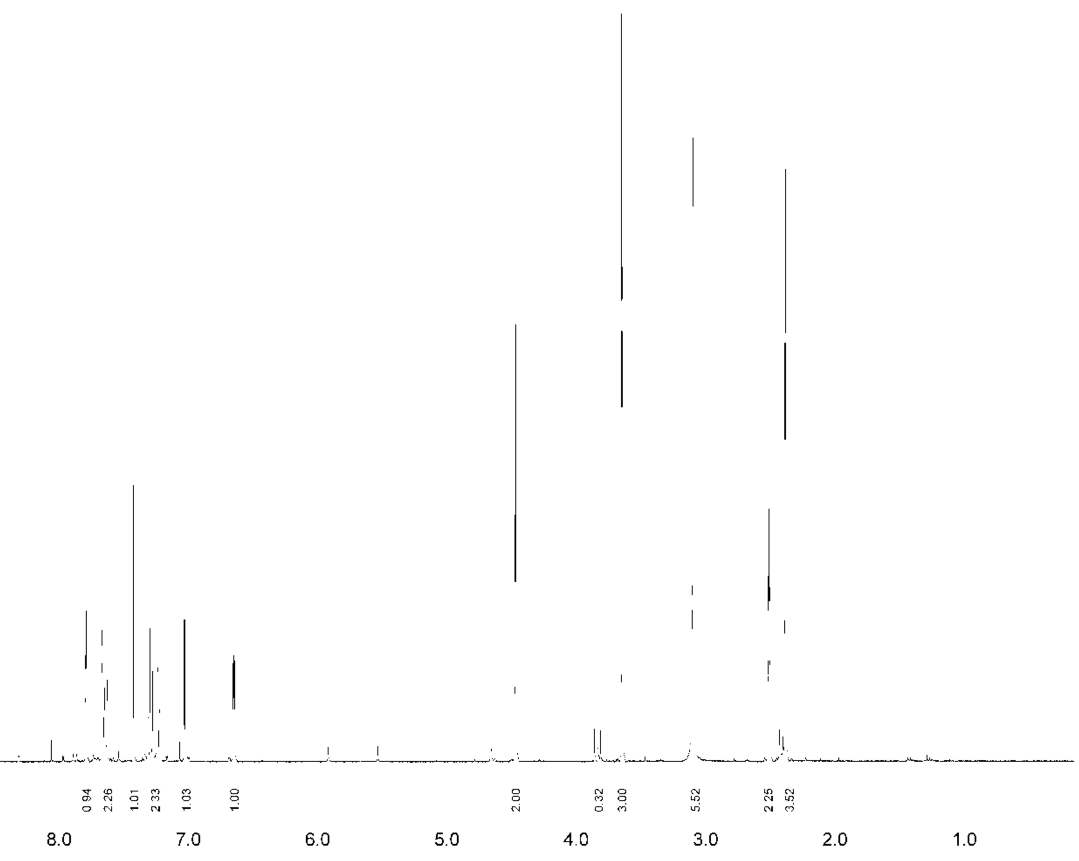

SPC-26 (STL416744, Vitas-M)

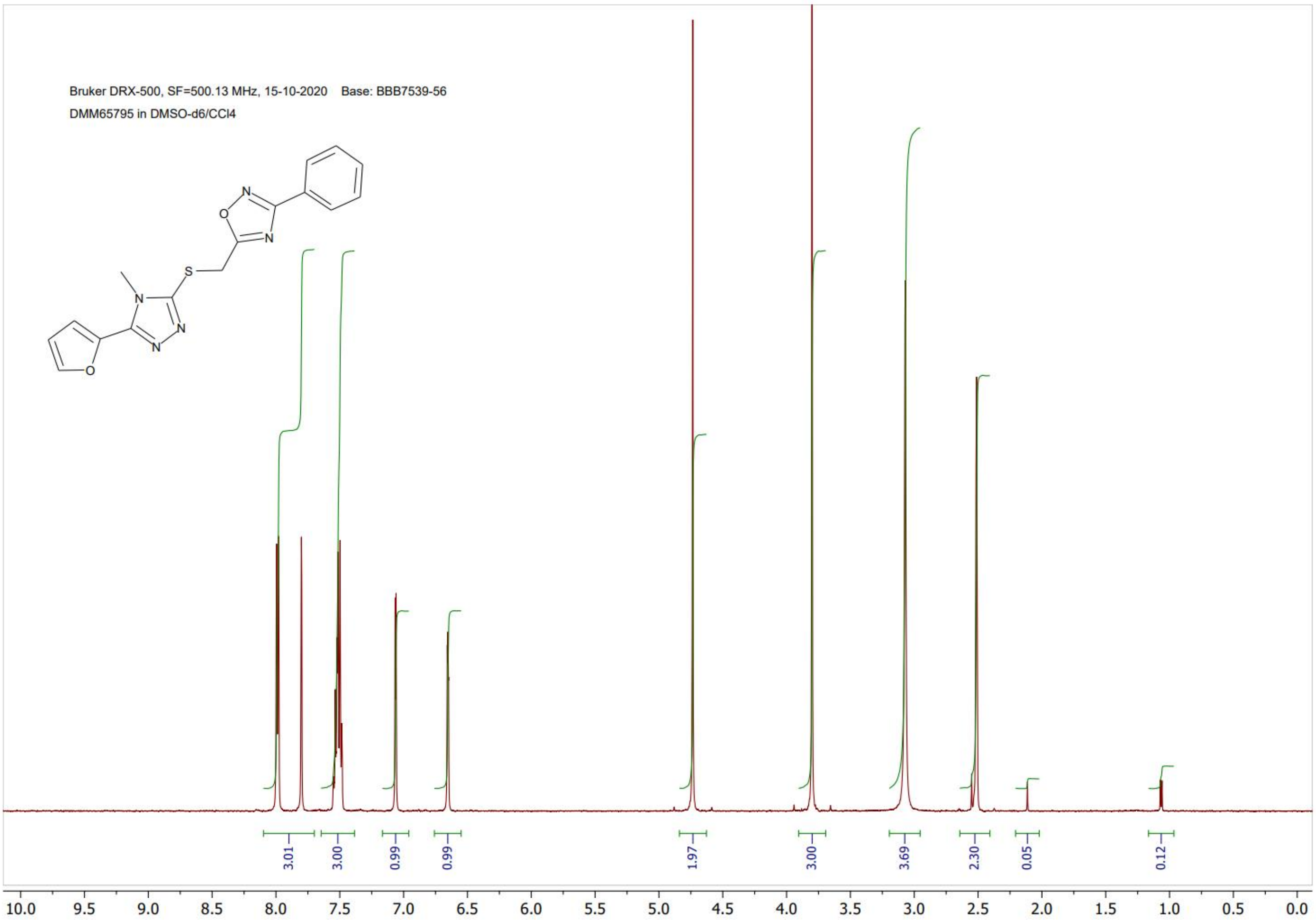

SPC-27 (Z92189339, Enamine)

MaxPeak: 100.00%
Ret_Time: 1.231 min

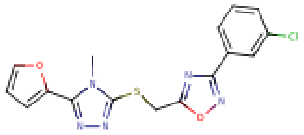

Mol Wt 373.82
Exact Mass 373.04

#	Time	Area%
1	1.231	100.00

RT 1.246

RT 1.241

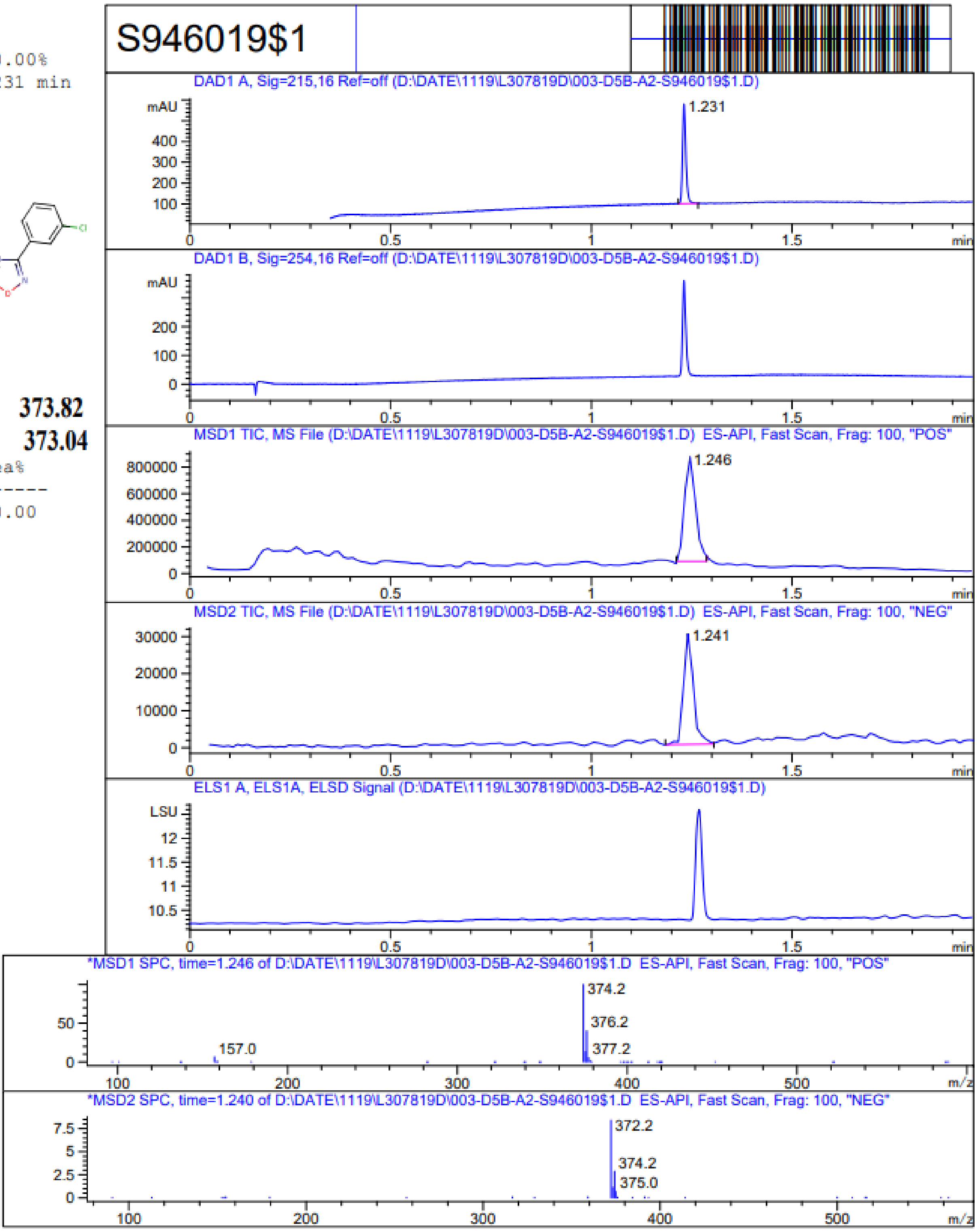

S946019$1

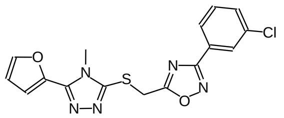

S946019$1   C16H12ClN5O2S   373.82

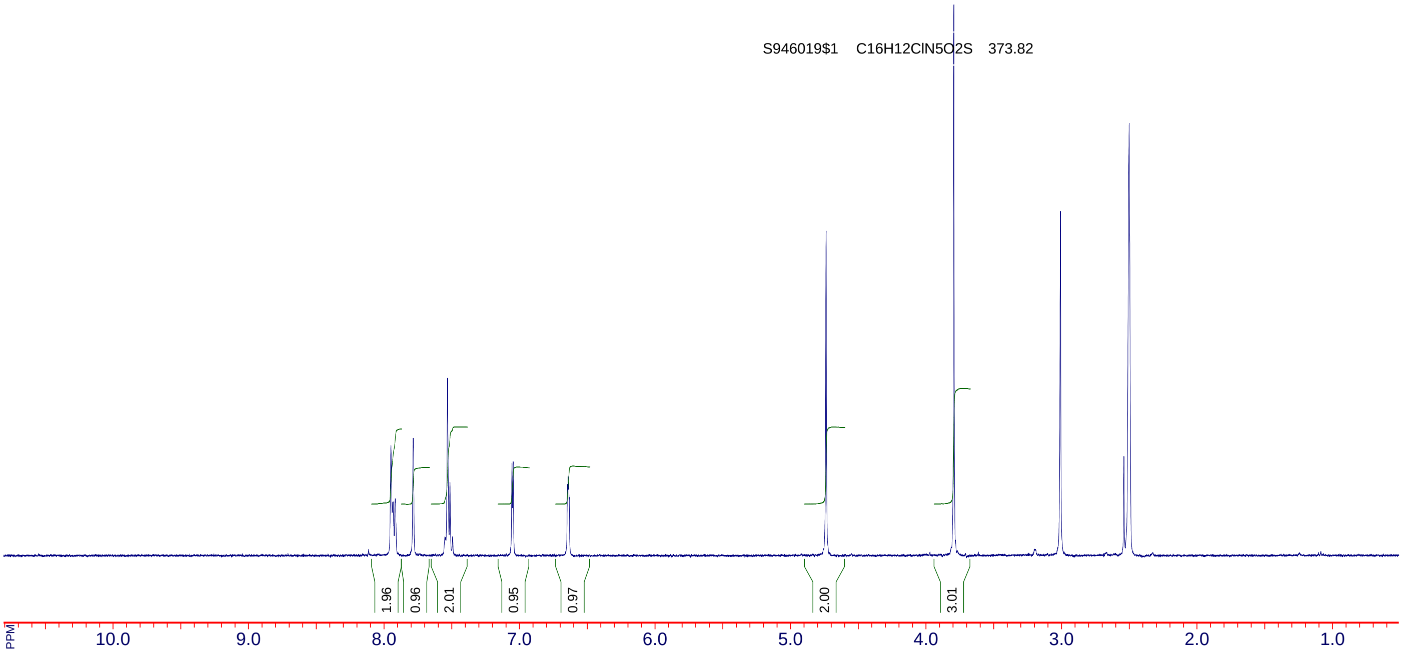

File name: S946019$1	Honchar	SF: 399.9755 MHz	NSC: 0	PW: 11.60 usec, RG: 40	SI: 65536
Date: 20-Nov-2020	Solvent: dmso+ccl4	SW: 7599 Hz	TE: 294 K	AQ: 2.11 sec, RD: 0.00 sec	S946019$1

SPC-28 (STL406665, Vitas-M)

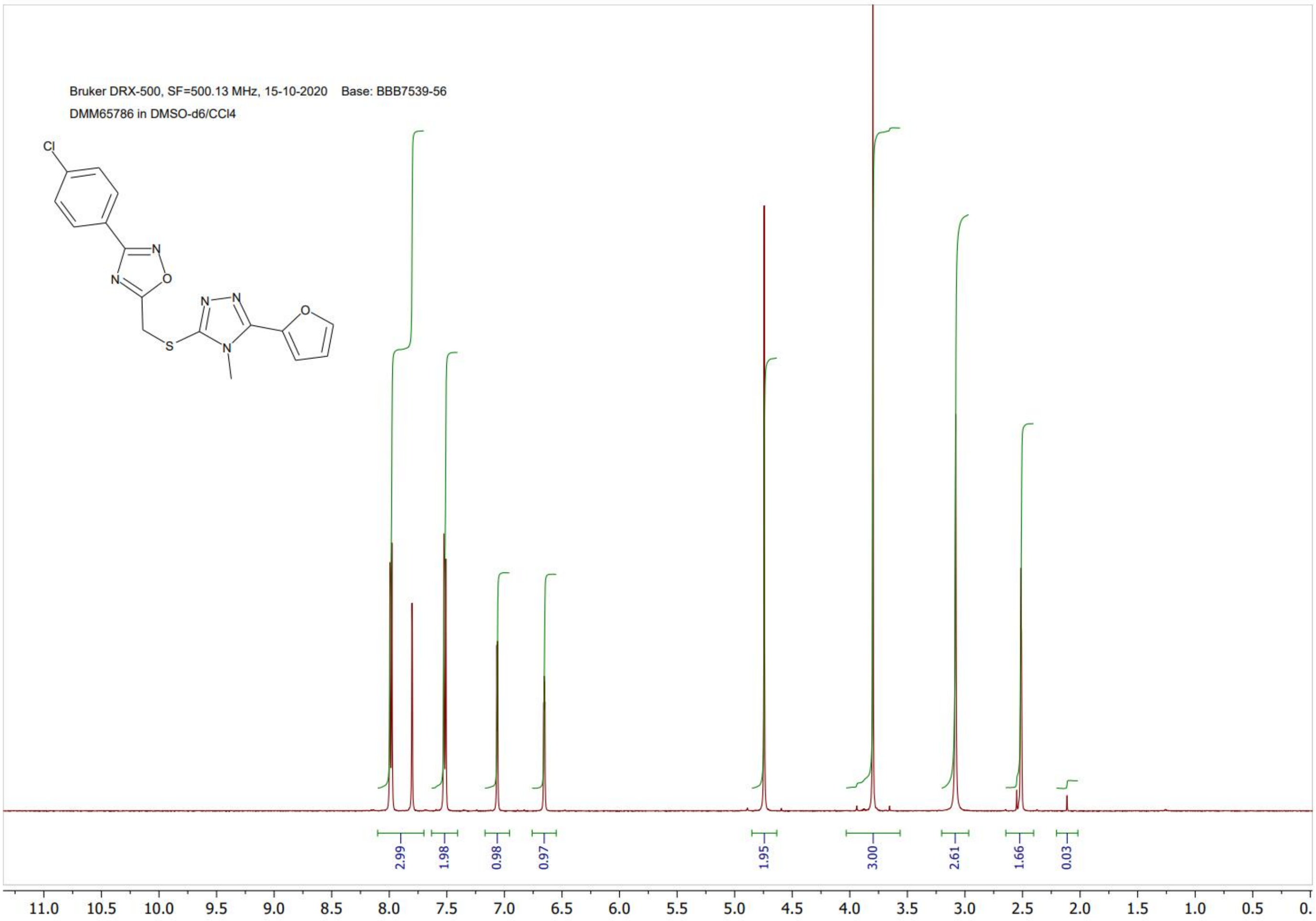

SPC-29 (Z17622202, Enamine)

MaxPeak: 97.19%
Ret_Time: 1.233 min

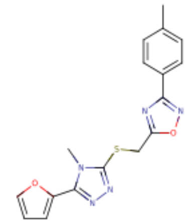

Mol Wt 353.4
Exact Mass 353.1

#	Time	Area%
1	0.799	2.81
2	1.233	97.19

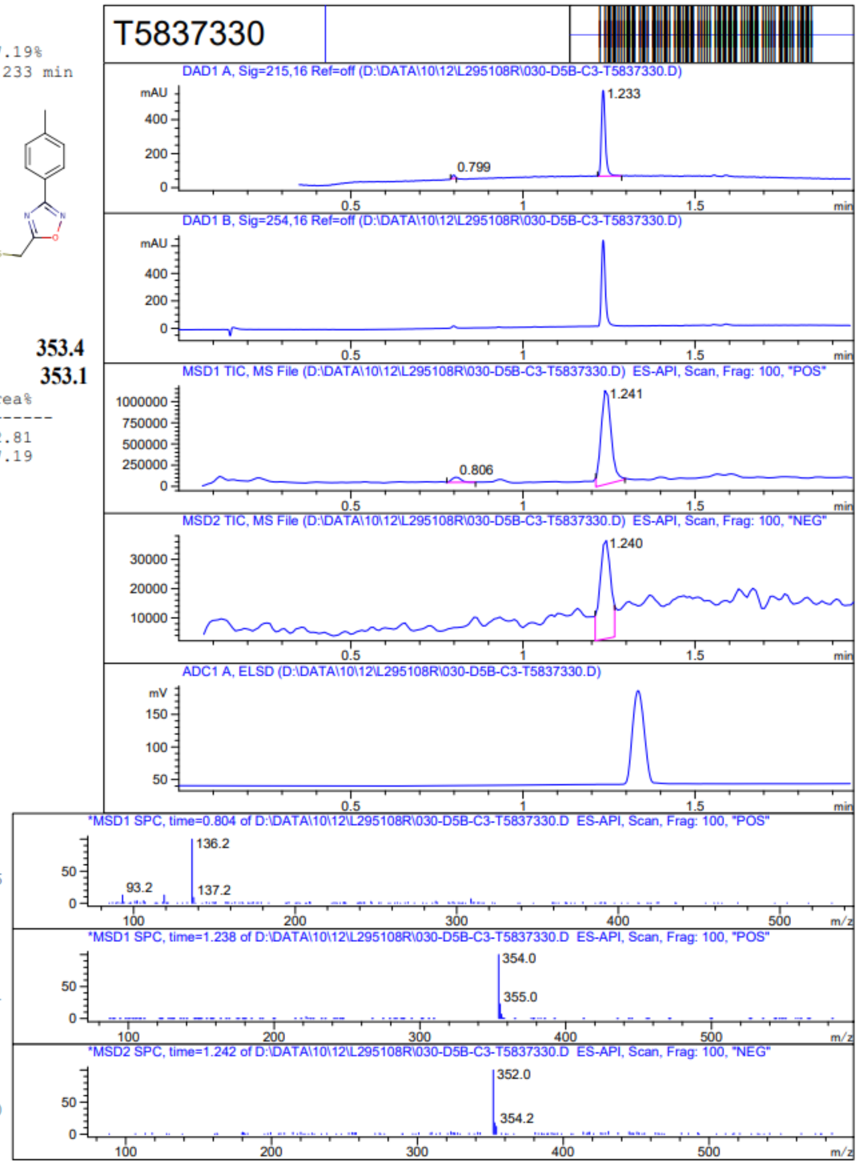

SPC-30 (STL407111, Vitas-M)

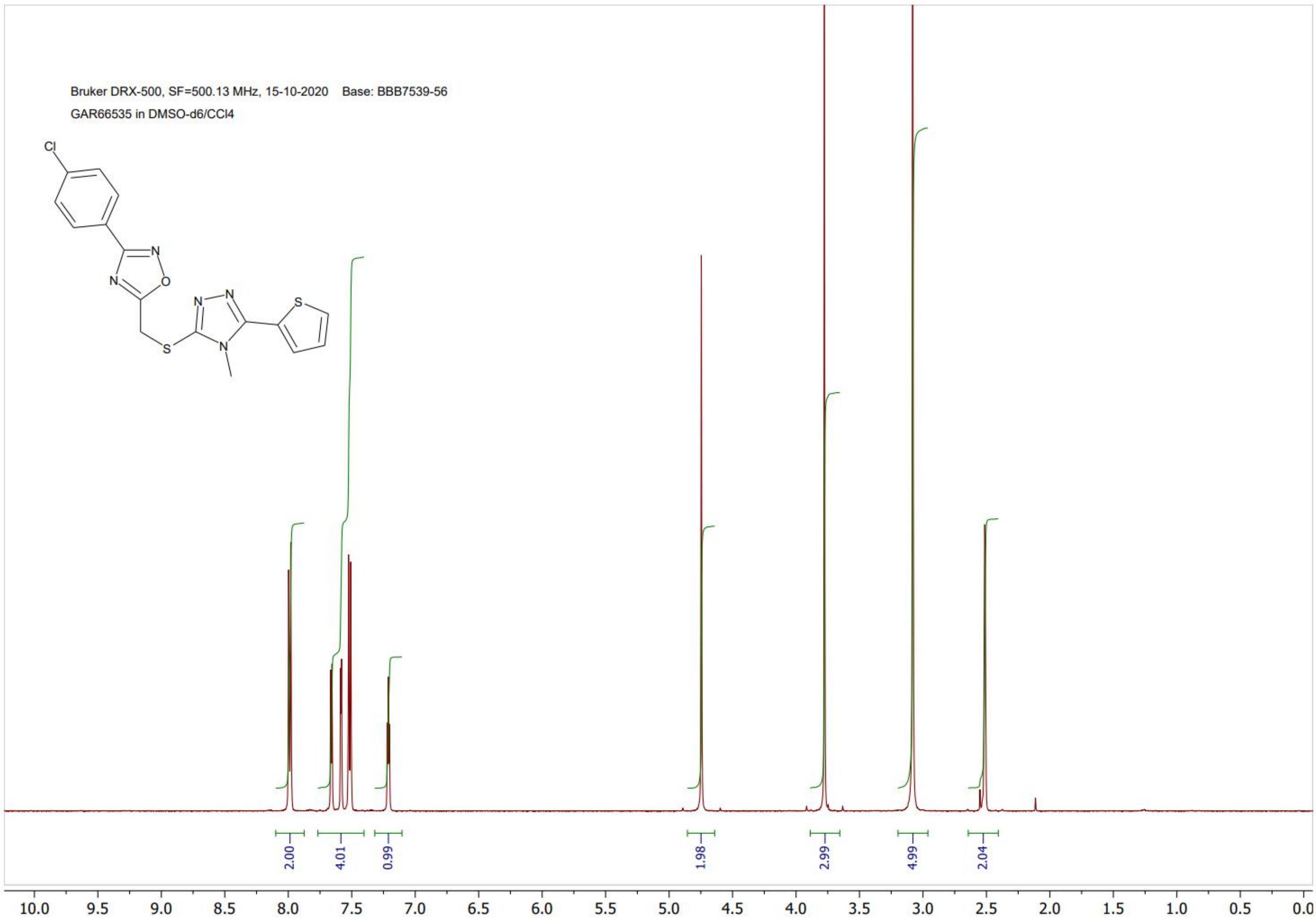

SPC-31 (STL407120, Vitas-M)

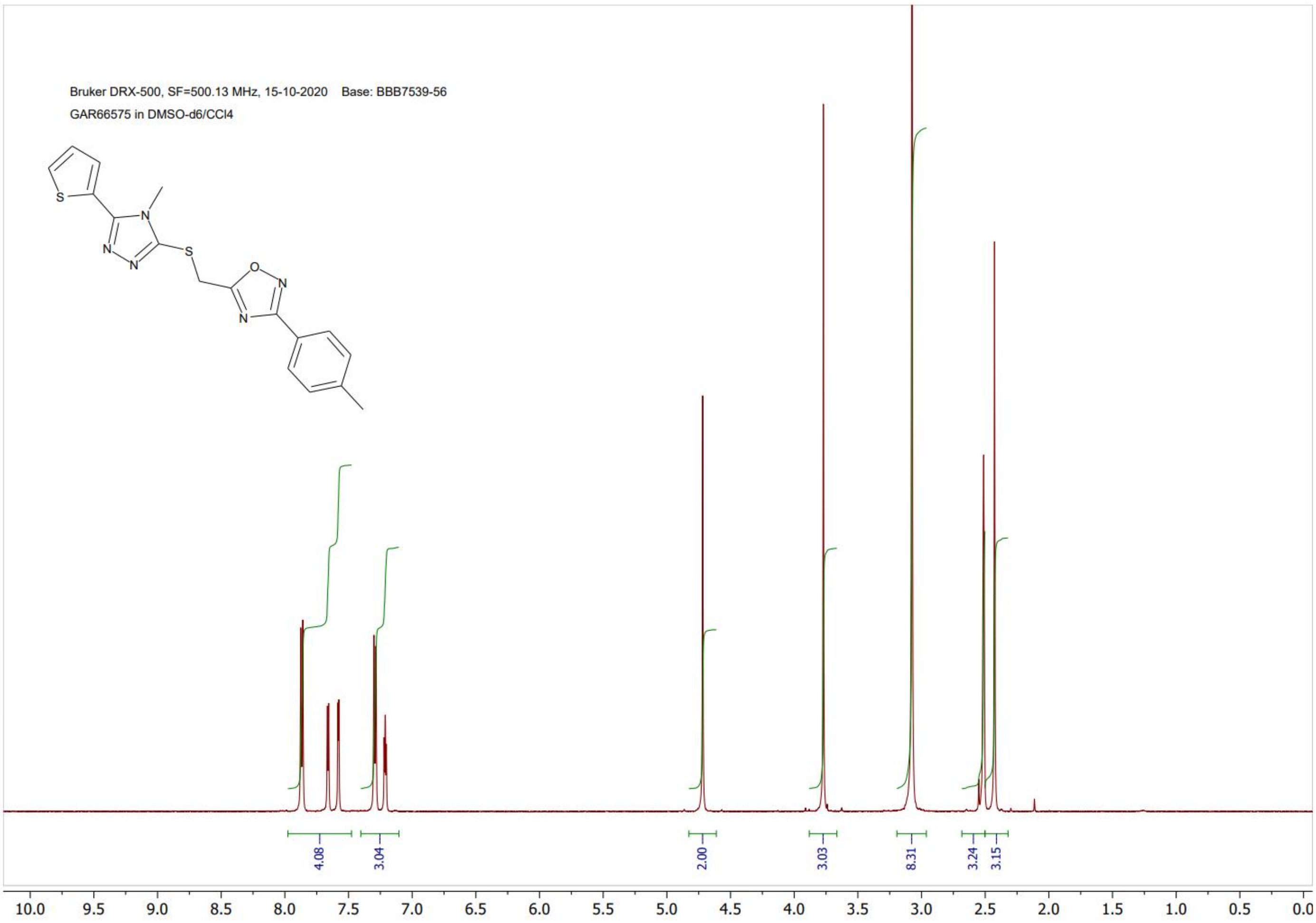

SPC-32 (7982551, ChemBridge)

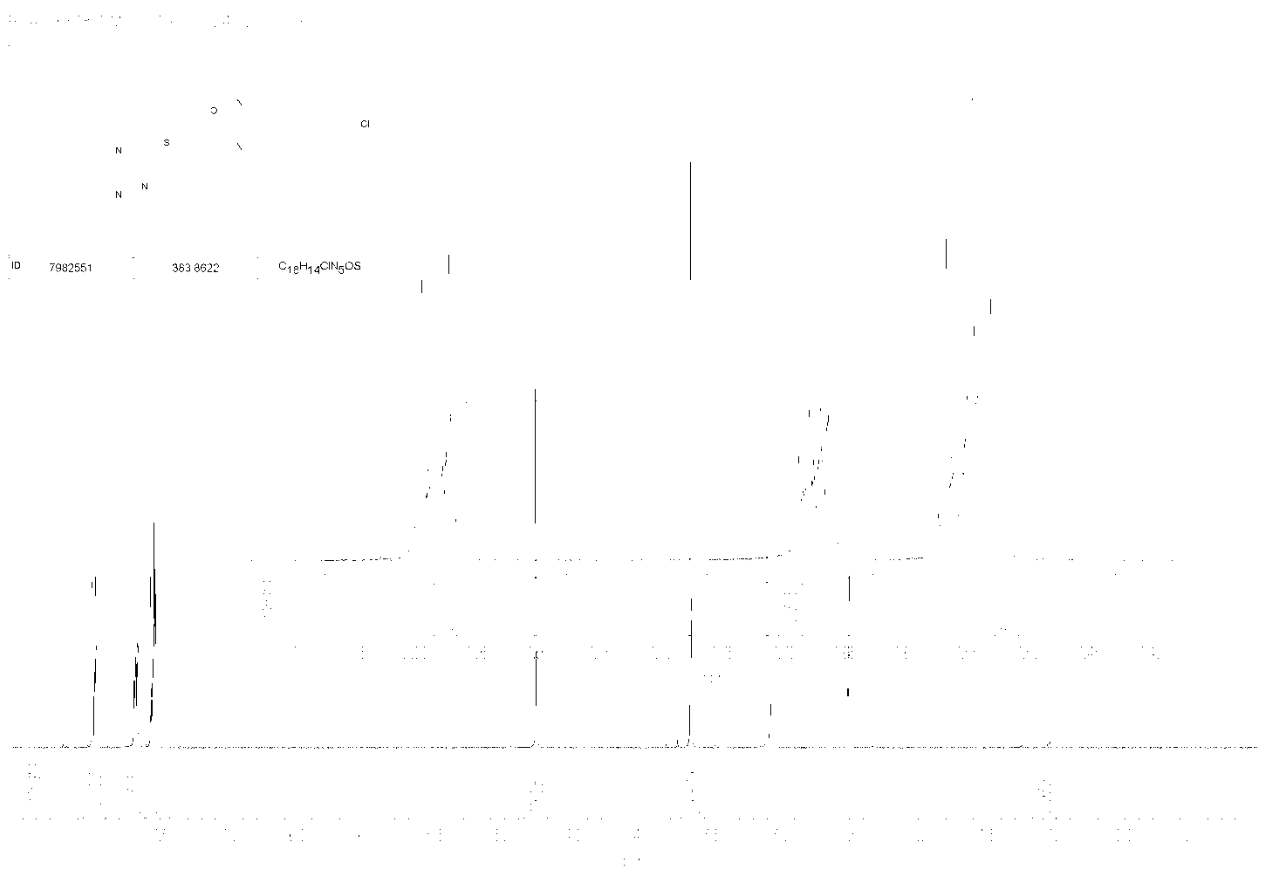

SPC-33 (STL419475, Vitas-M)

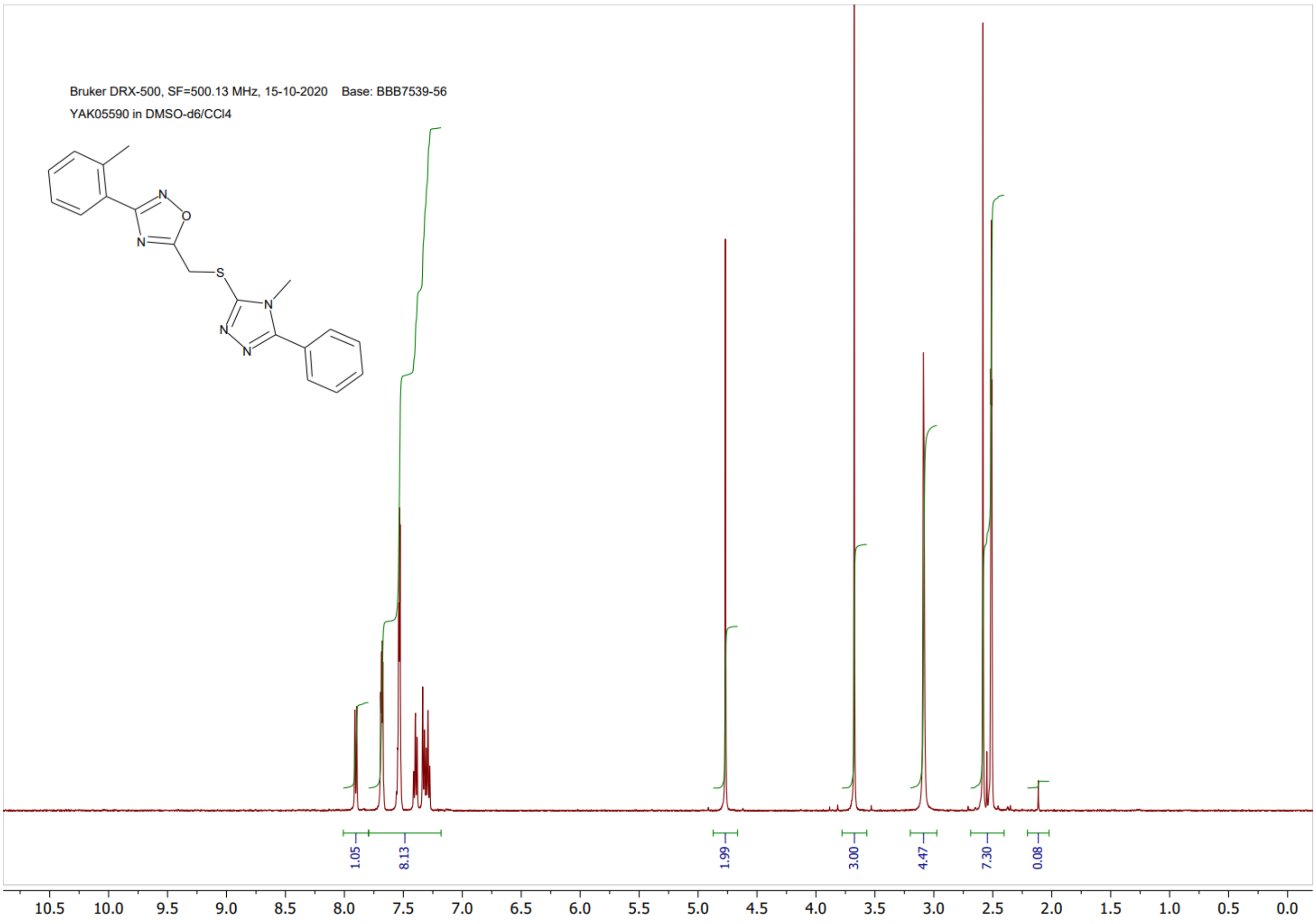

SPC-34 (Z92036425, Enamine)

MaxPeak: 100.00%
Ret_Time: 1.342 min

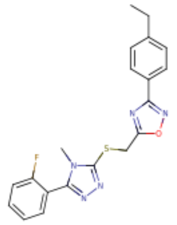

Mol Wt 395.45
Exact Mass 395.14

#	Time	Area%
1	1.342	100.00

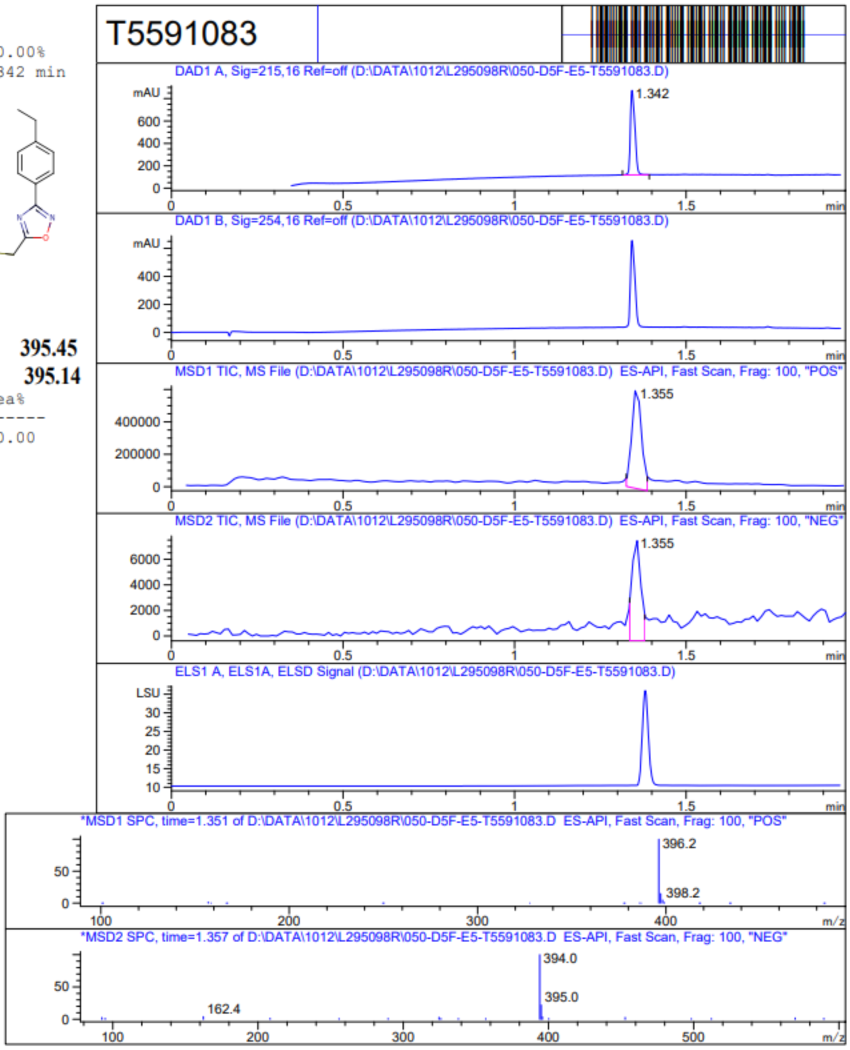

SPC-35 (Z51206239, Enamine)

MaxPeak: 100.00%
Ret_Time: 1.020 min

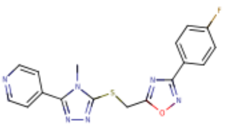

Mol Wt 368.39
Exact Mass 368.09

#	Time	Area%
1	1.020	100.00

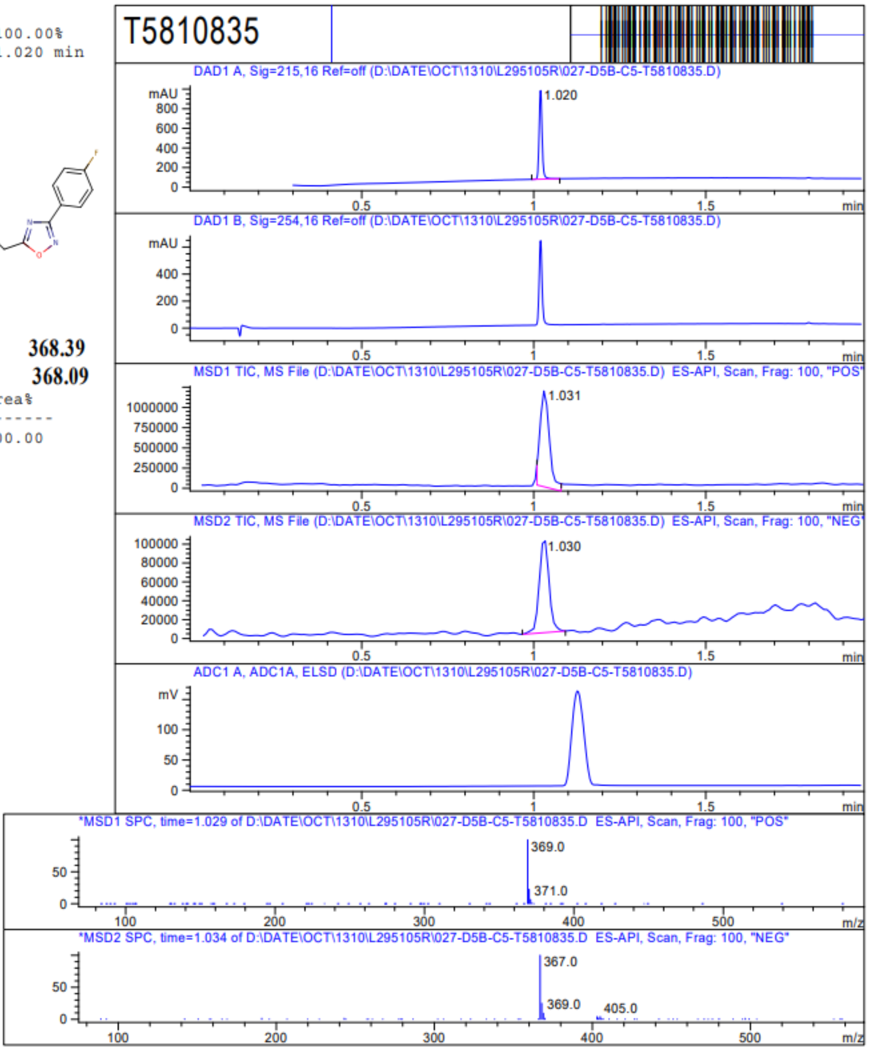

RT 1.031

RT 1.030

1582200

OK

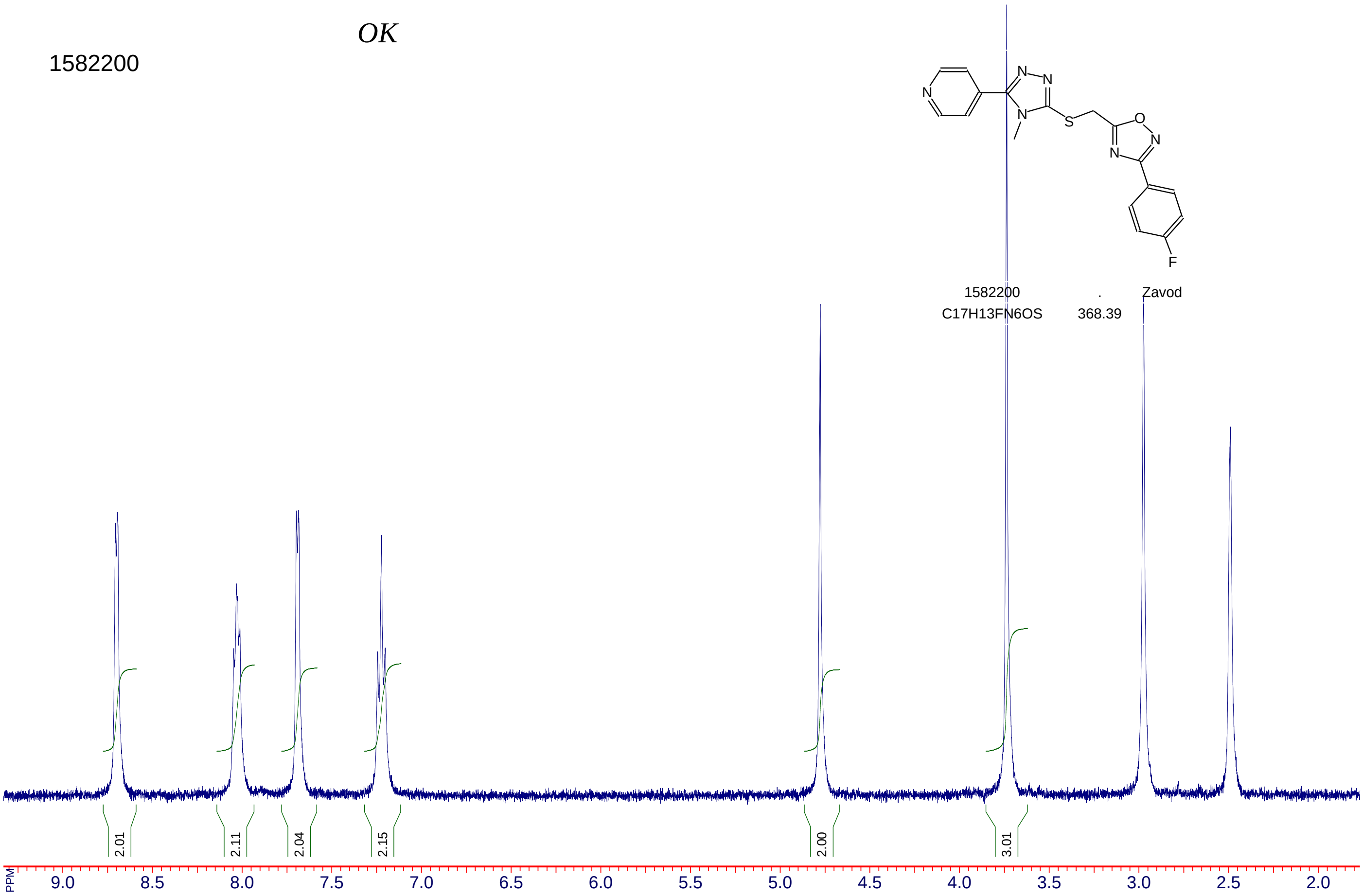

**File name: 1582200**	vaskivska	SF: 399.9681 MHz	NSC: 0	PW: 19.30 usec, RG: 50	SI: 65536
Date: 27-Dec-2006		SW: 6369 Hz	TE: 295 K	AQ: 1.50 sec, RD: 0.00 sec	*1582200*

SPC-36 (STL406361, Vitas-M)

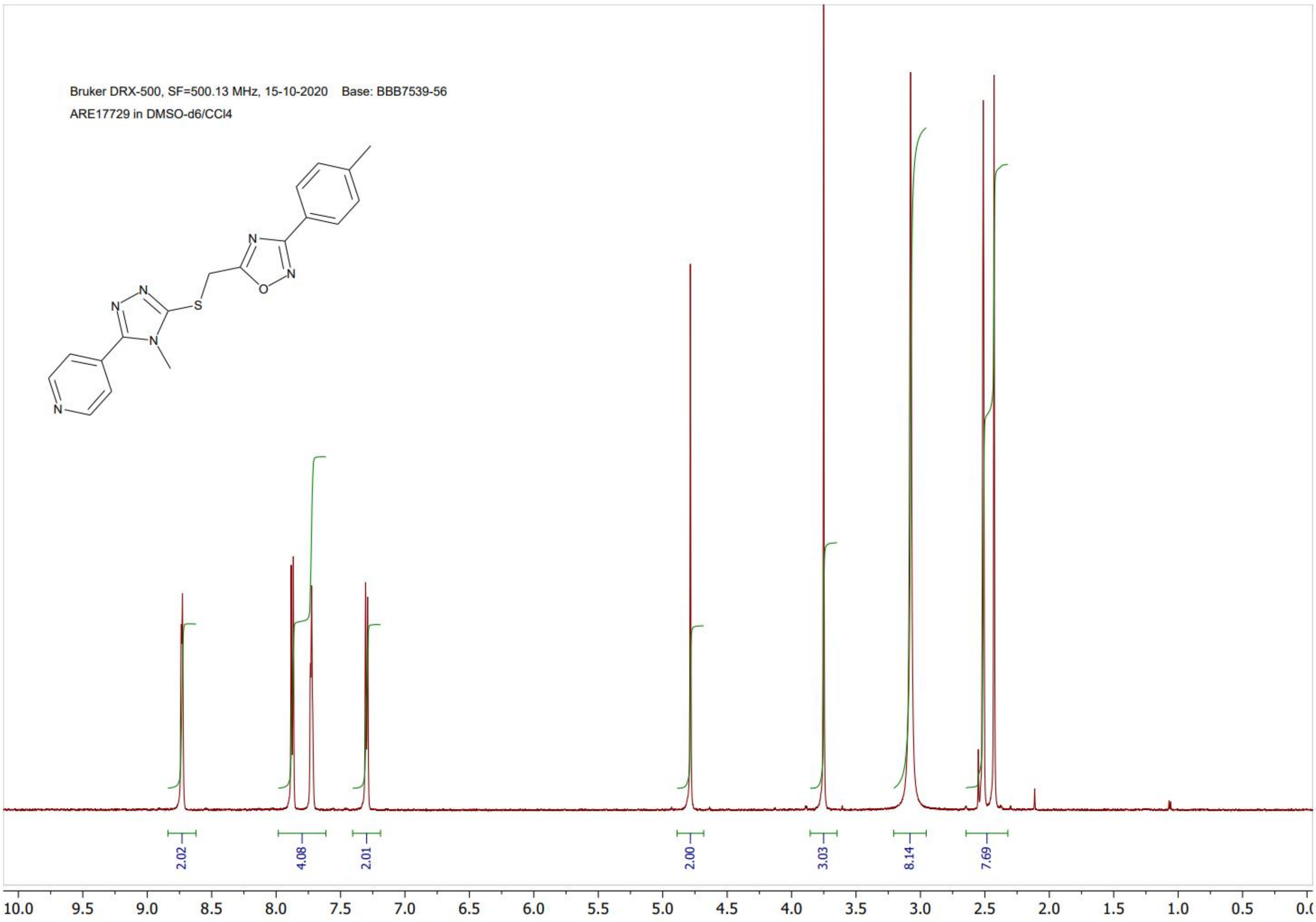

SPC-37 (7952109, ChemBridge)

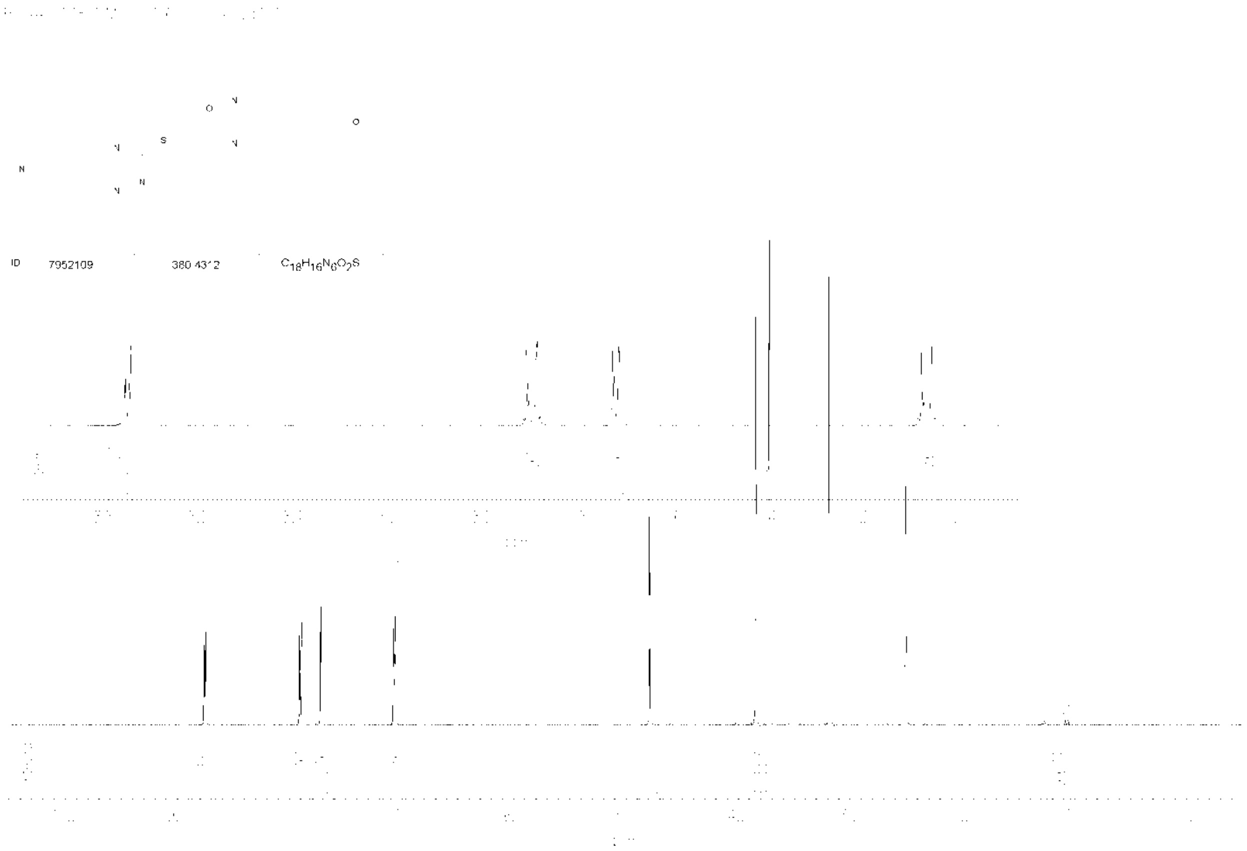

SPC-38 (Z51233214, Enamine)

```
MaxPeak:   100.00%
Ret_Time: 1.052 min
```

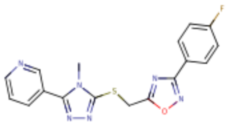

Mol Wt 368.39

Exact Mass 368.09

#	Time	Area%
1	1.052	100.00

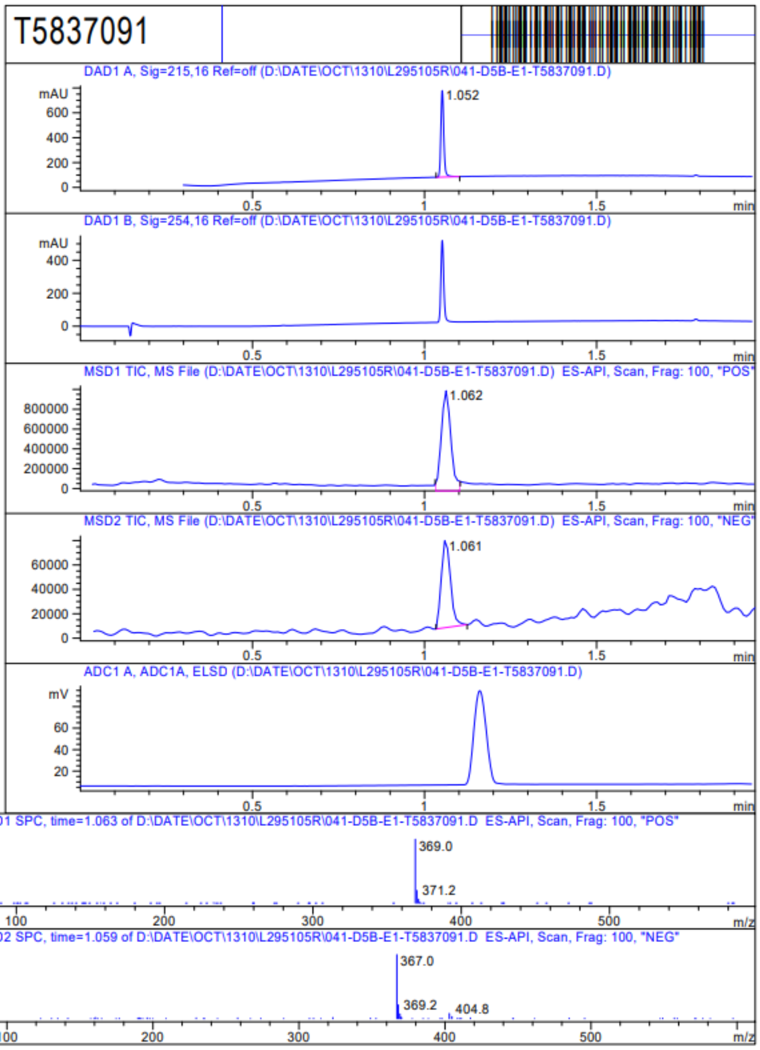

SPC-39 (STK860992, Vitas-M)

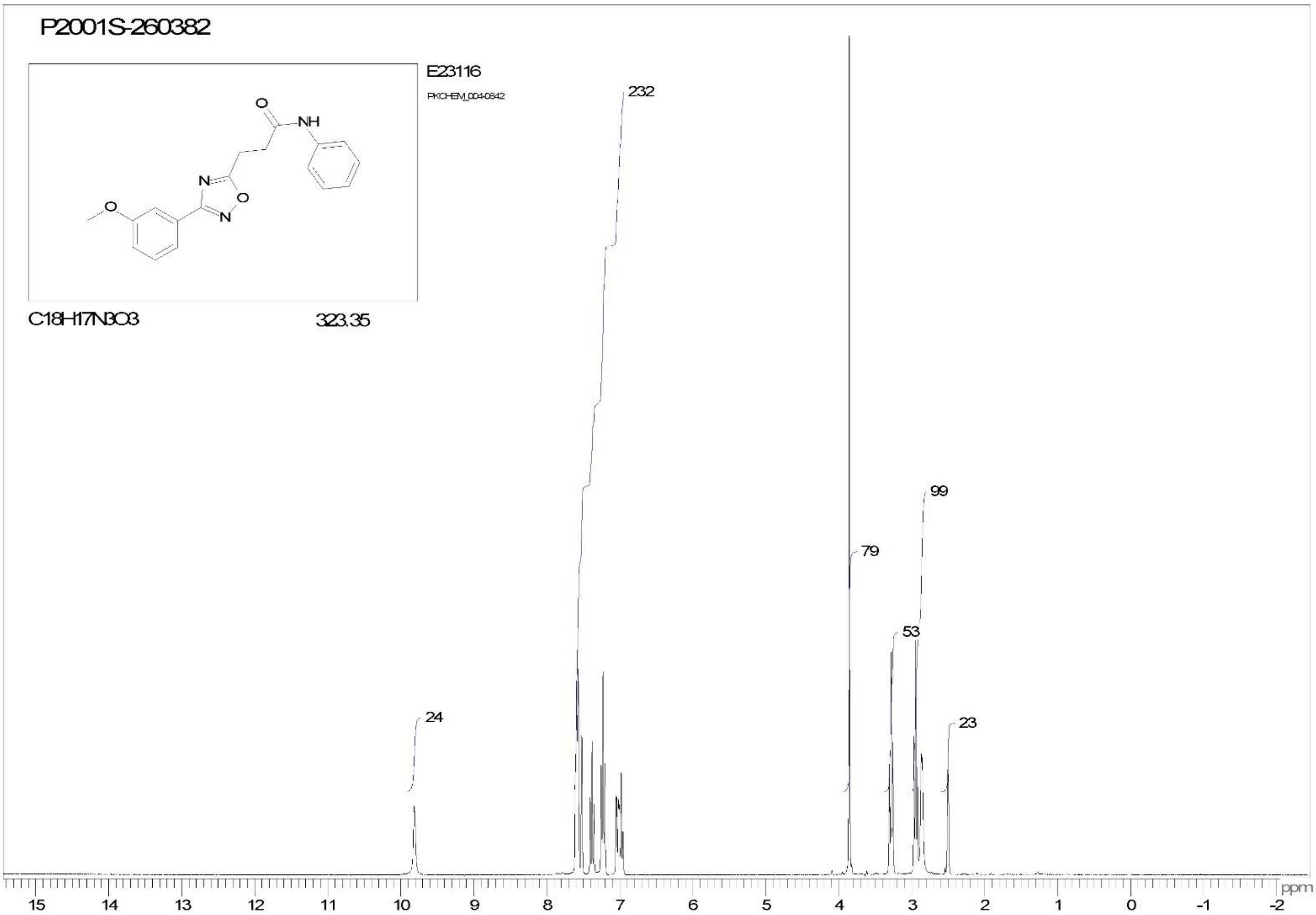

SPC-40 (STK860994, Vitas-M)

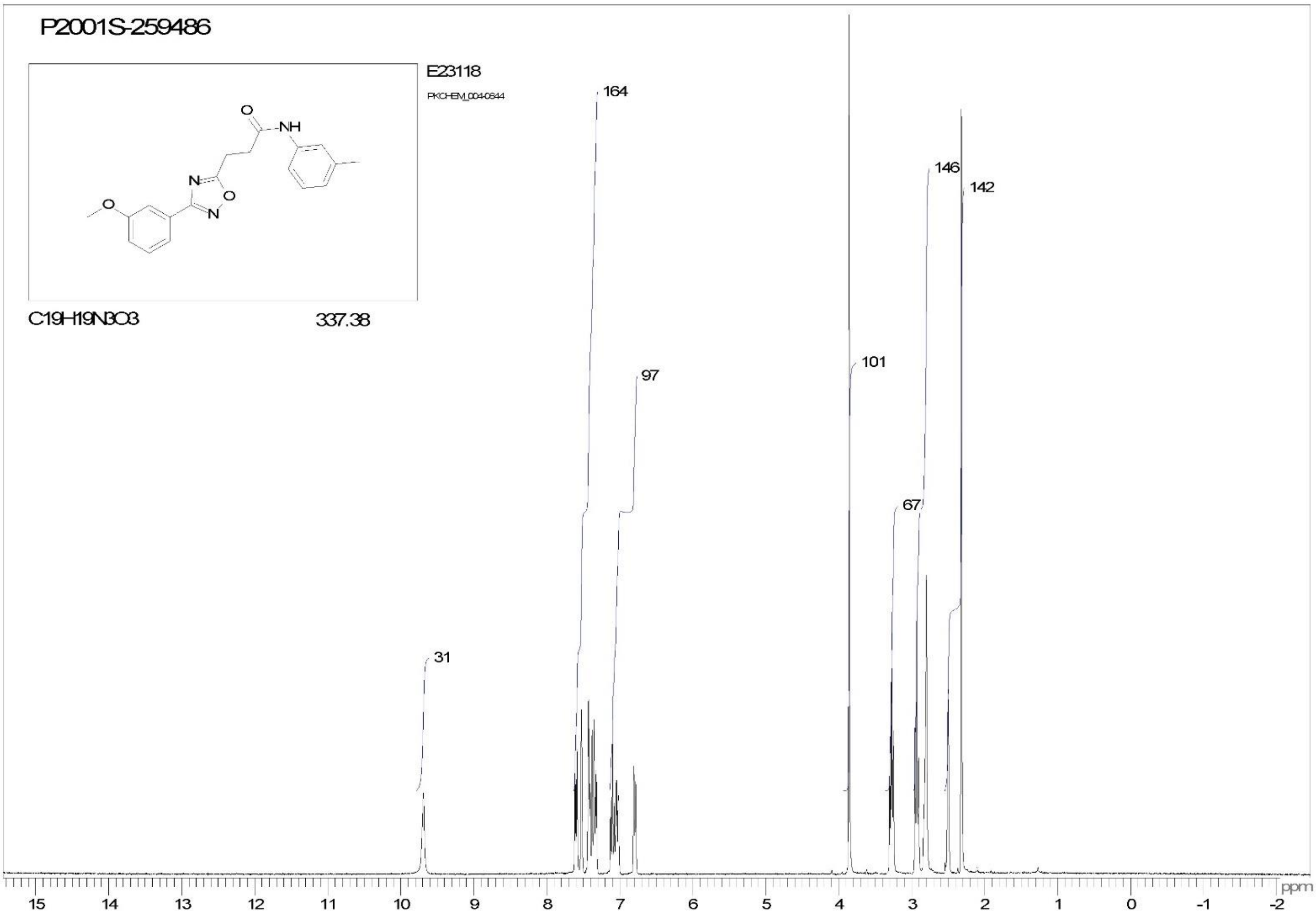

SPC-41 (STK860995, Vitas-M)

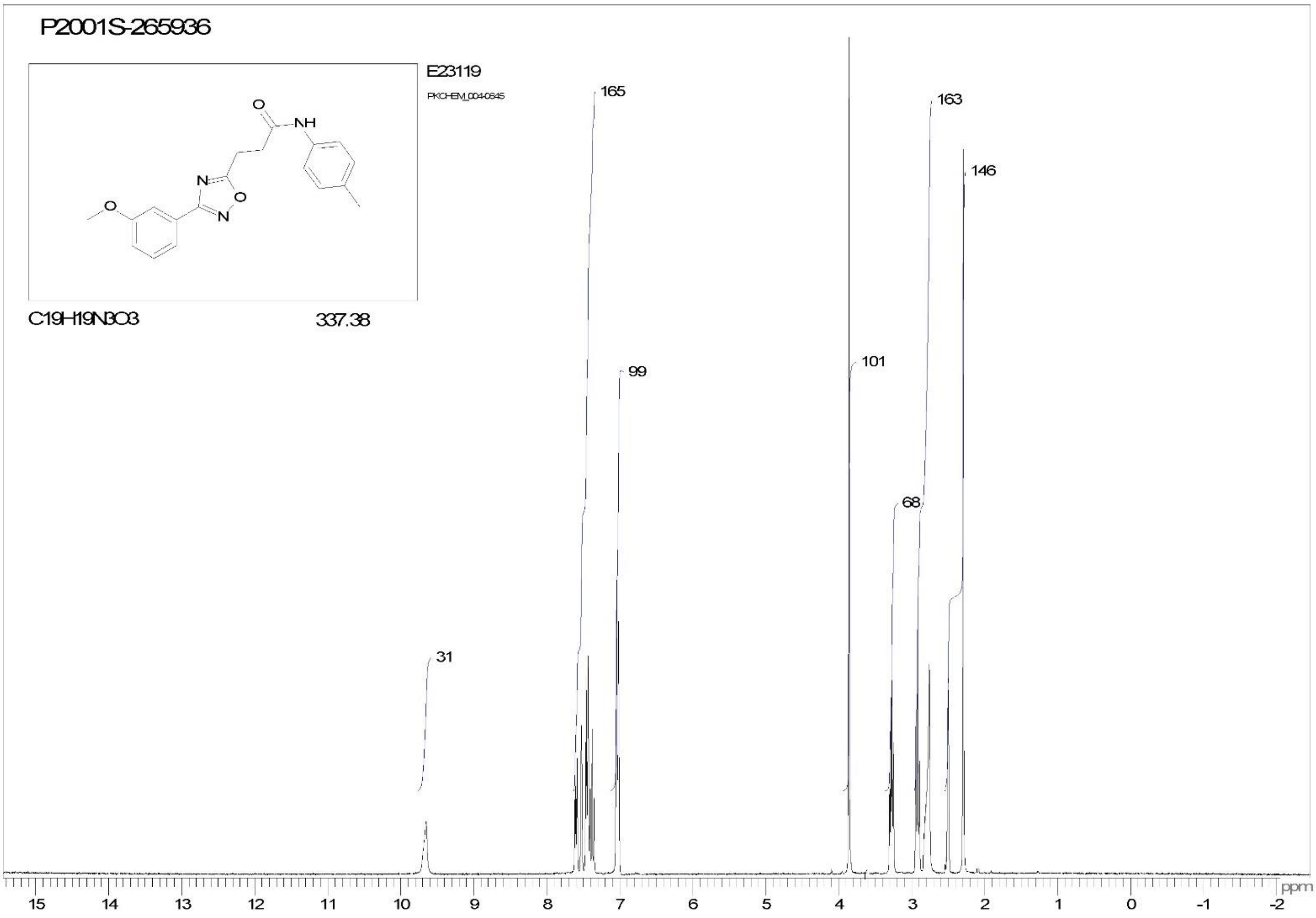

SPC-42 (Z2932911026, Enamine)

MaxPeak: 100.00%
Ret_Time: 1.476 min

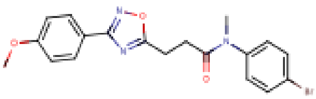

Mol Wt 416.27
Exact Mass 415.07

#	Time	Area%
1	1.476	100.00

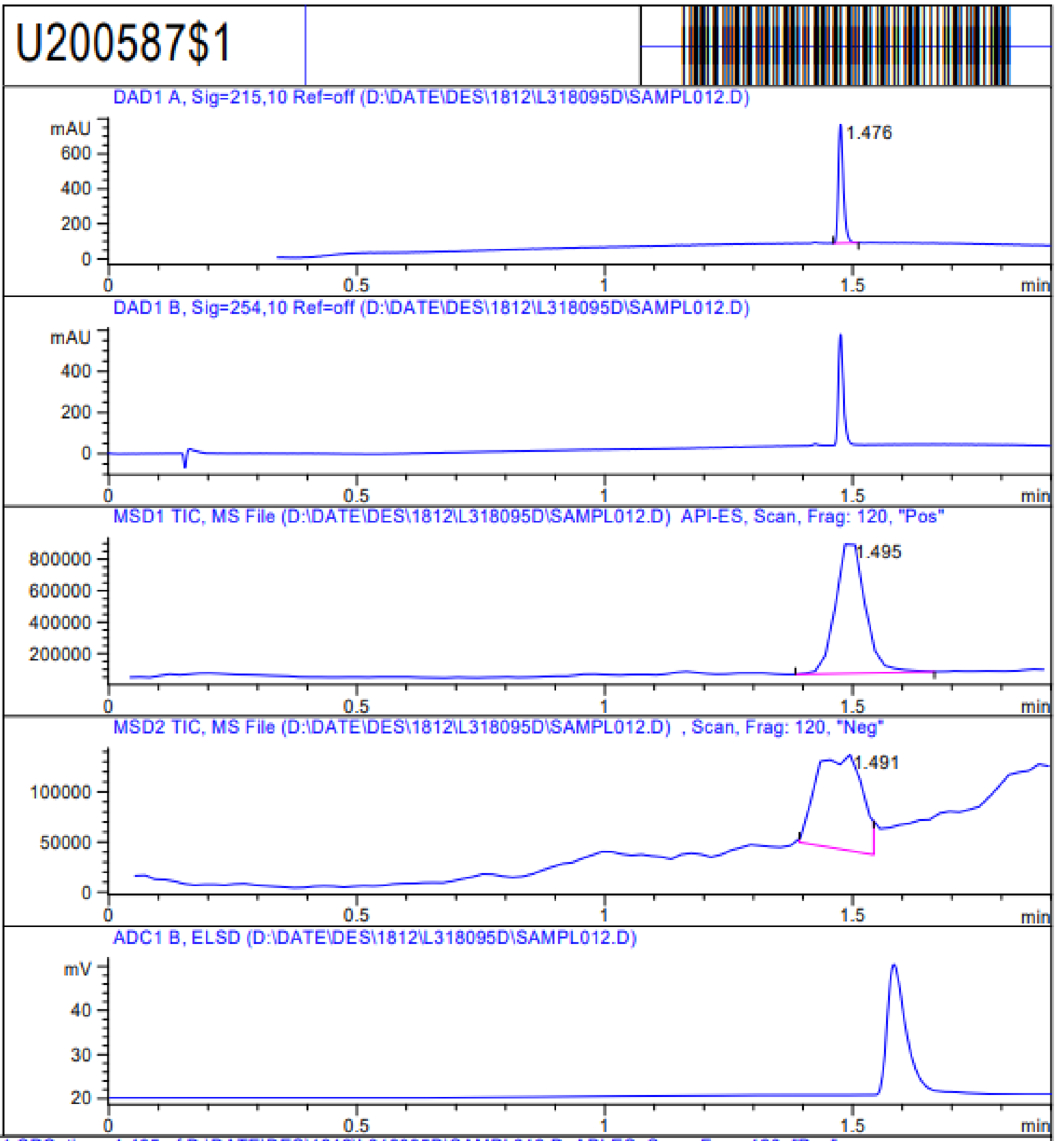

RT 1.495

RT 1.491

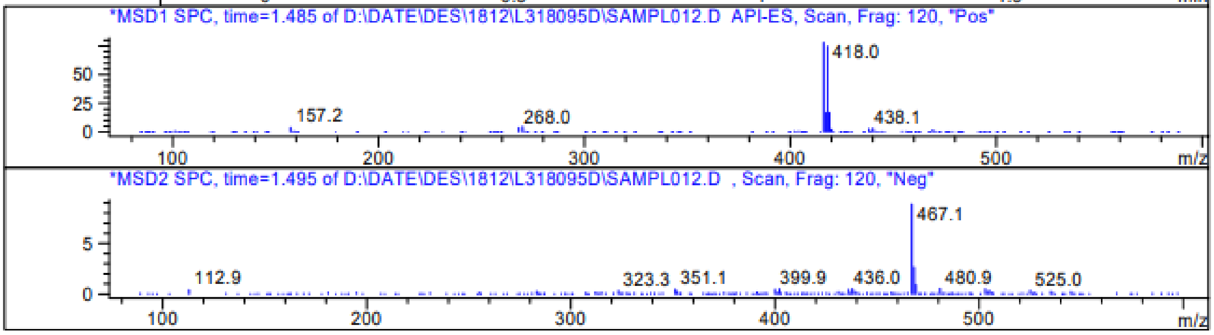

SPC-43 (STL287322, Vitas-M)

Analysis Info

Analysis Name	D:\Data\Kolotyrkina\2020\Iraida\1222002.d	Acquisition Date	22.12.2020 9:41:55
Method	tune_50-1600.m	Operator	BDAL@DE
Sample Name	/NGKO HRM07411	Instrument / Ser#	micrOTOF 10248
Comment	C18H17N3O3 mH 324.1342 calibrant added CH3CN		

Acquisition Parameter

Source Type	ESI	Ion Polarity	Positive	Set Nebulizer	1.0 Bar
Focus	Not active			Set Dry Heater	200 °C
Scan Begin	50 m/z	Set Capillary	4500 V	Set Dry Gas	4.0 l/min
Scan End	1600 m/z	Set End Plate Offset	-500 V	Set Divert Valve	Waste

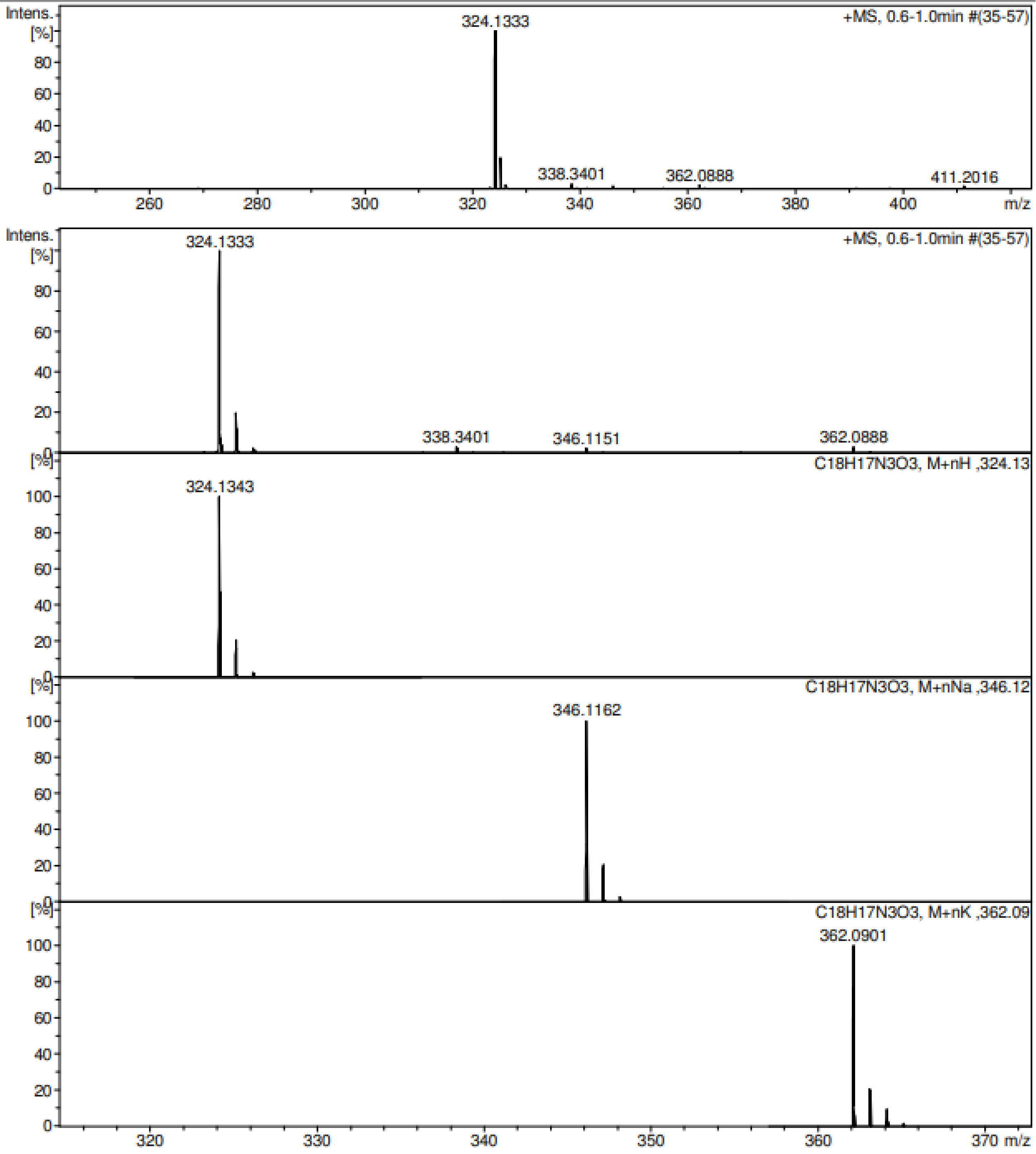

Supplement: Supplementary file 3 — oc2c01190_si_003.pdf [file oc2c01190_si_003.pdf]
